# The Burden of Chronic Pain on Women: A Secondary Analysis of Data From the National Study on Disability (ENDISC) in Chile

**DOI:** 10.1002/ejp.70080

**Published:** 2025-07-15

**Authors:** Marina Carvalho Arruda Barreto, Mariana Arias Ávila, Ricardo Cartes‐Velásquez, Shamyr Sulyvan de Castro

**Affiliations:** ^1^ Institute of Mathematical and Computer Sciences University of São Paulo São Carlos São Paulo Brazil; ^2^ Federal University of São Carlos Department of Physical Therapy São Carlos São Paulo Brazil; ^3^ Facultad de Derecho y Ciencias Sociales Universidad San Sebastián Concepción Chile; ^4^ Federal University of Ceará Public Health Department Fortaleza Ceará Brazil

**Keywords:** biopsychosocial model, chronic pain, disability, functioning, health equity, model disability survey, psychological factors, women

## Abstract

**Background:**

Chronic pain is a prevalent condition that disproportionately affects women, significantly impairing their functioning. Understanding its prevalence and impact is essential for designing effective interventions. This study aimed to determine the prevalence of chronic pain among women and assess its effects on functioning.

**Methods:**

This cross‐sectional analysis utilised data from the National Study on Disability (ENDISC‐2022) in Chile, focusing on women aged 18 years and older. Chronic pain was assessed through self‐reported data, and functioning was evaluated using performance indicators derived from the Model Disability Survey (MDS). Generalised Linear Models (GLM) were applied to examine associations with sociodemographic and psychosocial variables, and stratified analyses compared women with and without chronic pain.

**Results:**

Among the 30,010 participants in the ENDISC study, 14.8% reported chronic pain, with 67.1% of those affected being women. Women experiencing chronic pain exhibited significantly poorer performance scores across all evaluated domains compared to their counterparts without pain. The GLM regression analysis revealed that women with chronic pain had a significantly higher risk of worse functioning scores compared to those without chronic pain.

**Conclusions:**

Sociodemographic factors such as age, education level, and employment status further influenced outcomes, underscoring the vulnerability of women with chronic pain. Chronic pain is a significant and pervasive issue among women in Chile, markedly impairing their functioning and well‐being. Gender‐sensitive approaches and policies are crucial to reducing the burden of chronic pain and improving the quality of life for affected women, particularly those in vulnerable sociodemographic groups.

**Significance:**

The findings highlight the need for targeted interventions that address the multifaceted nature of chronic pain, including its physical, psychological, and social dimensions in women with pain and a more sensitive look at the difficulties encountered by this population, such as work issues.

## 1 Introduction

Chronic pain is characterised as pain persisting for more than 3 months beyond typical healing or associated with chronic pathological processes causing recurrent or continuous pain. It is a widespread condition, a leading cause of medical consultations, and a significant contributor to the Global Burden of Disease‐GBD, resulting in individual suffering and substantial societal costs (Barke et al. 2022; GBD 2021 Musculoskeletal Disorders Collaborators 2023; Mayer et al. 2019). Around 10% to 55.2% of the global population are estimated to experience chronic pain (Fayaz et al. 2016; Goldberg and McGee 2011; Rikard et al. 2023; Santiago et al. 2023).

Chronic pain is increasingly recognised as a distinct condition and classified as a disease in the International Classification of Diseases (Hebert et al. 2024; Treede et al. 2015). Chronic pain ranks among the top 10 causes of disability, exacerbating chronic conditions, and potentially contributing to new ones (Liu et al. 2022; Mullins et al. 2023; Vesal et al. 2024). The 2016–GBD report identified chronic pain as the leading cause of disability worldwide (GBD 2016 Disease and Injury Incidence and Prevalence Collaborators 2017). This condition is multifaceted, influenced by modifiable and non‐modifiable risk factors, and complex mechanisms (Kosek et al. 2016). Chronic pain is closely linked to affective disorders; its persistence elevates the risk of depression and anxiety, while these disorders, along with negative pain beliefs, are associated with worse pain outcomes (Fujiwara et al. 2021; De La Rosa et al. 2022; Mills et al. 2019; Rogers and Farris 2022).

Women are at greater risk than men, though the mechanisms behind these sex differences remain unclear (Rice et al. 2024; Santiago et al. 2023; Vesal et al. 2024). In Brazil, a systematic review reported that chronic pain prevalence is highest among women (Santiago et al. 2023). Additionally, data from a national health survey in Brazil indicate that women exhibit a higher prevalence of chronic spinal pain (Romero et al. 2019). Research on gender and sex differences in pain perception highlights several distinguishing characteristics: women frequently employ maladaptive coping strategies; they exhibit lower pain thresholds and tolerance, often reporting higher pain intensity and discomfort; and they demonstrate distinct sensitivities to analgesics. Evidence suggests that factors such as oestrogens, genetics, and sex‐specific variations in the descending pain modulatory system—including differences in pain‐associated genes—play a role in these disparities (El‐Shormilisy et al. 2015; Hebert et al. 2024; Meng et al. 2015; Rice et al. 2024).

Women are more likely to experience multiple pain site conditions simultaneously, leading to greater disability and psychological distress than isolated pain. Multiple pains also heighten the risk of developing additional conditions (International Association for the Study of Pain 2007). An Australian study found 30% of women reported endometriosis, menopause, and chronic pain as significantly impacting mental well‐being and employment (Victorian Government 2024). Women show lower functioning and higher disability rates than men (Boema et al., Boerma et al. 2016; Cieza et al. 2020; Moreno et al. 2020).

Despite these challenges, women's pain is often underrecognized and inadequately treated. Recently, a series of articles and editorials published highlighted the need for studies that specifically address pain from a gender perspective (Nogrady 2024; The Lancet Rheumatology 2024; Pogatzki‐Zahn et al. 2024). Gender bias and cultural norms in healthcare can lead professionals to downplay or dismiss women's pain, frequently attributing it to psychological factors or hormonal fluctuations rather than thoroughly investigating potential underlying medical conditions. Societal expectations for women to tolerate pain in contexts like menstruation, endometriosis, or menopause further diminish their experiences (eClinicalMedicine 2024; Paganini et al. 2023).

In this context, it is understood that research targeting women is essential to explore the complexity of chronic pain through a comprehensive and multidisciplinary approach, as evidenced by recent discussions and recommendations (Nogrady 2024; Pogatzki‐Zahn et al. 2024; Pickering et al. 2025). This study aimed to determine the prevalence of chronic pain among women in Chile and assess its effects on functioning.

## 2 Methods

This study is a secondary analysis of data from the III National Study of Disability in Chile (III Estudio Nacional de la Discapacidad en Chile—ENDISC III), a nationally representative household survey conducted in Chile in 2022. The study is conducted and funded by the Ministry of Social Development and Family. Data from the ENDISC III was obtained from a public and open‐access repository.

The ENDISC III survey is a nationally representative household survey conducted in Chile to assess disability and functioning across the population. It employed a complex sampling design that was probabilistic, stratified, multistage, and geographically clustered. The sample design was based on the 2017 Chilean Census and included three stages: selection of census districts (primary sampling units), selection of dwelling units, and selection of eligible individuals within households. Stratification considered regional and urban/rural classifications to ensure appropriate representativeness across the country's diverse territories. Data collection occurred between 2022 and 2023 through face‐to‐face interviews using standardised instruments. Aligned with the principles of the Convention on the Rights of Persons with Disabilities, the study reinforces the commitment to promoting inclusion, equality, and respect for human rights by adopting the International Classification of Functioning, Disability and Health (ICF) as a framework to measure functioning and the barriers individuals face.

### 2.1 Survey Population

The III ENDISC survey included a statistically representative sample of 35,536 individuals, of whom 30,010 were adults. For this study, the analysis focused on women with chronic pain aged 18 years or older, resulting in a final sample size of 2978 participants (Ministerio de Desarrollo Social y Familia 2023).

### 2.2 Study Variables

The presence of chronic pain was determined through self‐report by asking participants: “Do you have chronic pain lasting longer than 3 months?” (in Spanish:“¿Tiene usted salud: dolor crónico de duración mayor a tres meses?”) with response options yes (“si”) or no (“no”)^28^.

The assessment of functioning was performance‐based, recognising that performance represents the operationalization of functioning within the context of the ICF. Performance is defined as “carrying out tasks in the usual environment” (Cieza et al. 2018). The performance measure was derived from the Model Disability Survey (MDS) functioning module, used during the data collection process. This variable was constructed based on a set of 12 questions addressing the prompt: “In the past 30 days, how much of a problem has this been for you?” The questions covered the following areas: walking, getting to desired places, taking care of appearance or dressing, using the bathroom, managing health (exercising, eating well, or having meals), taking medications, feeling tired and lacking energy, completing all necessary tasks, remembering important things, doing household chores (sweeping, cooking, cleaning, or taking out the trash), participating in parties, religious events, neighbourhood meetings, or other community activities, using public transportation, and performing tasks required at work or school. Responses were given on a scale from 1 to 5, ranging from no difficulty to extreme difficulty. The total score ranges from 0 to 100, with higher scores indicating worse performance.

The MDS was developed by the World Health Organisation (WHO) and based on the ICF. The primary objectives of the MDS are to provide standardised and comparable estimates of disability prevalence across different countries, to gather data necessary for planning interventions, policies, and programmes for individuals with disabilities, and to furnish indicators for monitoring the implementation of recommendations outlined in the Convention on the Rights of Persons with Disabilities (Cieza et al. 2018).

The sociodemographic variables selected for the study were: age group (18 to 30; 31 to 50; 51 to 65 years, 65 years or older); educational level (illiterate, incomplete primary level, complete primary level, incomplete secondary level, complete secondary level, incomplete higher education, graduated); marital status (with partner; without partner); work situation—worked at least 1 h in the last week (no; yes); individual income (quintile I‐lowest, II, III, IV, V‐highest); self‐rated health (very good, good, fair, bad, very bad).

### 2.3 Statistical Analysis

The characteristics of the studied population were analysed using frequency distributions with 95% confidence intervals (CIs), stratified by the presence or absence of self‐reported chronic pain. Performance means, along with their respective 95% CIs, were presented for the subgroups of women with and without chronic pain, based on sociodemographic characteristics.

The inferential analysis employed Generalised Linear Models (GLMs) with regression to evaluate the comparison of variables, using arithmetic mean ratios (AMRs) and their respective 95% CIs. All study variables were included in the regression model adjustment, along with the presence of other comorbidities reported in the research database. These comorbidities included low vision, deafness, hearing impairment, migraine, COVID‐19, long COVID, diabetes, malnutrition, depression, anxiety, attention deficit disorder, stroke, thyroid disease, skin diseases, urinary incontinence, dementia, epilepsy, autism spectrum disorder, spinal cord injury, cerebral palsy, and Down syndrome. Their inclusion aimed to minimise potential confounding effects on the results. The stepwise method was used to determine the variables in the final model. The variables that presented statistical significance in the bivariate analysis were included in the model and subsequently gradually removed, with a significance threshold of *p* < 0.20 for model fit and *p* < 0.05 for overall model significance. The study employed a sampling design that incorporated stratification and weighting. Consequently, all analyses were performed using the Svy package in Stata 11 (STATA Corp., USA), ensuring the incorporation of appropriate weights into the analysis process.

## 3 Results

The III ENDISC study evaluated a total of 30,010 adults, of whom 4439 (14.8%) reported experiencing chronic pain. Among those with chronic pain, 67.1% (*n* = 2978) were women. Table 1 provides a detailed comparison of the sociodemographic characteristics of women with and without chronic pain. Of the 17,314 women included in the study, 2978 (17.2%) self‐reported chronic pain. Within this group, 35.6% (*n* = 1181) were aged 51–65 years, 42.6% (*n* = 1229) had completed intermediate education, and 51.5% (*n* = 1550) reported not having a partner. Additionally, 62.5% (*n* = 1952) had not worked in the past week, and 22.3% (*n* = 691) were in the lowest income quintile. Regarding self‐rated health, 55.4% (*n* = 1646) of women with chronic pain perceived their health as fair.

**TABLE 1:** Characterisation of the population of women with and without chronic pain.

Variables	Chronic pain						
	No		Yes		p	Total	
	*n* (14336)	% (82.8)	*n* (2.978)	% (17.2%)		*n* (17314)	%
Age groups (years)							
18–30	3190	27.1	175	8.1	*	3365	24.1
31–50	4539	36.2	653	27.0		5192	34.7
51–65	3612	21.3	1181	35.6		4793	23.9
> 65	2723	15.3	911	27.3		3634	17.3
Educational level							
Without formal	229	1.3	75	2.2	*	304	1.4
Incomplete fundamental	61	0.4	2	0.0		63	0.4
Complete fundamental	645	3.7	232	6.9		877	4.2
Incomplete intermediate	2524	15.1	748	22.3		3272	16.2
Complete intermediate	5888	41.6	1229	42.6		7117	41.8
Incomplete superior	1963	15.6	193	7.8		2156	14.3
Complete superior	3026	22.6	499	18.1		3525	21.7
Marital status							
With partner	6142	41.7	1428	48.5	*	7570	42.8
Without partner	8194	58.3	1550	51.5		9744	57.2
Work situation–worked at least 1 h in the last week							
No	8273	45.2	1952	62.5	*	10,225	56.0
Yes	6063	54.8	1026	37.4		7089	44.0
Income (quintiles)							
V (best)	2706	18.5	494	15.5	*	3200	18.1
IV	2753	19.4	641	22.6		3394	19.9
III	2880	20.7	594	20.4		3474	20.7
II	2791	20.1	556	19.2		3347	20.0
I (worse)	3200	21.1	691	22.3		3891	21.3
Health conditions							
Very good health	1180	8.8	36	1.1	*	1216	7.6
Good health	6372	44.6	629	20.1		7001	40.7
Regular health	5554	38.1	1646	55.4		7200	40.8
Bad health	983	6.8	515	17.9		1498	8.6
Very bad health	247	1.6	152	5.5		399	2.2

* *p* < 0.05.

In contrast, among women without chronic pain, 36.2% (*n* = 4539) were aged 31–50 years, 41.6% (5888) had completed intermediate education, and 58.3% (8194) reported not having a partner. A higher proportion of these women (54.8%/*n* = 6063) had worked in the past week, while 21.1% (*n* = 3200) belonged to the lowest income quintile. In terms of self‐rated health, 44.6% (*n* = 6372) of women without chronic pain described their health as good.

Table 2 presents the average performance scores (as an operational measure of functioning) for women with and without chronic pain, analysed in relation to sociodemographic characteristics and self‐reported health. The results indicate that women with chronic pain exhibit significantly lower average performance scores across all evaluated categories. In both groups—women with and without chronic pain—functioning scores worsened with increasing age, decreasing income, and lower education levels. Additionally, women without a partner or employment demonstrated poorer functioning outcomes in both groups.

**TABLE 2:** Average functioning of women with and without chronic pain.

Variables	Chronic Pain				Total	
	No		Yes			
	Mean	95% CI	Mean	CI95%	Mean	95% CI
Age groups (years)						
18–30	15.24	14.48–16.00	28.83	25.88–31.77	15.96	15.21–16.72
31–50	16.55	15.96–17.15	31.46	29.71–33.20	18.40	17.80–19.00
51–65	20.69	19.96–21.42	34.50	33.26–35.74	24.14	23.47–24.80
> 65	32.27	31.27–33.28	43.77	42.14–45.40	35.16	34.29–36.04
Educational level						
Without formal	42.23	37.66–46.80	49.60	43.95–55.26	44.06	40.22–47.89
Incomplete fundamental	40.41	33.90–46.91	41.87	30.61–53.13	40.42	34.00–46.85
Complete fundamental	34.72	32.63–36.80	44.46	40.97–47.95	37.28	35.44–39.11
Incomplete intermediate	24.03	23.08–24.97	38.59	36.97–40.21	27.21	26.33–28.06
Complete intermediate	18.28	17.69–18.87	34.27	32.94–35.59	20.87	20.30–21.44
Incomplete superior	16.59	15.56–17.62	29.98	26.71–33.25	17.74	16.72–18.76
Complete superior	16.38	15.64–17.13	33.23	31.44–35.02	18.61	17.88–19.34
Marital status						
With partner	19.23	18.67–19.79	34.88	33.68–36.07	22.05	21.51–22.58
Without partner	19.63	19.09–20.18	36.59	35.34–37.84	22.06	21.54–22.58
Work situation–worked at least 1 h in the last week						
No	22.10	21.56–22.63	39.06	37.92–40.20	25.10	24.58–25.62
Yes	16.28	15.73–16.84	30.25	29.03–31.47	18.17	17.63–18.71
Income (quintiles)						
V (best)	17.99	17.13–18.84	33.20	31.25–35.16	20.06	19.23–20.88
IV	18.89	17.97–19.82	35.36	33.62–37.10	21.86	20.99–22.73
III	19.65	18.77–20.53	36.02	34.14–37.91	22.21	21.38–23.05
II	20.13	19.26–21.01	36.53	34.50–38.56	22.64	21.80–23.47
I (worse)	20.49	19.68–21.30	37.11	35.23–38.99	23.24	22.44–24.05
Health conditions						
Very good health	5.77	5.00–6.54	18.42	10.42–26.42	6.06	5.27–6.84
Good health	11.91	11.48–12.34	20.93	19.42–22.43	12.62	12.20–13.04
Regular health	26.35	25.79–26.90	35.07	34.13–36.01	28.23	27.73–28.72
Bad health	40.99	39.72–42.26	48.76	46.94–50.58	43.56	42.50–44.63
Very bad	50.02	45.25–54.78	57.85	54.43–61.28	53.09	49.85–56.33

Abbreviation: 95% CI, 95% confidence intervals.

According to the AMR of the generalised linear model (GLM) regression analysis revealed that women with chronic pain had a significantly higher risk of worse functioning scores (adjusted coefficient: 1.07, 95% CI: 1.03–1.11) compared to those without chronic pain. Conversely, self‐reporting health as regular (adjusted coefficient: 0.90, 95% CI: 0.83–0.97), good (adjusted coefficient: 0.56, 95% CI: 0.52–0.61), or very good (adjusted coefficient: 0.33, 95% CI: 0.28–0.38) was identified as a protective factor associated with better functioning outcomes.

## 4 Discussion

The prevalence of chronic pain among adult women in Chile was 17.2%, with the highest prevalence observed in the 51–65 age group, among individuals with high school education, without a partner, and unemployed. Over half of the women with chronic pain rated their health as fair, and they demonstrated significantly worse functioning across all evaluated categories. GLM regression models confirmed a higher risk of poorer functioning among women with chronic pain compared to those without pain.

The elevated prevalence in the 51–65 age group aligns with literature describing this period as a transitional phase marked by biological, psychological, and social changes, including stressors related to health, family, and finances (Borra and Hardy 2023; McGinnis 2018). These factors may exacerbate pain perception and hinder coping mechanisms. Socioeconomic vulnerability, reflected in lower education, limited social support, and financial instability, further contributes to this population's susceptibility to chronic pain. Although causality cannot be inferred due to the study's cross‐sectional design, these findings align with patterns observed in the general population with chronic pain in Brazil and the United States (Carvalho et al. 2018; Rikard et al. 2023).

A study conducted in Brazil using online data collection reported a predominance of female participants (84.60%). The majority of respondents (68.88%) were over 55 years old, 33.79% had attained some level of formal education, and 63.27% reported a monthly income between less than R$999 and R$1999 (Carvalho et al. 2018). In contrast, a study conducted in a rural agricultural city in Chile, which aimed to determine the prevalence of chronic pain—classified as non‐cancer chronic pain, fibromyalgia, and neuropathic pain—found that 66.22% of individuals with pain were women. However, unlike the present findings, the highest prevalence was observed in the 45–54 age group (34.4%), followed by the 55–64 age group (30.4%). Additionally, 56.16% of individuals with chronic pain had completed only primary education. These findings may be attributed to the higher representation of individuals aged 45–54 in the Chilean sample, the overall low educational attainment, and the rural context in which the study was conducted (Durán et al. 2023). A similar finding regarding educational level was reported in a study conducted in Colombia involving individuals with chronic pain due to rheumatic conditions: 64% of the sample were women, and 72.3% had low or medium educational attainment (Londoño et al. 2018).

Women with chronic pain were more likely to be unemployed, and women with chronic pain who were employed reported better functional outcomes. In contrast, a study involving 1378 women with endometriosis from Latin America and Spain reported that 78.1% of participants were employed, which may be explained by the younger mean age of the sample (33.7 ± 7.2 years) (Flores et al. 2024). Employment is a known protective factor for health, as it reduces the risk of absenteeism, disability retirement, and work incapacity (Palermo et al. 2024; Lallukka et al. 2020). Employed women tend to maintain better health and experience a slower decline in health status compared to unemployed women. Job security further mitigates the risk of workforce exit among women with health challenges (Reeves et al. 2014; Schuring et al. 2020). A longitudinal study in England (Palermo et al. 2024) found that health‐related issues, including chronic pain, were significant contributors to early or disability retirement among women aged 50–64 years.

In some cases, women find it difficult to report their painful health condition at work for fear of losing their job or having their treatment changed, especially when it is a stigmatised or “invisible” condition, such as fibromyalgia (Oldfield et al. 2016; Paganini et al. 2023). Thus, although the disease appears to be a reason for job loss, discrimination plays a key role (Palermo et al. 2024; Palstam and Mannerkorpi 2017). These findings highlight the need for targeted interventions and medical support to enable women with chronic pain to remain in the workforce, particularly in sectors with high female representation, as well as the need for discussion on these issues to reduce biases.

In this study, women with chronic pain exhibited worse functioning scores across all evaluated categories compared to those without chronic pain. Performance was used as a proxy for functioning, and findings revealed an average functioning score 7% lower in women with chronic pain. This highlights the significant impact of chronic pain on daily functioning. While no directly comparable studies were identified that focused exclusively on women in population‐level health surveys, existing evidence supports that women generally have lower functioning levels than men (Cieza et al. 2020). In line with this, Mendoza‐Pinto et al. (2024) analysed data from the 2019 Global Burden of Disease study in Latin America and the Caribbean on the impact of musculoskeletal disorders and found that women have more Disability‐Adjusted Life Years (DALYs) than men—a pattern that has been consistently observed since 1990.

A 2018 systematic review highlighted the unique challenges women face in coping with pain while managing household and caregiving responsibilities, which often act as barriers to recovery (Samulowitz et al. 2018). The present findings align with these observations, emphasising the compounded effects of chronic pain on functioning, including caregiving and domestic tasks. This underscores the importance of raising healthcare professionals' awareness about potential gender biases that may lead to underestimating women's pain or providing inadequate treatment (Paganini et al. 2023; Samulowitz et al. 2018; Valenzuela and Cartes‐Velásquez 2019, 2020). A biopsychosocial approach to pain and functioning is crucial, as highlighted by Campbell et al. (2022), advocating contextualised pain assessments to address women's unique experiences.

This study has limitations, including the use of secondary data, which may carry inherent biases, the reliance on self‐reported chronic pain, and the absence of quantitative measures of pain or adjustment for menstrual cycle effects. Reverse causality may also limit causal interpretations. Furthermore, as the information is self‐reported, there is a risk of distortions, such as underestimation or overestimation of results, resulting from possible difficulties in understanding the issues. Nonetheless, the study's strengths include a robust sampling methodology ensuring a representative population in a middle‐income country, adherence to WHO‐recommended data collection methods, and a focus on gender‐specific challenges aligned with Sustainable Development Goal 5 on gender equality.

In conclusion, chronic pain is a significant and pervasive issue among women in Chile, markedly impairing their functioning and well‐being. The findings highlight the need for targeted interventions that address the multifaceted nature of chronic pain, including its physical, psychological, and social dimensions. Gender‐sensitive approaches and policies are crucial to reducing the burden of chronic pain and improving the quality of life for affected women, particularly those in vulnerable sociodemographic groups.

## Author Contributions

This study was designed by M.C.A.B., M.A.Á., R.C.‐V., and S.S.C. The data were analysed by M.C.A.B. and S.S.C., and the results were critically examined by all authors. M.C.A.B. had a primary role in preparing the manuscript, which was edited by M.A.Á., R.C.‐V., and S.S.C. All authors have approved the final version of the manuscript and agree to be accountable for all aspects of the work.

## Conflicts of Interest

The authors declare no conflicts of interest.

## Supporting information


Data S1.

Data S2.
