# Supplementary material for: The Burden of Chronic Pain on Women: A Secondary Analysis of Data From the National Study on Disability (ENDISC) in Chile
Source: Eur J Pain. 2025 Jul 15;29(7):e70080. doi: 10.1002/ejp.70080 (PMC12261274; doi:10.1002/ejp.70080)
Supplement: Supplementary file 1 — Data S1. [file EJP-29-0-s001.pdf]

Ministerio de Desarrollo Social y Familia
Gobierno de Chile

SENADIS
Ministerio de Desarrollo Social y Familia
Gobierno de Chile

SENAMA
Ministerio de Desarrollo Social y Familia
Gobierno de Chile

ENDIDE

Encuesta de Discapacidad y Dependencia 2022

Documento Metodológico

Septiembre 2023

(*) Documento elaborado por la división Observatorio Social de la Subsecretaría de Evaluación Social y el departamento de Evaluación y Estudios del Servicio Nacional de la Discapacidad.

Contenido

INTRODUCCIÓN 4

I. METODOLOGÍA DEL DISEÑO MUESTRAL 5

1. Antecedentes del diseño de ENDIDE 5
    1.1. Objetivos de estimación 5
    1.2. Población Objetivo 5
    1.3. Unidad de Análisis 5
    1.4. Dominios de estudio 6
    1.5. Objetivos de precisión 6
2. Marco Muestral de ENDIDE 7
    2.1. Definición del Marco Muestral 7
    2.2. Actualización del Marco Muestral: Empadronamiento 8
3. Diseño Muestral de ENDIDE 9
    3.1. Estrategia muestral 9
    3.2. Tamaño muestral 9
    3.2.1. Estimación del tamaño muestral 10
    3.2.2. Método de selección 12
4. Diseño del Factor de Expansión ENDIDE 14
    4.1. Factor de expansión de primera fase 15
    4.2. Factor de expansión de segunda fase 17
    4.3. Factor de expansión de primer y segunda fase 18
    4.3.1. Corrección por no elegibilidad 18
    4.3.2. Corrección por no respuesta 19
    4.3.3. Suavizamiento del factor de expansión 20
    4.4. Errores efectivos 24

II. METODOLOGÍA DE MEDICIÓN DE LA DISCAPACIDAD 28

1. Conceptualización de la Discapacidad 28
2. Medición de la Discapacidad en ENDIDE 30
3. El Modelo de Crédito Parcial de Rasch 33
4. Uso del Modelo de Crédito Parcial de Rasch para la medición de la Discapacidad 33

4.1 Estimación del Modelo de Crédito Parcial de Rasch 34
4.2 Evaluación de Supuestos y Ajuste del Modelo de Crédito Parcial de Rasch 35
a. Unidimensionalidad 35
b. Independencia local 36
c. Monotonicidad 36
d. Ordenamiento estocástico 37
e. Funcionamiento diferencial del ítem e invarianza (de grupo) 39
f. Bondad de ajuste del modelo y confiabilidad 40
5. Determinación de Niveles de Dificultad 41
6. Determinación de la Prevalencia de Discapacidad 43

ANEXOS 44
BIBLIOGRAFÍA 45

Documento Metodológico[1]

Encuesta Nacional de Discapacidad y Dependencia, ENDIDE 2022

INTRODUCCIÓN

La Encuesta Nacional de Discapacidad y Dependencia 2022, ENDIDE 2022, es un proyecto del Ministerio de Desarrollo Social y Familia (MDSF), liderado por la División Observatorio Social de la Subsecretaría de Evaluación Social, en colaboración con el Servicio Nacional de la Discapacidad (Senadis) y el Servicio del Adulto Mayor (Senama).

El objetivo principal de ENDIDE es estimar la prevalencia de la discapacidad y dependencia en Chile, entregando datos de caracterización fundamentales para el diseño de políticas públicas en materia de salud, calidad de vida e inclusión social de la población.

El levantamiento de los datos fue adjudicado, mediante licitación pública, al Centro de Microdatos de la Universidad de Chile. El trabajo de campo de la encuesta comenzó el 22 de abril y finalizó el 26 de agosto, extendiéndose por un total de 18 semanas.

El presente documento tiene por objetivo presentar antecedentes metodológicos relevantes de ENDIDE 2022, con foco en dos tópicos: 1) Las características metodológicas del diseño muestral de la Encuesta y 2) La metodología de medición de la discapacidad. De esta manera, el documento se estructura en dos secciones enfocadas en ambos temas respectivamente.

[1] Documento elaborado por la división Observatorio Social de la Subsecretaría de Evaluación Social y el departamento de Evaluación y Estudios del Servicio Nacional de la Discapacidad.

I. METODOLOGÍA DEL DISEÑO MUESTRAL

Esta sección tiene por objeto presentar la metodología definida para el diseño muestral de ENDIDE, abarcando los siguientes apartados: antecedentes generales del diseño, características del marco muestral, diseño muestral y finalmente la metodología de construcción de los factores de expansión.

1. Antecedentes del diseño de ENDIDE

Para definir las características del diseño muestral de ENDIDE se consideraron los siguientes antecedentes y requerimientos:

1.1. Objetivos de estimación

- Determinar la proporción de personas de 18 años y más con discapacidad y en situación de dependencia a nivel nacional, regional y por zona urbano y rural.
- Determinar la proporción de personas de 60 años y más con discapacidad y en situación de dependencia a nivel nacional, regional y por zona urbano y rural.
- Determinar la proporción de niños, niñas y adolescentes de 2 a 17 años con discapacidad.

1.2. Población Objetivo

La población objetivo de la encuesta son las personas de 2 años o más que residen en viviendas particulares en el territorio nacional, excluyendo las áreas de difícil acceso[2] excluidas del Marco muestral del INE para la encuesta Casen en Pandemia 2020.

1.3. Unidad de Análisis

La unidad de información de ENDIDE corresponde a las personas de 2 años o más que han sido seleccionadas para el levantamiento de esta encuesta.

[2] Las áreas de difícil acceso (ADA) corresponden a zonas geográficas que, por razones de orden climático, topográfico o por ausencias de vías y medios de comunicación expeditos permanecen aisladas parcial o totalmente en el año, lo cual ha impedido su correcta incorporación al marco muestral del INE. Las ADA's incluyen a las comunas de General Lagos, Colchane, Ollagüe, Juan Fernández, Isla de Pascua, Cochamó, Chaitén, Futaleufú, Hualaihué, Palena, Lago Verde, Guaitecas, O'Higgins, Tortel, Laguna Blanca, Río Verde, San Gregorio, Cabo de Hornos (Ex - Navarino), Antártica, Primavera, Timaukel, Torres del Paine.

1.4. Dominios de estudio

Los niveles de estimación o dominios de estudio de ENDIDE, para los cuales la muestra fue diseñada, son los siguientes:

- Población adulta (18 años o más): Nacional, por zona geográfica urbana y rural, y regional.
- Población de personas mayores (60 años o más): Nacional, por zona geográfica urbana y rural, y regional.
- Niños, Niñas y Adolescentes (2 a 17 años): Nacional.

1.5. Objetivos de precisión

Los criterios de precisión se establecieron definiendo la variable de interés como la proporción de las personas con discapacidad. Así, los errores muestrales esperados se fijaron en:

<u>Población adulta (18 o más años):</u>
- Error Relativo a nivel nacional: 1,7%.
- Error Absoluto a nivel nacional: 0,3%.
- Error Relativo y Absoluto a nivel nacional urbano: 1,9% y 0,4%, respectivamente.
- Error Relativo y Absoluto a nivel nacional rural: 5,6% y 1,2%, respectivamente.
- Error Relativo: menor al 15% a nivel regional, con excepción de la región de Aysén[3].
- Error Absoluto: menos de 5% a nivel regional.

<u>Población de personas mayores (60 años y más):</u>
- Error Relativo a nivel nacional: 1,3%.
- Error Absoluto a nivel nacional: 0,5%.
- Error Relativo y Absoluto a nivel nacional urbano: 1,6% y 0,6%, respectivamente.
- Error Relativo y Absoluto a nivel nacional rural: 3,3% y 1,3%, respectivamente.
- Error Relativo: menor al 15% a nivel regional, con excepción de la región de Aysén (29,1%).
- Error Absoluto: menos de 7% a nivel regional, con excepción de la región de Aysén (7,2%).

<u>Niños, Niñas y Adolescentes (2 a 17 años):</u>
- Error Relativo: 25,2% a nivel nacional.
- Error Absoluto: 1,5% a nivel nacional.

[3] Error estimado de 30,1%

2. Marco Muestral de ENDIDE

2.1. Definición del Marco Muestral

Tras evaluar las ventajas y desventajas del uso de distintos marcos muestrales para la Encuesta, se definió el uso del precontacto logrado de viviendas de Casen en Pandemia 2020, compuesto por 86.189 viviendas.

El precontacto de Casen en Pandemia 2020 corresponde a la primera etapa presencial del proceso de recolección de Casen en Pandemia 2020[4] cuyo objetivo principal fue recuperar el número telefónico de contacto, para que en una segunda etapa la recolección se realizara de manera telefónica. Dentro de las principales ventajas del uso de este marco se destacan:

- *Elegibilidad "conocida" de las viviendas:* Todas las viviendas que son parte del precontacto logrado fueron viviendas elegibles para la encuesta Casen en Pandemia 2020 (viviendas particulares), y fueron visitadas en septiembre de 2020.
- *Información de la composición de un porcentaje importante de las viviendas:* Del total de viviendas logradas en el precontacto, 62.540 son parte de la muestra lograda en Casen en Pandemia 2020 (72,6%), contando con información respecto a su composición y a las características de sus integrantes.
- *Disponibilidad de número telefónico de contacto:* Dentro del proceso de precontacto de Casen en Pandemia 2020 se registró un número de contacto telefónico para todas las viviendas logradas.

Si bien se esperaba un porcentaje de desactualización respecto a la elegibilidad y a los números telefónicos de contacto de las viviendas[5], el uso del precontacto logrado de Casen en Pandemia 2020 permitió la construcción de un marco de personas para la selección de la muestra de los distintos grupos de población objetivo, de una manera más eficiente y ajustada al presupuesto disponible para la Encuesta.

[4] El marco muestral de Casen en Pandemia 2020 consideró: El marco muestral de manzanas, para área urbana y Resto de Áreas Urbanas (RAU) actualizado con información de Precenso 2016 (MMM 2016) y el Marco muestral de secciones para área rural y Resto de Áreas Urbanas (RAU) actualizado con información de Precenso 2016 (MMS 2016). La actualización de ambos marcos mantuvo la estructura y conformación del marco construido en base al Censo 2002.

[5] La desactualización esperada se atribuye al tiempo que transcurrió entre el trabajo de campo del precontacto de Casen en Pandemia 2020 (septiembre y octubre 2020) y el trabajo de campo de la Encuesta de Discapacidad y Dependencia (abril a agosto 2022).

2.2. Actualización del Marco Muestral: Empadronamiento

Debido a que ENDIDE es una encuesta dirigida a personas, que requiere tamaños muestrales diferenciados por tramo de edad para lograr los niveles de representatividad esperados para sus principales indicadores, fue necesario construir un marco muestral de personas con información respecto a su edad. Para ello, previo a la selección de la muestra, se llevó a cabo un proceso de empadronamiento del marco del precontacto logrado de Casen en Pandemia 2020, registrando la información de composición de las viviendas y las principales características de sus integrantes (sexo y edad).

El proceso de empadronamiento fue realizado en una modalidad mixta entre los meses de noviembre 2021 y febrero 2022. En primera instancia, se realizó un empadronamiento 100% telefónico y posteriormente se combinó esta modalidad con la modalidad presencial, en función de los escenarios de logro de cada región.

Tras finalizar el proceso de empadronamiento, se construyó el marco muestral de ENDIDE, el cual contó con la distribución presentada en Tabla 1.

Tabla 1: Número de personas del Marco muestral de ENDIDE, según región y zona.

Zona/Región	Tramo de edad			Total	%
	2 a 17 años	18 a 59 años	60 años y más		
Urbano				160.997	84,20%
Rural				30.215	15,80%
Arica y Parinacota	1.651	4.323	1.606	7.580	4,00%
Tarapacá	1.966	4.851	1.420	8.237	4,30%
Antofagasta	1.988	5.077	1.341	8.406	4,40%
Atacama	2.016	4.944	1.835	8.795	4,60%
Coquimbo	1.627	4.422	1.882	7.931	4,10%
Valparaíso	3.583	9.993	4.578	18.154	9,50%
Metropolitana	7.289	21.679	7.931	36.899	19,30%
O'Higgins	2.579	7.383	3.082	13.044	6,80%
Maule	2.558	7.398	3.294	13.250	6,90%
Ñuble	1.171	3.438	1.722	6.331	3,30%
Biobío	3.592	10.492	4.678	18.762	9,80%
La Araucanía	2.618	6.937	2.933	12.488	6,50%
Los Ríos	1.914	5.162	2.296	9.372	4,90%
Los Lagos	2.310	6.572	2.731	11.613	6,10%
Aysén	1.150	2.819	1.019	4.988	2,60%
Magallanes	1.045	3.002	1.315	5.362	2,80%

Zona/Región	Tramo de edad			Total	%
	2 a 17 años	18 a 59 años	60 años y más		
Total	39.057	108.492	43.663	191.212	100,00%

Fuente: elaboración en base a Proyecto ENDIDE 2022

3. Diseño Muestral de ENDIDE

3.1. Estrategia muestral

El diseño muestral de ENDIDE corresponde a un diseño probabilístico, estratificado y bifásico. La primera fase corresponde a la probabilidad de selección de las viviendas en el precontacto logrado de Casen en Pandemia 2020. Mientras que la segunda fase corresponde a la probabilidad de selección en el marco muestral de ENDIDE.

Para la segunda fase del diseño, se trabajó con 96 estratos explícitos de muestreo conformados por la combinación de las variables: tramo de edad (2 a 17 años, 18 a 59 años y 60 años y más), región y zona. Posteriormente, al interior de cada uno de estos estratos, se realizó la selección de las personas en forma sistemática y con igual probabilidad al interior de cada tramo de edad. Adicionalmente, para efectos de ordenamiento, se definieron estratos implícitos definidos por: unidad primaria de muestreo (UPM), vivienda y orden de la persona dentro de la vivienda empadronada en ENDIDE.

3.2. Tamaño muestral

A partir de los objetivos de estimación y dominios de estudio, se definió una muestra objetivo nacional de 35.730 personas de 2 años o más. Para compensar las pérdidas asociadas a la no respuesta, el diseño de la muestra consideró un porcentaje de sobremuestreo que permitiera asegurar el cumplimiento de la muestra objetivo.

Para estimar el tamaño muestral con sobremuestreo inicialmente se consideró como referencia la tasa de no respuesta de Casen 2017, última encuesta levantada presencialmente a cargo del MDSF. Sin embargo, debido al efecto de la pandemia Covid-19 y al uso del marco Casen en Pandemia 2020 en otras encuestas del MDSF (Encuesta de Bienestar Social y Encuesta Covid-19 ambas telefónicas), se anticipó un posible incrementó en el rechazo de algunas viviendas y en las tasas de no respuesta de la encuesta. Frente a ello, la estimación de la muestra con sobremuestreo consideró la tasa de no logro[6] de Casen en Pandemia 2020, obteniendo una muestra total de 58.963 personas de 2 años y más.

[6] La tasa de no logro utilizada considera las etapas de precontacto y el levantamiento telefónico de Casen en Pandemia 2020.

3.2.1. Estimación del tamaño muestral

El tamaño de la muestra fue calculado considerando los diferentes tramos de edad, niños, niñas y adolescentes de 2 a 17 años, personas adultas de 18 a 59 años y personas mayores de 60 años y más, de manera independiente.

Para determinar el tamaño muestral requerido para cada región se consideró el número de personas en cada tramo de edad, fijando un error propuesto de 4% inicial y realizando un ajuste de la muestra según la distribución de la población por región del Censo 2017 en ese tramo de edad. La variable de diseño fue definida como la proporción de personas con discapacidad por región determinada en el Segundo Estudio Nacional de la Discapacidad 2015 (ENDISC II).

Asumiendo el muestreo aleatorio simple para determinar el tamaño de la muestra se tiene:

$$(1) \qquad n = \frac{N * Z^2_{(1-\propto/_2)} * S^2}{(N-1) * e^2 + Z^2_{(1-\propto/_2)} * S^2}$$

donde,

N : Número de personas en viviendas particulares en el marco muestral de ENDIDE.
$Z^2_{(1-\propto/_2)}$: Percentil de nivel $(1 - \alpha/2)$ de la distribución Normal, correspondiente a la estimación del intervalo de $(1 - \alpha)$ de confianza.
S^2: Cuasi varianza usando la variable de discapacidad ENDISC 2015.
e^2: Error absoluto esperado propuesto

El tamaño estimado de la muestra objetivo para cada tramo de edad y región se presenta en la Tabla 2. El tamaño estimado para la muestra objetivo para cada tramo de edad y zona se presenta en la Tabla 3.

Tabla 2: Distribución del tamaño de muestra objetivo cada tramo de edad por región

Región	Tramo de edad				Total personas 2 y más años
	Personas de 2 a 17 años[7]	Personas de 18 a 59 años	Personas de 60 años y más	Total personas 18 y más	
Arica y Parinacota	200	650	300	950	1.150
Tarapacá	200	650	220	870	1.070

[7] La distribución regional de la muestra de niños, niñas y adolescentes (NNA) de 2 a 17 años solo fue realizada de manera orientativa y para asegurar presencia de NNA en cada región, debido a que para este tramo de edad solo se esperaba representatividad a nivel nacional.

Región	Tramo de edad				Total personas 2 y más años
	Personas de 2 a 17 años[7]	Personas de 18 a 59 años	Personas de 60 años y más	Total personas 18 y más	
Antofagasta	250	1.100	340	1.440	1.690
Atacama	200	700	340	1.040	1.240
Coquimbo	280	1.100	400	1.500	1.780
Valparaíso	450	2.500	900	3.400	3.850
Metropolitana	1.300	5.000	2.000	7.000	8.300
O'Higgins	280	1.300	450	1.750	2.030
Maule	350	1.490	510	2.000	2.350
Ñuble	200	700	320	1.020	1.220
Biobío	400	2.250	800	3.050	3.450
La Araucanía	300	1.400	500	1.900	2.200
Los Ríos	290	600	290	890	1.180
Los Lagos	300	1.190	360	1.550	1.850
Aysén	200	550	350	900	1.100
Magallanes	200	700	370	1.070	1.270
**Total**	**5.400**	**21.880**	**8.450**	**30.330**	**35.730**

Fuente: elaboración en base a Proyecto ENDIDE 2022

Tabla 3: Distribución del tamaño de muestra objetivo cada tramo de edad por zona

Zona	Tramo de edad				Total personas 2 y más años
	Personas de 2 a 17 años[8]	Personas de 18 a 59 años	Personas de 60 años y más	Total personas 18 y más	
Urbano	4.703	19.015	7.240	26.255	30.958
Rural	697	2.865	1.210	4.075	4.772
**Total**	**5.400**	**21.880**	**8.450**	**30.330**	**35.730**

Fuente: elaboración en base a Proyecto ENDIDE 2022

A partir del tamaño de la muestra objetivo por tramo de edad y región, se estimó el tamaño de la muestra con sobremuestreo, considerando una tasa de no respuesta nacional del 39,3% correspondiente a la tasa de no logro de Casen en Pandemia 2020. A nivel regional, la tasa de no

[8] La distribución regional de la muestra de niños, niñas y adolescentes (NNA) de 2 a 17 años solo fue realizada de manera orientativa y para asegurar presencia de NNA en cada región, debido a que para este tramo de edad solo se esperaba representatividad a nivel nacional.

respuesta utilizada varió entre el 35,6% y el 42,1%. La Tabla 4 presenta la distribución del tamaño de muestra con sobremuestreo para cada tramo de edad y región, así como la tasa considerada para la estimación de sobremuestreo en cada región.

Cabe destacar que la estimación de la muestra con sobremuestreo fue obtenida de manera redondeada en cada tramo de edad por región, obteniendo la muestra total regional a partir de su suma.

Tabla 4: Distribución del tamaño de muestra con sobremuestreo cada tramo de edad por región

Región	Tasa de No Logro Casen en Pandemia 2020	2 a 17 años	18 a 59 años	60 años y más	Total Personas	Total Viviendas
Arica y Parinacota	40,1%	334	1.086	501	1.921	1.391
Tarapacá	39,5%	331	1.074	364	1.769	1.282
Antofagasta	40,7%	422	1.855	573	285	1.801
Atacama	38,5%	325	1.138	553	2.016	1.498
Coquimbo	38,6%	456	1.793	652	2.901	1.821
Valparaíso	39,4%	743	4.125	1.485	6.353	4.367
Metropolitana	42,1%	2.245	8.635	3.454	14.334	8.653
O'Higgins	37,4%	447	2.075	718	324	253
Maule	37,0%	556	2.365	809	373	2.563
Ñuble	38,6%	326	1.141	521	1.988	1.364
Biobío	37,5%	640	3.602	1.281	5.523	3.758
La Araucanía	38,7%	490	2.285	816	3.591	2.505
Los Ríos	38,8%	473	980	473	1.926	137
Los Lagos	38,2%	485	1.925	582	2.992	2.237
Aysén	35,6%	311	854	543	1.708	1.125
Magallanes	40,1%	334	1.169	618	2.121	1.377
**Total**	**39,3%**	**8.918**	**36.102**	**13.943**	**58.963**	**39.642**

Fuente: elaboración en base a Proyecto ENDIDE 2022

3.2.2. Método de selección

Una vez construido el marco de personas a partir del empadronamiento del precontacto Casen en Pandemia 2020, se construyó el marco muestral de selección de ENDIDE considerando a todas las personas desde los 2 años. La selección de personas fue realizada por medio de una estrategia aleatoria sistemática, tras ordenarlas en cada estrato por unidad primaria de muestreo (UPM), vivienda y orden de la persona dentro de la vivienda empadronada en ENDIDE. Una vez ordenado el estrato, con la

información geográfica, se procedió a realizar la selección en forma sistemática y con igual probabilidad dentro de cada estrato de muestreo definido.

Paso 1

El sorteo entonces consistió en definir en cada estrato r un salto, calculado como:

$$(2) \qquad salto_r = \frac{n_r^{marco}}{n_r^{sobremuestreo}}$$

donde,
$n_r^{sobremuestreo}$ el número de personas a seleccionar con sobremuestreo por estrato r
n_r^{marco} el número de personas en el marco de ENDIDE por estrato r

Paso 2

El punto de arranque aleatorio fue calculado como:

$$(3) \quad arranque_r = ceil\ ((n_r^{marco} - \ salto_r * \ n_r^{sobremuetreo} + 1) * uniform())$$

siendo,
$uniform()$ el número aleatorio de una distribución uniforme
$ceil()$ es una función de aproximación con redondeo hacia arriba, se fija una semilla, que corresponde al día y mes del sorteo (se fija el resultado de la distribución uniforme en cada estrato r).

Paso 3

Posteriormente, al interior de cada estrato r se ordenó, como ya se mencionó, a cada persona del marco muestral por unidad primaria de muestreo (UPM), vivienda y orden de la persona dentro de la vivienda empadronada en ENDIDE. Respetando este orden se creó un índice para cada persona k en el estrato r, que se denota por $n_{r,k}$.

El procedimiento de selección a cada individuo cumple con el siguiente criterio:

$$(4) \quad n_{r,k} = arranque_r + \ salto_r * (i-1) \qquad \forall\, i\ \in\ [1,\ n_r^{sobremuestreo}]$$

El procedimiento de selección presentado se realizó en primera instancia sobre todas las personas del marco muestral en los grupos de personas de 18 años y más (18 a 59 años y 60 años y más). Posteriormente, para seleccionar al grupo de niños, niñas y adolescentes de 2 a 17 años, se utilizó el

mismo algoritmo, pero realizando la selección sobre las viviendas en que ya se había seleccionado una persona adulta.

Como resultado de este procedimiento se seleccionaron 58.963 personas en 39.642 viviendas (muestra con sobremuestreo). En el 39,0% de las viviendas de la muestra se seleccionó a más de una persona, la Tabla 5 presenta la distribución del número de personas seleccionadas por vivienda.

Tabla 5: Distribución del número de personas seleccionadas por vivienda

Número de personas seleccionadas	Número de viviendas	Porcentaje
1	24.192	61,03%
2	12.252	30,91%
3	2.647	6,68%
4	450	1,14%
5	82	0,21%
6	17	0,04%
7	2	0,01%

Fuente: elaboración en base a Proyecto ENDIDE 2022

4. Diseño del Factor de Expansión ENDIDE

Para que las encuestas con diseño muestral probabilístico tengan validez sobre el comportamiento de la población objetivo, se utiliza un ponderador o peso en la estimación de cualquier variable de interés, dicho ponderador expresa el número de personas de la población que representa un individuo que participa en dicha encuesta, y se conoce como "factor de expansión". Así, el factor de expansión permite garantizar que las estimaciones sean insesgadas y consistentes, que el error de muestreo sea pequeño condicionado al diseño muestral y al tamaño muestral, y que los errores de cobertura sean corregidos.

Como fue señalado previamente, ENDIDE posee un diseño muestral bifásico o en dos fases, donde la primera fase corresponde a las viviendas logradas en el precontacto de Casen en Pandemia 2020, y la segunda fase corresponde a un diseño muestral estratificado, considerando el estrato tramo de edad (2 a 17 años, 18 a 59 años y 60 años o más), región y zona.

Dado lo anterior, el factor de expansión de ENDIDE se construye considerando un **factor de expansión de primera fase**, el que proviene de la probabilidad de selección de la vivienda proveniente del diseño muestral del precontacto de Casen en Pandemia 2020, y **un factor de expansión de segunda fase**, que refleja cuántas personas en la población representan las personas de 2 años y más seleccionadas en la muestra de ENDIDE. Posteriormente, se estima el producto del **factor de primera y segunda fase** al cual se le aplican varios **ajustes**: El primer ajuste es un *ajuste por no elegibilidad* el que busca corregir sacando

aquellas personas que no pertenecen a la población objetivo ya definida. El segundo ajuste corresponde a un *ajuste por no respuesta*, que minimiza el sesgo en las estimaciones que se genera por la ausencia de respuesta en las personas que forman parte de la muestra. Adicionalmente a estos ajustes, se lleva a cabo **un suavizamiento** del factor de expansión el que tiene como objetivo centrar los valores que sean considerados atípicos. Y, por último, **la calibración**, que permite mantener la consistencia con las proyecciones de población desarrolladas por el INE y mejorar las propiedades estadísticas de los estimadores utilizados.

A continuación, se describe la metodología para la construcción de cada ponderador utilizado en el cálculo del factor de expansión de ENDIDE.

4.1. Factor de expansión de primera fase

El cálculo de la probabilidad de selección de primera fase de la ENDIDE da cuenta del diseño muestral del precontacto de la encuesta Casen en Pandemia 2020, considerando la probabilidad de selección de las viviendas, que constan de dos etapas:

i) la selección del conglomerado (UPM) y
ii) la selección de la vivienda

Posteriormente se corrige por no elegibilidad y no respuesta, con ello se obtiene la información de todas las personas que pertenecen a las viviendas que respondieron el precontacto de la encuesta Casen en Pandemia 2020.

El **ponderador de selección de los conglomerados** corregido w'_{hi} del precontado de Casen en Pandemia 2020, está dado por:

$$(5) \qquad w'_{hi} = \widehat{R_h} \cdot w_{hi}$$

Donde,

$$(6) \qquad \hat{R}_h = \frac{M_h}{\sum_{i \in \Omega_h} w_{hi} * M_{hi}}$$

$\widehat{R_h}$ es el ajuste por omisión de conglomerados por estrato h.
M_h es el número de viviendas en el estrato h.
w_{hi} es el ponderador de selección base de la unidad primaria de muestreo o conglomerado i (sección o manzana) del estrato h.
Ω_h es el conjunto de conglomerados seleccionados en el estrato h.

M_{hi} es el número de viviendas del conglomerado i del estrato h.
i es el índice de la unidad primaria de muestreo o conglomerado (manzana o sección).
h es el índice del estrato de muestreo, o área (urbana, RAU o rural) de una comuna.

$$(7) \quad w_{hi} = \begin{cases} \frac{M_h}{n_h * M_{hi}} & , Si\ unidad\ i\ es\ una\ sección \\ \frac{N_{hg}}{n_{hg}} & , Si\ unidad\ i\ es\ una\ manzana \end{cases}$$

n_h es el número de secciones seleccionadas en el estrato h.
N_{hg} es el número de manzanas según el MMM2016, en el grupo de tamaño g del estrato h.
n_{hg} es el número de manzanas en la muestra, seleccionadas en el grupo de tamaño g del estrato h.
El **ponderador de selección de la vivienda del precontacto de Casen en Pandemia 2020 corregido por elegibilidad desconocida** incorporando el ajuste por omisión de conglomerado, al diferenciarlo por el Marco desde el cual fue seleccionada la vivienda, se expresa en la siguiente fórmula:

$$(8) \quad w'_{hij} = \widehat{R_{h,know}} \cdot w_{hij}$$

Donde w_{hij}' es el ponderador de selección de viviendas corregido por elegibilidad y $\widehat{R_{h,know}}$ es la proporción de los pesos a redistribuir y se calcula como la razón entre dos estimaciones realizadas en base al ponderador de selección de viviendas.

Siendo:

$$(9) \quad \widehat{R_{h,know}} = \frac{\sum_{i \in \Omega_h} \sum_{j \in \theta_i} w_{hij}}{\sum_{i \in \Omega_h} \sum_{j \in \theta_{i,know}} w_{hij}}$$

θ_i es el conjunto de viviendas j seleccionadas en el conglomerado i.
$\theta_{i,know}$ es el conjunto de viviendas j seleccionadas en el conglomerado i y clasificadas como elegibles o no elegibles.
Ω_h es el conjunto de estratos h (áreas geográficas urbana/rural).
w_{hij} es el ponderador de selección de la vivienda j en el conglomerado i del estrato h.

A partir de estos datos se procede a realizar la corrección por no respuesta.

El **ponderador de selección corregido por no respuesta** w_{hij}^{NR} del precontacto de Casen en Pandemia 2020 se expresa como,

$$(10) \quad w_{hij}^{NR} = \widehat{R_{g,R}} * w'_{hij}$$

Este ponderador se asigna a las viviendas elegibles entrevistadas. A las viviendas elegibles no entrevistadas se asigna un valor blanco (“missing”). Siendo:

$$(11) \qquad \widehat{R_{g,R}} = \frac{\sum_{i\epsilon\phi_g}\sum_{j\epsilon\theta_{i,eleg}} w'_{hij}}{\sum_{i\epsilon\phi_g}\sum_{j\epsilon\theta_{i,eleg,R}} w'_{hij}}$$

Donde, el numerador corresponde a la estimación del total de viviendas en la población y el denominador corresponde a la estimación del total de viviendas con elegibilidad conocida (viviendas elegibles y no elegibles). Ambas estimaciones se realizan con el ponderador de selección de viviendas, $\widehat{R_{g,R}}$

Siendo:
g es el índice del grupo para la corrección de no respuesta.
ϕ_g es el conjunto de conglomerados asignadas al veintil g.
$\theta_{g,eleg}$ es el conjunto de viviendas pertenecientes a la unidad i y catalogadas como elegibles.
$\theta_{g,eleg,R}$ es el conjunto de viviendas pertenecientes a la unidad i, catalogadas como elegibles y que responden la encuesta.

Luego, se sabe que este ponderador corresponde al de la primera fase, por tanto:

$$(12) \qquad F1_{hij} = w_{hij}^{NR} = \widehat{R_{g,R}} * w'_{hij}$$

4.2. Factor de expansión de segunda fase

A partir de lo anterior y conociendo la cantidad de personas de 2 años y más que pertenecen a las viviendas logradas en el precontacto de la encuesta Casen en Pandemia 2020, se continua con el ponderador de selección de las personas en el estrato:

$$(13) \qquad F2_{r,k} = \frac{M_{r,k}}{m_{r,k}}$$

Donde,
$m_{r,k}$ es el total de personas k seleccionadas en la encuesta ENDIDE por tramo de edad, región y zona (estrato r).
$M_{r,k}$ es el total de personas k disponibles por tramo de edad, región y zona (estrato r), según el levantamiento del marco muestral de ENDIDE.

4.3. Factor de expansión de primer y segunda fase

El producto de la selección de la primera fase y segunda fase representa los factores teóricos de selección de la unidad (personas) en ENDIDE.

$$(14) \quad w_{r,k} = F1_{hij} * F2_{r,k}$$

4.3.1. Corrección por no elegibilidad

El ajuste o corrección por no elegibilidad tiene como objetivo corregir los posibles errores de haber incluido en la muestra a personas que no corresponden al universo de estudio.
Como se ha mencionado antes, el objetivo de ENDIDE es estudiar la población que reside en viviendas particulares, por ello, no deben considerarse para fines de análisis a las personas que no forman parte de la población objetivo de la encuesta, llamados también no elegibles, como, por ejemplo, viviendas desocupadas, viviendas de veraneo, entre otras.
Así, se corrige o ajusta dentro de las unidades seleccionadas, reemplazando con missing aquellas que tienen un Código de Disposición Final de Casos[9] asociado a "No Elegibilidad" en el resultado del trabajo de terreno de ENDIDE. El resultado de este análisis es presentado en la Tabla 6.

Tabla 6: Número de personas según relación de elegibilidad, levantamiento ENDIDE

Estado	Personas	Porcentaje
Personas elegibles que respondieron	35.536	60,2%
Personas elegibles que no respondieron	21.033	35,7%
Personas no elegibles	2.394	4,1%
**Total**	**58.963**	**100,0%**

Fuente: elaboración en base a Proyecto ENDIDE 2022

De la muestra total de ENDIDE, 1.957 adultos no son elegibles, y 437 niños, niñas y adolescentes tampoco lo son, con esto el total de personas no elegibles alcanza las 2.394.

El ajuste por no elegibilidad reparte los ponderadores o factores de expansión de diseño de las unidades "No Elegibles" a aquellas con elegibilidad conocida.

[9] En Anexo 1 se presentan los códigos de disposición final de casos utilizados en el Levantamiento de ENDIDE.

4.3.2. Corrección por no respuesta

Las encuestas en general adolecen del error o sesgo de no respuesta porque no todas las unidades de la muestra, en este caso personas, participan exitosamente de la encuesta (no respuesta a la unidad), esto se debe por ejemplo a que resultó imposible contactar a la persona a encuestar (no contacto), o no quiso participar de la misma (rechazo) o bien tenía una inhabilidad para hacerlo. La no respuesta a la unidad genera problemas de reducción de la muestra, provocando sesgos en la estimación de las estadísticas de la encuesta. Sin embargo, si la no respuesta es aleatoria, es posible realizar estimaciones validas ya que los estimadores serán insesgados, aunque disminuirá la precisión de esta (Groves, *et al* 2004).

Para ENDIDE se observan 21.033 personas que no respondieron pese a que resultaron elegibles. Así, de las 56.569 personas elegibles seleccionadas en la muestra, se realizaron 35.536 encuestas de manera exitosa, obteniendo una tasa de respuesta del 62,8%.

Para ajustar los factores de expansión por "No Respuesta", se debe estimar la probabilidad de contestar de cada persona. Como ENDIDE utiliza como marco muestral el resultado del empadronamiento del precontacto Casen en Pandemia 2020, se considerarán las variables recogidas desde ahí, para estimar un modelo probabilístico que estime la probabilidad de respuesta.

Para estimar la probabilidad de contestar se utiliza un modelo probit, el cual es un modelo binario que sirve para analizar si el modelo propuesto es capaz de clasificar de manera correcta la capacidad de respuesta del individuo, en este caso se consideran como variables explicativas la edad y sexo del jefe de hogar, así como la comuna y la zona (urbano o rural) de la vivienda.

Con el objetivo de ajustar la no respuesta se utiliza un modelo "Propensity score" que calcula la probabilidad de responder de cada persona de la muestra, para ello se ordenan las unidades de acuerdo con sus probabilidades estimadas de menor a mayor, creando estratos llamados veintiles, que consisten en 20 grupos de igual cantidad de personas ordenados según lo establecido anteriormente.

Cuando ya se tienen las 20 celdas de ajuste, se procede a estimar el factor de personas ajustado a no respuesta, el cual está dado por la siguiente ecuación:

$$(15) \qquad w_{r,k}^{NR} = \widehat{R_v} * w_{r,k}$$

Siendo:

$$(16) \qquad \widehat{R_v} = \frac{\sum_{k\epsilon\phi_r,eleg,v} w_{r,k}}{\sum_{k\epsilon\phi_r,log,v} w_{r,k}}$$

Donde, el numerador representa la suma del conjunto de personas seleccionadas en el estrato r (región) clasificadas como elegibles dentro del veintil ν y el denominador como la suma del conjunto de personas logradas en el estrato r (región) clasificadas como elegibles y que responden la encuesta, en el veintil ν. Y siendo $w_{r,k}$ un factor teórico correspondiente al ponderador de elegibilidad. Si la persona la persona k es elegible y no responde toma el valor perdido o missing.

Los veintiles quedan clasificados de acuerdo con lo presentado en la Tabla 7.

Tabla 7: Número de personas según veintil

Veintil	Personas que responden
1	1.173
2	1.439
3	1.548
4	1.788
5	1.678
6	1.889
7	1.812
8	1.776
9	1.840
10	1.893
11	1.910
12	2.025
13	1.992
14	1.886
15	1.862
16	1.989
17	1.862
18	1.618
19	1.603
20	1.953

Fuente: elaboración en base a Proyecto ENDIDE 2022

4.3.3. Suavizamiento del factor de expansión

Para poder controlar la variabilidad final de los estimadores se analizaron los factores de expansión resultantes de los ajustes por no elegibilidad y no respuesta, identificando factores de expansión típicos y atípicos.

El objetivo de esta etapa es reducir el coeficiente de variación de los factores de expansión (corregir valores anómalos o extremos).

Siguiendo lo utilizado por las últimas encuestas del INE y del MDSF se aplicará un método de suavización mixta que utiliza el método de contracción a la media (CM) y el método R-K.

4.3.3.1. Método de contracción a la media

El método de contracción de media, tal como su nombre lo indica, consiste en contraer los factores de expansión acercándolos en distancia a la media. De esta forma, la distribución de los factores se vuelve más homogénea (INE, 2020).

Sea w_i el factor de expansión i-ésimo corregido por no respuesta y w^* el promedio de los factores al nivel al cual se requiera aplicar el suavizamiento. El factor suavizado o contraído a la media w_i

$$(17) \quad S = \beta \cdot wi + (1 - \beta) \cdot w^*; \; 0 \leq \beta \leq 1$$

En este caso β es un parámetro real que permite controlar la intensidad de la contracción. Si se desea contraer muy poco la distribución original de los factores, entonces se le debe dar más peso a los valores originales (w_i) asignándole a β valores cercanos a 1. Por otra parte, si se desea maximizar la homogeneidad de los factores, es decir, que todos los factores sean lo más parecido a la media, se debe ponderar más el valor de la media (w^*), asignándole a β valores cercanos a 0.

4.3.3.2. Método R-K

El método R-K consiste en disminuir o aumentar los factores en una proporción (r o k), dicha proporción se basa en si los valores se consideran atípicos o no atípicos. La proporción r es para la disminución de los factores atípicos mientras que la proporción k es para el aumento de los no atípicos. De esta forma, se busca acercar los valores atípicos y no atípicos, mediante un tratamiento diferenciado (INE, 2020).
Para este procedimiento se requieren dos restricciones, la primera es que la suma de los factores de expansión siga siendo la misma, y la segunda es que para mantener el orden en la distribución de los factores el mayor factor de los no atípicos suavizados debe ser menor al menor factor de los atípicos suavizados.

El primer paso es ver la cantidad de casos atípicos presentes en cada región, resumidos en la Tabla 8:

Tabla 8: Número de casos atípicos por región

Región	Casos atípicos
Arica y Parinacota	0
Tarapacá	0
Antofagasta	3
Atacama	0
Coquimbo	48
Valparaíso	96
Metropolitana	2.105
O'Higgins	40
Maule	54
Biobío	58
Ñuble	27
La Araucanía	49
Los Ríos	3
Los Lagos	58
Aysén	0
Magallanes	0
**Total**	**2.541**

Fuente: elaboración en base a Proyecto ENDIDE 2022

En la Tabla 8 se observa que la región con mayor cantidad de casos anómalos corresponde a la región Metropolitana con 2.105 casos. Al diferenciar por zona, se tiene 2.386 en la zona urbano y 155 en la zona rural.

Para ENDIDE se aplicó un método mixto, para el CM se utilizó un valor de β=0,8 es decir, se les dio más importancia a los valores originales. Para el caso de R-K se utilizó un valor $\lambda = 0{,}99$.

4.3.3.3. Calibración del factor de expansión

La calibración rehace el cálculo de los factores de expansión, pero añadiendo nueva información auxiliar que permite ajustar las estimaciones a los totales poblacionales conocidos. Así se obtienen factores de expansión más cercanos a los factores de expansión originales, asimismo se ajustan a los totales poblacionales previamente definidos, los que se conocen como ecuaciones de calibración o marginales (Särndal, 2007; INE, 2020b).

Para llevar a cabo la calibración se utiliza el método de Raking, el cual busca determinar el conjunto de pesos $\{w_i\}$ tal que minimice la ecuación $L(\mathrm{w}, \mathrm{d}) = \sum \frac{(w_i - d_i)^2}{d_i}$ donde w son los pesos "calibrados" y d los pesos originales.

Para la calibración de ENDIDE se utilizó la información de las proyecciones de población al 30 de junio del 2022 en base al Censo 2017 publicadas por el Instituto Nacional de Estadísticas. En particular, se utilizaron los siguientes marginales de calibración:

- Población total por región
- Población total por sexo
- Población total por zona
- Población total por tramo de edad: [2-17], [18-29], [30-44], [45-59], [60-69], [70-79] y [80 y más].
- Población total por región y zona
- Población total por región y sexo
- Población total por tramo de edad y sexo

4.3.3.4. Redondeo de los factores de expansión

El redondeo de los factores de expansión se lleva a cabo para no tener problemas con las estimaciones realizadas con la base de datos al momento de obtener resultados que involucren decimales.

De modo general, el problema de redondeo puede ser afrontado con una perspectiva probabilística, en el sentido que los excesos de la parte decimal que se eliminan en un proceso de redondeo determinístico se distribuyan entre los elementos en base a una distribución de probabilidades que refleje la pérdida relativa en el proceso de redondeo determinístico. De hecho, si en primera instancia se utiliza la parte entera (el entero máximo que sea menor o igual) del ponderador, como su valor redondeado; entonces bastará con añadir aleatoriamente una unidad a algunos ponderadores para asegurar que la suma de los ponderadores redondeados sea aproximadamente idéntica a los totales originales. De esta forma, se puede recuperar la propiedad del insesgamiento que se pierde con el procedimiento de redondeo determinístico.

Como resultado de la construcción de los factores de expansión, incluyendo los procedimientos de calibración y redondeo, los factores de expansión se calibraron a un total de 19.348.925 personas de 2 años y más, tal como se muestra en la Tabla 9.

Tabla 9: Distribución de la población de 2 años y más aplicando factor de expansión ENDIDE según nivel de estimación

Región/Zona	Tamaño expandido a población de 2 años y más	Tamaño Muestra
Urbano	17.141.403	4.968
Rural	2.207.522	30.568
Arica y Parinacota	251.353	1.146
Tarapacá	380.430	1.105
Antofagasta	689.775	1.723
Atacama	309.778	1.259
Coquimbo	838.798	1.802
Valparaíso	1.952.466	3.520
Metropolitana	8.100.276	7.968
O'Higgins	986.921	2.075
Maule	1.126.696	2.376
Biobío	1.639.644	3.575
Ñuble	506.525	1.266
La Araucanía	1.000.564	2.277
Los Ríos	400.759	1.184
Los Lagos	882.229	1.850
Aysén	105.459	1.120
Magallanes	177.252	1.290
**Total**	**19.348.925**	**35.536**

Fuente: elaboración en base a Proyecto ENDIDE 2022

4.4. Errores efectivos

A partir de los resultados del levantamiento de ENDIDE, se calcularon los estadísticos asociados a los parámetros de interés definidos para la encuesta. En particular, se obtienen los errores efectivos de los siguientes resultados:

- Discapacidad a nivel Nacional, Zona y Región, Personas Adultas (18 años y más), ver Tabla 10.
- Discapacidad a nivel Nacional, Zona y Región, Personas Mayores (60 años y más), ver Tabla 11.
- Discapacidad a nivel Nacional, Niños, Niñas y Adolescentes (2 a 17 años), ver Tabla 12.
- Dependencia a nivel Nacional, Zona y Región, Personas Adultas (18 años y más), ver Tabla 13.
- Dependencia a nivel Nacional, Zona y Región, Personas Mayores (60 años y más), ver Tabla 14.

Tabla 10: Prevalencia de discapacidad en personas adultas (18 años y más), por zona y región.

Zona/Región	Estimación (%)	Error Absoluto	Error Relativo
Urbano	17,6	0,6	3,4
Rural	17,8	1,5	8,4
Arica y Parinacota	13,4	2,2	16,7
Tarapacá	10,8	2,3	21,4
Antofagasta	7,8	1,4	18,0
Atacama	16,7	2,5	14,9
Coquimbo	10,6	1,6	15,5
Valparaíso	16,5	1,6	9,8
Metropolitana	19,1	1,1	5,7
O'Higgins	14,4	1,9	13,1
Maule	14,2	1,8	12,7
Ñuble	22,9	3,1	13,7
Biobío	21,9	1,6	7,3
La Araucanía	22,0	2,1	9,4
Los Ríos	22,0	3,0	13,5
Los Lagos	17,0	1,9	11,4
Aysén	19,6	2,9	15,1
Magallanes	9,3	2,1	22,1
**Total**	**17,6**	**0,6**	**3,1**

Fuente: Elaboración propia con base de datos ENDIDE 2022

Tabla 11: Prevalencia de discapacidad en personas mayores (60 años y más), por zona y región.

Zona/Región	Estimación (%)	Error Absoluto	Error Relativo
Urbano	32,2	1,3	4,0
Rural	35,8	2,9	8,2
Arica y Parinacota	28,1	5,0	17,8
Tarapacá	19,0	6,1	32,2
Antofagasta	22,0	4,4	19,8
Atacama	39,1	5,8	14,9
Coquimbo	28,4	4,3	15,1
Valparaíso	31,8	3,4	10,6
Metropolitana	31,5	2,1	6,8
O'Higgins	28,8	4,5	15,5
Maule	33,7	4,3	12,8
Ñuble	42,5	5,6	13,1
Biobío	37,3	3,4	9,1
La Araucanía	43,7	4,5	10,3
Los Ríos	41,8	6,2	14,8
Los Lagos	37,2	5,0	13,6
Aysén	27,8	4,8	17,1
Magallanes	18,4	4,4	24,2

Zona/Región	Estimación (%)	Error Absoluto	Error Relativo
**Total**	**32,6**	**1,2**	**3,6**

Fuente: Elaboración propia con base de datos ENDIDE 2022

Tabla 12: Prevalencia de discapacidad en niños, niñas y adolescente (2 a 17 años).

Zona/Región	Estimación (%)	Error Absoluto	Error Relativo
**Total**	**14,7**	**1,2**	**7,8**

Fuente: Elaboración propia con base de datos ENDIDE 2022

Tabla 13: Prevalencia de dependencia en personas adultas (18 años y más), por zona y región.

Zona/Región	Estimación (%)	Error Absoluto	Error Relativo
Urbano	9,7	0,4	4,5
Rural	9,9	1,1	11,1
Arica y Parinacota	7,7	1,7	22,4
Tarapacá	6,2	1,7	26,9
Antofagasta	4,5	1,0	23,1
Atacama	9,7	1,9	19,6
Coquimbo	5,9	1,2	21,1
Valparaíso	9,0	1,2	13,2
Metropolitana	11,0	0,8	7,3
O'Higgins	9,4	1,5	15,4
Maule	7,3	1,2	16,2
Ñuble	13,1	2,5	18,8
Biobío	9,9	1,1	11,3
La Araucanía	11,5	1,5	12,7
Los Ríos	11,6	2,3	19,5
Los Lagos	9,8	1,5	15,4
Aysén	10,3	2,3	22,7
Magallanes	4,6	1,2	26,8
**Total**	**9,8**	**0,4**	**4,2**

Fuente: Elaboración propia con base de datos ENDIDE 2022

Tabla 14: Prevalencia de dependencia en personas mayores (60 años y más), por zona y región.

Zona/Región	Estimación (%)	Error Absoluto	Error Relativo
Urbano	22,1	1,1	5,1
Rural	23,1	2,6	11,1
Arica y Parinacota	18,3	4,4	24,3
Tarapacá	15,3	5,6	36,7
Antofagasta	14,3	3,6	25,4

Zona/Región	Estimación (%)	Error Absoluto	Error Relativo
Atacama	27,5	5,4	19,7
Coquimbo	17,7	3,8	21,6
Valparaíso	20,3	2,9	14,1
Metropolitana	22,1	1,9	8,4
O'Higgins	22,3	4,2	18,7
Maule	20,1	3,5	17,6
Ñuble	26,0	4,9	18,7
Biobío	23,9	3,0	12,7
La Araucanía	30,9	4,4	14,3
Los Ríos	28,5	5,6	19,6
Los Lagos	26,5	4,6	17,2
Aysén	19,5	4,6	23,6
Magallanes	11,0	3,1	28,1
**Total**	**22,2**	**1,0**	**4,7**

Fuente: Elaboración propia con base de datos ENDIDE 2022

II. METODOLOGÍA DE MEDICIÓN DE LA DISCAPACIDAD

Esta sección tiene por objeto presentar la metodología de medición de discapacidad de ENDIDE para la población de personas adultas, abarcando los siguientes apartados: conceptualización de la discapacidad, medición de la discapacidad, modelo de Rasch, uso del modelo de Rasch para la medición de la discapacidad y la determinación de los niveles de dificultad y la prevalencia de la discapacidad.

1. Conceptualización de la Discapacidad

El marco conceptual para la medición de discapacidad utilizado en la Encuesta Nacional de Discapacidad y Dependencia 2022 (ENDIDE), se basa en la Clasificación Internacional del Funcionamiento, de la Discapacidad y de la Salud (CIF), publicada por la Organización Mundial de la Salud (OMS) el año 2001. La CIF tiene como objetivo brindar un marco conceptual comparable entre países y diferentes disciplinas sanitarias, que permite describir la salud y los estados "relacionados con la salud" (OMS, 2001). Esta clasificación define los componentes y los describe en términos de *dominios de salud* y *dominios relacionados con la salud*, entendiendo como dominio "un conjunto relevante y práctico de funciones fisiológicas, estructuras anatómicas, acciones, tareas o áreas de la vida relacionadas entre sí" (OMS, 2001, p. 231). De esta manera, los componentes de la salud refieren, por ejemplo, a visión, audición, movilidad, aprendizaje y memoria, mientras que los componentes relacionados con la salud refieren a aspectos tales como el transporte, la educación y las interacciones sociales. Con todo, si bien esta clasificación considera un amplio concepto de la salud, no cubre circunstancias que no estén relacionadas de manera directa con ella, tales como los factores socioeconómicos (OMS, 2001).

La CIF ha sido aceptada como una de las clasificaciones sociales de las Naciones Unidas e incorpora las Normas Establecidas para la Igualdad de Oportunidades para las Personas con Discapacidad[10]. Por estas razones, se ha instaurado como un marco conceptual apropiado para implementar los mandatos internacionales sobre los derechos humanos, así como las legislaciones nacionales (OMS, 2001). La información que entrega la CIF está organizada en dos partes, la primera sobre Funcionamiento y Discapacidad, con sus componentes relativos al cuerpo, las actividades y la participación; y la segunda agrupa a los factores contextuales, diferenciando entre aquellos ambientales y personales (OMS, 2001).

[10] Más información sobre la Ley 20.422 se encuentra disponible en: https://www.bcn.cl/leychile/navegar?idLey=20422

Figura 1: Modelo conceptual de la CIF

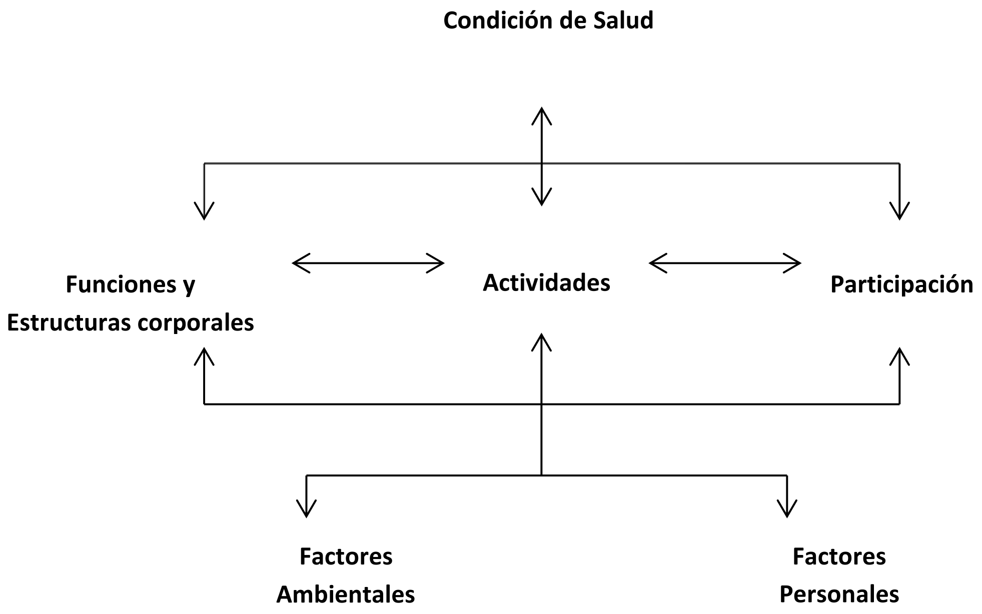

Fuente: Organización Mundial de la Salud, Clasificación Internacional del Funcionamiento, de la Discapacidad y de la Salud, 2001.

El diagrama presentado en la Figura 1 muestra los componentes de la CIF, donde el Funcionamiento y Discapacidad de una persona en un dominio específico, se entienden como una interacción dinámica entre la Condición de Salud y los Factores Contextuales (ambientales y personales[11]). El dinamismo implica que un elemento tiene la potencialidad de modificar uno o más de los otros elementos (interacciones bidireccionales). Además, estas interacciones son específicas y no siempre se dan en una relación recíproca predecible. Como el objetivo de la CIF es describir la experiencia de salud de manera global, todos sus componentes son importantes (OMS, 2001). En este sentido, los Factores Contextuales (personales y ambientales) tienen un rol en el proceso dinámico y complejo de la discapacidad. Estos factores interactúan con la condición de salud de la persona determinando el nivel y extensión de su funcionamiento.

El año 2011, la OMS y el Banco Mundial (BM) dieron inicio al proyecto de la Encuesta Modelo de Discapacidad (MDS, por sus siglas en inglés). Esta encuesta se basa en la CIF y representa un avance en

[11] Factores Ambientales: son aquellos factores que «ejercen un efecto en todos los componentes del funcionamiento y de la discapacidad» (CIF, 2001, p. 9). Están organizados en una lista que va desde el entorno más inmediato del individuo hasta el más general. Factores Personales: son un componente de los factores contextuales, pero que no se encuentran clasificados en la CIF dada su gran variabilidad social y cultural.

la forma de medir la discapacidad: en lugar de enfocarse únicamente en el estado de salud de una persona, la MDS explora la discapacidad como la experiencia de vivir con una condición de salud o discapacidad en un entorno que puede facilitar o dificultar la participación plena en la sociedad.

De acuerdo con el marco conceptual de la CIF, la MDS adopta los siguientes enfoques:

- La discapacidad no es un atributo interno de la persona, sino una experiencia.
- La discapacidad no tiene una causa específica.
- La discapacidad se presenta en un continuo, con distintos grados que van desde la ausencia de discapacidad hasta una discapacidad extrema.
- La discapacidad es universal, lo que significa que todas las personas se encuentran en algún punto del continuo de discapacidad.

La MDS es una encuesta dirigida a población general, que considera una muestra representativa de la población, y que cuenta con tres objetivos principales:

- Obtener tasas de prevalencia de discapacidad comparables y estandarizadas en todos los países.
- Proporcionar datos necesarios para diseñar intervenciones, programas y políticas adecuadas para personas con diferentes niveles de discapacidad.
- Supervisar la implementación de los Objetivos de Desarrollo Sostenible (ODS) y la Convención de las Naciones Unidas sobre los Derechos de las Personas con Discapacidad (CRPD, por sus siglas en inglés), permitiendo comparaciones directas entre personas con distintos niveles de discapacidad y aquellas sin discapacidad.

Considerando el modelo de medición de discapacidad planteado por la MDS, el año 2015 Senadis con colaboración del MDSF realizaron el Segundo Estudio Nacional de Discapacidad (ENDISC II) siguiendo los lineamientos planteados previamente. A partir de esta experiencia, la necesidad de actualizar la medición y recientes avances en la medición de discapacidad (nueva versión corta de la MDS disponible a partir de 2018) se llevó a cabo una nueva medición de discapacidad en la Encuesta Nacional de Discapacidad y Dependencia 2022.

2. Medición de la Discapacidad en ENDIDE

La metodología de medición de discapacidad a partir de los datos de ENDIDE, sigue los lineamientos de la OMS aplicados en Endisc 2015, entendiendo a la discapacidad como el resultado de la interacción entre una persona con una condición de salud y diversos factores ambientales y personales, en lugar de centrarse sólo en la salud o capacidad de la persona. Bajo este enfoque la discapacidad se comprende como un continuo que va desde “bajos o nulos niveles de discapacidad” a “altos niveles de discapacidad” y en el cual toda la población puede posicionarse.

Para medir a la discapacidad como un continuo, se elaboran escalas métricas que permiten ubicar a las personas de acuerdo con los niveles de dificultad reportados en los diferentes de dominios del funcionamiento considerados en la encuesta (dominios de la CIF).

ENDIDE mide el funcionamiento desde la óptica de la Capacidad y el Desempeño, en línea con el diseño de la MDS. A través de la capacidad se busca medir el máximo nivel posible de funcionamiento que puede alcanzar una persona en un momento dado, considerando solo su estado de salud. La capacidad se mide en un contexto/entorno uniforme o normalizado, y por ello, refleja la habilidad de un individuo ajustada en función al ambiente (OMS, 2001).

En contraste, el concepto de desempeño busca medir lo que los individuos hacen en su entorno real, considerando los efectos de los factores ambientales en su funcionamiento. Esto es, el uso de ayudas técnicas, medicamentos, asistencia personal, así como las características de su entorno (facilitadores y barreras).

La ENDIDE incorpora en su cuestionario a personas adultas los módulos "Capacidad y condición de salud" y "Desempeño" basados en los constructos mencionados. A partir de los resultados recogidos en ellos, se construyen escalas con propiedades métricas para ambos constructos.

Una vez estimadas las métricas de capacidad y desempeño, la información es combinada para identificar a las personas clasificadas como personas con discapacidad, así como para graduar la severidad de la situación de discapacidad.

La Tabla 15 presenta los ítems que componen la métrica de capacidad y la Tabla 16 aquellos que componen la métrica de desempeño.

Tabla 15. Ítems de la escala de Capacidad, ENDIDE 2022

Módulo C: Capacidad y Condición de Salud	
Las siguientes preguntas refieren a dificultades que pudiera tener para realizar ciertas actividades debido solamente a su salud y sin considerar dispositivos de ayuda o personas que le ayudan.<br><br>Ahora, quiero que me responda las siguientes preguntas utilizando una escala de 1 a 5, en la que 1 significa ninguna dificultad y 5 significa dificultad extrema o que no puede hacer la actividad.<br><br>**En los últimos 30 días y sin tener en cuenta ningún tipo de ayuda. Debido a su salud, ¿qué grado de dificultad ha tenido para…**	
c2	Ver, sin usar anteojos ópticos o lentes?
c3	Oír, sin usar audífono o dispositivo de ayuda para oír?
c4	Caminar o subir peldaños?
c5	Recordar cosas o concentrarse?

Módulo C: Capacidad y Condición de Salud	
c6	Asearse o vestirse?
c11	Dormir
c12	Hacer las tareas de la casa como barrer, cocinar, hacer arreglos o sacar la basura?
c15	Participar en fiestas, eventos religiosos, reuniones vecinales u otras actividades de la comunidad?
c16	Llevarse bien con la gente cercana a usted, incluyendo su familia, amigos y amigas?
Debido a su salud, **¿qué grado de dificultad le ha generado en su vida…**	
c22	Sentirse triste, deprimido(a), preocupado(a) o ansioso(a)?
c23	Sentir algún malestar o dolor físico, por ejemplo, dolor de espalda, dolor de estómago o dolor de cabeza?

Fuente: Cuestionario Personas Adultas ENDIDE 2022

Tabla 16. Ítems de la escala de Desempeño, ENDIDE 2022

Módulo D: Desempeño	
En esta sección quiero que reflexione acerca de los tipos de problemas que experimenta en su vida. Estos problemas pueden surgir debido a su condición de salud, al ambiente en el que vive, o a las actitudes o conductas de las personas que lo rodean. Por favor al responder tenga en cuenta las personas que le ayudan, los medicamentos que toma y todos los dispositivos de apoyo que utiliza, tales como lentes, audífonos, bastón, silla de ruedas, prótesis, elementos tecnológicos, entre otros.<br><br>Para cada pregunta, por favor dígame qué tan problemático es para usted usando una escala de 1 a 5, en la que 1 significa nada problemático y 5 extremadamente problemático o no puede hacerlo.<br><br>**En los** últimos 30 días**, ¿qué tan problemático ha sido para usted…**	
d1	Caminar diez cuadras o un kilómetro?
d2	Llegar a los lugares donde ha querido ir?
d3	Asearse o vestirse?
d4	Usar el baño (W.C.)?
d5	Cuidar de su salud, como por ejemplo, hacer ejercicio, alimentarse bien o tomar sus medicamentos?
d6	Sentirse cansado(a) y no tener suficiente energía?
d7	Enfrentar todas las tareas que tiene que hacer?
d8	Recordar las cosas importantes que tiene que hacer en su día a día?
d9	Hacer las tareas de la casa como barrer, cocinar, hacer arreglos o sacar la basura?
d10	Participar en fiestas, eventos religiosos, reuniones vecinales u otras actividades de la comunidad?
d11	Utilizar los servicios de transporte público?
d12	Realizar las tareas que le solicitan en su trabajo o establecimiento educacional?

Fuente: Cuestionario Personas Adultas ENDIDE 2022

Siguiendo la metodología propuesta por la OMS, para construir las métricas de capacidad y desempeño y medir la discapacidad a partir de los datos recogidos en la ENDIDE, se utiliza la técnica estadística *Modelo de Rasch*, en específico, el *Modelo de Crédito Parcial de Rasch*. Esta técnica permite la

construcción de una escala continua a partir de un conjunto de ítems de respuesta ordinal, obteniendo medidas precisas y confiables, lo que resulta fundamental para la planificación de intervenciones, el seguimiento de progresos y la toma de decisiones informadas.

El modelo de Rasch ha sido ampliamente utilizado en el campo de la discapacidad para evaluar y medir distintas habilidades, capacidades y niveles de funcionamiento en personas con y sin discapacidad. Esta técnica estadística ha demostrado su utilidad para evaluar la calidad de instrumentos de medición utilizados en el ámbito de la discapacidad, así como para comparar y clasificar a los individuos en una misma escala de habilidad.

3. El Modelo de Crédito Parcial de Rasch

El Modelo de Crédito Parcial de Rasch es un modelo unidimensional de la Teoría de Respuesta al ítem (TRI) adecuado para ítems politómicos ordinales. Las principales contribuciones de la TRI refieren a la posibilidad de medir el nivel de habilidad que tiene una persona con relación al constructo o competencia que se desea medir (Nakano y Primi, 2014). La TRI, además, permite analizar de manera detallada la estructura interna de los ítems que se utilizan para evaluar un área de competencia, indicando cuáles de ellos exigen una menor o mayor habilidad por parte del individuo. Particularmente, el análisis de Rasch se ha convertido en uno de los principales recursos técnicos para determinar las propiedades métricas de los ítems de una prueba (Backhoff et al, 2022).

El modelo de Crédito Parcial de Rasch asume que existe un constructo latente unidimensional que puede ser medido a partir de la respuesta a ítems politómicos, es decir, que existe una escala subyacente en la que personas e ítems pueden ser ubicados: para las personas la ubicación se denomina “habilidad de la persona” y para los ítems “dificultad del ítem” (Masters, 1982).

El enfoque del modelo para la medición es un enfoque probabilístico, basado en la frecuencia esperada de respuesta de las personas a los ítems. En este modelo, la probabilidad de que un individuo dé cierta respuesta a un ítem determinado depende exclusivamente de su ubicación en el continuo del rasgo latente (habilidad de la persona) y de la dificultad del ítem. Por lo tanto, las personas con niveles más altos en el constructo medido (por ejemplo, dificultad severa en desempeño), son más propensas a responder los ítems que reflejan el aumento de impedimentos, es decir, que cuentan con un mayor nivel de dificultad.

4. Uso del Modelo de Crédito Parcial de Rasch para la medición de la Discapacidad

El modelo de Rasch y en particular el Modelo de Crédito Parcial de Rasch, fue escogido por la OMS para la medición del funcionamiento humano y la discapacidad debido a que permite tomar datos ordinales

y transformarlos en una escala con propiedades métricas. Adicionalmente, este modelo cuenta con otras propiedades importantes que lo convierten en un método particularmente útil para la construcción de escalas métricas acordes con el marco conceptual entregado por la CIF:

- No establece suposiciones sobre la distribución de las capacidades (medida) de personas.
- Las dificultades de los ítems pueden compararse independientemente de las capacidades de las personas.
- Las capacidades de dos personas se pueden comparar independientemente de las dificultades de los ítems.
- La diferencia entre las capacidades de las personas y las dificultades de los ítems es una estadística confiable.

4.1 Estimación del Modelo de Crédito Parcial de Rasch

Para la estimación de las métricas a partir del Modelo de Crédito Parcial de Rasch, el procedimiento básico consiste en evaluar el ajuste de los datos al modelo y el cumplimiento de sus supuestos.

A diferencia de otros enfoques en los cuales se adapta el modelo a los datos, el modelo de Rasch es considerado el ideal y se busca ajustar los datos al modelo. Si los datos se ajustan de manera razonable al modelo, cumpliendo sus supuestos y obteniendo buenos indicadores de ajuste, es posible confiar en que se ha obtenido una escala de intervalo válida y confiable y que cumple con las propiedades del modelo de Rasch.

La estimación del modelo de Rasch es un proceso iterativo que requiere realizar múltiples pasos para obtener resultados. El proceso consiste en:

1. Ejecutar el modelo en los datos
2. Probar los supuestos
3. Ajustar los datos

Este procedimiento se repite hasta lograr un modelo óptimo, que cumple con los supuestos del Modelo de Rasch y presenta indicadores de ajuste adecuados. La cantidad de iteraciones dependerá exclusivamente de la calidad de los datos.

En la siguiente sección se presentan los supuestos del modelo y las pruebas realizadas a los datos de ENDIDE.

4.2 Evaluación de Supuestos y Ajuste del Modelo de Crédito Parcial de Rasch

a. Unidimensionalidad

El supuesto de unidimensionalidad establece que solo una dimensión explica las respuestas de los individuos a los ítems analizados. Dada la dificultad de que solo un factor explique completamente las respuestas de los individuos, se espera identificar al menos unidimensionalidad esencial o dominante.

De esta manera, en ENDIDE se buscó probar que todos los ítems analizados midieran el mismo constructo (capacidad o desempeño dependiendo de los ítems considerados), aportando cada uno por separado a explicar una proporción de la variación en la información de la escala métrica principal.

Para evaluar la unidimensionalidad de los datos (Reeve et al, 2007) se llevó a cabo un análisis exploratorio mediante un análisis bi-factor. Este análisis asume la presencia de un único factor general y múltiples factores de grupo independientes. Las dos condiciones que se deben cumplir para asumir una unidimensionalidad latente subyacente son:

1. Todos los ítems deben cargar alto en el factor general. Esto es, las cargas factoriales del primer factor deben estar próximas al factor general al solicitar un solo factor en el análisis bi-factor. Las cargas de los factores de grupo no deben ser más altas que las del factor general para todos los ítems que componen la escala métrica.
2. Las cargas factoriales de los ítems en el factor general deben exceder las de los factores de grupo.

A partir del análisis bi-factor, se probó el cumplimiento de la unidimensionalidad con los datos de ENDIDE. Tanto para la métrica de capacidad como de desempeño, los datos de los modelos finales satisfacen el supuesto evaluado.

Durante el proceso de ajuste de los datos, en todos los modelos creados se obtuvo un valor cercano al factor general. Sin embargo, entre algunos ítems (ítems sobre movilidad y autocuidado) se observó una alta correlación residual luego de aplicar el modelo de Rasch, afectando con ello el cumplimiento del supuesto de independencia local. Siguiendo las recomendaciones de la OMS, lo anterior fue corregido a través de la creación de un testlet con ambas variables. De esta manera, al corregir el incumplimiento del supuesto de independencia local, también se corrige la unidimensionalidad, por lo cual se recomienda evaluar nuevamente el cumplimiento de este supuesto tras la creación de testlet.

b. Independencia local

El supuesto de independencia local refiere a que, dado el nivel de un individuo en el rasgo o constructo medido, la probabilidad de respuesta a un ítem determinado es independiente localmente de las respuestas a los demás ítems de la escala. De esta manera, se busca que las respuestas a un ítem no se relacionen fuertemente con las respuestas de otro ítem (Cortada de Kohan, Nuria, 2004), considerando que la correlación se presenta cuando los ítems están vinculados por atributos, estructuras o contenidos comunes.

La independencia local puede ser examinada inicialmente revisando las correlaciones residuales del análisis factorial con rotación bifactorial al pedir un sólo factor, resultados que derivan del punto anterior. Sin embargo, para asegurar el cumplimiento de la independencia local se deben examinar las correlaciones residuales de los parámetros de habilidad de las personas obtenidos con el modelo de Crédito Parcial de Rasch, buscando que las correlaciones no superen el valor 0,2 para afirmar que no existe evidencia de correlación significativa entre los ítems. Por el contrario, se considerará que dos ítems muestran dependencia local cuando las correlaciones residuales obtenidas posterior a la aplicación del Modelo de Rasch son mayores a 0,2. En caso de hallar dependencia local entre ítems, a fin de corregir dicha situación, se recomienda agrupar los ítems mediante una estrategia de *testlet*, el cual corresponde a una suma simple de las respuestas dadas a los ítems que muestran dependencia local.

Como fue señalado en el punto anterior, en el proceso de ajuste de los datos de las escalas de capacidad y desempeño se crearon *testlet* entre ítems que presentaron correlaciones residuales superiores al umbral de 0,2. Esta estrategia permitió el cumplimiento de los supuestos de unidimensionalidad e independencia local en ambas métricas.

c. Monotonicidad

Este supuesto implica identificar de manera distributiva o visual si existe un crecimiento monótono de la escala métrica en razón de las respuestas de las personas a los ítems. De esta manera se espera que cada vez que una persona puntúa algún nivel de dificultad a cualquier ítem, éste afecte la escala métrica aumentando o disminuyendo dependiendo de su respuesta. Por lo tanto, si una persona o grupo de personas declara mayor dificultad en los ítems, el valor en la escala métrica debería aumentar y no disminuir (en caso contrario debería disminuir).

La monotonicidad, en el caso de ENDIDE, significa que la probabilidad de que una persona responda que tiene dificultad en un ítem, aumenta a medida que se incrementa el nivel de severidad en la escala de capacidad o desempeño. Este supuesto es probado para cada ítem mediante una comparación (que puede ser mediante un cruce de variables o un gráfico de mosaico) entre las puntuaciones en dicho

ítem y la media de las puntuaciones del resto de los ítems, la cual es calculada para cada persona como el puntaje promedio de todos los ítems restantes (Reeve, B.B. et al, 2007). Si hay una tendencia constante de que las personas con puntuaciones medias (restantes) más altas son más propensas a tener más problemas en el ítem analizado, entonces se puede asumir la monotonicidad en dicho ítem. En caso de que se observen umbrales desordenados al aplicar el Modelo de Crédito Parcial de Rasch, las categorías de respuesta a los ítems deben ser colapsadas hasta que todos los umbrales estén en el orden correcto.

La monotonicidad puede ser observada en los gráficos de distribución de mosaicos. Asimismo, se pueden considerar subdivisiones de intervalos para poder realizar un análisis más detallado. Estos gráficos corresponden a un cruce de variables que considera, por una parte, el ítem con todas sus categorías, y por otro, la media de la suma de los puntajes de todos los ítems restantes. La monotonicidad se puede asumir si las personas con puntuaciones medias más altas son consistentemente más propensas a tener más problemas en el ítem seleccionado (gráficamente, un comportamiento de escalera ascendente de izquierda a derecha).

Además, la monotonicidad puede ser evaluada en el gráfico de Ítem-Persona (obtenido dentro del modelo de Rasch) al observar el orden de los umbrales de las categorías de respuesta.

d. Ordenamiento estocástico

El ordenamiento estocástico refiere al orden de los umbrales de respuesta, es decir a los límites entre las opciones de respuesta. En este punto se busca identificar si los umbrales de respuesta tienen un orden correcto según las opciones de respuesta, es decir, identificar si los límites de respuesta se encuentran ordenados.

La comprobación del orden estocástico se lleva a cabo para cada ítem, esperando que la probabilidad de que una persona (con cierto valor en la escala métrica) cruce el primer umbral, sea mayor a la probabilidad de que cruce el segundo, y así sucesivamente dependiendo de la cantidad de umbrales. En el caso de ENDIDE, la probabilidad de que una persona responda dificultad leve a un ítem debe ser mayor a la probabilidad de que responda dificultad moderada, y la probabilidad de que responda una dificultad moderada debe ser mayor a que responda dificultad severa, etc.

El ordenamiento estocástico se puede evaluar en la gráfica *Person Item Map*, herramienta que ordena visualmente los componentes del análisis de Rasch, ubicando los umbrales en el continuo de la métrica. En el caso de identificar problemas con el orden de los umbrales de respuesta, se recomienda recodificar las opciones de respuesta, lo que disminuye el número de umbrales.

En el caso de ENDIDE, para las métricas de capacidad y desempeño fue necesario recodificar categorías de respuesta hasta lograr el orden estocástico de cada uno de los ítems. Como ejemplo, la figura 2 presenta el resultado del *Person Item Map* en la métrica de desempeño.

Figura 2: Mapa ítem-persona de la métrica de desempeño, ENDIDE 2022

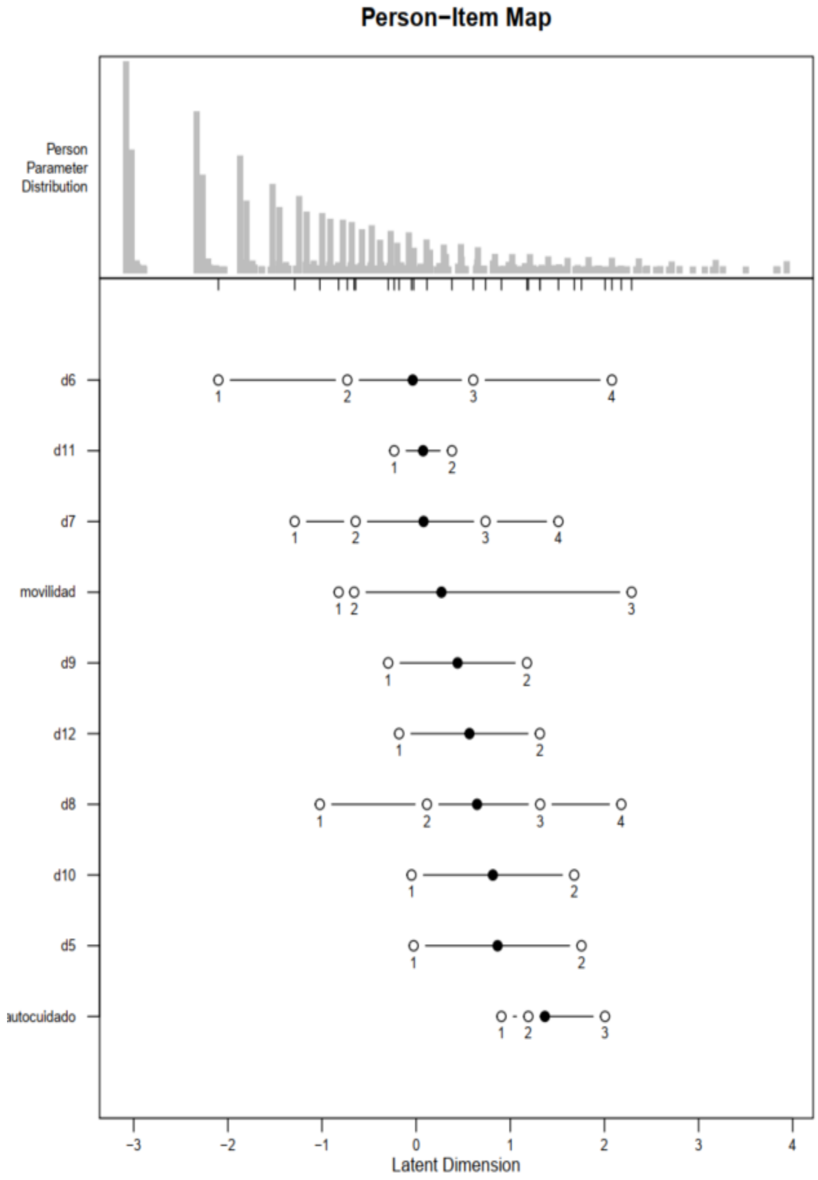

Fuente: Elaboración propia con base de datos ENDIDE 2022

e. Funcionamiento diferencial del ítem e invarianza (de grupo)

El principio de invarianza, común a todos los modelos de la TRI, establece que los parámetros estimados no varían en función de las características de los individuos a los cuales se les administre la escala o instrumento (Tennant, A., 2007). Un caso ejemplificador es el análisis de los datos según el sexo de los entrevistados(as), identificando si los patrones de respuesta son similares o no. En este escenario, si se identifican diferencias significativas en los parámetros de hombres y mujeres, ello podría indicar una distorsión en la métrica que debe ser abordada con una estrategia particular. Si el comportamiento de la métrica difiere entre grupos de interés, se conoce como "Funcionamiento diferencial del ítem" (DIF, por sus siglas en inglés).

Para probar la invarianza de los ítems se debe analizar los resultados del modelo según grupos de interés. Para las escalas métricas se sugiere estudiar la invarianza por edad y sexo, sin embargo, para la escala de capacidad y desempeño, dado que la habilidad en ambas métricas varía producto del envejecimiento, el hecho de que los ítems muestren DIF por edad significa una buena discriminación de los ítems utilizados.

El análisis de invarianza se realiza a través de la comparación de la métrica original versus la métrica que considera estudiar la invarianza en las variables de interés, por ejemplo, sexo. Posteriormente, se realiza una regresión lineal de la métrica original en construcción con la métrica que tiene parámetros separados para los ítems que mostraron funcionamiento diferencial. En el caso de las métricas de desempeño y capacidad, los resultados mostraron que la variable sexo no afecta las métricas originales, validando su uso y el cumplimiento del principio de invarianza de los ítems de cada escala.

Como ejemplo de lo señalado, la Figura 3 muestra la comparación de los puntajes brutos de ENDIDE de la métrica de capacidad (sin DIF) en contraste con la métrica de capacidad construida asumiendo DIF por sexo, este es un procedimiento en donde se realiza una regresión lineal para estudiar la relación entre ambas, permitiendo identificar diferencias en las puntuaciones de una y otra.

Figura 3: Regresión Líneal para la medición de la Invarianza

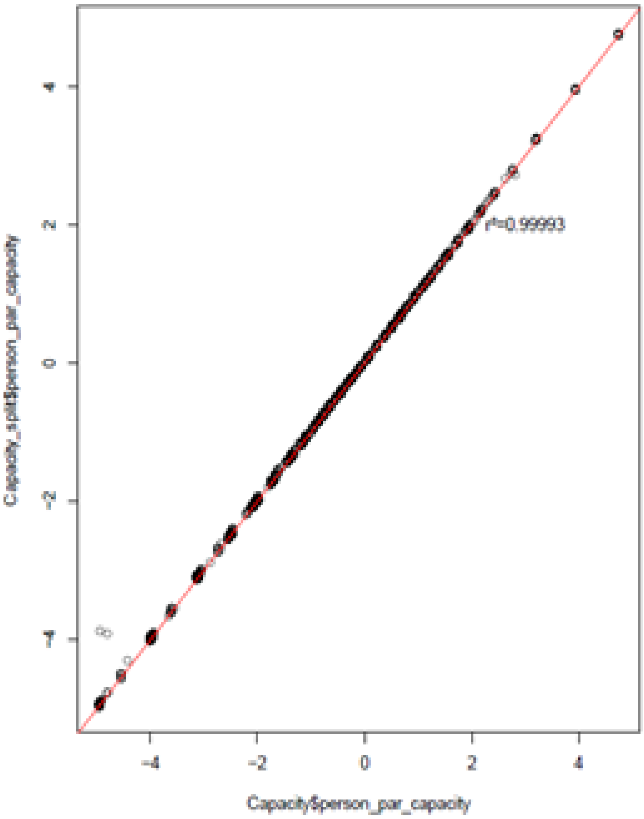

Fuente: Elaboración Propia con base de datos ENDIDE 2022

Tras realizar pruebas de DIF para las métricas de capacidad y desempeño obtenidas con los modelos finales de ENDIDE, fue posible validar el cumplimiento del principio de invarianza.

f. Bondad de ajuste del modelo y confiabilidad

En adición a la comprobación de los supuestos anteriormente mencionados, cada vez que se analiza la construcción del Modelo, se debe evaluar la calidad de su ajuste. Para lo anterior es importante observar distintas pruebas que determinan las características del modelo, de las puntuaciones de las personas y de los ítems. Este análisis debe considerar lo siguiente:

- Ajuste de los ítems al modelo: La bondad describe lo bien que ajustan las observaciones de los Ítems dentro del modelo de Rasch. En este caso existen dos parámetros que permiten su análisis, los indicadores *outfit* e *infit* (Bond, T.G., Fox, C.M., 2007). Estos indicadores permiten, por un lado, identificar valores atípicos dentro de los patrones de respuesta y además,

identificar cuándo un ítem está sobreestimando o subestimando el parámetro de medición, que en este caso corresponde a las escalas de Capacidad y Desempeño.

Para los estadísticos *infit* y *outfit* se espera cifras lo más cercanas posible a 1, siendo aceptables valores entre 0,5 y 1,5. De acuerdo con los lineamientos entregados por la OMS, si los valores de estos indicadores son inferiores a 0,5, las medidas de fiabilidad pueden ser engañosamente altas, pero las puntuaciones pueden ser utilizadas. Si los valores se encuentran entre 1,5 y 2,0, se considera una señal de advertencia de que los ítems pueden estar causando problemas y se recomienda ajustar los datos para mejorar el ajuste.

Los resultados de ENDIDE de las métricas de capacidad y desempeño presentaron valores de ajuste de los indicadores *infit* y *outfit* en el rango 0,4 y 1,4, concentrándose mayoritariamente en el rango 0,8 y 1,2, validando con ello el adecuado ajuste al modelo.

- Confiabilidad del Modelo General: Para analizar la confiabilidad de las puntuaciones de las personas obtenidas con el modelo de Rasch, se debe utilizar el índice de separación de las personas (PSI), el cual es un equivalente al alfa de Cronbach pero aplicado para analizar la consistencia de los parámetros de las personas obtenidos al aplicar el modelo de Rasch. Por lo general, valores ≥ 0,7 se consideran aceptables, valores ≥ 0,8 buenos para grupo y ≥ 0,9 para uso individual (Tennant, A., 2007).

  En el caso de ENDIDE, el PSI obtenido fue de 0,7 y 0,8 para las métricas de capacidad y desempeño respectivamente.

Tras medir y evaluar la confiabilidad de las respuestas en el instrumento y la confiabilidad de las puntuaciones (escala métrica), los resultados de ENDIDE permitieron validar el ajuste de los modelos finales de las métricas de capacidad y desempeño.

Considerando los supuestos y criterios de evaluación presentados en esta sección, los datos de ENDIDE fueron analizados, permitiendo la construcción de las métricas de capacidad y desempeño a partir del Modelo de Crédito Parcial de Rasch. Los modelos finales con los cuales se construyeron ambas métricas cumplen con todos los supuestos y criterios expuestos.

5. Determinación de Niveles de Dificultad

Las métricas de capacidad y desempeño fueron construidas a partir del Modelo de Crédito Parcial de Rasch, haciendo uso del paquete estadístico *whomds* desarrollado por la Organización Mundial de la Salud en el software *R*.

La métrica resultante de la aplicación del modelo de Crédito Parcial de Rasch se expresa en puntajes logit, con un rango que varía entre valores -4 y 4 aproximadamente. Para facilitar la interpretación del puntaje de las métricas, se llevó a cabo una transformación lineal de los puntajes a valores en el rango 0 a 100. De esta manera, personas con valores cercanos a 0 en la métrica de desempeño, por ejemplo, corresponderán a personas con bajas o nulas dificultades en su funcionamiento considerando su entorno real. Mientras que personas con valores cercanos a 100, corresponderán a personas con dificultades severas en su desempeño funcional.

Una vez estimadas las métricas continuas de capacidad y desempeño, se determinan niveles de dificultad a través de la aplicación de puntos de corte. Para ello se aplican los puntos de corte recomendados por la OMS, los cuales se basan en la distribución de los puntajes. La Tabla 17 presenta los puntos de corte y criterios para ordinalizar las métricas de capacidad y desempeño.

Tabla 17. Criterios de puntos de corte para métricas capacidad y desempeño

Nivel	Criterio
Sin dificultad	>= 0 y < (media – 1 desviación estándar)
Dificultad Leve	>= (media – 1 desviación estándar) y < media
Dificultad Moderada	>= media y < (media + 1 desviación estándar)
Dificultad Severa	>= (media + 1 desviación estándar)

Fuente: Organización Mundial de la Salud, Guía de implementación: Encuesta Modelo de Discapacidad Versión Corta, 2018.

Los resultados de la implementación de estos umbrales en población adulta se presentan en las Tablas 18 y 19.

Tabla 18. Resultados puntos de corte para la métrica capacidad en personas adultas (18 años y más)

Nivel de dificultad en Capacidad	Puntos de corte	N	%
Sin dificultad	0 - 9,63	3.859.132	25,1
Dificultad Leve	9,64 - 24,99	3.158.496	20,6
Dificultad Moderada	24,99 - 40,35	6.093.239	39,7
Dificultad Severa	40,35 - 100	2.245.093	14,6

Fuente: Elaboración propia con base de datos ENDIDE 2022

Tabla 19. Resultados puntos de corte para la métrica desempeño en personas adultas (18 años y más)

Nivel de dificultad en Desempeño	Puntos de corte	N	%
Sin dificultad	0	4.075.369	26,5
Dificultad Leve	0,01 - 18,95	4.781.991	31,1
Dificultad Moderada	18,95 - 38,19	3.794.707	24,7
Dificultad Severa	38,19 - 100	2.703.893	17,6

Fuente: Elaboración propia con base de datos ENDIDE 2022

6. Determinación de la Prevalencia de Discapacidad

Una vez que las métricas fueron construidas y validadas, y se diferencian niveles de dificultad, la metodología para determinar la situación de discapacidad y graduarla en niveles de severidad, se realiza en dos etapas. En primer lugar, mediante la métrica de desempeño se identifica a la población adulta con discapacidad (personas de 18 años o más con dificultades severas de desempeño) quienes, debido a sus condiciones de salud y a barreras de su entorno, presentan significativas restricciones en su participación en distintos ámbitos de la sociedad. En segundo término, se analiza en qué lugar se ubican las personas con discapacidad, a lo largo de la escala de capacidad. Así, las personas con discapacidad que coincidentemente tienen problemas severos en su capacidad se consideran "en situación de discapacidad severa", pues tienen mayores dificultades debido a condiciones de salud más complejas. Análogamente, se consideran "en situación de discapacidad leve a moderada" aquellas personas con discapacidad que tienen problemas leves a moderados en su capacidad. Sobre la base de esta metodología, la Tabla 20 informa la distribución de la población adulta según situación y grado de discapacidad.

Tabla 20: Situación de discapacidad según niveles de severidad

Situación de Discapacidad según nivel de severidad	N	%
Personas sin Discapacidad	12.652.067	82,4%
Personas con Discapacidad	2.703.893	17,6%
• *Personas con Discapacidad Leve a Moderada*	*957.058*	*6,2%*
• *Personas con Discapacidad Severa*	*1.746.835*	*11,4%*
Total	15.355.960	100,0%

Fuente: Elaboración propia con base de datos ENDIDE 2022

Al respecto, cabe destacar que se ha decidido utilizar el concepto "persona sin discapacidad", lo cual no significa que las personas clasificadas en esta categoría no experimenten dificultades en su desempeño. Dentro del 82,4% de personas clasificadas "sin discapacidad", se incluye a personas con riesgo de estar en situación de discapacidad (personas con dificultades moderadas en su desempeño, que representan el 24,7% de la población adulta) y a personas con dificultades leves en su desempeño (31,1% de las personas de 18 años y más). Sólo un 26,5% de la población no tiene dificultades en desempeño.

A pesar de lo antes señalado, a fin de presentar brechas e identificar un grupo prioritario para la política pública, se ha escogido el grupo con dificultad severa en desempeño como "personas con discapacidad", y los análisis del estudio se han realizado para este grupo.

Anexo 1: Códigos de Disposición Final de casos, levantamiento ENDIDE 2022

CDF	Glosa
**Entrevistado, elegible**	
110	Entrevista completa
**No entrevistado, elegible**	
200	Se agenda entrevista
211	Se rechazó la entrevista
212	Se interrumpió la entrevista
223	Se impidió acceso a la vivienda
224	Vivienda ocupada sin moradores presentes
225-0	Entrevistado(a) no puede atender
225-1	Entrevistado(a) se cambió a domicilio conocido
225-2	Entrevistado(a) se cambió a domicilio desconocido
225-3	Entrevistado(a) se fue del país
231	Muerte del entrevistado(a)
232	Casos especiales (personas bajo los efectos del alcohol, drogas u otras situaciones especiales, que no estén en condiciones de dar información adecuada)
236	Otra razón, elegible. Especifique
**No entrevistado, elegibilidad desconocida**	
317	Área peligrosa o de difícil acceso
318	No fue posible localizar la dirección
390	Otra razón de elegibilidad desconocida
**No elegible**	
400	Persona ya no habita en la vivienda
410	Fuera de muestra (persona ya no está dentro del rango de edad)
411	Entrevistado(a) nunca ha vivido en la vivienda
412	Otra razón, entrevistado(a) no elegible. Especifique
451	Empresa, oficina de gobierno u otra organización
452	Institución (Hospital, cárcel, asilo de ancianos, etc.)
453	Dormitorio colectivo (Militar, de trabajo, internado)
454	Vivienda en demolición, incendiada, destruida o erradicada
461	Vivienda particular desocupada
462	Vivienda de veraneo o de uso temporal
463	Otra razón, no elegible

BIBLIOGRAFÍA

- *Backhoff, et al. (2022). Uso del modelo de crédito parcial de Rasch y Masters en la evaluación de competencias matemáticas. REICE. Revista Iberoamericana sobre Calidad, Eficacia y Cambio en Educación, 20(1), 41-55.*]
- Bond, T.G.; Fox, C.M. (2007): Applying the Rasch Model: Fundamental Measurement in the Human Sciences, 2nd ed.; Lawrence Erlbaum Associates, Inc.: Mahwah, NK, USA.
- Buja, A.; Eyuboglu, N. (1992): Remarks on parallel analysis. Multivar. Behav. Res. 27, 509–540.
- Cortada de Kohan, N. (2004). Teoría de Respuesta al Ítem: supuestos básicos. *Revista Evaluar*, *4*(1), 95–110.
- Groves, Fowler, F. J., Couper, M. P., & Lepkowski, J. M., Singer, E., & Tourangeau, R. (2004). Survey Methodology. Jonh Wiley & Sons, Inc.
- INE. (2020). Análisis del efecto del COVID-19 sobre el diseño muestral y las estimaciones de la ENE en EFM 2020. Documentos de Trabajo.
- INE. (2020b). La nueva metodología de calibración de la Encuesta Nacional de Empleo: método de calibración Raking.
- Masters, G.N. A Rasch model for partial credit scoring (1982): Psychometrika, 47, 149-174.
- OMS. (2018). Guía de implementación: Encuesta Modelo de Discapacidad Versión Corta.
- OMS. (2001): Clasificación Internacional del Funcionamiento, de la Discapacidad y de la Salud. Madrid, España: Grafo S.A.
- Primi, Tatiana & Primi, Ricardo. (2014). Rasch-Master's Partial Credit Model in the Assessment of Children's Creativity in Drawings. The Spanish journal of psychology. 17. E35. 10.1017/sjp.2014.36.
- Reeve, B.B.; Hays, R.D.; Bjorner, J.B.; Cook, K.F.; Crane, P.K.; Teresi, J.A.; Thissen, D.; Revicki, D.A.; Weiss, D.J.; Hambleton, R.K.; et al. (2007): Pychometric evaluation and calibration of helth-related quality of life item banks: Plans for the patient-reported outcomes measurement information system (PROMIS). Med. Care, 45, S22–S31
- Särndal, C. E. (2007). The calibration approach in survey theory and practice. Survey Methodology, 33(2), 99-119.
- Tennant A. (2007): DIF matters: A practical approach to test if Differential Item Functioning makes a difference, Rasch Measurement Transactions, 20 :4 p. 1082-84.
