# Supplementary material for: The Burden of Chronic Pain on Women: A Secondary Analysis of Data From the National Study on Disability (ENDISC) in Chile
Source: Eur J Pain. 2025 Jul 15;29(7):e70080. doi: 10.1002/ejp.70080 (PMC12261274; doi:10.1002/ejp.70080)
Supplement: Supplementary file 2 — Data S2. [file EJP-29-0-s002.pdf]

Ministerio de Desarrollo Social y Familia
Gobierno de Chile

SENADIS
Ministerio de Desarrollo Social y Familia
Gobierno de Chile

SENAMA
Ministerio de Desarrollo Social y Familia
Gobierno de Chile

ENDIDE

Encuesta de Discapacidad y Dependencia 2022

Libro de Códigos

Junio 2023

ID	*Nombre variable*	*Etiqueta de variable*	*Valores*	*Etiquetas de valores*	*Frecuencia*
1	id_vivienda	Identificador de la vivienda	Válidos		83.747
2	orden_selec_vivienda	Orden de la(s) persona(s) seleccionada(s) en la vivienda	Rango: 1 - 7		
3	id_hogar	Identificador del hogar	Válidos		83.747
4	orden_persona_hogar	Orden de la persona en el hogar	Rango: 1 - 14		
5	id_persona_hogar	Identificador de personas en el hogar	Válidos		83.747
6	confirma_seleccionado	Personas seleccionadas en la vivienda	0	No seleccionado	47.102
			1	Seleccionado	35.536
7	folio	Identificador de cada persona seleccionada en la vivienda	Válidos		35.536
8	region	Región	1	Región de Tarapacá	2.936
			2	Región de Antofagasta	3.917
			3	Región de Atacama	3.424
			4	Región de Coquimbo	3.891
			5	Región de Valparaíso	8.357
			6	Región de O'Higgins	5.560
			7	Región del Maule	5.589
			8	Región del Biobío	8.562
			9	Región de la Araucanía	5.560
			10	Región de Los Lagos	4.839
			11	Región de Aysén	2.309
			12	Región de Magallanes	2.640
			13	Región Metropolitana	17.624
			14	Región de Los Ríos	2.812
			15	Región de Arica y Parinacota	2.957
			16	Región de Ñuble	2.770
9	zona	Zona	1	Urbano	71.302
			2	Rural	12.445
10	estrato	Estrato	Rango: 1 - 32		
11	cod_upm	Unidad primaria de muestreo	Válidos		35.536
12	fexp	Factor de expansión personas seleccionadas en la muestra	Rango: 24 - 15.246		
13	forma_ent_hogar_inicio	Forma de inicio del cuestionario hogar	1	Responderá la encuesta por sí mismo(a)	24.275
			2	Responderá la encuesta con ayuda de otra persona	1.327
			3	Responderá la encuesta con ayuda del sistema Vi-Sor	0
14	sexo	¿Es [NOMBRE] hombre o mujer?	1	Hombre	39.466
			2	Mujer	44.281
15	edad	¿Qué edad tiene [NOMBRE]?	Rango: 0 - 106		
16	pco1_a	¿Quién es el jefe o jefa de este hogar?	0	No es jefe(a) de hogar	44.750
			1	Jefe(a) de hogar	25.602
17	pco1_b	¿Qué relación tiene [NOMBRE] con [NOMBRE JEFE(A) DE HOGAR]?	2	Esposo(a) o pareja de distinto sexo	14.854
			3	Esposo(a) o pareja de igual sexo	55
			4	Hijo(a) de ambos	16.034
			5	Hijo(a) sólo del jefe(a)	12.339
			6	Hijo(a) sólo del esposo(a) o pareja	887
			7	Padre o madre	1.275
			8	Suegro(a)	456
			9	Yerno o nuera	1.218
			10	Nieto(a)	6.228
			11	Hermano(a)	1.365
			12	Cuñado(a)	291
			13	Otro familiar	2.515
			14	No familiar	576
			15	Servicio doméstico puertas adentro	52
18	pco1	Parentesco con el jefe o jefa de hogar	1	Jefe(a) de hogar	25.602
			2	Esposo(a) o pareja de distinto sexo	14.854
			3	Esposo(a) o pareja de igual sexo	55

ID	Nombre variable	Etiqueta de variable	Valores	Etiquetas de valores	Frecuencia
			4	Hijo(a) de ambos	16.034
			5	Hijo(a) sólo del jefe(a)	12.339
			6	Hijo(a) sólo del esposo(a) o pareja	887
			7	Padre o madre	1.275
			8	Suegro(a)	456
			9	Yerno o nuera	1.218
			10	Nieto(a)	6.228
			11	Hermano(a)	1.365
			12	Cuñado(a)	291
			13	Otro familiar	2.515
			14	No familiar	576
			15	Servicio doméstico puertas adentro	52
19	ecivil	¿Cuál es el estado conyugal o civil actual de [NOMBRE]?	1	Casado(a)	23.299
			2	Conviviente o pareja sin acuerdo de unión civil	8.601
			3	Conviviente civil (con acuerdo de unión civil)	182
			4	Anulado(a)	69
			5	Separado(a)	3.116
			6	Divorciado(a)	1.757
			7	Viudo(a)	3.763
			8	Soltero(a)	29.565
20	inm	Cuando [NOM. SELEC] nació, ¿en qué país vivía su madre?	-88	No sabe	58
			1	En este país	34.017
			2	En otro país. Especifique	1.461
21	inm_esp	País donde vivía la madre. Codificado	-88	No sabe	1
			1	Perú	287
			2	Venezuela	477
			3	Haití	83
			4	Colombia	184
			5	Bolivia	188
			6	Argentina	80
			7	Ecuador	39
			8	Brasil	22
			9	República Dominicana	15
			10	Paraguay	8
			11	Uruguay	6
			12	Estados Unidos	13
			13	España	13
			14	Rusia	3
			15	Cuba	8
			16	Santo Domingo	0
			17	Costa Rica	1
			18	México	1
			19	Alemania	7
			20	Italia	1
			21	Portugal	1
			22	Bielorrusia	1
			23	Marruecos	1
			24	Francia	3
			25	Palestina	1
			26	El Líbano	2
			27	Austria	1
			28	Suiza	1
			29	Canadá	3
			30	Nicaragua	1
			31	Noruega	1
			32	República Checa	1
			33	Guatemala	1
			34	Kenia	1
			35	Malta	1

ID	Nombre variable	Etiqueta de variable	Valores	Etiquetas de valores	Frecuencia
			36	Sudáfrica	1
			37	Lituania	1
			38	Dinamarca	1
			39	Holanda	0
			40	Israel	0
			41	Nueva Zelanda	0
			42	Bosnia	0
			43	Australia	0
			44	Inglaterra	0
			45	Panamá	0
			46	India	1
22	ind	En Chile, la ley reconoce diez pueblos indígenas. ¿Pertenece [NOMBRE]…?	1	Aimara	560
			2	Rapa-Nui o Pascuenses	2
			3	Quechua	81
			4	Mapuche	3.424
			5	Atacameño (Likan-Antai)	72
			6	Collas	61
			7	Kawashkar o Alacalufes	6
			8	Yámana o Yagán	4
			9	Diaguita	341
			10	Chango	40
			11	No pertenece a ninguno de estos pueblos indígenas	30.945
23	e1	¿Participa en clases virtuales o presenciales impartidas por un establecimiento?	1	Sí	23.805
			2	No	59.942
24	e2	¿Cuál es la principal razón por la cual [NOM. SELEC] no asiste…? 2 a 6 años	1	No es necesario porque lo(a) cuidan en la casa	102
			2	No me parece necesario que asista a esta edad	20
			3	Desconfío del cuidado que recibiría	6
			4	Se enfermaría mucho	16
			5	Dada su discapacidad, prefiero que no asista	4
			6	Dificultad económica	0
			7	No fue priorizado por el establecimiento	4
			8	No hay matrícula (vacantes)	14
			9	No lo(a) aceptan	5
			10	Dada su discapacidad, el establecimiento educacional no lo acepta o no ofrecen las condiciones adecuadas	1
			11	No existe establecimiento cercano	3
			12	Problemas de accesibilidad (ej. establecimiento con barreras arquitectónicas, dificultad para acceder a establecimiento o salir de casa)	1
			13	Problemas de transporte	0
			14	Por la pandemia COVID-19	4
			77	Otra razón	16
25	e3	¿Cuál es la principal razón por la cual [NOM. SELEC] no asiste…? 7 a 30 años	1	Ayuda en la casa o quehaceres del hogar	130
			2	Embarazo	48
			3	Ayuda o se dedica al cuidado de alguien del hogar	122
			4	Tiene una enfermedad o condición de salud que lo inhabilita	70
			5	Requiere establecimiento de educación especial	15

ID	Nombre variable	Etiqueta de variable	Valores	Etiquetas de valores	Frecuencia
			6	Problemas familiares	38
			7	No le interesa	122
			8	Terminó de estudiar	1.800
			9	A su edad no le sirve estudiar o no conoce la manera para completar sus estudios	8
			10	Se encuentra preparando la Prueba de Acceso a la Educación Superior (PAES o Ex PSU) en preuniversitario o por su cuenta	46
			11	Dificultad económica	235
			12	Trabaja o busca trabajo	1.100
			13	Problemas de rendimiento o cancelación de matrícula	24
			14	No existe establecimiento cercano	6
			15	Problemas de accesibilidad (ej. establecimiento con barreras arquitectónicas, dificultad para acceder a establecimiento o salir de casa)	2
			16	Problemas de transporte	3
			17	Por la pandemia COVID-19	22
			77	Otra razón	158
26	e4_asiste	¿Cuál es el nivel educacional actual de [NOMBRE]? (Asiste)	2	Sala cuna	260
			3	Jardín Infantil (Medio menor y Medio mayor)	1.053
			4	Prekínder o Kínder (Transición menor y Transición Mayor)	1.627
			5	Educación Especial (Diferencial)	185
			7	Educación Básica	9.152
			9	Educación Media Científico-Humanista	4.320
			11	Educación Media Técnica Profesional	1.113
			12	Técnico Nivel Superior Incompleto (Carreras 1 a 4 años)	1.538
			14	Profesional Incompleto (Carreras 4 o más años)	4.337
			16	Postgrado Incompleto	220
27	e4_no_asiste	¿Cuál es el nivel educacional más alto alcanzado de [NOMBRE]? (No asiste)	1	Nunca asistió	2.655
			2	Sala cuna	43
			3	Jardín Infantil (Medio menor y Medio mayor)	46
			4	Prekínder o Kínder (Transición menor y Transición Mayor)	12
			5	Educación Especial (Diferencial)	305
			6	Primaria o Preparatoria (Sistema antiguo)	2.825
			7	Educación Básica	11.513
			8	Humanidades (Sistema Antiguo)	1.901
			9	Educación Media Científico-Humanista	19.483
			10	Técnica, Comercial, Industrial o Normalista (Sistema Antiguo)	612
			11	Educación Media Técnica Profesional	4.910
			12	Técnico Nivel Superior Incompleto (Carreras 1 a 4 años)	1.287
			13	Técnico Nivel Superior Completo (Carreras 1 a 4 años)	4.715
			14	Profesional Incompleto (Carreras 4 o más años)	1.757

ID	Nombre variable	Etiqueta de variable	Valores	Etiquetas de valores	Frecuencia
			15	Profesional Completo (Carreras 4 o más años)	7.169
			16	Postgrado Incompleto	41
			17	Postgrado Completo	668
28	e4	Nivel educacional de [NOMBRE] (consolidado)	1	Nunca asistió	2.655
			2	Sala cuna	303
			3	Jardín Infantil (Medio menor y Medio mayor)	1.099
			4	Prekínder o Kínder (Transición menor y Transición Mayor)	1.639
			5	Educación Especial (Diferencial)	490
			6	Primaria o Preparatoria (Sistema antiguo)	2.825
			7	Educación Básica	20.665
			8	Humanidades (Sistema Antiguo)	1.901
			9	Educación Media Científico-Humanista	23.803
			10	Técnica, Comercial, Industrial o Normalista (Sistema Antiguo)	612
			11	Educación Media Técnica Profesional	6.023
			12	Técnico Nivel Superior Incompleto (Carreras 1 a 4 años)	2.825
			13	Técnico Nivel Superior Completo (Carreras 1 a 4 años)	4.715
			14	Profesional Incompleto (Carreras 4 o más años)	6.094
			15	Profesional Completo (Carreras 4 o más años)	7.169
			16	Postgrado Incompleto	261
			17	Postgrado Completo	668
29	e5_asiste	En ese nivel educacional, ¿cuál es el curso que [NOMBRE] cursa…? (Asiste)	-88	No sabe	71
			1	1	3.783
			2	2	3.782
			3	3	3.317
			4	4	3.789
			5	5	2.043
			6	6	1.321
			7	7	1.267
			8	8	1.265
			9	9	20
			10	10	14
			11	11	1
			12	12	2
			13	13	0
			14	14	0
			15	15	2
			16	16	0
			17	17	1
			18	18	1
			19	19	0
			20	20	1
30	e5_no_asiste	En ese nivel educacional, ¿cuál fue el último curso que [NOMBRE]…? (No asiste)	-88	No sabe	550
			1	1	2.491
			2	2	5.407
			3	3	5.837
			4	4	23.856
			5	5	6.880
			6	6	4.520
			7	7	1.337
			8	8	5.452
			9	9	67
			10	10	391

ID	*Nombre variable*	*Etiqueta de variable*	*Valores*	*Etiquetas de valores*	*Frecuencia*
			11	11	16
			12	12	22
			13	13	8
			14	14	13
			15	15	0
			16	16	5
			17	17	6
			18	18	2
			19	19	1
			20	20	20
31	e5	Curso de [NOMBRE] (consolidado)	-88	No sabe	621
			1	1	6.274
			2	2	9.189
			3	3	9.154
			4	4	27.645
			5	5	8.923
			6	6	5.841
			7	7	2.604
			8	8	6.717
			9	9	87
			10	10	405
			11	11	17
			12	12	24
			13	13	8
			14	14	13
			15	15	2
			16	16	5
			17	17	7
			18	18	3
			19	19	1
			20	20	21
32	o1	La semana pasada, ¿[NOMBRE] trabajó al menos una hora, sin considerar…?	1	Sí	31.940
			2	No	37.279
33	o2	Aunque no trabajó la semana pasada, ¿[NOMBRE] realizó alguna actividad…?	1	Sí	982
			2	No	36.297
34	o3	Aunque no trabajó la semana pasada, ¿[NOMBRE] tenía algún empleo, negocio…?	1	Sí	1.214
			2	No	35.083
35	o4	¿[NOM. SELEC] ha trabajado alguna vez?	1	Sí	10.669
			2	No	4.639
36	o5	Si le ofrecieran un trabajo, ¿[NOM. SELEC] estaría disponible para comenzar...?	1	Sí, ahora mismo	3.055
			2	Sí, en otra época del año	1.770
			3	No	10.483
37	o6	¿[NOMBRE] buscó trabajo remunerado o realizó alguna gestión para iniciar…?	1	Sí	2.947
			2	No	32.136
38	o7	¿Cuál es la razón principal por la que [NOM. SELEC] no buscó trabajo o realizó…?	1	Consiguió trabajo que empezará pronto o iniciará pronto una actividad por cuenta propia	58
			2	Está esperando resultado de gestiones ya emprendidas	154
			3	Ayuda o se dedica al cuidado de alguien del hogar	1.250
			4	Tiene una enfermedad o condición de salud que lo inhabilita	2.236
			5	Piensa que nadie le dará trabajo porque no cuenta con la capacitación requerida, por su edad, etc.	90
			6	Las condiciones laborales (sueldo, horarios, distancias, etc.) no le acomodan	77
			7	Ofrecen sueldos muy bajos	60
			8	Quehaceres del hogar	2.367

ID	Nombre variable	Etiqueta de variable	Valores	Etiquetas de valores	Frecuencia
			9	Estudiante	2.733
			10	Jubilado(a), montepiado(a) o pensionado(a)	3.661
			11	Tiene otra fuente de ingreso (Seguro de Cesantía, mesadas, rentas, transferencias del Estado, etc.)	30
			12	Por la pandemia COVID-19	88
			13	Se cansó de buscar o cree que no hay trabajo disponible	60
			14	Busca cuando realmente lo necesita o tiene trabajo esporádico	81
			15	No tiene interés en trabajar	238
			16	Razones de accesibilidad al lugar de trabajo (infraestructura, equipamiento, dispositivos de ayuda)	12
			77	Otra razón	599
39	o8	¿Cuántas semanas buscó o ha estado buscando trabajo [NOM. SELEC]?	-88	No sabe	110
40	rama4_caenes	Actividad económica empresa a 4 dígitos según clasificación CAENES	-99	No responde	540
			-88	No bien especificado	0
			101	Cultivo de plantas no perennes	728
			102	Cultivo de plantas perennes	685
			103	Ganadería	257
			199	Otras actividades relacionadas con la agricultura, ganadería, caza y actividades de apoyo n.c.p.	277
			201	Silvicultura, extracción de madera y actividades de servicios conexas	206
			301	Pesca, acuicultura y actividades de servicios conexas	473
			401	Extracción y procesamiento de cobre	687
			501	Extracción de carbón de piedra y lignito	3
			601	Extracción de petróleo crudo y gas natural	37
			701	Extracción de minerales metalíferos, excepto cobre	28
			801	Extracción y procesamiento de litio	13
			899	Extracción de otras minas y canteras n.c.p., excepto litio	46
			901	Actividades de apoyo para la explotación de minas y canteras	235
			1001	Elaboración y conservación de carne, pescado, crustáceos y moluscos	189
			1002	Elaboración y conservación de frutas, legumbres y hortalizas; aceites y grasas	63
			1003	Elaboración de productos lácteos	49
			1004	Elaboración de productos de panadería	496
			1099	Elaboración de otros productos alimenticios n.c.p.	209
			1101	Elaboración de bebidas alcohólicas y no alcohólicas, excepto vino	109
			1102	Elaboración de vinos	70

ID	Nombre variable	Etiqueta de variable	Valores	Etiquetas de valores	Frecuencia
			1201	Elaboración de productos de tabaco	0
			1401	Fabricación de productos textiles, fabricación de prendas de vestir, productos de cuero y productos conexos	248
			1601	Producción de madera y fabricación de productos de madera y corcho, excepto muebles; fabricación de artículos de paja y de materiales trenzados	227
			1701	Fabricación de papel y productos de papel	91
			1801	Impresión y servicios de apoyo; reproducción de grabaciones	88
			1901	Fabricación de coque y productos de la refinación del petróleo	0
			2001	Fabricación de sustancias y productos químicos	94
			2101	Fabricación de productos farmacéuticos, sustancias químicas medicinales y productos botánicos de uso farmacéutico	42
			2201	Fabricación de productos de caucho y de plástico	72
			2301	Fabricación de otros productos minerales no metálicos	70
			2401	Fabricación de metales comunes	33
			2501	Fabricación de productos elaborados de metal y servicios de trabajos de metales, excepto máquinas y equipos	337
			2701	Fabricación de productos informáticos, electrónicos, ópticos, equipos eléctricos y maquinaria	47
			3001	Fabricación de vehículos automotores, remolques y semirremolques y otros tipos de equipo de transporte	37
			3101	Fabricación de muebles	203
			3201	Otras industrias manufactureras	111
			3301	Mantenimiento, reparación e instalación de maquinaria y equipos	162
			3501	Electricidad, gas, vapor y aire acondicionado	136
			3601	Captación, tratamiento y distribución de agua	109
			3701	Evacuación de aguas residuales	7
			3801	Recogida, tratamiento y eliminación de desechos	62
			3802	Recuperación de materiales	37
			3901	Actividades de descontaminación y otros servicios de gestión de desechos	2
			4101	Construcción de edificios	2.123
			4201	Obras de ingeniería civil	229
			4301	Actividades especializadas de construcción	795

ID	Nombre variable	Etiqueta de variable	Valores	Etiquetas de valores	Frecuencia
			4501	Comercio y reparación de vehículos automotores y motocicletas	823
			4801	Comercio de intermediación	77
			4802	Comercio de materias primas agropecuarias y animales vivos	43
			4803	Comercio especializado de alimentos, bebidas y tabaco	1.281
			4804	Comercio de productos textiles, prendas de vestir y calzado, y enseres domésticos	1.036
			4805	Comercio de materiales de construcción, artículos de ferretería, gasfitería pinturas, espejos y artículos para revestimiento	336
			4806	Comercio de combustible para vehículos y productos conexos para uso doméstico	206
			4807	Comercio de mercaderías n.c.p., incluso mercaderías usadas	209
			4808	Tiendas no especializadas con o sin predominio de la venta de alimentos, bebidas o tabaco	1.133
			4809	Comercio al por menor en mercados, ferias y otros puestos móviles	542
			4810	Comercio al por menor por correo, televisión, internet; comercio ambulante y otros tipos de ventas n.c.p.	454
			4901	Transporte por ferrocarril	25
			4902	Transporte de pasajeros por vía terrestre	1.100
			4903	Transporte de carga por vía terrestre	574
			4904	Transporte por tuberías	0
			5001	Transporte por vía acuática	57
			5101	Transporte por vía aérea	17
			5201	Depósito y almacenamiento	70
			5202	Servicios auxiliares al transporte	259
			5301	Actividades postales y de mensajería	73
			5501	Actividades de alojamiento	205
			5601	Servicios de expendio de comidas y bebidas, excepto por vendedores ambulantes	962
			5602	Servicios de expendio de comidas y bebidas por vendedores ambulantes	229
			5603	Servicios de suministro de comidas para eventos y otras actividades de servicio de comidas	241
			5801	Actividades de edición	7
			5901	Actividades de producción de películas cinematográficas, videos y programas de televisión y grabación de sonido y edición de música	26
			6001	Actividades de programación y transmisión	30

ID	Nombre variable	Etiqueta de variable	Valores	Etiquetas de valores	Frecuencia
			6101	Actividades de telecomunicaciones	192
			6201	Actividades de programación y consultorías informáticas y otras actividades conexas	187
			6301	Actividades de servicios de información	11
			6401	Intermediación financiera y otros servicios financieros, excepto los de seguros y administración de fondos de jubilaciones pensiones	267
			6501	Isapres	17
			6502	Seguros de vida o generales, reaseguros y fondos de pensiones, excepto Isapres	93
			6601	Otras actividades auxiliares relacionadas con los servicios financieros, seguros y fondos de pensiones; otras actividades de gestión de fondos n.c.p.	23
			6699	Administradoras de Fondos de Pensiones (AFP)	41
			6801	Actividades inmobiliarias	130
			6901	Actividades jurídicas y de contabilidad	340
			7001	Actividades de oficinas principales, actividades de consultoría de gestión	28
			7101	Actividades de arquitectura e ingeniería; ensayos y análisis técnicos	237
			7201	Investigación científica y desarrollo	31
			7301	Publicidad y estudios de mercado	114
			7401	Otras actividades profesionales, científicas y técnicas	126
			7501	Actividades veterinarias	37
			7701	Actividades de alquiler y arrendamiento	362
			7801	Actividades de empleo	112
			7901	Agencias de viajes, operadores turísticos y actividades conexas	49
			8001	Actividades de investigación y seguridad	350
			8101	Actividades de servicios de apoyo a edificios y actividades de limpieza en general	397
			8102	Barrido y riego de calles, plazas, caminos, mercados, jardines y parques; actividades de paisajismo y jardinería	161
			8201	Actividades administrativas de oficinas y otras actividades auxiliares de las empresas	178
			8401	Administración del Estado y aplicación de la política económica y social de la comunidad	1.181
			8402	Prestación de servicios a la comunidad en general	458
			8403	Actividades de planes de seguridad social de afiliación obligatoria	13

ID	Nombre variable	Etiqueta de variable	Valores	Etiquetas de valores	Frecuencia
			8501	Enseñanza preescolar, primaria, secundaria científico humanista y técnico profesional, pública y privada	2.553
			8502	Enseñanza superior	454
			8599	Otros tipos de enseñanza	141
			8601	Actividades de hospitales públicos y privados	1.148
			8602	Actividades de médicos y odontólogos	617
			8699	Otras actividades de atención de la salud humana n.c.p.	185
			8701	Actividades de asistencia social en instituciones	109
			8801	Actividades de asistencia social sin alojamiento	98
			9001	Actividades creativas, artísticas y de entretenimiento	83
			9101	Actividades de bibliotecas, archivos, museos y otras actividades culturales	24
			9201	Actividades de juegos de azar y apuestas	54
			9301	Actividades deportivas, de esparcimiento y recreativas	164
			9401	Actividades de asociaciones	347
			9501	Reparación de equipos informáticos	32
			9502	Reparación de equipos de comunicación	5
			9503	Reparación de efectos personales y enseres domésticos, excepto computadores y equipo periférico	140
			9601	Lavado y limpieza, incluida la limpieza en seco, de productos textiles y de piel	40
			9602	Servicios de peluquería y otros tratamientos de belleza	376
			9603	Pompas fúnebres y servicios conexos	43
			9699	Otras actividades de servicios personales n.c.p.	93
			9701	Actividades de los hogares como empleadores de personal doméstico	1.487
			9801	Actividades de los hogares como productores de bienes o servicios para uso propio	28
			9901	Actividades de organizaciones y órganos extraterritoriales	3
41	rama1_caenes	Actividad económica empresa a 1 dígito según clasificación CAENES	-99	No responde	295
			-88	No bien especificado	245
			1	A. Agricultura, Ganadería, Silvicultura y Pesca	2.626
			2	B. Explotación de Minas y Canteras	1.049
			3	C. Industrias Manufactureras	3.050
			4	D. Suministro de Electricidad, Gas, Vapor y Aire Acondicionado	136
			5	E. Suministro de Agua; Evacuación de Aguas Residuales, Gestión de Desechos y Descontaminación	217
			6	F. Construcción	3.144

ID	Nombre variable	Etiqueta de variable	Valores	Etiquetas de valores	Frecuencia
			7	G. Comercio al Por Mayor y al Por Menor; Reparación de Vehículos Automotores y Motocicletas	6.140
			8	H. Transporte y Almacenamiento	2.175
			9	I. Actividades de Alojamiento y de Servicio de Comidas	1.637
			10	J. Información y Comunicaciones	453
			11	K. Actividades Financieras y de Seguros	441
			12	L. Actividades Inmobiliarias	130
			13	M. Actividades Profesionales, Científicas y Técnicas	913
			14	N. Actividades de Servicios Administrativos y de Apoyo	1.609
			15	O. Administración Pública y Defensa; Planes de Seguridad Social de Afiliación Obligatoria	1.652
			16	P. Enseñanza	3.148
			17	Q. Actividades de Atención de la Salud Humana y de Asistencia Social	2.157
			18	R. Actividades Artísticas, de Entretenimiento y Recreativas	325
			19	S. Otras Actividades de Servicios	1.076
			20	T. Actividades de los Hogares como Empleadores; Actividades No diferenciadas de los Hogares como Productores de Bienes y Servicios para Uso propio	1.515
			21	U. Actividades de Organizaciones y Órganos Extraterritoriales	3
42	oficio4_ciuo_08cl	Ocupación a 4 dígitos según clasificación CIUO 08.CL	-99	No responde	0
			-88	No bien especificado	270
			110	Oficiales de las Fuerzas Armadas	7
			210	Suboficiales de las Fuerzas Armadas	26
			310	Otros miembros de las Fuerzas Armadas	39
			1111	Miembros del poder ejecutivo y legislativo	9
			1112	Personal directivo de la administración pública	26
			1113	Jefes de comunidades indígenas	0
			1114	Dirigentes de organizaciones sociales y/o políticas (sindicatos, organizaciones sociales, partidos políticos, entre otras)	4
			1120	Directores y gerentes generales de empresas	9
			1211	Directores, gerentes y administradores de finanzas	11
			1212	Directores, gerentes y administradores de recursos humanos	36
			1213	Directores, gerentes y administradores de políticas empresariales y planificación	3
			1219	Otros directores, gerentes y administradores de servicios administrativos no clasificados previamente	22

ID	Nombre variable	Etiqueta de variable	Valores	Etiquetas de valores	Frecuencia
			1221	Directores, gerentes y administradores de comercialización	29
			1222	Directores, gerentes y administradores de publicidad y relaciones públicas	7
			1223	Directores, gerentes y administradores de investigación y desarrollo	6
			1311	Directores, gerentes y administradores de producción y operaciones agropecuarias y de silvicultura	43
			1312	Directores, gerentes y administradores de producción y operaciones de acuicultura y pesca	8
			1321	Directores, gerentes y administradores de industrias manufactureras	90
			1322	Directores, gerentes y administradores de explotaciones mineras	11
			1323	Directores, gerentes y administradores de empresas de construcción	54
			1324	Directores, gerentes y administradores de empresas de abastecimiento, almacenamiento y distribución	49
			1330	Directores, gerentes y administradores de servicios de tecnología de la información y las comunicaciones	22
			1341	Directores, gerentes y administradores de servicios de cuidados infantiles	2
			1342	Directores, gerentes y administradores de servicios de salud	16
			1343	Directores, gerentes y administradores de servicios de cuidado de adultos mayores	3
			1344	Directores, gerentes y administradores de servicios de bienestar social	1
			1345	Directores, gerentes y administradores de servicios de educación	63
			1346	Directores, gerentes y administradores de servicios financieros	13
			1349	Directores, gerentes y administradores de otros servicios profesionales no clasificados previamente	20
			1411	Directores, gerentes y administradores de hoteles	44
			1412	Directores, gerentes y administradores de restaurantes	78
			1420	Directores, gerentes y administradores de comercios al por mayor y al por menor	124

ID	Nombre variable	Etiqueta de variable	Valores	Etiquetas de valores	Frecuencia
			1431	Directores, gerentes y administradores de centros deportivos, de esparcimiento y culturales	14
			1439	Directores, gerentes y administradores de otros servicios no clasificados previamente	47
			2111	Físicos y astrónomos	7
			2112	Meteorólogos	1
			2113	Químicos	22
			2114	Geólogos y geofísicos	11
			2120	Matemáticos y estadísticos	4
			2131	Biólogos, botánicos, zoólogos, genetistas y farmacólogos	23
			2132	Agrónomos y profesionales del ámbito forestal y pesquero	67
			2133	Profesionales de la protección medioambiental	7
			2134	Bioquímicos	7
			2141	Ingenieros industriales y de producción	66
			2142	Ingenieros civiles, ingenieros en construcción y constructores civiles	206
			2143	Ingenieros medioambientales	18
			2144	Ingenieros mecánicos	69
			2145	Ingenieros químicos	24
			2146	Ingenieros en minas y metalúrgicos	45
			2147	Ingenieros biomédicos	9
			2149	Otros ingenieros no clasificados previamente	162
			2151	Ingenieros eléctricos	55
			2152	Ingenieros electrónicos	12
			2153	Ingenieros en telecomunicaciones	15
			2161	Arquitectos	68
			2162	Arquitectos paisajistas	6
			2163	Diseñadores de productos y de vestuario	20
			2164	Urbanistas e ingenieros de transporte y tránsito	2
			2165	Cartógrafos y agrimensores	5
			2166	Diseñadores gráficos y de multimedia	81
			2211	Médicos generales	52
			2212	Médicos especialistas	39
			2221	Enfermeros profesionales	200
			2222	Profesionales de matronería	34
			2230	Veterinarios	27
			2241	Dentistas	47
			2242	Químicos farmacéuticos	38
			2243	Ingenieros en prevención de riesgos y otros profesionales de la seguridad e higiene laboral y ambiental	130
			2244	Kinesiólogos	77
			2245	Dietistas y nutricionistas	56
			2246	Fonoaudiólogos	39
			2247	Tecnólogos médicos	45
			2248	Terapeutas ocupacionales	33
			2249	Otros profesionales de la salud no clasificados previamente	9

ID	Nombre variable	Etiqueta de variable	Valores	Etiquetas de valores	Frecuencia
			2310	Profesores de la educación superior	180
			2320	Profesores de educación media técnico profesional (especialidades) y de formación laboral	13
			2330	Profesores de educación media	291
			2341	Profesores de educación básica	439
			2342	Educadores de párvulos	181
			2351	Especialistas en métodos pedagógicos	33
			2352	Educadores para necesidades especiales	129
			2353	Otros profesores de idiomas	23
			2354	Otros profesores de música	17
			2355	Otros profesores de artes	14
			2356	Instructores en tecnologías de la información	3
			2359	Otros profesionales de la educación no clasificados previamente	135
			2411	Contadores	234
			2412	Asesores financieros y en inversiones	15
			2413	Analistas financieros	26
			2421	Especialistas y asesores de gestión	52
			2422	Especialistas en políticas de administración	26
			2423	Especialistas en políticas y servicios de personal	77
			2424	Especialistas en formación del personal	10
			2431	Profesionales de la publicidad y la comercialización	78
			2432	Profesionales de las relaciones públicas	13
			2433	Profesionales de ventas técnicas y médicas (excluyendo las TIC)	23
			2434	Profesionales de ventas de tecnología de la información y las comunicaciones (TIC)	13
			2511	Analistas de sistemas	86
			2512	Desarrolladores de software	61
			2513	Desarrolladores web y multimedia	19
			2514	Programadores de aplicaciones	11
			2519	Otros desarrolladores y analistas de software y multimedia no clasificados previamente	22
			2521	Diseñadores y administradores de bases de datos	9
			2522	Administradores de sistemas	5
			2523	Profesionales en redes de computadores	12
			2529	Otros especialistas en bases de datos y en redes de computadores no clasificados previamente	21
			2611	Abogados	132
			2612	Jueces	3
			2619	Otros profesionales del derecho no clasificados previamente	8

ID	Nombre variable	Etiqueta de variable	Valores	Etiquetas de valores	Frecuencia
			2621	Archivistas y curadores de museos	1
			2622	Bibliotecarios y documentalistas de archivos	21
			2631	Economistas	7
			2632	Sociólogos, antropólogos, geógrafos y arqueólogos	15
			2633	Filósofos, historiadores y especialistas en ciencias políticas	1
			2634	Psicólogos	172
			2635	Profesionales del trabajo social	192
			2636	Profesionales religiosos	16
			2641	Autores y otros escritores	11
			2642	Periodistas	53
			2643	Traductores, intérpretes de lengua de señas y lingüistas	14
			2651	Artistas de artes plásticas	23
			2652	Músicos, cantantes y compositores	31
			2653	Bailarines y coreógrafos	4
			2654	Directores de cine, radio y teatro	9
			2655	Actores	5
			2656	Locutores de radio, televisión y otros medios de comunicación	17
			2659	Otros artistas creativos e interpretativos no clasificados previamente	5
			3111	Técnicos en ciencias físicas y químicas	44
			3112	Técnicos en construcción y topógrafos	75
			3113	Técnicos en electricidad	84
			3114	Técnicos en electrónica	25
			3115	Técnicos en ingeniería mecánica	143
			3116	Técnicos en química industrial	2
			3117	Técnicos en ingeniería de minas y metalurgia	18
			3118	Delineantes y dibujantes técnicos	30
			3119	Otros técnicos en ciencias físicas y en ingeniería no clasificados previamente	9
			3121	Supervisores de minas	31
			3122	Supervisores de industrias manufactureras	58
			3123	Supervisores de la construcción	104
			3131	Operadores de instalaciones de producción de energía	7
			3132	Operadores de incineradores y de instalaciones de tratamiento de agua	28
			3133	Controladores de instalaciones de procesamiento de productos químicos	2
			3134	Operadores de instalaciones de refinación de petróleo y gas natural	9
			3135	Controladores de procesos de producción de metales	1
			3139	Técnicos en control de procesos no clasificados previamente	7
			3141	Técnicos en ciencias biológicas (excluyendo la medicina)	4

ID	Nombre variable	Etiqueta de variable	Valores	Etiquetas de valores	Frecuencia
			3142	Técnicos agropecuarios (incluyendo acuícolas)	45
			3143	Técnicos forestales	1
			3151	Oficiales maquinistas en navegación	3
			3152	Capitanes y oficiales de cubierta	11
			3153	Pilotos de aviación	5
			3154	Controladores de tráfico aéreo	3
			3155	Técnicos electrónicos aeronáuticos	2
			3211	Técnicos y auxiliares paramédicos de radiología e imagenología y otros aparatos de diagnóstico y tratamiento médico	14
			3212	Técnicos de laboratorios clínicos y servicios de sangre	23
			3213	Técnicos y asistentes farmacéuticos	22
			3214	Técnicos de prótesis médicas	2
			3215	Laboratoristas dentales o técnicos en prótesis dentales	17
			3216	Auxiliares paramédicos de anatomía patológica	6
			3221	Técnicos y auxiliares paramédicos de enfermería	508
			3222	Técnicos y auxiliares paramédicos de esterilización	4
			3223	Ayudantes de ambulancia	0
			3231	Profesionales y técnicos de las terapias complementarias	6
			3232	Practicantes de la medicina tradicional	5
			3240	Técnicos y asistentes veterinarios	8
			3251	Técnicos y auxiliares paramédicos en odontología	70
			3252	Técnicos en documentación e información sanitaria	1
			3253	Podólogos	24
			3254	Técnicos ópticos y contactólogos	8
			3255	Técnicos y asistentes fisioterapeutas	16
			3256	Técnicos y auxiliares paramédicos de alimentación	10
			3257	Inspectores de la salud y técnicos en prevención de riesgos	57
			3258	Quiroprácticos y osteópatas	0
			3259	Otros técnicos de la salud no clasificados previamente	3
			3311	Agentes de bolsa y otros servicios financieros	48
			3312	Ejecutivos de préstamos y créditos	48
			3313	Técnicos y asistentes en contabilidad	223
			3314	Técnicos de servicios estadísticos y matemáticos	12
			3315	Tasadores	5
			3321	Agentes de seguros y ejecutivos de fondos de pensiones	93

ID	Nombre variable	Etiqueta de variable	Valores	Etiquetas de valores	Frecuencia
			3322	Representantes comerciales (excepto venta de productos y servicios industriales, farmacéuticos y de tecnologías de la información y las comunicaciones)	138
			3323	Agentes responsables de adquisiciones	76
			3324	Corredores comerciales y consignatarios	10
			3331	Agentes de aduana	26
			3332	Organizadores de conferencias y eventos	19
			3333	Agentes de empleo y contratistas de personal	14
			3334	Agentes inmobiliarios	74
			3339	Otros agentes de servicios comerciales no clasificados previamente	6
			3341	Supervisores de oficina	23
			3342	Secretarios jurídicos	26
			3343	Secretarios administrativos y ejecutivos	505
			3344	Secretarios médicos	124
			3351	Inspectores de aduana	2
			3352	Agentes de la administración tributaria	11
			3353	Agentes de servicios de prestaciones sociales	1
			3354	Agentes de servicios de tramitación y entrega de licencias y permisos	13
			3355	Inspectores y detectives de la Policía de Investigaciones (PDI)	12
			3359	Agentes de la administración pública para la aplicación de la ley no clasificados previamente	33
			3411	Técnicos de los servicios jurídicos	36
			3412	Técnicos en trabajo social	46
			3413	Técnicos de los servicios religiosos	4
			3421	Atletas y deportistas	6
			3422	Entrenadores, instructores y árbitros de actividades deportivas	50
			3423	Instructores de acondicionamiento físico y actividades recreativas	25
			3431	Fotógrafos	12
			3432	Diseñadores y decoradores de interior	18
			3433	Técnicos en galerías de arte, museos y bibliotecas	13
			3434	Chefs	51
			3435	Tatuadores	22
			3439	Otros técnicos en actividades culturales y artísticas no clasificados previamente	2
			3511	Técnicos en operaciones de tecnología de la información y las comunicaciones	27

ID	Nombre variable	Etiqueta de variable	Valores	Etiquetas de valores	Frecuencia
			3512	Técnicos en asistencia al usuario de tecnología de la información y las comunicaciones	26
			3513	Técnicos en redes y sistemas de computadores	29
			3514	Técnicos de la web	5
			3521	Técnicos de radiodifusión y grabación audiovisual	35
			3522	Técnicos de ingeniería de las telecomunicaciones	20
			3611	Técnicos en educación parvularia	345
			3612	Técnicos en educación diferencial	40
			4110	Trabajadores de tareas administrativas generales	174
			4120	Secretarios generales	198
			4131	Operadores de equipo de procesamiento de texto y mecanógrafos	0
			4132	Digitadores de datos	35
			4211	Cajeros de bancos y de oficinas de correo	34
			4212	Receptores de apuestas	13
			4213	Empleados de casa de empeño y prestamistas	1
			4214	Cobradores	38
			4221	Empleados de agencias de viajes	15
			4222	Empleados de centros de llamadas de informaciones	38
			4223	Telefonistas	45
			4224	Recepcionistas de hoteles	33
			4225	Empleados de informaciones, reclamos o sugerencias	99
			4226	Recepcionistas (funciones generales)	73
			4227	Entrevistadores de investigaciones de mercado, estudios de opinión pública y encuestadores	14
			4229	Otros empleados de servicios de información al cliente no clasificados previamente	6
			4311	Auxiliares y ayudantes de registros de contabilidad y cálculo de costos	78
			4312	Auxiliares y ayudantes de servicios estadísticos, financieros y de seguros	11
			4313	Empleados encargados de las nóminas o registros de remuneraciones	20
			4321	Empleados encargados del control de abastecimiento e inventario	485
			4322	Empleados de cálculo de los insumos y materiales para la producción	0
			4323	Empleados de servicios de transporte	64
			4411	Asistentes y ayudantes de bibliotecas	10
			4412	Carteros y empleados de servicios de correos y encomiendas	38

ID	Nombre variable	Etiqueta de variable	Valores	Etiquetas de valores	Frecuencia
			4413	Codificadores de datos y correctores de pruebas de imprenta	0
			4414	Escribientes públicos	0
			4415	Empleados administrativos de archivos	32
			4416	Empleados y asistentes de recursos humanos	35
			4419	Otro personal de apoyo administrativo no clasificado previamente	119
			5111	Auxiliares de servicio a bordo de aeronaves y barcos	4
			5112	Revisores y cobradores de los transportes públicos	11
			5113	Guías de turismo	15
			5120	Cocineros	661
			5131	Garzones de mesa	207
			5132	Bármanes	24
			5141	Peluqueros	231
			5142	Cosmetólogos y especialistas en tratamientos de belleza	114
			5151	Supervisores de mantenimiento y limpieza en oficinas, hoteles y otros establecimientos	34
			5152	Amas de llaves, mayordomos domésticos y dueños/administradores de pequeños establecimientos de alojamiento	24
			5153	Conserjes	150
			5161	Astrólogos y adivinadores	1
			5162	Acompañantes	0
			5163	Personal de pompas fúnebres y embalsamadores	9
			5164	Cuidadores de animales	52
			5165	Instructores de manejo	4
			5169	Otros trabajadores de servicios a las personas no clasificados previamente	10
			5211	Vendedores en quioscos o puestos de feria	521
			5212	Vendedores ambulantes de productos comestibles de consumo inmediato (elaboración propia)	119
			5221	Comerciantes y dueños operadores de locales comerciales, tiendas, almacenes y mercados	861
			5222	Supervisores de locales comerciales, tiendas y almacenes	116
			5223	Vendedores y asistentes de venta de tiendas, almacenes y puestos de mercado	1.385
			5230	Vendedores de entradas (entretenciones y eventos deportivos) y cajeros de comercio	422
			5241	Modelos de moda, arte y publicidad	2
			5242	Promotores de tiendas	32
			5243	Representantes de ventas de puerta a puerta (venta a hogares)	87

ID	Nombre variable	Etiqueta de variable	Valores	Etiquetas de valores	Frecuencia
			5244	Vendedores por internet y otros medios de comunicación	140
			5245	Bomberos de gasolineras	53
			5246	Vendedores de comida al mostrador	75
			5249	Otros vendedores no clasificados previamente	137
			5311	Cuidadores de niños en instituciones y a domicilios	186
			5312	Asistentes de aula e inspectores de patio	288
			5321	Trabajadores de los cuidados personales en instituciones	61
			5322	Trabajadores de los cuidados personales a domicilio	144
			5329	Otros trabajadores de los cuidados personales en instituciones y a domicilio no clasificados previamente	11
			5411	Bomberos	15
			5412	Carabineros	76
			5413	Gendarmes	27
			5414	Guardias de seguridad	761
			5419	Otro personal de los servicios de protección no clasificados previamente	20
			6111	Agricultores y trabajadores calificados de cultivos extensivos	225
			6112	Agricultores y trabajadores calificados de plantaciones de árboles y arbustos	102
			6113	Agricultores y trabajadores calificados de huertas, invernaderos, viveros y jardines	239
			6114	Agricultores y trabajadores calificados de cultivos mixtos	72
			6121	Criadores de ganado	153
			6122	Avicultores y trabajadores calificados de la avicultura	18
			6123	Trabajadores calificados de la apicultura y la sericultura	10
			6129	Otros criadores y trabajadores calificados de la cría de animales no clasificados previamente	1
			6130	Productores y trabajadores calificados de explotaciones agropecuarias mixtas	42
			6210	Trabajadores forestales calificados	96
			6221	Trabajadores de explotaciones de acuicultura	42
			6222	Pescadores en agua dulce y en aguas costeras	129
			6223	Pescadores de alta mar	7
			6224	Cazadores y tramperos	0
			6310	Trabajadores agrícolas de subsistencia	13
			6320	Criadores de subsistencia	1
			6330	Trabajadores agropecuarios de subsistencia	6
			6340	Pescadores, cazadores, tramperos y recolectores de subsistencia	9

ID	Nombre variable	Etiqueta de variable	Valores	Etiquetas de valores	Frecuencia
			7111	Constructores de casas	198
			7112	Albañiles	303
			7113	Tronzadores, labrantes y grabadores de piedra	6
			7114	Operarios en cemento armado	50
			7115	Carpinteros de obra	558
			7119	Otros operarios de la construcción (obra gruesa) no clasificados previamente	195
			7121	Instaladores o reparadores de techos	15
			7122	Instaladores de parqué, cerámicas, baldosas y alfombras	47
			7123	Yeseros, estucadores y revocadores	25
			7124	Instaladores de material aislante y de insonorización	3
			7125	Cristaleros	27
			7126	Gásfiter e instaladores de tuberías	180
			7127	Mecánicos de instalaciones de refrigeración y aire acondicionado	37
			7131	Pintores y empapeladores de paredes	141
			7132	Barnizadores y pulverizadores de productos manufacturados	43
			7133	Limpiadores de fachadas y deshollinadores	3
			7211	Moldeadores y macheros	0
			7212	Soldadores y oxicortadores	254
			7213	Chapistas y caldereros	70
			7214	Montadores de estructuras metálicas	190
			7215	Aparejadores y empalmadores de cables no eléctricos	5
			7221	Herreros y forjadores	19
			7222	Herramentistas	11
			7223	Reguladores y operarios de máquinas herramientas	16
			7224	Pulidores de metales y afiladores de herramientas	2
			7231	Mecánicos y reparadores de vehículos de motor	478
			7232	Mecánicos y reparadores de motores de avión	5
			7233	Mecánicos y reparadores de máquinas agrícolas e industriales	189
			7234	Reparadores de bicicletas	16
			7311	Mecánicos y reparadores de instrumentos de precisión	5
			7312	Fabricantes y afinadores de instrumentos musicales	1
			7313	Joyeros, orfebres y plateros	26
			7314	Alfareros (barro, arcilla y abrasivos)	10
			7315	Sopladores, modeladores, laminadores, cortadores y pulidores de vidrio	3
			7316	Pintores de carteles, pintores decorativos y grabadores	8

ID	Nombre variable	Etiqueta de variable	Valores	Etiquetas de valores	Frecuencia
			7317	Artesanos en madera, cestería y materiales similares	33
			7318	Artesanos de los tejidos, el cuero y materiales similares	54
			7319	Artesanos no clasificados previamente	41
			7321	Tipógrafos	1
			7322	Impresores	52
			7323	Encuadernadores	16
			7411	Electricistas de obras	291
			7412	Mecánicos y ajustadores electricistas	87
			7413	Instaladores y reparadores de líneas eléctricas	40
			7421	Mecánicos y reparadores en electrónica	36
			7422	Instaladores y reparadores en tecnología de la información y las comunicaciones	93
			7511	Carniceros y pescaderos	163
			7512	Panaderos, pasteleros y confiteros	487
			7513	Operarios de la elaboración de productos lácteos	9
			7514	Operarios de la conservación de frutas, legumbres y verduras	15
			7515	Catadores, clasificadores y controladores de calidad de alimentos y bebidas	65
			7516	Elaboradores de tabaco y sus productos	0
			7521	Operarios del tratamiento de la madera	29
			7522	Ebanistas y mueblistas	174
			7523	Operadores y reguladores de máquinas para trabajar la madera	5
			7531	Sastres, modistos, peleteros y sombrereros	97
			7532	Patronistas y cortadores de telas	3
			7533	Costureros y bordadores	126
			7534	Tapiceros	20
			7535	Apelambradores, pellejeros y curtidores	2
			7536	Zapateros	16
			7541	Buzos	42
			7542	Dinamiteros y pegadores	10
			7543	Clasificadores, probadores de productos e inspectores de calidad (excluyendo alimentos y bebidas)	24
			7544	Fumigadores y otros controladores de plagas y malezas	29
			7549	Operarios de otros oficios no clasificados previamente	9
			8111	Mineros y operadores de instalaciones mineras	104
			8112	Operadores de instalaciones de procesamiento de minerales y rocas	24
			8113	Perforadores y sondistas de pozos	39

ID	Nombre variable	Etiqueta de variable	Valores	Etiquetas de valores	Frecuencia
			8114	Operadores de máquinas para fabricar cemento y otros productos minerales	10
			8121	Operadores de instalaciones de procesamiento de metales	13
			8122	Operadores de máquinas de acabado de metales (pulidores, galvanizadores y recubridores de metales)	8
			8131	Operadores de plantas y máquinas para fabricar productos químicos	23
			8132	Operadores de máquinas para fabricar productos fotográficos	1
			8141	Operadores de máquinas para fabricar productos de caucho	25
			8142	Operadores de máquinas para fabricar productos de material plástico	22
			8143	Operadores de máquinas para fabricar productos de papel	17
			8151	Operadores de máquinas de preparación de fibras, hilado y devanado	3
			8152	Operadores de telares y otras máquinas tejedoras	5
			8153	Operadores de máquinas de coser	21
			8154	Operadores de máquinas de blanqueamiento, teñido y limpieza de tejidos	13
			8155	Operadores de máquinas de tratamiento de pieles y cueros	1
			8156	Operadores de máquinas para la fabricación de calzado, bolsos y accesorios de talabartería	0
			8157	Operadores de máquinas de lavanderías	14
			8159	Operadores de máquinas para fabricar productos textiles y artículos de piel y cuero no clasificados previamente	13
			8160	Operadores de máquinas para elaborar alimentos, bebidas y cigarrillos	134
			8171	Operadores de instalaciones para la preparación de papel y de pasta para papel	2
			8172	Operadores de instalaciones de procesamiento de la madera	42
			8181	Operadores de instalaciones de vidriería y cerámica	7
			8182	Operadores de máquinas de vapor y calderas	13
			8183	Operadores de máquinas de embalaje, embotellamiento y etiquetado	69
			8189	Operadores de máquinas y de instalaciones fijas no clasificados previamente	6
			8211	Ensambladores de maquinaria mecánica	0

ID	Nombre variable	Etiqueta de variable	Valores	Etiquetas de valores	Frecuencia
			8212	Ensambladores de equipos eléctricos y electrónicos	6
			8219	Ensambladores no clasificados previamente	2
			8311	Maquinistas de locomotoras	7
			8312	Guardafrenos, guardagujas y agentes de maniobras	4
			8321	Conductores de motocicletas	29
			8322	Conductores de automóviles, taxis y camionetas	977
			8331	Conductores de buses y trolebuses	272
			8332	Conductores de camiones pesados y de alto tonelaje	663
			8341	Operadores de maquinaria agrícola y forestal móvil	119
			8342	Operadores de máquinas de movimiento de tierras	245
			8343	Operadores de grúas y aparatos elevadores	84
			8344	Operadores de autoelevadoras y montacargas	80
			8350	Tripulantes de cubierta de barco	39
			9111	Trabajadores de casa particular y asistentes domésticos	1.108
			9112	Auxiliares de aseo de oficinas, hoteles y otros establecimientos	1.236
			9121	Lavanderos y planchadores manuales	18
			9122	Limpiadores de vehículos	45
			9123	Limpiadores de ventanas	3
			9129	Otro personal de limpieza no clasificado previamente	16
			9211	Obreros de explotaciones agrícolas	903
			9212	Obreros de explotaciones ganaderas	34
			9213	Obreros de explotaciones agropecuarias	30
			9214	Ayudantes de jardinería y horticultura	79
			9215	Obreros forestales	63
			9216	Obreros de pesca y acuicultura	77
			9311	Obreros de la minería	19
			9312	Obreros de obras públicas	89
			9313	Obreros de la construcción de edificios	466
			9321	Empacadores manuales	44
			9329	Obreros de la industria manufacturera no clasificados previamente	41
			9331	Conductores de vehículos accionados a pedal o a brazo (no motorizados)	5
			9332	Conductores de vehículos y máquinas de tracción animal	4
			9333	Obreros de carga	231
			9334	Reponedores de estanterías	394
			9411	Cocineros de comida rápida	183
			9412	Ayudantes de cocina	156
			9510	Trabajadores ambulantes de servicios	16

ID	Nombre variable	Etiqueta de variable	Valores	Etiquetas de valores	Frecuencia
			9520	Vendedores ambulantes (excluyendo comida de consumo inmediato)	151
			9611	Recolectores de basura y material reciclable	69
			9612	Clasificadores de desechos	24
			9613	Barrenderos	77
			9621	Mensajeros, estafetas, maleteros y repartidores de folletos y diarios a domicilio	135
			9622	Auxiliares de mantenimiento (pequeñas reparaciones)	54
			9623	Recolectores de dinero en máquinas expendedoras de venta automática y lectores de medidores	20
			9624	Acarreadores de agua y recolectores de leña	22
			9629	Otras ocupaciones elementales no clasificadas previamente	33
43	oficio1_ciuo_08cl	Ocupación a 1 dígitos según clasificación CIUO 08.CL	-99	No responde	0
			-88	No bien especificado	270
			0	Ocupaciones de las Fuerzas Armadas	72
			1	Directores, gerentes y administradores	864
			2	Profesionales, científicos e intelectuales	4.849
			3	Técnicos y profesionales de nivel medio	3.909
			4	Personal de apoyo administrativo	1.708
			5	Trabajadores de los servicios y vendedores de comercios y mercados	7.090
			6	Agricultores y trabajadores calificados agropecuarios, forestales y pesqueros	1.165
			7	Artesanos y operarios de oficios	5.208
			8	Operadores de instalaciones, máquinas y ensambladores	3.156
			9	Ocupaciones elementales	5.845
44	o10	En su trabajo o negocio principal, ¿[NOM. SELEC] trabaja como?	1	Patrón o empleador	546
			2	Trabajador por cuenta propia	4.396
			3	Empleado u obrero del sector público (Gobierno Central o Municipal)	1.517
			4	Empleado u obrero de empresas públicas	832
			5	Empleado u obrero del sector privado	8.015
			6	Servicio doméstico puertas adentro	57
			7	Servicio doméstico puertas afuera	391
			8	FF.AA. y del Orden	63
			9	Familiar no remunerado	60
45	o11	¿Cuántas horas trabaja [NOM. SELEC] habitualmente por semana en su trabajo...?	Rango: 1 - 105		
46	o12	La empresa o neg. en que [NOM. SELEC] trabaja, ¿está registrada en el SII o...?	-88	No sabe	80
			1	Sí	1.813
			2	No	3.049
47	o13	La empresa, negocio o actividad por cuenta propia de [NOM.	-88	No sabe	167

ID	Nombre variable	Etiqueta de variable	Valores	Etiquetas de valores	Frecuencia
		SELEC]…?	1	Acude a los servicios de un contador para llevar la contabilidad completa	1.183
			2	Se encuentra acogida al régimen de contabilidad simplificada	171
			3	Solo cuenta con registros personales de gastos e ingresos	621
			4	No cuenta con ningún tipo de contabilidad	2.800
48	o14a	¿El empleador de [NOM. SELEC] cotiza por él(ella) en el sistema previsional?	-88	No sabe	36
			1	Sí	9.283
			2	No	1.556
49	o14b	¿El empleador de [NOM. SELEC] cotiza por él(ella) en el sistema de salud?	-88	No sabe	49
			1	Sí	9.515
			2	No	1.311
50	o15	¿Cotizó [NOM. SELEC] durante el mes pasado en algún sistema previsional…?	-88	No sabe	203
			1	Sí, AFP (Administradora de Fondos de Pensiones). Cotización obligatoria del trabajador dependiente	10.185
			2	Sí, AFP (Administradora de Fondos de Pensiones). Cotización voluntaria del trabajador independiente	584
			3	Sí, IPS ex INP [Caja Nacional de Empleados Públicos (CANAEMPU)], Caja de Empleados Particulares (EMPART), Servicio de Seguro Social (SSS) u otras	78
			4	Sí, Caja de Previsión de la Defensa Nacional (CAPREDENA)	88
			5	Sí, Dirección de Previsión de Carabineros (DIPRECA)	50
			6	Sí, otra. Especifique	16
			7	No está cotizando	17.001
			8	No está afiliado a un sistema previsional	2.980
51	o15_esp	Especifique. Cotizó en otro sistema previsional	Válidos		16
52	y1	Mes pasado, ingresos provenientes del trabajo, ocupación o actividad	-88	No sabe	4.655
			0	No recibió ingresos provenientes del trabajo	2.173
			Válidos		27.308
53	y2	Mes pasado, ingresos recibidos por jubilación o pensión	-88	No sabe	2.396
			0	No recibió ingresos por jubilación o pensión	66.712
			Válidos		14.639
54	y2_1a	Tipo jubilación o pensión: Pensión Garantizada Universal (PGU) o Ex PBS de Vejez	0	No	8.103
			1	Sí	4.416
55	y2_1b	Tipo jubilación o pensión: Jubilación o Pensión de Vejez con PGU o APS	0	No	11.454
			1	Sí	1.065
56	y2_1c	Tipo jubilación o pensión: Jubilación o Pensión de Vejez	0	No	8.852
			1	Sí	5.088
57	y2_1d	Tipo jubilación o pensión: Pensión Básica Solidaria de Invalidez	0	No	2.523
			1	Sí	714
58	y2_1e	Tipo jubilación o pensión: Jubilación o Pensión de Invalidez con APS	0	No	3.104
			1	Sí	133
59	y2_1f	Tipo jubilación o pensión: Jubilación o Pensión de Invalidez	0	No	13.245
			1	Sí	1.259
60	y2_1g	Tipo jubilación o pensión: Montepío o Pensión de Viudez	0	No	13.440
			1	Sí	1.199
61	y2_1h	Tipo jubilación o pensión: Pensión de Orfandad	0	No	14.491
			1	Sí	148
62	y2_1i	Tipo jubilación o pensión: Pensión por Leyes Especiales	0	No	14.405

ID	Nombre variable	Etiqueta de variable	Valores	Etiquetas de valores	Frecuencia
			1	Sí	234
63	y2_1j	Tipo jubilación o pensión: Otro	0	No	14.003
			1	Sí	636
64	y2_2	Monto de Jubilación o Pensión de Vejez o Invalidez sin PGU o APS	-88	No sabe	243
			Válidos		955
65	y2_3	Monto de la Pensión Garantizada Universal o Aporte Previsional Solidario	-88	No sabe	290
			Válidos		908
66	y3	Mes pasado, ingresos por subsidios, bonos o aportes mensuales del Estado	-88	No sabe	2.580
			0	No recibió ingresos	74.993
			Válidos		6.174
67	y4	Últimos 12 meses, ingresos por subsidios, bonos o aportes del Estado sin IFE	-88	No sabe	2.763
			0	No recibió ingresos	74.643
			Válidos		6.341
68	y5_hog	Incluyéndose a Ud. ¿alguien en su hogar recibió IFE Universal? (jun-nov 2021)	-88	No sabe	1.364
			1	Sí	71.971
			2	No	10.412
69	y5_preg	Recibió IFE Universal	0	No	33.800
			1	Sí	28.522
70	y5_mes	¿Desde qué mes fue beneficiario del IFE Universal [NOMBRE]?	-88	No sabe	1.377
			1	Junio 2021	18.942
			2	Julio 2021	1.332
			3	Agosto 2021	2.304
			4	Septiembre 2021	3.158
			5	Octubre 2021	893
			6	Noviembre 2021	516
71	y5_integrantes	Incluyendo a [NOMBRE], ¿por cuántas personas le pagaron el IFE Universal?	-88	No sabe	136
			1	1 persona	10.429
			2	2 personas	7.652
			3	3 personas	5.454
			4	4 personas	3.308
			5	5 personas	1.065
			6	6 personas	327
			7	7 personas	89
			8	8 personas	31
			9	9 personas	27
			10	10 o más personas	4
72	y5_monto	¿Puede indicarme cuál es el monto que recibió [NOMBRE] por IFE Universal?	-88	No sabe	120
			Válidos		16
73	y6	Mes pasado, ingreso por arriendo urbano, pensión de alimentos o aporte familiares	-88	No sabe	1.115
			0	No recibió ingresos	79.336
			Válidos		3.296
74	y7	Mes pasado, pensando en el ingreso mensual total del hogar, ¿usted diría que….?	-88	No sabe/No responde	285
			1	No les alcanzó, tuvieron muchas dificultades	15.471
			2	No les alcanzó, tuvieron algunas dificultades	25.111
			3	Les alcanzó justo, sin mayores dificultades	32.117
			4	Les alcanzó bien, no tuvieron dificultades	10.763
75	y8_a	¿El ingreso del hogar le permite pagar los servicios básicos de luz, agua, gas?	-88	No sabe/No responde	228
			1	Sí	69.559
			2	No	13.960
76	y8_b	¿El ingreso del hogar le permite pagar los alimentos para comer todo el mes?	-88	No sabe/No responde	165
			1	Sí	68.521
			2	No	15.061
77	y8_c	¿El ingreso del hogar le permite pagar los gastos en salud?	-88	No sabe/No responde	1.510
			1	Sí	46.592
			2	No	35.645
78	v1	Su hogar, ¿bajo qué situación ocupa la vivienda?	1	Propia pagada	48.858
			2	Propia pagándose	7.184
			3	Propia compartida (pagada) con otros hogares de la vivienda	754

ID	Nombre variable	Etiqueta de variable	Valores	Etiquetas de valores	Frecuencia
			4	Propia compartida (pagándose) con otros hogares de la vivienda	103
			5	Arrendada con contrato	9.196
			6	Arrendada sin contrato	4.758
			7	Cedida por servicio o trabajo	1.095
			8	Cedida por familiar u otro	9.282
			9	Usufructo (sólo uso y goce)	1.859
			10	Ocupación irregular (de hecho)	568
			11	Poseedor irregular	90
79	v2	¿Cuántos dormitorios de uso exclusivo para dormir ocupa su hogar?	-88	No sabe	142
80	informante	Identificación del informante del cuestionario hogar	0	No informante	40.086
			1	Informante	25.602
81	forma_ent_hogar_fin	Forma de finalización del cuestionario hogar	1	Respondió la encuesta por sí mismo(a)	24.141
			2	Respondió la encuesta con ayuda de otra persona	1.461
			3	Respondió la encuesta con ayuda del sistema Vi-Sor	0
82	forma_ent_adulto_inicio	Forma de inicio del cuestionario adulto	1	Responderá la encuesta por sí mismo(a)	28.307
			2	Responderá la encuesta con ayuda de otra persona	1.238
			3	Responderá la encuesta con ayuda del sistema Vi-Sor	0
			4	Otra persona responderá la encuesta en su nombre	465
83	m1_intento1_1	Memoria. Primer intento para repetir la palabra: Rojo	0	No	301
			1	Sí	7.723
84	m1_intento1_2	Memoria. Primer intento para repetir la palabra: Cara	0	No	1.677
			1	Sí	6.347
85	m1_intento1_3	Memoria. Primer intento para repetir la palabra: Seda	0	No	3.657
			1	Sí	4.367
86	m1_intento1_4	Memoria. Primer intento para repetir la palabra: Iglesia	0	No	2.757
			1	Sí	5.267
87	m1_intento1_5	Memoria. Primer intento para repetir la palabra: Clavel	0	No	2.477
			1	Sí	5.547
88	m1_intento1_78	Memoria. No repite ninguna palabra en el primer intento	0	No	7.948
			1	Sí	76
89	m1_intento2_1	Memoria. Segundo intento para repetir la palabra: Rojo	0	No	353
			1	Sí	7.671
90	m1_intento2_2	Memoria. Segundo intento para repetir la palabra: Cara	0	No	933
			1	Sí	7.091
91	m1_intento2_3	Memoria. Segundo intento para repetir la palabra: Seda	0	No	2.099
			1	Sí	5.925
92	m1_intento2_4	Memoria. Segundo intento para repetir la palabra: Iglesia	0	No	1.502
			1	Sí	6.522
93	m1_intento2_5	Memoria. Segundo intento para repetir la palabra: Clavel	0	No	1.510
			1	Sí	6.514
94	m1_intento2_78	Memoria. No repite ninguna palabra en el segundo intento	0	No	7.953
			1	Sí	71
95	m2	Fluidez Verbal: Número de palabras que comienzan con la letra P	Rango: 0 - 56		
96	m3_1	Orientación: ¿En qué día del mes estamos?	1	Responde correctamente	7.093
			2	Responde incorrectamente	931
97	m3_2	Orientación: ¿En qué mes estamos?	1	Responde correctamente	7.756
			2	Responde incorrectamente	268
98	m3_3	Orientación: ¿En qué año estamos?	1	Responde correctamente	7.374
			2	Responde incorrectamente	650
99	m3_4	Orientación: ¿En qué día de la semana estamos?	1	Responde correctamente	7.714
			2	Responde incorrectamente	310
100	m3_5	Orientación: ¿En qué lugar estamos?	1	Responde correctamente	7.846
			2	Responde incorrectamente	178

ID	*Nombre variable*	*Etiqueta de variable*	*Valores*	*Etiquetas de valores*	*Frecuencia*
101	m3_6	Orientación: ¿En qué comuna estamos?	1	Responde correctamente	7.900
			2	Responde incorrectamente	124
102	m4_libre_1	Recuerdo diferido. Repite la palabra en modalidad libre: Rojo	0	No	1.734
			1	Sí	6.290
103	m4_libre_2	Recuerdo diferido. Repite la palabra en modalidad libre: Cara	0	No	2.634
			1	Sí	5.390
104	m4_libre_3	Recuerdo diferido. Repite la palabra en modalidad libre: Seda	0	No	3.198
			1	Sí	4.826
105	m4_libre_4	Recuerdo diferido. Repite la palabra en modalidad libre: Iglesia	0	No	2.910
			1	Sí	5.114
106	m4_libre_5	Recuerdo diferido. Repite la palabra en modalidad libre: Clavel	0	No	3.821
			1	Sí	4.203
107	m4_libre_78	Recuerdo diferido. No repite ninguna palabra en modalidad libre	0	No	7.395
			1	Sí	629
108	m4_clave_sem_1	Recuerdo diferido. Repite la palabra en clave semántica: Rojo	0	No	526
			1	Sí	1.208
109	m4_clave_sem_2	Recuerdo diferido. Repite la palabra en clave semántica: Cara	0	No	1.581
			1	Sí	1.053
110	m4_clave_sem_3	Recuerdo diferido. Repite la palabra en clave semántica: Seda	0	No	1.711
			1	Sí	1.487
111	m4_clave_sem_4	Recuerdo diferido. Repite la palabra en clave semántica: Iglesia	0	No	1.739
			1	Sí	1.171
112	m4_clave_sem_5	Recuerdo diferido. Repite la palabra en clave semántica: Clavel	0	No	2.155
			1	Sí	1.666
113	m4_clave_sem_78	Recuerdo diferido. No repite ninguna palabra en clave semántica	0	No	4.159
			1	Sí	1.714
114	m4_clave_sem_2_1	Recuerdo diferido. Repite la palabra con listado de respuestas: Rojo	0	No	2.661
			1	Sí	273
115	m4_clave_sem_2_2	Recuerdo diferido. Repite la palabra con listado de respuestas: Cara	0	No	2.497
			1	Sí	760
116	m4_clave_sem_2_3	Recuerdo diferido. Repite la palabra con listado de respuestas: Seda	0	No	2.344
			1	Sí	783
117	m4_clave_sem_2_4	Recuerdo diferido. Repite la palabra con listado de respuestas: Iglesia	0	No	2.263
			1	Sí	1.009
118	m4_clave_sem_2_5	Recuerdo diferido. Repite la palabra con listado de respuestas: Clavel	0	No	2.206
			1	Sí	991
119	m4_clave_sem_2_78	Recuerdo diferido. No repite ninguna palabra con listado de respuestas	0	No	2.559
			1	Sí	1.200
120	d1	¿Qué tan problemático ha sido…? Caminar 10 cuadras o un kilómetro	-99	No responde	14
			-88	No sabe	29
			1	Nada problemático	19.952
			2	Levemente problemático	3.488
			3	Moderadamente problemático	3.161
			4	Severamente problemático	1.837
			5	Extremadamente problemático o no puede hacerlo	1.529
121	d2	¿Qué tan problemático ha sido…? Llegar a los lugares donde ha querido ir	-99	No responde	9
			-88	No sabe	17
			1	Nada problemático	21.604
			2	Levemente problemático	3.216
			3	Moderadamente problemático	2.566
			4	Severamente problemático	1.473
			5	Extremadamente problemático o no puede hacerlo	1.125
122	d3	¿Qué tan problemático ha sido…? Asearse o vestirse	-99	No responde	1
			-88	No sabe	1
			1	Nada problemático	26.880
			2	Levemente problemático	1.450
			3	Moderadamente problemático	855
			4	Severamente problemático	445
			5	Extremadamente problemático o no puede hacerlo	378
123	d4	¿Qué tan problemático ha sido…? Usar el baño (W.C.)	-99	No responde	5

ID	*Nombre variable*	*Etiqueta de variable*	*Valores*	*Etiquetas de valores*	*Frecuencia*
			-88	No sabe	2
			1	Nada problemático	28.124
			2	Levemente problemático	828
			3	Moderadamente problemático	496
			4	Severamente problemático	264
			5	Extremadamente problemático o no puede hacerlo	291
124	d5	¿Qué tan problemático ha sido…? Cuidar de su salud	-99	No responde	9
			-88	No sabe	18
			1	Nada problemático	24.330
			2	Levemente problemático	2.980
			3	Moderadamente problemático	1.628
			4	Severamente problemático	592
			5	Extremadamente problemático o no puede hacerlo	453
125	d6	¿Qué tan problemático ha sido…? Sentirse cansado y no tener suficiente energía	-99	No responde	8
			-88	No sabe	7
			1	Nada problemático	15.598
			2	Levemente problemático	7.343
			3	Moderadamente problemático	4.521
			4	Severamente problemático	1.887
			5	Extremadamente problemático o no puede hacerlo	646
126	d7	¿Qué tan problemático ha sido…? Enfrentar todas las tareas que tiene que hacer	-99	No responde	8
			-88	No sabe	5
			1	Nada problemático	18.513
			2	Levemente problemático	5.438
			3	Moderadamente problemático	3.716
			4	Severamente problemático	1.488
			5	Extremadamente problemático o no puede hacerlo	842
127	d8	¿Qué tan problemático ha sido…? Recordar cosas importantes que tiene que hacer	-99	No responde	8
			-88	No sabe	2
			1	Nada problemático	20.193
			2	Levemente problemático	5.918
			3	Moderadamente problemático	2.567
			4	Severamente problemático	852
			5	Extremadamente problemático o no puede hacerlo	470
128	d9	¿Qué tan problemático ha sido…? Hacer las tareas de la casa	-99	No responde	30
			-88	No sabe	72
			1	Nada problemático	23.268
			2	Levemente problemático	3.139
			3	Moderadamente problemático	1.915
			4	Severamente problemático	779
			5	Extremadamente problemático o no puede hacerlo	807
129	d10	¿Qué tan problemático ha sido…? Participar en fiestas, eventos	-99	No responde	608
			-88	No sabe	1.493
			1	Nada problemático	22.729
			2	Levemente problemático	2.160
			3	Moderadamente problemático	1.309
			4	Severamente problemático	708
			5	Extremadamente problemático o no puede hacerlo	1.003
130	d11	¿Qué tan problemático ha sido…? Utilizar los servicios de transporte público	-99	No responde	179
			-88	No sabe	773
			1	Nada problemático	22.412
			2	Levemente problemático	2.401
			3	Moderadamente problemático	1.825
			4	Severamente problemático	1.127
			5	Extremadamente problemático o no puede hacerlo	1.293

ID	*Nombre variable*	*Etiqueta de variable*	*Valores*	*Etiquetas de valores*	*Frecuencia*
131	d12	¿Qué tan problemático ha sido…? Realizar las tareas del trabajo o estudios	-99	No responde	82
			-88	No sabe	47
			1	Nada problemático	15.251
			2	Levemente problemático	1.523
			3	Moderadamente problemático	719
			4	Severamente problemático	247
			5	Extremadamente problemático o no puede hacerlo	121
132	d13	En los últimos 12 meses, ¿producto del COVID-19 estas dificultades…?	1	Han disminuido mucho	771
			2	Han disminuido poco	1.873
			3	Se han mantenido igual	10.544
			4	Han aumentado poco	4.170
			5	Han aumentado mucho	2.170
133	c1	Incluyendo su salud física y mental, ¿cómo calificaría su estado de salud hoy?	1	Muy mala	595
			2	Mala	2.188
			3	Regular	11.483
			4	Buena	13.104
			5	Muy buena	2.640
134	c2	¿Qué grado de dificultad ha tenido para? Ver, sin usar anteojos ópticos o lentes	-99	No responde	4
			-88	No sabe	11
			1	Ninguna	11.162
			2	Leve	7.525
			3	Moderada	6.468
			4	Severa	3.550
			5	Extrema o no puede hacerlo	1.290
135	c3	¿Qué grado de dificultad ha tenido para...? Oír, sin usar audífono o dispositivo	-99	No responde	9
			-88	No sabe	9
			1	Ninguna	25.512
			2	Leve	2.092
			3	Moderada	1.405
			4	Severa	719
			5	Extrema o no puede hacerlo	264
136	c4	¿Qué grado de dificultad ha tenido para...? Caminar o subir peldaños	-99	No responde	2
			-88	No sabe	3
			1	Ninguna	21.012
			2	Leve	3.393
			3	Moderada	2.745
			4	Severa	1.690
			5	Extrema o no puede hacerlo	1.165
137	c5	¿Qué grado de dificultad ha tenido para...? Recordar cosas o concentrarse	-99	No responde	2
			-88	No sabe	3
			1	Ninguna	18.909
			2	Leve	6.566
			3	Moderada	3.162
			4	Severa	929
			5	Extrema o no puede hacerlo	439
138	c6	¿Qué grado de dificultad ha tenido para...? Asearse o vestirse	-99	No responde	1
			-88	No sabe	0
			1	Ninguna	27.060
			2	Leve	1.259
			3	Moderada	874
			4	Severa	383
			5	Extrema o no puede hacerlo	433
139	c7	¿Qué grado de dificultad ha tenido para...? Alimentarse	-99	No responde	4
			-88	No sabe	2
			1	Ninguna	28.091
			2	Leve	937
			3	Moderada	582
			4	Severa	198
			5	Extrema o no puede hacerlo	196
140	c8	¿Qué grado de dificultad ha tenido para...? Usar el baño (W.C.)	-99	No responde	3
			-88	No sabe	4
			1	Ninguna	28.192

ID	*Nombre variable*	*Etiqueta de variable*	*Valores*	*Etiquetas de valores*	*Frecuencia*
			2	Leve	725
			3	Moderada	484
			4	Severa	263
			5	Extrema o no puede hacerlo	339
141	c9	¿Qué grado de dificultad ha tenido para...? Moverse o desplazarse en la casa	-99	No responde	3
			-88	No sabe	1
			1	Ninguna	27.092
			2	Leve	1.306
			3	Moderada	889
			4	Severa	372
			5	Extrema o no puede hacerlo	347
142	c10	¿Qué grado de dificultad ha tenido para...? Acostarse o levantarse de la cama	-99	No responde	4
			-88	No sabe	2
			1	Ninguna	25.737
			2	Leve	2.125
			3	Moderada	1.223
			4	Severa	561
			5	Extrema o no puede hacerlo	358
143	c11	¿Qué grado de dificultad ha tenido para...? Dormir	-99	No responde	3
			-88	No sabe	4
			1	Ninguna	20.044
			2	Leve	4.354
			3	Moderada	3.378
			4	Severa	1.677
			5	Extrema o no puede hacerlo	550
144	c12	¿Qué grado de dificultad ha tenido para…? Hacer las tareas de la casa	-99	No responde	23
			-88	No sabe	69
			1	Ninguna	24.041
			2	Leve	2.579
			3	Moderada	1.727
			4	Severa	702
			5	Extrema o no puede hacerlo	869
145	c13	¿Qué grado de dificultad ha tenido para…? Salir a la calle	-99	No responde	10
			-88	No sabe	29
			1	Ninguna	24.605
			2	Leve	1.797
			3	Moderada	1.542
			4	Severa	962
			5	Extrema o no puede hacerlo	1.065
146	c14	¿Qué grado de dificultad ha tenido para…? Hacer compras o ir al médico	-99	No responde	12
			-88	No sabe	22
			1	Ninguna	23.935
			2	Leve	1.980
			3	Moderada	1.741
			4	Severa	1.070
			5	Extrema o no puede hacerlo	1.250
147	c15	¿Qué grado de dificultad ha tenido para…? Participar en fiestas, eventos	-99	No responde	553
			-88	No sabe	1.407
			1	Ninguna	23.460
			2	Leve	1.610
			3	Moderada	1.196
			4	Severa	665
			5	Extrema o no puede hacerlo	1.119
148	c16	¿Qué grado de dificultad ha tenido para…? Llevarse bien con la gente cercana	-99	No responde	13
			-88	No sabe	18
			1	Ninguna	27.126
			2	Leve	1.581
			3	Moderada	882
			4	Severa	211
			5	Extrema o no puede hacerlo	179
149	c17	¿Qué grado de dificultad ha tenido para…? Cuidar o dar apoyo a otros	-99	No responde	32
			-88	No sabe	56
			1	Ninguna	25.785

ID	*Nombre variable*	*Etiqueta de variable*	*Valores*	*Etiquetas de valores*	*Frecuencia*
			2	Leve	1.670
			3	Moderada	1.068
			4	Severa	471
			5	Extrema o no puede hacerlo	928
150	c18	¿Qué grado de dificultad ha tenido para…? Manejar sus medicamentos	-99	No responde	158
			-88	No sabe	305
			1	Ninguna	25.866
			2	Leve	1.632
			3	Moderada	990
			4	Severa	441
			5	Extrema o no puede hacerlo	618
151	c19	¿Qué grado de dificultad ha tenido para…? Manejar o administrar dinero	-99	No responde	16
			-88	No sabe	23
			1	Ninguna	27.281
			2	Leve	1.106
			3	Moderada	707
			4	Severa	274
			5	Extrema o no puede hacerlo	603
152	c20	¿Qué grado de dificultad ha tenido para…? Hacer o recibir llamadas o utilizar…	-99	No responde	16
			-88	No sabe	46
			1	Ninguna	27.084
			2	Leve	1.122
			3	Moderada	782
			4	Severa	364
			5	Extrema o no puede hacerlo	596
153	c21	¿Qué grado de dificultad ha tenido para…? Mantener el equilibrio físico	-99	No responde	5
			-88	No sabe	6
			1	Ninguna	23.680
			2	Leve	2.913
			3	Moderada	1.914
			4	Severa	832
			5	Extrema o no puede hacerlo	660
154	c22	¿Qué grado de dificultad le ha generado? Sentirse triste, deprimido, preocupado	-99	No responde	15
			-88	No sabe	47
			1	Ninguna	12.948
			2	Leve	7.643
			3	Moderada	6.130
			4	Severa	2.292
			5	Extrema	935
155	c23	¿Qué grado de dificultad le ha generado? Sentir algún malestar o dolor físico	-99	No responde	9
			-88	No sabe	29
			1	Ninguna	11.263
			2	Leve	8.310
			3	Moderada	6.689
			4	Severa	2.735
			5	Extrema	975
156	c24	¿Qué grado de dificultad le ha generado? La pérdida de fuerza en las manos	-99	No responde	7
			-88	No sabe	56
			1	Ninguna	20.326
			2	Leve	4.346
			3	Moderada	3.181
			4	Severa	1.496
			5	Extrema	598
157	c25	¿Ha perdido mucho peso recientemente sin que usted quisiera?	1	Sí	5.680
			2	No	24.330
158	c26_1	Enfermedad o condición de salud: Ceguera	0	No	29.729
			1	Sí	281
159	c26_2	Enfermedad o condición de salud: Pérdida de la visión	0	No	20.866
			1	Sí	9.144
160	c26_3	Enfermedad o condición de salud: Sordera	0	No	29.520
			1	Sí	490
161	c26_4	Enfermedad o condición de salud: Pérdida de la audición	0	No	27.497
			1	Sí	2.513

ID	*Nombre variable*	*Etiqueta de variable*	*Valores*	*Etiquetas de valores*	*Frecuencia*
162	c26_5	Enfermedad o condición de salud: Sordoceguera	0	No	29.964
			1	Sí	46
163	c26_6	Enfermedad o condición de salud: Presión arterial alta	0	No	21.997
			1	Sí	8.013
164	c26_7	Enfermedad o condición de salud: Migraña	0	No	25.640
			1	Sí	4.370
165	c26_8	Enfermedad o condición de salud: COVID-19	0	No	25.005
			1	Sí	5.005
166	c26_9	Enfermedad o condición de salud: COVID prolongado	0	No	29.634
			1	Sí	376
167	c26_10	Enfermedad o condición de salud: Diabetes	0	No	25.835
			1	Sí	4.175
168	c26_11	Enfermedad o condición de salud: Malnutrición por exceso o déficit	0	No	28.127
			1	Sí	1.883
169	c26_12	Enfermedad o condición de salud: Depresión o trastorno depresivo	0	No	26.501
			1	Sí	3.509
170	c26_13	Enfermedad o condición de salud: Ansiedad o trastornos de ansiedad	0	No	26.480
			1	Sí	3.530
171	c26_14	Enfermedad o condición de salud: Trastorno por déficit de atención	0	No	29.222
			1	Sí	788
172	c26_15	Enfermedad o condición de salud: Problema o trastorno del sueño-vigilia	0	No	25.306
			1	Sí	4.704
173	c26_16	Enfermedad o condición de salud: Trastorno alimentario	0	No	29.784
			1	Sí	226
174	c26_17	Enfermedad o condición de salud: Enfermedad al corazón	0	No	28.529
			1	Sí	1.481
175	c26_18	Enfermedad o condición de salud: Accidente o ataque cerebrovascular	0	No	29.601
			1	Sí	409
176	c26_19	Enfermedad o condición de salud: Enfermedad tiroidea	0	No	27.283
			1	Sí	2.727
177	c26_20	Enfermedad o condición de salud: Asma o enfermedad alérgica respiratoria	0	No	28.204
			1	Sí	1.806
178	c26_21	Enfermedad o condición de salud: Enfermedad respiratoria crónica	0	No	29.218
			1	Sí	792
179	c26_22	Enfermedad o condición de salud: Enfermedades de la piel	0	No	28.942
			1	Sí	1.068
180	c26_23	Enfermedad o condición de salud: Enfermedad renal crónica	0	No	29.346
			1	Sí	664
181	c26_24	Enfermedad o condición de salud: Tumor o cáncer	0	No	29.253
			1	Sí	757
182	c26_25	Enfermedad o condición de salud: Enfermedades reumatológicas	0	No	28.596
			1	Sí	1.414
183	c26_26	Enfermedad o condición de salud: Incontinencia urinaria	0	No	29.119
			1	Sí	891
184	c26_27	Enfermedad o condición de salud: Úlcera venosa crónica o del pie diabético	0	No	29.819
			1	Sí	191
185	c26_28	Enfermedad o condición de salud: Demencia	0	No	29.729
			1	Sí	281
186	c26_29	Enfermedad o condición de salud: Parkinson o trastornos del movimiento	0	No	29.851
			1	Sí	159
187	c26_30	Enfermedad o condición de salud: Dolor crónico de duración mayor a tres meses	0	No	25.571
			1	Sí	4.439
188	c26_31	Enfermedad o condición de salud: Amputación/ausencia/malformación extremidades	0	No	29.891
			1	Sí	119
189	c26_32	Enfermedad o condición de salud: Epilepsia	0	No	29.776
			1	Sí	234
190	c26_33	Enfermedad o condición de salud: Autismo (Trastorno del espectro autista)	0	No	29.938
			1	Sí	72
191	c26_34	Enfermedad o condición de salud: Bipolaridad	0	No	29.799
			1	Sí	211
192	c26_35	Enfermedad o condición de salud: Esquizofrenia u otros trastornos psicóticos	0	No	29.929
			1	Sí	81
193	c26_36	Enfermedad o condición de salud: Problema o trastorno por	0	No	29.873

ID	*Nombre variable*	*Etiqueta de variable*	*Valores*	*Etiquetas de valores*	*Frecuencia*
		consumo de alcohol	1	Sí	137
194	c26_37	Enfermedad o condición de salud: Problema o trastorno por consumo de drogas	0	No	29.912
			1	Sí	98
195	c26_38	Enfermedad o condición de salud: Lesión medular o disfaria espinal	0	No	29.953
			1	Sí	57
196	c26_39	Enfermedad o condición de salud: Enfermedad neuromuscular	0	No	29.937
			1	Sí	73
197	c26_40	Enfermedad o condición de salud: Parálisis cerebral	0	No	29.976
			1	Sí	34
198	c26_41	Enfermedad o condición de salud: Síndrome de Down	0	No	29.971
			1	Sí	39
199	c26_42	Enfermedad o condición de salud: SIDA/VIH o enfermedades de transmisión sexual	0	No	29.969
			1	Sí	41
200	c26_43	Enfermedad o condición de salud: Lesiones/secuelas graves accidentes de tránsito	0	No	29.684
			1	Sí	326
201	c26_44	Enfermedad o condición de salud: Lesiones/secuelas graves caída/quemadura/golpes	0	No	29.420
			1	Sí	590
202	c26_45	Enfermedad o condición de salud: Lesiones/secuelas graves por otras personas	0	No	29.926
			1	Sí	84
203	c26_77	Enfermedad o condición de salud: Otro problema de salud crónico	0	No	28.170
			1	Sí	1.840
204	c26_78	Enfermedad o condición de salud: No tiene ninguna enfermedad o condición	0	No	22.595
			1	Sí	7.415
205	c26_77_esp	Enfermedad o condición de salud: Especifique. Otro problema de salud crónico	Válidos		1.840
206	c27	¿Toma medicamentos de manera habitual debido a esta(s) enfermedad(es)?	1	Sí	14.903
			2	No	7.692
207	c28_1	Condición permanente y/o larga duración: Dificultad física y/o movilidad	0	No	26.650
			1	Sí	3.360
208	c28_2	Condición permanente y/o larga duración: Mudez o dificultad en el habla	0	No	29.718
			1	Sí	292
209	c28_3	Condición permanente y/o larga duración: Dificultad psiquiátrica	0	No	29.452
			1	Sí	558
210	c28_4	Condición permanente y/o larga duración: Dificultad mental o intelectual	0	No	29.444
			1	Sí	566
211	c28_5	Condición permanente y/o larga duración: Dificultad psicosocial	0	No	29.656
			1	Sí	354
212	c28_6	Condición permanente y/o larga duración: Sordera o dificultad para oír	0	No	29.322
			1	Sí	688
213	c28_7	Condición permanente y/o larga duración: Ceguera o dificultad para ver	0	No	28.748
			1	Sí	1.262
214	c28_8	Condición permanente y/o larga duración: No tiene ninguna de estas condiciones	0	No	5.326
			1	Sí	24.684
215	fa1	¿En qué medida le hacen fácil o difícil…? Espacios públicos	-99	No responde	126
			-88	No sabe	600
			1	Muy fácil	7.136
			2	Fácil	13.797
			3	Ni fácil ni difícil	4.802
			4	Difícil	2.584
			5	Muy difícil	965
216	fa2	¿En qué medida le hacen fácil o difícil…? Tiendas del vecindario	-99	No responde	50
			-88	No sabe	118
			1	Muy fácil	7.105
			2	Fácil	14.741
			3	Ni fácil ni difícil	4.027
			4	Difícil	2.939
			5	Muy difícil	1.030
217	fa3	¿En qué medida le hacen fácil o difícil…? Transporte público utilizarlo	-99	No responde	165
			-88	No sabe	732
			1	Muy fácil	6.572
			2	Fácil	13.572
			3	Ni fácil ni difícil	3.747
			4	Difícil	3.529

ID	*Nombre variable*	*Etiqueta de variable*	*Valores*	*Etiquetas de valores*	*Frecuencia*
			5	Muy difícil	1.693
218	fa4	¿En qué medida le hacen fácil o difícil…? Su vivienda, baño y habitaciones vivir	-99	No responde	6
			-88	No sabe	8
			1	Muy fácil	10.233
			2	Fácil	16.342
			3	Ni fácil ni difícil	2.028
			4	Difícil	1.066
			5	Muy difícil	327
219	fa5	¿En qué medida le hacen fácil o difícil…? Servicios de salud utilizarlos	-99	No responde	30
			-88	No sabe	124
			1	Muy fácil	6.658
			2	Fácil	14.476
			3	Ni fácil ni difícil	3.987
			4	Difícil	3.590
			5	Muy difícil	1.145
220	fa6	¿En qué medida le hacen fácil o difícil…? Entorno donde vive hacer actividades	-99	No responde	15
			-88	No sabe	20
			1	Muy fácil	7.115
			2	Fácil	16.431
			3	Ni fácil ni difícil	3.901
			4	Difícil	2.053
			5	Muy difícil	475
221	fa7	¿En qué medida le hacen fácil o difícil…? Su lugar de trabajo hacer lo que debe	-99	No responde	45
			-88	No sabe	30
			1	Muy fácil	4.293
			2	Fácil	8.944
			3	Ni fácil ni difícil	1.727
			4	Difícil	710
			5	Muy difícil	102
222	fa8	¿En qué medida le hacen fácil o difícil…? Est. educacional hacer lo que debe	-99	No responde	55
			-88	No sabe	19
			1	Muy fácil	1.015
			2	Fácil	1.605
			3	Ni fácil ni difícil	394
			4	Difícil	118
			5	Muy difícil	19
223	fa9	¿En qué medida le resultaría fácil o difícil…? Ayuda de pariente o familiar	-99	No responde	13
			-88	No sabe	82
			1	Muy fácil	7.211
			2	Fácil	15.656
			3	Ni fácil ni difícil	2.846
			4	Difícil	3.336
			5	Muy difícil	866
224	fa10	¿En qué medida le resultaría fácil o difícil…? Ayuda de amigos o compañeros	-99	No responde	232
			-88	No sabe	529
			1	Muy fácil	4.398
			2	Fácil	13.558
			3	Ni fácil ni difícil	4.586
			4	Difícil	5.164
			5	Muy difícil	1.543
225	fa11	¿En qué medida le resultaría fácil o difícil…? Ayuda de vecinos o conocidos	-99	No responde	40
			-88	No sabe	321
			1	Muy fácil	3.693
			2	Fácil	13.427
			3	Ni fácil ni difícil	5.131
			4	Difícil	5.577
			5	Muy difícil	1.821
226	fa12	¿Con qué frecuencia…? Puede tomar sus propias decisiones en su vida diaria	1	Siempre	23.991
			2	Muchas veces	3.000
			3	Algunas veces	2.139
			4	Casi nunca	484
			5	Nunca	396
227	fa13	¿Con qué frecuencia…? Siente que las demás personas lo	1	Siempre	21.731

ID	Nombre variable	Etiqueta de variable	Valores	Etiquetas de valores	Frecuencia
		respetan o valoran	2	Muchas veces	4.778
			3	Algunas veces	3.008
			4	Casi nunca	344
			5	Nunca	149
228	fa14	Grado de acuerdo: En general su familia lo invita a actividades sociales	1	Muy en desacuerdo	1.301
			2	En desacuerdo	2.479
			3	De acuerdo	15.647
			4	Muy de acuerdo	10.583
229	fa15	Grado de acuerdo: Cuenta con un buen grupo de amistades y conocidos	1	Muy en desacuerdo	1.231
			2	En desacuerdo	5.050
			3	De acuerdo	16.196
			4	Muy de acuerdo	7.533
230	fa16	Grado de acuerdo: Siente que no cuenta con personas para compartir pena/alegría	1	Muy en desacuerdo	5.111
			2	En desacuerdo	14.703
			3	De acuerdo	8.272
			4	Muy de acuerdo	1.924
231	fa17	Grado de acuerdo: Siente que tiene suficientes personas cercanas	1	Muy en desacuerdo	675
			2	En desacuerdo	3.204
			3	De acuerdo	18.211
			4	Muy de acuerdo	7.920
232	fa18	Grado de acuerdo: En general se lleva bastante bien con su familia y cercanos	1	Muy en desacuerdo	367
			2	En desacuerdo	1.178
			3	De acuerdo	17.851
			4	Muy de acuerdo	10.614
233	fa19_a	¿Con qué frecuencia ha necesitado ayuda para…? Ver	1	Nunca	12.525
			2	Casi nunca	2.502
			3	Algunas veces (sólo algunas veces que realiza la actividad)	2.508
			4	Muchas veces (la mayoría de las veces que realiza la actividad)	753
			5	Siempre (todas las veces que realiza la actividad)	545
234	fa20_a	¿Con qué frecuencia ha necesitado ayuda para…? Oír	1	Nunca	2.135
			2	Casi nunca	736
			3	Algunas veces (sólo algunas veces que realiza la actividad)	967
			4	Muchas veces (la mayoría de las veces que realiza la actividad)	373
			5	Siempre (todas las veces que realiza la actividad)	269
235	fa21_a	¿Con qué frecuencia ha necesitado ayuda para…? Recordar cosas o concentrarse	1	Nunca	4.421
			2	Casi nunca	2.497
			3	Algunas veces (sólo algunas veces que realiza la actividad)	2.915
			4	Muchas veces (la mayoría de las veces que realiza la actividad)	803
			5	Siempre (todas las veces que realiza la actividad)	460
236	fa22_a	¿Con qué frecuencia ha necesitado ayuda para…? Asearse o lavarse	1	Nunca	1.395
			2	Casi nunca	411
			3	Algunas veces (sólo algunas veces que realiza la actividad)	424
			4	Muchas veces (la mayoría de las veces que realiza la actividad)	234
			5	Siempre (todas las veces que realiza la actividad)	485
237	fa23_a	¿Con qué frecuencia ha necesitado ayuda para…? Vestirse	1	Nunca	1.348
			2	Casi nunca	426
			3	Algunas veces (sólo algunas veces que realiza la actividad)	515
			4	Muchas veces (la mayoría de las veces que realiza la actividad)	198

ID	*Nombre variable*	*Etiqueta de variable*	*Valores*	*Etiquetas de valores*	*Frecuencia*
			5	Siempre (todas las veces que realiza la actividad)	462
238	fa24_a	¿Con qué frecuencia ha necesitado ayuda para…? Alimentarse	1	Nunca	983
			2	Casi nunca	336
			3	Algunas veces (sólo algunas veces que realiza la actividad)	241
			4	Muchas veces (la mayoría de las veces que realiza la actividad)	123
			5	Siempre (todas las veces que realiza la actividad)	230
239	fa25_a	¿Con qué frecuencia ha necesitado ayuda para…? Usar el baño	1	Nunca	775
			2	Casi nunca	286
			3	Algunas veces (sólo algunas veces que realiza la actividad)	263
			4	Muchas veces (la mayoría de las veces que realiza la actividad)	152
			5	Siempre (todas las veces que realiza la actividad)	335
240	fa26_a	¿Con qué frecuencia ha necesitado ayuda para…? Manejar sus medicamentos	1	Nunca	1.154
			2	Casi nunca	595
			3	Algunas veces (sólo algunas veces que realiza la actividad)	781
			4	Muchas veces (la mayoría de las veces que realiza la actividad)	388
			5	Siempre (todas las veces que realiza la actividad)	763
241	fa27_a	¿Con qué frecuencia ha necesitado ayuda para…? Conciliar o mantener el sueño	1	Nunca	5.603
			2	Casi nunca	1.402
			3	Algunas veces (sólo algunas veces que realiza la actividad)	1.743
			4	Muchas veces (la mayoría de las veces que realiza la actividad)	704
			5	Siempre (todas las veces que realiza la actividad)	507
242	fa28_a	¿Con qué frecuencia ha necesitado ayuda para…? Hacer las tareas de la casa	1	Nunca	2.025
			2	Casi nunca	981
			3	Algunas veces (sólo algunas veces que realiza la actividad)	1.326
			4	Muchas veces (la mayoría de las veces que realiza la actividad)	583
			5	Siempre (todas las veces que realiza la actividad)	962
243	fa29_a	¿Con qué frecuencia ha necesitado ayuda para…? Cuidar o dar apoyo a otros	1	Nunca	1.531
			2	Casi nunca	749
			3	Algunas veces (sólo algunas veces que realiza la actividad)	799
			4	Muchas veces (la mayoría de las veces que realiza la actividad)	329
			5	Siempre (todas las veces que realiza la actividad)	729
244	fa30_a	¿Con qué frecuencia ha necesitado ayuda para…? Hacer compras o ir al médico	1	Nunca	1.574
			2	Casi nunca	764
			3	Algunas veces (sólo algunas veces que realiza la actividad)	1.249
			4	Muchas veces (la mayoría de las veces que realiza la actividad)	794
			5	Siempre (todas las veces que realiza la actividad)	1.660
245	fa31_a	¿Con qué frecuencia ha necesitado ayuda para…? Participar en fiestas, eventos	1	Nunca	1.924
			2	Casi nunca	686
			3	Algunas veces (sólo algunas veces que realiza la actividad)	685

ID	Nombre variable	Etiqueta de variable	Valores	Etiquetas de valores	Frecuencia
			4	Muchas veces (la mayoría de las veces que realiza la actividad)	357
			5	Siempre (todas las veces que realiza la actividad)	938
246	fa32_a	¿Con qué frecuencia ha necesitado ayuda para…? Relacionarse con gente cercana	1	Nunca	1.426
			2	Casi nunca	480
			3	Algunas veces (sólo algunas veces que realiza la actividad)	502
			4	Muchas veces (la mayoría de las veces que realiza la actividad)	186
			5	Siempre (todas las veces que realiza la actividad)	259
247	fa33_a	¿Con qué frecuencia ha necesitado ayuda para…? Manejar o controlar sus emociones	1	Nunca	9.081
			2	Casi nunca	2.924
			3	Algunas veces (sólo algunas veces que realiza la actividad)	3.395
			4	Muchas veces (la mayoría de las veces que realiza la actividad)	919
			5	Siempre (todas las veces que realiza la actividad)	681
248	fa34_a	¿Con qué frecuencia ha necesitado ayuda para…? Manejar sensaciones de malestar	1	Nunca	10.453
			2	Casi nunca	3.042
			3	Algunas veces (sólo algunas veces que realiza la actividad)	3.552
			4	Muchas veces (la mayoría de las veces que realiza la actividad)	926
			5	Siempre (todas las veces que realiza la actividad)	736
249	fa35_a	¿Con qué frecuencia ha necesitado ayuda para…? Hacer llamadas o comunicarse	1	Nunca	844
			2	Casi nunca	439
			3	Algunas veces (sólo algunas veces que realiza la actividad)	650
			4	Muchas veces (la mayoría de las veces que realiza la actividad)	293
			5	Siempre (todas las veces que realiza la actividad)	638
250	fa36_a	¿Con qué frecuencia ha necesitado ayuda para…? Manejar o administrar dinero	1	Nunca	856
			2	Casi nunca	430
			3	Algunas veces (sólo algunas veces que realiza la actividad)	497
			4	Muchas veces (la mayoría de las veces que realiza la actividad)	244
			5	Siempre (todas las veces que realiza la actividad)	663
251	fa37_a	¿Con qué frecuencia ha necesitado ayuda para…? Caminar o subir peldaños	1	Nunca	4.257
			2	Casi nunca	1.275
			3	Algunas veces (sólo algunas veces que realiza la actividad)	1.570
			4	Muchas veces (la mayoría de las veces que realiza la actividad)	760
			5	Siempre (todas las veces que realiza la actividad)	1.131
252	fa38_a	¿Con qué frecuencia ha necesitado ayuda para…? Moverse o desplazarse en la casa	1	Nunca	1.134
			2	Casi nunca	537
			3	Algunas veces (sólo algunas veces que realiza la actividad)	575
			4	Muchas veces (la mayoría de las veces que realiza la actividad)	254
			5	Siempre (todas las veces que realiza la actividad)	414
253	fa39_a	¿Con qué frecuencia ha necesitado ayuda para…? Salir a la calle	1	Nunca	1.803
			2	Casi nunca	741

ID	*Nombre variable*	*Etiqueta de variable*	*Valores*	*Etiquetas de valores*	*Frecuencia*
			3	Algunas veces (sólo algunas veces que realiza la actividad)	948
			4	Muchas veces (la mayoría de las veces que realiza la actividad)	564
			5	Siempre (todas las veces que realiza la actividad)	1.310
254	fa40_a	¿Con qué frecuencia ha necesitado ayuda para…? Acostarse o levantarse de la cama	1	Nunca	2.090
			2	Casi nunca	708
			3	Algunas veces (sólo algunas veces que realiza la actividad)	751
			4	Muchas veces (la mayoría de las veces que realiza la actividad)	280
			5	Siempre (todas las veces que realiza la actividad)	438
255	fa19_b	¿Con qué frecuencia ha recibido ayuda para…? Ver	1	Nunca	1.267
			2	Casi nunca	1.613
			3	Algunas veces (sólo algunas veces que realiza la actividad)	2.053
			4	Muchas veces (la mayoría de las veces que realiza la actividad)	585
			5	Siempre (todas las veces que realiza la actividad)	790
256	fa20_b	¿Con qué frecuencia ha recibido ayuda para…? Oír	1	Nunca	322
			2	Casi nunca	538
			3	Algunas veces (sólo algunas veces que realiza la actividad)	800
			4	Muchas veces (la mayoría de las veces que realiza la actividad)	318
			5	Siempre (todas las veces que realiza la actividad)	367
257	fa21_b	¿Con qué frecuencia ha recibido ayuda para…? Recordar cosas o concentrarse	1	Nunca	974
			2	Casi nunca	1.828
			3	Algunas veces (sólo algunas veces que realiza la actividad)	2.424
			4	Muchas veces (la mayoría de las veces que realiza la actividad)	696
			5	Siempre (todas las veces que realiza la actividad)	753
258	fa22_b	¿Con qué frecuencia ha recibido ayuda para…? Asearse o lavarse	1	Nunca	156
			2	Casi nunca	261
			3	Algunas veces (sólo algunas veces que realiza la actividad)	399
			4	Muchas veces (la mayoría de las veces que realiza la actividad)	191
			5	Siempre (todas las veces que realiza la actividad)	547
259	fa23_b	¿Con qué frecuencia ha recibido ayuda para…? Vestirse	1	Nunca	164
			2	Casi nunca	292
			3	Algunas veces (sólo algunas veces que realiza la actividad)	447
			4	Muchas veces (la mayoría de las veces que realiza la actividad)	175
			5	Siempre (todas las veces que realiza la actividad)	523
260	fa24_b	¿Con qué frecuencia ha recibido ayuda para…? Alimentarse	1	Nunca	104
			2	Casi nunca	225
			3	Algunas veces (sólo algunas veces que realiza la actividad)	202
			4	Muchas veces (la mayoría de las veces que realiza la actividad)	119
			5	Siempre (todas las veces que realiza la actividad)	280
261	fa25_b	¿Con qué frecuencia ha recibido ayuda para…? Usar el baño	1	Nunca	89

ID	Nombre variable	Etiqueta de variable	Valores	Etiquetas de valores	Frecuencia
			2	Casi nunca	180
			3	Algunas veces (sólo algunas veces que realiza la actividad)	245
			4	Muchas veces (la mayoría de las veces que realiza la actividad)	133
			5	Siempre (todas las veces que realiza la actividad)	389
262	fa26_b	¿Con qué frecuencia ha recibido ayuda para…? Manejar sus medicamentos	1	Nunca	173
			2	Casi nunca	400
			3	Algunas veces (sólo algunas veces que realiza la actividad)	720
			4	Muchas veces (la mayoría de las veces que realiza la actividad)	356
			5	Siempre (todas las veces que realiza la actividad)	878
263	fa27_b	¿Con qué frecuencia ha recibido ayuda para…? Conciliar o mantener el sueño	1	Nunca	910
			2	Casi nunca	998
			3	Algunas veces (sólo algunas veces que realiza la actividad)	1.386
			4	Muchas veces (la mayoría de las veces que realiza la actividad)	507
			5	Siempre (todas las veces que realiza la actividad)	555
264	fa28_b	¿Con qué frecuencia ha recibido ayuda para…? Hacer las tareas de la casa	1	Nunca	337
			2	Casi nunca	663
			3	Algunas veces (sólo algunas veces que realiza la actividad)	1.218
			4	Muchas veces (la mayoría de las veces que realiza la actividad)	582
			5	Siempre (todas las veces que realiza la actividad)	1.052
265	fa29_b	¿Con qué frecuencia ha recibido ayuda para…? Cuidar o dar apoyo a otros	1	Nunca	347
			2	Casi nunca	515
			3	Algunas veces (sólo algunas veces que realiza la actividad)	702
			4	Muchas veces (la mayoría de las veces que realiza la actividad)	284
			5	Siempre (todas las veces que realiza la actividad)	758
266	fa30_b	¿Con qué frecuencia ha recibido ayuda para…? Hacer compras o ir al médico	1	Nunca	267
			2	Casi nunca	587
			3	Algunas veces (sólo algunas veces que realiza la actividad)	1.163
			4	Muchas veces (la mayoría de las veces que realiza la actividad)	672
			5	Siempre (todas las veces que realiza la actividad)	1.778
267	fa31_b	¿Con qué frecuencia ha recibido ayuda para…? Participar en fiestas, eventos	1	Nunca	354
			2	Casi nunca	471
			3	Algunas veces (sólo algunas veces que realiza la actividad)	626
			4	Muchas veces (la mayoría de las veces que realiza la actividad)	310
			5	Siempre (todas las veces que realiza la actividad)	905
268	fa32_b	¿Con qué frecuencia ha recibido ayuda para…? Relacionarse con gente cercana	1	Nunca	215
			2	Casi nunca	341
			3	Algunas veces (sólo algunas veces que realiza la actividad)	433
			4	Muchas veces (la mayoría de las veces que realiza la actividad)	167
			5	Siempre (todas las veces que realiza la actividad)	271

ID	Nombre variable	Etiqueta de variable	Valores	Etiquetas de valores	Frecuencia
269	fa33_b	¿Con qué frecuencia ha recibido ayuda para…? Manejar o controlar sus emociones	1	Nunca	1.257
			2	Casi nunca	2.075
			3	Algunas veces (sólo algunas veces que realiza la actividad)	2.876
			4	Muchas veces (la mayoría de las veces que realiza la actividad)	806
			5	Siempre (todas las veces que realiza la actividad)	905
270	fa34_b	¿Con qué frecuencia ha recibido ayuda para…? Manejar sensaciones de malestar	1	Nunca	1.333
			2	Casi nunca	2.154
			3	Algunas veces (sólo algunas veces que realiza la actividad)	2.949
			4	Muchas veces (la mayoría de las veces que realiza la actividad)	837
			5	Siempre (todas las veces que realiza la actividad)	983
271	fa35_b	¿Con qué frecuencia ha recibido ayuda para…? Hacer llamadas o comunicarse	1	Nunca	125
			2	Casi nunca	345
			3	Algunas veces (sólo algunas veces que realiza la actividad)	556
			4	Muchas veces (la mayoría de las veces que realiza la actividad)	297
			5	Siempre (todas las veces que realiza la actividad)	697
272	fa36_b	¿Con qué frecuencia ha recibido ayuda para…? Manejar o administrar dinero	1	Nunca	144
			2	Casi nunca	310
			3	Algunas veces (sólo algunas veces que realiza la actividad)	434
			4	Muchas veces (la mayoría de las veces que realiza la actividad)	239
			5	Siempre (todas las veces que realiza la actividad)	707
273	fa37_b	¿Con qué frecuencia ha recibido ayuda para…? Caminar o subir peldaños	1	Nunca	547
			2	Casi nunca	968
			3	Algunas veces (sólo algunas veces que realiza la actividad)	1.365
			4	Muchas veces (la mayoría de las veces que realiza la actividad)	662
			5	Siempre (todas las veces que realiza la actividad)	1.194
274	fa38_b	¿Con qué frecuencia ha recibido ayuda para…? Moverse o desplazarse en la casa	1	Nunca	157
			2	Casi nunca	401
			3	Algunas veces (sólo algunas veces que realiza la actividad)	503
			4	Muchas veces (la mayoría de las veces que realiza la actividad)	235
			5	Siempre (todas las veces que realiza la actividad)	484
275	fa39_b	¿Con qué frecuencia ha recibido ayuda para…? Salir a la calle	1	Nunca	223
			2	Casi nunca	550
			3	Algunas veces (sólo algunas veces que realiza la actividad)	881
			4	Muchas veces (la mayoría de las veces que realiza la actividad)	527
			5	Siempre (todas las veces que realiza la actividad)	1.382
276	fa40_b	¿Con qué frecuencia ha recibido ayuda para…? Acostarse o levantarse de la cama	1	Nunca	229
			2	Casi nunca	525
			3	Algunas veces (sólo algunas veces que realiza la actividad)	660
			4	Muchas veces (la mayoría de las veces que realiza la actividad)	244

ID	Nombre variable	Etiqueta de variable	Valores	Etiquetas de valores	Frecuencia
			5	Siempre (todas las veces que realiza la actividad)	519
277	fa41	¿Cuán insatisfecho o satisfecho se encuentra con la ayuda que recibe?	1	Muy insatisfecho(a)	220
			2	Insatisfecho(a)	527
			3	Ni insatisfecho(a) ni satisfecho(a)	1.627
			4	Satisfecho(a)	7.690
			5	Muy satisfecho(a)	3.454
278	fa42	En los últimos 12 meses, ¿producto del COVID-19 su necesidad de asistencia...?	1	Ha disminuido mucho	559
			2	Ha disminuido poco	1.066
			3	Se ha mantenido igual	8.577
			4	Ha aumentado poco	2.137
			5	Ha aumentado mucho	1.179
279	fa43	¿Cuenta con una persona que debido a su salud le proporcione ayuda permanente?	1	Sí	2.937
			2	No	2.571
280	fa45_1	Cuidador(a) 1: Pertenencia al hogar del adulto entrevistado	1	Sí	2.327
			2	No	610
281	fa46_1	Cuidador(a) 1: Identificación dentro del hogar (orden persona hogar)	Rango: 1 - 10		
282	fa47_1	Cuidador(a) 1: Edad cuidador(a) fuera del hogar	Rango: 15 - 99		
283	fa48_1	Cuidador(a) 1: Sexo cuidador(a) fuera del hogar	1	Hombre	122
			2	Mujer	485
284	fa49_1	Cuidador(a) 1: Relación del cuidador(a) con el adulto entrevistado	1	Cónyuge o conviviente	884
			2	Hijo(a) o hijastro(a)	1.083
			3	Yerno o nuera	55
			4	Nieto(a)	87
			5	Hermano(a) o cuñado(a)	186
			6	Padres o suegros	307
			7	Abuelo(a)	15
			8	Otro pariente	114
			9	Vecino(a)	25
			10	Amigo(a)	62
			11	Servicio doméstico puertas adentro	15
			12	Servicio personal de salud	22
			77	Otro no pariente	82
285	fa50_1	Cuidador(a) 1: ¿Recibe remuneración?	1	Sí	151
			2	No	2.786
286	fa45_2	Cuidador(a) 2: Pertenencia al hogar del adulto entrevistado	1	Sí	502
			2	No	416
287	fa46_2	Cuidador(a) 2: Identificación dentro del hogar (orden persona hogar)	Rango: 1 - 9		
288	fa47_2	Cuidador(a) 2: Edad cuidador(a) fuera del hogar	Rango: 2 - 99		
289	fa48_2	Cuidador(a) 2: Sexo cuidador(a) fuera del hogar	1	Hombre	102
			2	Mujer	312
290	fa49_2	Cuidador(a) 2: Relación del cuidador(a) con el adulto entrevistado	1	Cónyuge o conviviente	64
			2	Hijo(a) o hijastro(a)	404
			3	Yerno o nuera	44
			4	Nieto(a)	77
			5	Hermano(a) o cuñado(a)	100
			6	Padres o suegros	71
			7	Abuelo(a)	5
			8	Otro pariente	58
			9	Vecino(a)	15
			10	Amigo(a)	36
			11	Servicio doméstico puertas adentro	4
			12	Servicio personal de salud	8
			77	Otro no pariente	32
291	fa50_2	Cuidador(a) 2: ¿Recibe remuneración?	1	Sí	45
			2	No	873
292	fa45_3	Cuidador(a) 3: Pertenencia al hogar del adulto entrevistado	1	Sí	149
			2	No	188

ID	Nombre variable	Etiqueta de variable	Valores	Etiquetas de valores	Frecuencia
293	fa46_3	Cuidador(a) 3: Identificación dentro del hogar (orden_persona_hogar)	Rango: 1 - 9		
294	fa47_3	Cuidador(a) 3: Edad cuidador(a) fuera del hogar	Rango: 6 - 85		
295	fa48_3	Cuidador(a) 3: Sexo cuidador(a) fuera del hogar	1	Hombre	65
			2	Mujer	123
296	fa49_3	Cuidador(a) 3: Relación del cuidador(a) con el adulto entrevistado	1	Cónyuge o conviviente	16
			2	Hijo(a) o hijastro(a)	158
			3	Yerno o nuera	8
			4	Nieto(a)	33
			5	Hermano(a) o cuñado(a)	41
			6	Padres o suegros	17
			7	Abuelo(a)	0
			8	Otro pariente	20
			9	Vecino(a)	8
			10	Amigo(a)	16
			11	Servicio doméstico puertas adentro	3
			12	Servicio personal de salud	6
			77	Otro no pariente	11
297	fa50_3	Cuidador(a) 3: ¿Recibe remuneración?	1	Sí	16
			2	No	321
298	fa45_4	Cuidador(a) 4: Pertenencia al hogar del adulto entrevistado	1	Sí	36
			2	No	85
299	fa46_4	Cuidador(a) 4: Identificación dentro del hogar (orden_persona_hogar)	Rango: 1 - 7		
300	fa47_4	Cuidador(a) 4: Edad cuidador(a) fuera del hogar	Rango: 18 - 78		
301	fa48_4	Cuidador(a) 4: Sexo cuidador(a) fuera del hogar	1	Hombre	31
			2	Mujer	54
302	fa49_4	Cuidador(a) 4: Relación del cuidador(a) con el adulto entrevistado	1	Cónyuge o conviviente	4
			2	Hijo(a) o hijastro(a)	50
			3	Yerno o nuera	5
			4	Nieto(a)	12
			5	Hermano(a) o cuñado(a)	13
			6	Padres o suegros	5
			7	Abuelo(a)	0
			8	Otro pariente	12
			9	Vecino(a)	1
			10	Amigo(a)	12
			11	Servicio doméstico puertas adentro	2
			12	Servicio personal de salud	1
			77	Otro no pariente	4
303	fa50_4	Cuidador(a) 4: ¿Recibe remuneración?	1	Sí	2
			2	No	119
304	fa45_5	Cuidador(a) 5: Pertenencia al hogar del adulto entrevistado	1	Sí	11
			2	No	37
305	fa46_5	Cuidador(a) 5: Identificación dentro del hogar (orden_persona_hogar)	Rango: 1 - 6		
306	fa47_5	Cuidador(a) 5: Edad cuidador(a) fuera del hogar	Rango: 20 - 71		
307	fa48_5	Cuidador(a) 5: Sexo cuidador(a) fuera del hogar	1	Hombre	19
			2	Mujer	18
308	fa49_5	Cuidador(a) 5: Relación del cuidador(a) con el adulto entrevistado	1	Cónyuge o conviviente	3
			2	Hijo(a) o hijastro(a)	15
			3	Yerno o nuera	6
			4	Nieto(a)	5
			5	Hermano(a) o cuñado(a)	6
			6	Padres o suegros	3
			7	Abuelo(a)	1
			8	Otro pariente	5
			9	Vecino(a)	0
			10	Amigo(a)	3
			11	Servicio doméstico puertas adentro	0

ID	*Nombre variable*	*Etiqueta de variable*	*Valores*	*Etiquetas de valores*	*Frecuencia*
			12	Servicio personal de salud	0
			77	Otro no pariente	1
309	fa50_5	Cuidador(a) 5: ¿Recibe remuneración?	1	Sí	0
			2	No	48
310	fa45_6	Cuidador(a) 6: Pertenencia al hogar del adulto entrevistado	1	Sí	2
			2	No	16
311	fa46_6	Cuidador(a) 6: Identificación dentro del hogar (orden_persona_hogar)	Rango: 1 - 5		
312	fa47_6	Cuidador(a) 6: Edad cuidador(a) fuera del hogar	Rango: 25 - 69		
313	fa48_6	Cuidador(a) 6: Sexo cuidador(a) fuera del hogar	1	Hombre	9
			2	Mujer	7
314	fa49_6	Cuidador(a) 6: Relación del cuidador(a) con el adulto entrevistado	1	Cónyuge o conviviente	0
			2	Hijo(a) o hijastro(a)	7
			3	Yerno o nuera	1
			4	Nieto(a)	0
			5	Hermano(a) o cuñado(a)	1
			6	Padres o suegros	1
			7	Abuelo(a)	0
			8	Otro pariente	2
			9	Vecino(a)	1
			10	Amigo(a)	4
			11	Servicio doméstico puertas adentro	0
			12	Servicio personal de salud	0
			77	Otro no pariente	1
315	fa50_6	Cuidador(a) 6: ¿Recibe remuneración?	1	Sí	0
			2	No	18
316	fa45_7	Cuidador(a) 7: Pertenencia al hogar del adulto entrevistado	1	Sí	0
			2	No	2
317	fa46_7	Cuidador(a) 7: Identificación dentro del hogar (orden_persona_hogar)	Rango: 1 - 20		
318	fa47_7	Cuidador(a) 7: Edad cuidador(a) fuera del hogar	Rango: 35 - 38		
319	fa48_7	Cuidador(a) 7: Sexo cuidador(a) fuera del hogar	1	Hombre	2
			2	Mujer	0
320	fa49_7	Cuidador(a) 7: Relación del cuidador(a) con el adulto entrevistado	1	Cónyuge o conviviente	0
			2	Hijo(a) o hijastro(a)	1
			3	Yerno o nuera	0
			4	Nieto(a)	0
			5	Hermano(a) o cuñado(a)	0
			6	Padres o suegros	0
			7	Abuelo(a)	0
			8	Otro pariente	1
			9	Vecino(a)	0
			10	Amigo(a)	0
			11	Servicio doméstico puertas adentro	0
			12	Servicio personal de salud	0
			77	Otro no pariente	0
321	fa50_7	Cuidador(a) 7: ¿Recibe remuneración?	1	Sí	0
			2	No	2
322	fa45_8	Cuidador(a) 8: Pertenencia al hogar del adulto entrevistado	1	Sí	0
			2	No	2
323	fa46_8	Cuidador(a) 8: Identificación dentro del hogar (orden_persona_hogar)	Rango: 1 - 20		
324	fa47_8	Cuidador(a) 8: Edad cuidador(a) fuera del hogar	Rango: 42 - 45		
325	fa48_8	Cuidador(a) 8: Sexo cuidador(a) fuera del hogar	1	Hombre	1
			2	Mujer	1
326	fa49_8	Cuidador(a) 8: Relación del cuidador(a) con el adulto entrevistado	1	Cónyuge o conviviente	0
			2	Hijo(a) o hijastro(a)	1
			3	Yerno o nuera	0
			4	Nieto(a)	0
			5	Hermano(a) o cuñado(a)	0
			6	Padres o suegros	0

ID	Nombre variable	Etiqueta de variable	Valores	Etiquetas de valores	Frecuencia
			7	Abuelo(a)	0
			8	Otro pariente	1
			9	Vecino(a)	0
			10	Amigo(a)	0
			11	Servicio doméstico puertas adentro	0
			12	Servicio personal de salud	0
			77	Otro no pariente	0
327	fa50_8	Cuidador(a) 8: ¿Recibe remuneración?	1	Sí	0
			2	No	2
328	fa45_9	Cuidador(a) 9: Pertenencia al hogar del adulto entrevistado	1	Sí	0
			2	No	1
329	fa46_9	Cuidador(a) 9: Identificación dentro del hogar (orden_persona_hogar)	Rango: 1 - 20		
330	fa47_9	Cuidador(a) 9: Edad cuidador(a) fuera del hogar	Rango: 42 - 42		
331	fa48_9	Cuidador(a) 9: Sexo cuidador(a) fuera del hogar	1	Hombre	0
			2	Mujer	1
332	fa49_9	Cuidador(a) 9: Relación del cuidador(a) con el adulto entrevistado	1	Cónyuge o conviviente	0
			2	Hijo(a) o hijastro(a)	0
			3	Yerno o nuera	0
			4	Nieto(a)	0
			5	Hermano(a) o cuñado(a)	0
			6	Padres o suegros	0
			7	Abuelo(a)	0
			8	Otro pariente	1
			9	Vecino(a)	0
			10	Amigo(a)	0
			11	Servicio doméstico puertas adentro	0
			12	Servicio personal de salud	0
			77	Otro no pariente	0
333	fa50_9	Cuidador(a) 9: ¿Recibe remuneración?	1	Sí	0
			2	No	1
334	fa45_10	Cuidador(a) 10: Pertenencia al hogar del adulto entrevistado	1	Sí	0
			2	No	1
335	fa46_10	Cuidador(a) 10: Identificación dentro del hogar (orden_persona_hogar)	Rango: 1 - 20		
336	fa47_10	Cuidador(a) 10: Edad cuidador(a) fuera del hogar	Rango: 76 - 76		
337	fa48_10	Cuidador(a) 10: Sexo cuidador(a) fuera del hogar	1	Hombre	0
			2	Mujer	1
338	fa49_10	Cuidador(a) 10: Relación del cuidador(a) con el adulto entrevistado	1	Cónyuge o conviviente	0
			2	Hijo(a) o hijastro(a)	0
			3	Yerno o nuera	0
			4	Nieto(a)	0
			5	Hermano(a) o cuñado(a)	1
			6	Padres o suegros	0
			7	Abuelo(a)	0
			8	Otro pariente	0
			9	Vecino(a)	0
			10	Amigo(a)	0
			11	Servicio doméstico puertas adentro	0
			12	Servicio personal de salud	0
			77	Otro no pariente	0
339	fa50_10	Cuidador(a) 10: ¿Recibe remuneración?	1	Sí	0
			2	No	1
340	fa45_11	Cuidador(a) 11: Pertenencia al hogar del adulto entrevistado	1	Sí	0
			2	No	1
341	fa46_11	Cuidador(a) 11: Identificación dentro del hogar (orden_persona_hogar)	Rango: 1 - 20		
342	fa47_11	Cuidador(a) 11: Edad cuidador(a) fuera del hogar	Rango: 18 - 18		
343	fa48_11	Cuidador(a) 11: Sexo cuidador(a) fuera del hogar	1	Hombre	1
			2	Mujer	0

ID	*Nombre variable*	*Etiqueta de variable*	*Valores*	*Etiquetas de valores*	*Frecuencia*
344	fa49_11	Cuidador(a) 11: Relación del cuidador(a) con el adulto entrevistado	1	Cónyuge o conviviente	0
			2	Hijo(a) o hijastro(a)	0
			3	Yerno o nuera	0
			4	Nieto(a)	0
			5	Hermano(a) o cuñado(a)	0
			6	Padres o suegros	0
			7	Abuelo(a)	0
			8	Otro pariente	1
			9	Vecino(a)	0
			10	Amigo(a)	0
			11	Servicio doméstico puertas adentro	0
			12	Servicio personal de salud	0
			77	Otro no pariente	0
345	fa50_11	Cuidador(a) 11: ¿Recibe remuneración?	1	Sí	0
			2	No	1
346	fa45_12	Cuidador(a) 12: Pertenencia al hogar del adulto entrevistado	1	Sí	0
			2	No	1
347	fa46_12	Cuidador(a) 12: Identificación dentro del hogar (orden_persona_hogar)	Rango: 1 - 20		
348	fa47_12	Cuidador(a) 12: Edad cuidador(a) fuera del hogar	Rango: 56 - 56		
349	fa48_12	Cuidador(a) 12: Sexo cuidador(a) fuera del hogar	1	Hombre	1
			2	Mujer	0
350	fa49_12	Cuidador(a) 12: Relación del cuidador(a) con el adulto entrevistado	1	Cónyuge o conviviente	0
			2	Hijo(a) o hijastro(a)	0
			3	Yerno o nuera	0
			4	Nieto(a)	0
			5	Hermano(a) o cuñado(a)	0
			6	Padres o suegros	0
			7	Abuelo(a)	0
			8	Otro pariente	1
			9	Vecino(a)	0
			10	Amigo(a)	0
			11	Servicio doméstico puertas adentro	0
			12	Servicio personal de salud	0
			77	Otro no pariente	0
351	fa50_12	Cuidador(a) 12: ¿Recibe remuneración?	1	Sí	0
			2	No	1
352	fa45_13	Cuidador(a) 13: Pertenencia al hogar del adulto entrevistado	1	Sí	0
			2	No	1
353	fa46_13	Cuidador(a) 13: Identificación dentro del hogar (orden_persona_hogar)	Rango: 1 - 20		
354	fa47_13	Cuidador(a) 13: Edad cuidador(a) fuera del hogar	Rango: 45 - 45		
355	fa48_13	Cuidador(a) 13: Sexo cuidador(a) fuera del hogar	1	Hombre	0
			2	Mujer	1
356	fa49_13	Cuidador(a) 13: Relación del cuidador(a) con el adulto entrevistado	1	Cónyuge o conviviente	0
			2	Hijo(a) o hijastro(a)	0
			3	Yerno o nuera	0
			4	Nieto(a)	0
			5	Hermano(a) o cuñado(a)	0
			6	Padres o suegros	0
			7	Abuelo(a)	0
			8	Otro pariente	1
			9	Vecino(a)	0
			10	Amigo(a)	0
			11	Servicio doméstico puertas adentro	0
			12	Servicio personal de salud	0
			77	Otro no pariente	0
357	fa50_13	Cuidador(a) 13: ¿Recibe remuneración?	1	Sí	0
			2	No	1

ID	Nombre variable	Etiqueta de variable	Valores	Etiquetas de valores	Frecuencia
358	fa51	Identificación cuidador(a) principal (número de columna cuidador rango 1 a 13)	Rango: 1 - 13		
359	fa52	Identificación cuidador(a) principal del hogar (número de columna cuidador)	Rango: 1 - 5		
360	fa53	Además de la ayuda que recibe, ¿cree usted que necesita ayuda adicional?	1	Sí	1.956
			2	No	11.562
361	fa54	Debido a su salud, ¿cree que necesita a alguien que le ayude?	1	Sí	135
			2	No	1.802
362	fa55_1	Ayudas técnicas utilizadas: Ninguna	0	No	16.901
			1	Sí	13.109
363	fa55_2	Ayudas técnicas utilizadas: Anteojos o lentes de contacto para ver	0	No	14.178
			1	Sí	15.832
364	fa55_3	Ayudas técnicas utilizadas: Audífonos	0	No	29.274
			1	Sí	736
365	fa55_4	Ayudas técnicas utilizadas: Muleta o bastón	0	No	28.784
			1	Sí	1.226
366	fa55_5	Ayudas técnicas utilizadas: Andadores	0	No	29.691
			1	Sí	319
367	fa55_6	Ayudas técnicas utilizadas: Silla de ruedas	0	No	29.493
			1	Sí	517
368	fa55_7	Ayudas técnicas utilizadas: Bastón blanco o bastón guiador	0	No	29.711
			1	Sí	299
369	fa55_8	Ayudas técnicas utilizadas: Calzado ortopédico y/o plantillas	0	No	29.677
			1	Sí	333
370	fa55_9	Ayudas técnicas utilizadas: Cojines y/o colchones antiescaras	0	No	29.860
			1	Sí	150
371	fa55_10	Ayudas técnicas utilizadas: Catre clínico	0	No	29.918
			1	Sí	92
372	fa55_11	Ayudas técnicas utilizadas: Órtesis para miembro inferior, superior o columna	0	No	29.957
			1	Sí	53
373	fa55_12	Ayudas técnicas utilizadas: Prótesis de miembro inferior	0	No	29.887
			1	Sí	123
374	fa55_13	Ayudas técnicas utilizadas: Prótesis de miembro superior	0	No	29.971
			1	Sí	39
375	fa55_14	Ayudas técnicas utilizadas: Bipedestador	0	No	30.008
			1	Sí	2
376	fa55_15	Ayudas técnicas utilizadas: Productos para transferencias	0	No	30.009
			1	Sí	1
377	fa55_16	Ayudas técnicas utilizadas: Equipos tecnológicos adaptados	0	No	29.919
			1	Sí	91
378	fa55_17	Ayudas técnicas utilizadas: Teclado o mouse adaptados	0	No	29.980
			1	Sí	30
379	fa55_18	Ayudas técnicas utilizadas: Programas de accesibilidad informática	0	No	29.993
			1	Sí	17
380	fa55_19	Ayudas técnicas utilizadas: Tablero de comunicación	0	No	30.007
			1	Sí	3
381	fa55_20	Ayudas técnicas utilizadas: Lupa	0	No	29.885
			1	Sí	125
382	fa55_21	Ayudas técnicas utilizadas: Equipos para proporcionar oxígeno	0	No	29.968
			1	Sí	42
383	fa55_77	Ayudas técnicas utilizadas: Otros	0	No	29.696
			1	Sí	314
384	fa56_1	Ayudas técnicas adicionales: Ninguna	0	No	4.562
			1	Sí	12.339
385	fa56_2	Ayudas técnicas adicionales: Anteojos o lentes de contacto para ver	0	No	14.247
			1	Sí	2.654
386	fa56_3	Ayudas técnicas adicionales: Audífonos	0	No	15.922
			1	Sí	979
387	fa56_4	Ayudas técnicas adicionales: Muleta o bastón	0	No	16.424
			1	Sí	477
388	fa56_5	Ayudas técnicas adicionales: Andadores	0	No	16.743
			1	Sí	158
389	fa56_6	Ayudas técnicas adicionales: Silla de ruedas	0	No	16.689

ID	*Nombre variable*	*Etiqueta de variable*	*Valores*	*Etiquetas de valores*	*Frecuencia*
			1	Sí	212
390	fa56_7	Ayudas técnicas adicionales: Bastón blanco o bastón guiador	0	No	16.754
			1	Sí	147
391	fa56_8	Ayudas técnicas adicionales: Calzado ortopédico y/o plantillas	0	No	16.632
			1	Sí	269
392	fa56_9	Ayudas técnicas adicionales: Cojines y/o colchones antiescaras	0	No	16.744
			1	Sí	157
393	fa56_10	Ayudas técnicas adicionales: Catre clínico	0	No	16.761
			1	Sí	140
394	fa56_11	Ayudas técnicas adicionales: Órtesis para miembro inferior, superior o columna	0	No	16.875
			1	Sí	26
395	fa56_12	Ayudas técnicas adicionales: Prótesis de miembro inferior	0	No	16.852
			1	Sí	49
396	fa56_13	Ayudas técnicas adicionales: Prótesis de miembro superior	0	No	16.890
			1	Sí	11
397	fa56_14	Ayudas técnicas adicionales: Bipedestador	0	No	16.892
			1	Sí	9
398	fa56_15	Ayudas técnicas adicionales: Productos para transferencias	0	No	16.885
			1	Sí	16
399	fa56_16	Ayudas técnicas adicionales: Equipos tecnológicos adaptados	0	No	16.827
			1	Sí	74
400	fa56_17	Ayudas técnicas adicionales: Teclado o mouse adaptados	0	No	16.876
			1	Sí	25
401	fa56_18	Ayudas técnicas adicionales: Programas de accesibilidad informática	0	No	16.856
			1	Sí	45
402	fa56_19	Ayudas técnicas adicionales: Tablero de comunicación	0	No	16.892
			1	Sí	9
403	fa56_20	Ayudas técnicas adicionales: Lupa	0	No	16.825
			1	Sí	76
404	fa56_21	Ayudas técnicas adicionales: Equipos para proporcionar oxígeno	0	No	16.872
			1	Sí	29
405	fa56_77	Ayudas técnicas adicionales: Otros	0	No	16.617
			1	Sí	284
406	fa57_1	No utiliza. Ayudas técnicas necesarias: Ninguna	0	No	2.665
			1	Sí	10.444
407	fa57_2	No utiliza. Ayudas técnicas necesarias: Anteojos o lentes de contacto para ver	0	No	10.761
			1	Sí	2.348
408	fa57_3	No utiliza. Ayudas técnicas necesarias: Audífonos	0	No	12.830
			1	Sí	279
409	fa57_4	No utiliza. Ayudas técnicas necesarias: Muleta o bastón	0	No	13.010
			1	Sí	99
410	fa57_5	No utiliza. Ayudas técnicas necesarias: Andadores	0	No	13.090
			1	Sí	19
411	fa57_6	No utiliza. Ayudas técnicas necesarias: Silla de ruedas	0	No	13.085
			1	Sí	24
412	fa57_7	No utiliza. Ayudas técnicas necesarias: Bastón blanco o bastón guiador	0	No	13.072
			1	Sí	37
413	fa57_8	No utiliza. Ayudas técnicas necesarias: Calzado ortopédico y/o plantillas	0	No	13.040
			1	Sí	69
414	fa57_9	No utiliza. Ayudas técnicas necesarias: Cojines y/o colchones antiescaras	0	No	13.098
			1	Sí	11
415	fa57_10	No utiliza. Ayudas técnicas necesarias: Catre clínico	0	No	13.095
			1	Sí	14
416	fa57_11	No utiliza. Ayudas técnicas necesarias: Órtesis para miembro inf./sup. o columna	0	No	13.101
			1	Sí	8
417	fa57_12	No utiliza. Ayudas técnicas necesarias: Prótesis de miembro inferior	0	No	13.105
			1	Sí	4
418	fa57_13	No utiliza. Ayudas técnicas necesarias: Prótesis de miembro superior	0	No	13.104
			1	Sí	5
419	fa57_14	No utiliza. Ayudas técnicas necesarias: Bipedestador	0	No	13.108
			1	Sí	1
420	fa57_15	No utiliza. Ayudas técnicas necesarias: Productos para transferencias	0	No	13.107
			1	Sí	2

ID	*Nombre variable*	*Etiqueta de variable*	*Valores*	*Etiquetas de valores*	*Frecuencia*
421	fa57_16	No utiliza. Ayudas técnicas necesarias: Equipos tecnológicos adaptados	0	No	13.096
			1	Sí	13
422	fa57_17	No utiliza. Ayudas técnicas necesarias: Teclado o mouse adaptados	0	No	13.107
			1	Sí	2
423	fa57_18	No utiliza. Ayudas técnicas necesarias: Programas de accesibilidad informática	0	No	13.104
			1	Sí	5
424	fa57_19	No utiliza. Ayudas técnicas necesarias: Tablero de comunicación	0	No	13.108
			1	Sí	1
425	fa57_20	No utiliza. Ayudas técnicas necesarias: Lupa	0	No	13.099
			1	Sí	10
426	fa57_21	No utiliza. Ayudas técnicas necesarias: Equipos para proporcionar oxígeno	0	No	13.107
			1	Sí	2
427	fa57_77	No utiliza. Ayudas técnicas necesarias: Otros	0	No	13.068
			1	Sí	41
428	fa58	Últ. 30 días, ¿qué tan insatisfecho o satisfecho está con sus recursos de apoyo?	1	Muy insatisfecho(a)	475
			2	Insatisfecho(a)	2.066
			3	Ni insatisfecho(a) ni satisfecho(a)	1.799
			4	Satisfecho(a)	9.312
			5	Muy satisfecho(a)	3.249
429	fa59_1	Razón de insatisfacción con ayuda técnica: Talla o forma inadecuada	0	No	1.780
			1	Sí	761
430	fa59_2	Razón de insatisfacción con ayuda técnica: Uso incómodo o causa dolor	0	No	2.283
			1	Sí	258
431	fa59_3	Razón de insatisfacción con ayuda técnica: Muy pesado o grande	0	No	2.493
			1	Sí	48
432	fa59_4	Razón de insatisfacción con ayuda técnica: No le agrada aspecto o apariencia	0	No	2.484
			1	Sí	57
433	fa59_5	Razón de insatisfacción con ayuda técnica: No entrega seguridad o estabilidad	0	No	2.363
			1	Sí	178
434	fa59_6	Razón de insatisfacción con ayuda técnica: Deteriorado, cumplió vida útil	0	No	1.063
			1	Sí	1.478
435	fa59_77	Razón de insatisfacción con ayuda técnica: Otro	0	No	2.333
			1	Sí	208
436	fa60_1	Adaptaciones de vivienda utilizadas: Rampas o rebajes	0	No	29.643
			1	Sí	367
437	fa60_2	Adaptaciones de vivienda utilizadas: Pisos adaptados	0	No	29.754
			1	Sí	256
438	fa60_3	Adaptaciones de vivienda utilizadas: Puertas automáticas	0	No	30.004
			1	Sí	6
439	fa60_4	Adaptaciones de vivienda utilizadas: Puertas, manillas adaptadas	0	No	29.881
			1	Sí	129
440	fa60_5	Adaptaciones de vivienda utilizadas: Ascensor o dispositivo elevador	0	No	29.974
			1	Sí	36
441	fa60_6	Adaptaciones de vivienda utilizadas: Ampliación de puertas, pasillos	0	No	29.879
			1	Sí	131
442	fa60_7	Adaptaciones de vivienda utilizadas: Barandas, pasamanos	0	No	28.344
			1	Sí	1.666
443	fa60_8	Adaptaciones de vivienda utilizadas: Tina o ducha, silla elevable	0	No	29.478
			1	Sí	532
444	fa60_9	Adaptaciones de vivienda utilizadas: Mobiliario adaptado	0	No	29.873
			1	Sí	137
445	fa60_10	Adaptaciones de vivienda utilizadas: Alarmas, timbres, citófonos u otros	0	No	29.869
			1	Sí	141
446	fa60_11	Adaptaciones de vivienda utilizadas: Luminaria, enchufe adaptado	0	No	29.882
			1	Sí	128
447	fa60_12	Adaptaciones de vivienda utilizadas: Señalización de recintos	0	No	29.998
			1	Sí	12
448	fa60_77	Adaptaciones de vivienda utilizadas: Otra	0	No	29.924
			1	Sí	86
449	fa60_78	Adaptaciones de vivienda utilizadas: No, ninguna	0	No	2.560
			1	Sí	27.450
450	fa61_1	Adaptaciones de vivienda adicionales: Rampas o rebajes	0	No	2.358
			1	Sí	202

ID	*Nombre variable*	*Etiqueta de variable*	*Valores*	*Etiquetas de valores*	*Frecuencia*
451	fa61_2	Adaptaciones de vivienda adicionales: Pisos adaptados	0	No	2.406
			1	Sí	154
452	fa61_3	Adaptaciones de vivienda adicionales: Puertas automáticas	0	No	2.528
			1	Sí	32
453	fa61_4	Adaptaciones de vivienda adicionales: Puertas, manillas adaptadas	0	No	2.485
			1	Sí	75
454	fa61_5	Adaptaciones de vivienda adicionales: Ascensor o dispositivo elevador	0	No	2.505
			1	Sí	55
455	fa61_6	Adaptaciones de vivienda adicionales: Ampliación de puertas, pasillos	0	No	2.458
			1	Sí	102
456	fa61_7	Adaptaciones de vivienda adicionales: Barandas, pasamanos	0	No	2.199
			1	Sí	361
457	fa61_8	Adaptaciones de vivienda adicionales: Tina o ducha, silla elevable	0	No	2.253
			1	Sí	307
458	fa61_9	Adaptaciones de vivienda adicionales: Mobiliario adaptado	0	No	2.455
			1	Sí	105
459	fa61_10	Adaptaciones de vivienda adicionales: Alarmas, timbres, citófonos u otros	0	No	2.441
			1	Sí	119
460	fa61_11	Adaptaciones de vivienda adicionales: Luminaria, enchufe adaptado	0	No	2.480
			1	Sí	80
461	fa61_12	Adaptaciones de vivienda adicionales: Señalización de recintos	0	No	2.538
			1	Sí	22
462	fa61_77	Adaptaciones de vivienda adicionales: Otra	0	No	2.505
			1	Sí	55
463	fa61_78	Adaptaciones de vivienda adicionales: No, ninguna	0	No	965
			1	Sí	1.595
464	fa62_1	No utiliza. Adaptaciones de vivienda necesarias: Rampas o rebajes	0	No	27.038
			1	Sí	412
465	fa62_2	No utiliza. Adaptaciones de vivienda necesarias: Pisos adaptados	0	No	27.092
			1	Sí	358
466	fa62_3	No utiliza. Adaptaciones de vivienda necesarias: Puertas automáticas	0	No	27.396
			1	Sí	54
467	fa62_4	No utiliza. Adaptaciones de vivienda necesarias: Puertas, manillas adaptadas	0	No	27.309
			1	Sí	141
468	fa62_5	No utiliza. Adaptaciones de vivienda necesarias: Ascensor o dispositivo elevador	0	No	27.357
			1	Sí	93
469	fa62_6	No utiliza. Adaptaciones de vivienda necesarias: Ampliación de puertas, pasillos	0	No	27.227
			1	Sí	223
470	fa62_7	No utiliza. Adaptaciones de vivienda necesarias: Barandas, pasamanos	0	No	25.884
			1	Sí	1.566
471	fa62_8	No utiliza. Adaptaciones de vivienda necesarias: Tina o ducha, silla elevable	0	No	26.526
			1	Sí	924
472	fa62_9	No utiliza. Adaptaciones de vivienda necesarias: Mobiliario adaptado	0	No	27.208
			1	Sí	242
473	fa62_10	No utiliza. Adaptaciones de vivienda necesarias: Alarmas, timbres, citófonos	0	No	27.166
			1	Sí	284
474	fa62_11	No utiliza. Adaptaciones de vivienda necesarias: Luminaria, enchufe adaptado	0	No	27.177
			1	Sí	273
475	fa62_12	No utiliza. Adaptaciones de vivienda necesarias: Señalización de recintos	0	No	27.426
			1	Sí	24
476	fa62_77	No utiliza. Adaptaciones de vivienda necesarias: Otra	0	No	27.362
			1	Sí	88
477	fa62_78	No utiliza. Adaptaciones de vivienda necesarias: No, ninguna	0	No	2.548
			1	Sí	24.902
478	s1	Últ. 12 meses, veces que ha recibido atención o consulta de salud	-88	No sabe/No Responde	163
			0	Ninguna	7.627
			Válidos		22.220
479	s2	Últ. 12 meses, est. de salud en que recibió atención de forma más frecuente	-88	No sabe/No Responde	13
			1	Centro de Salud Familiar CESFAM o consultorio (Municipal)	9.978
			2	Posta Rural (Municipal o SNSS)	609
			3	CRS o CDT (Consultorio de especialidades del SNSS)	134

ID	Nombre variable	Etiqueta de variable	Valores	Etiquetas de valores	Frecuencia
			4	COSAM (Centro de salud mental comunitaria)	77
			5	SAPU (Servicio de Atención Primaria de Urgencia)	474
			6	SAMU (Servicio de Atención Médico de Urgencias)	92
			7	Posta (Servicio de urgencia de hospital público)	388
			8	Hospital público o del SNSS	2.748
			9	CCR, Centro Comunitario de Rehabilitación	16
			10	Consulta, centro médico, clínica u hospital privado	6.559
			11	Centro de salud mental privado	135
			12	Establecimiento de las FF.AA. o del Orden	204
			13	Servicio de urgencia de clínica u hospital privado	431
			14	Mutual de seguridad	168
			15	Servicio médico de alumnos del lugar en que estudia	19
			77	Otro	175
480	s3_1	Últ. 12 meses, profesional que lo atendió más: Médico general	0	No	6.252
			1	Sí	15.968
481	s3_2	Últ. 12 meses, profesional que lo atendió más: Traumatólogo	0	No	20.139
			1	Sí	2.081
482	s3_3	Últ. 12 meses, profesional que lo atendió más: Ginecólogo	0	No	20.562
			1	Sí	1.658
483	s3_4	Últ. 12 meses, profesional que lo atendió más: Psiquiatra	0	No	21.427
			1	Sí	793
484	s3_5	Últ. 12 meses, profesional que lo atendió más: Oftalmólogo	0	No	20.441
			1	Sí	1.779
485	s3_6	Últ. 12 meses, profesional que lo atendió más: Neurólogo	0	No	21.489
			1	Sí	731
486	s3_7	Últ. 12 meses, profesional que lo atendió más: Geriatra	0	No	22.026
			1	Sí	194
487	s3_8	Últ. 12 meses, profesional que lo atendió más: Otro médico especialista	0	No	19.039
			1	Sí	3.181
488	s3_9	Últ. 12 meses, profesional que lo atendió más: Enfermero	0	No	20.027
			1	Sí	2.193
489	s3_10	Últ. 12 meses, profesional que lo atendió más: Matrona	0	No	20.773
			1	Sí	1.447
490	s3_11	Últ. 12 meses, profesional que lo atendió más: Dentista	0	No	20.158
			1	Sí	2.062
491	s3_12	Últ. 12 meses, profesional que lo atendió más: Kinesiólogo	0	No	21.182
			1	Sí	1.038
492	s3_13	Últ. 12 meses, profesional que lo atendió más: Psicólogo	0	No	20.960
			1	Sí	1.260
493	s3_14	Últ. 12 meses, profesional que lo atendió más: Nutricionista	0	No	20.971
			1	Sí	1.249
494	s3_15	Últ. 12 meses, profesional que lo atendió más: Tecnólogo médico	0	No	21.959
			1	Sí	261
495	s3_16	Últ. 12 meses, profesional que lo atendió más: Fonoaudiólogo	0	No	22.080
			1	Sí	140
496	s3_17	Últ. 12 meses, profesional que lo atendió más: Terapeuta ocupacional	0	No	22.118
			1	Sí	102
497	s3_18	Últ. 12 meses, profesional que lo atendió más: Especialista en med. alternativa	0	No	22.152
			1	Sí	68
498	s3_77	Últ. 12 meses, profesional que lo atendió más: Otro	0	No	21.018
			1	Sí	1.202
499	s3_88	Últ. 12 meses, profesional que lo atendió más: No sabe/No responde	0	No	22.197
			1	Sí	23

ID	Nombre variable	Etiqueta de variable	Valores	Etiquetas de valores	Frecuencia
500	s4	Últ. 12 meses, razón más frecuente por la que necesitó atención	1	Enfermedad contagiosa (infecciones, malaria, tuberculosis, VIH, COVID-19)	1.275
			2	Afecciones relacionadas con la maternidad o perinatales (embarazo, parto y posparto)	254
			3	Deficiencias nutricionales	72
			4	Enfermedades agudas (diarrea, fiebre, gripe, dolores fuertes de cabeza, tos, gastritis, otros)	1.752
			5	Lesiones no relacionadas con el trabajo	660
			6	Enfermedades o lesiones relacionadas con su trabajo u ocupación	423
			7	Cirugía (atenciones relacionadas a cirugías: control postcirugía, planificación cirugía, etc.)	679
			8	Problemas para dormir	154
			9	Dolor crónico muscular o en las articulaciones	1.639
			10	Diabetes o complicaciones asociadas	1.101
			11	Problemas cardiacos (incluso dolor en el pecho sin causa aparente)	387
			12	Problemas bucodentales: en la boca, en los dientes o al tragar	520
			13	Problemas respiratorios	610
			14	Problemas visuales o auditivos	515
			15	Presión arterial alta o hipertensión	1.016
			16	Accidente cerebrovascular o parálisis repentina de una parte del cuerpo	124
			17	Dolor generalizado (de estómago, muscular o inespecífico)	571
			18	Problemas de salud mental: depresión, estrés, ansiedad, entre otros	1.119
			19	Problemas de memoria, alzheimer y/o demencia	77
			20	Cáncer	287
			21	Chequeo preventivo	6.330
			77	Otro	2.655
501	s5	Últ. 12 meses, necesitó atención de salud ambulatoria pero no la recibió	1	Sí	2.955
			2	No	27.055
502	s6	Principal razón por la que no recibió la atención de salud que necesitaba	1	No había disponibilidad del profesional especialista requerido	1.538
			2	No podía pagar el costo de la atención	199
			3	Le suspendieron la atención o tratamiento debido a la pandemia COVID-19	222
			4	Decidió no asistir debido a la pandemia COVID-19	86
			5	Intentó conseguir asistencia de salud, pero se la negaron	232
			6	Lo trataron mal o con negligencia anteriormente y decidió no asistir	66

ID	Nombre variable	Etiqueta de variable	Valores	Etiquetas de valores	Frecuencia
			7	No le pareció confiable o adecuada la atención que recibiría	38
			8	Tuvo problemas para llegar al establecimiento de salud	75
			9	No podía ausentarse del trabajo o tenía otros compromisos	100
			10	No lo consideró necesario	180
			77	Otra razón	219
503	s7	Últ. 12 meses, número de veces hospitalizado al menos una noche	-88	No sabe/No Responde	256
			0	Ninguna	27.317
			Válidos		2.437
504	s8	Últ. 12 meses, necesitó estar hospitalizado pero no recibió atención	1	Sí	363
			2	No	29.647
505	s9	Principal razón por la que no fue hospitalizado	1	No había disponibilidad del profesional especialista requerido	119
			2	No podía pagar el costo de la atención	32
			3	Le suspendieron la atención o tratamiento debido a la pandemia COVID-19	52
			4	Decidió no asistir debido a la pandemia COVID-19	12
			5	Intentó conseguir asistencia de salud, pero se la negaron	40
			6	Lo trataron mal o con negligencia anteriormente y decidió no asistir	9
			7	No le pareció confiable o adecuada la atención que recibiría	3
			8	Tuvo problemas para llegar al establecimiento de salud	2
			9	No podía ausentarse del trabajo o tenía otros compromisos	9
			10	No lo consideró necesario	25
			77	Otra razón	60
506	s10	Últ. 12 meses, número de veces que ha recibido atención de rehabilitación	-88	No sabe/No Responde	436
			0	Ninguna	27.384
			Válidos		2.190
507	s11	Últ. 12 meses, est de salud en que recibió rehabilitación de forma más frecuente	-88	No sabe/No responde	3
			1	Centro de Salud Familiar CESFAM o consultorio (Municipal)	521
			2	Posta Rural (Municipal o SNSS)	24
			3	CRS o CDT (Consultorio de especialidades del SNSS)	23
			4	COSAM (Centro de salud mental comunitaria)	26
			5	SAPU (Servicio de Atención Primaria de Urgencia)	7
			6	SAMU (Servicio de Atención Médico de Urgencias)	0
			7	Posta (servicio de urgencia de hospital público)	30
			8	Hospital público o del SNSS	336
			9	CCR, Centro Comunitario de Rehabilitación	57
			10	Centro diurno o centro de día (para personas mayores o con discapacidad)	13
			11	Consulta, centro médico, clínica u hospital privado	831
			12	Centro de salud mental privado	22

ID	*Nombre variable*	*Etiqueta de variable*	*Valores*	*Etiquetas de valores*	*Frecuencia*
			13	Establecimiento de las FF.AA. o del Orden	26
			14	Servicio de urgencia de clínica privada	21
			15	Mutual de seguridad	68
			16	Servicio médico de alumnos del lugar en que estudia	10
			17	En su domicilio	124
			77	Otro	48
508	s12_1	Últ. 12 meses, prof. de rehabilitación que lo atendió más: Traumatólogo	0	No	1.769
			1	Sí	421
509	s12_2	Últ. 12 meses, prof. de rehabilitación que lo atendió más: Ginecólogo	0	No	2.155
			1	Sí	35
510	s12_3	Últ. 12 meses, prof. de rehabilitación que lo atendió más: Psiquiatra	0	No	2.082
			1	Sí	108
511	s12_4	Últ. 12 meses, prof. de rehabilitación que lo atendió más: Oftalmólogo	0	No	2.145
			1	Sí	45
512	s12_5	Últ. 12 meses, prof. de rehabilitación que lo atendió más: Neurólogo	0	No	2.149
			1	Sí	41
513	s12_6	Últ. 12 meses, prof. de rehabilitación que lo atendió más: Geriatra	0	No	2.177
			1	Sí	13
514	s12_7	Últ. 12 meses, prof. de rehabilitación que lo atendió más: Fisiatra	0	No	2.155
			1	Sí	35
515	s12_8	Últ. 12 meses, prof. de rehabilitación que lo atendió más: Otro med especialista	0	No	2.021
			1	Sí	169
516	s12_9	Últ. 12 meses, prof. de rehabilitación que lo atendió más: Kinesiólogo	0	No	689
			1	Sí	1.501
517	s12_10	Últ. 12 meses, prof. de rehabilitación que lo atendió más: Psicólogo	0	No	1.997
			1	Sí	193
518	s12_11	Últ. 12 meses, prof. de rehabilitación que lo atendió más: Fonoaudiólogo	0	No	2.146
			1	Sí	44
519	s12_12	Últ. 12 meses, prof. de rehabilitación que lo atendió más: Terapeuta ocupacional	0	No	2.103
			1	Sí	87
520	s12_13	Últ. 12 meses, prof. de rehabilitación que lo atendió más: Especialista med. alt	0	No	2.170
			1	Sí	20
521	s12_77	Últ. 12 meses, prof. de rehabilitación que lo atendió más: Otro	0	No	2.071
			1	Sí	119
522	s12_88	Últ. 12 meses, prof. de rehabilitación que lo atendió más: No sabe/No responde	0	No	2.175
			1	Sí	15
523	s13	Últ. 12 meses, necesitó atención de rehabilitación y no recibió	1	Sí	838
			2	No	29.172
524	s14	Principal razón por la que no recibió la atención de rehabilitación necesitada	1	No había disponibilidad del profesional especialista requerido	342
			2	No podía pagar el costo de la atención	136
			3	Le suspendieron la atención o tratamiento debido a la pandemia COVID-19	62
			4	Decidió no asistir debido a la pandemia COVID-19	20
			5	Intentó conseguir asistencia de salud, pero se la negaron	55
			6	Lo trataron mal o con negligencia anteriormente y decidió no asistir	6
			7	No le pareció confiable o adecuada la atención que recibiría	6
			8	Tuvo problemas para llegar al establecimiento de salud	27
			9	No podía ausentarse del trabajo o tenía otros compromisos	53
			10	No lo consideró necesario	62

ID	Nombre variable	Etiqueta de variable	Valores	Etiquetas de valores	Frecuencia
			77	Otra razón	69
525	s15	¿A qué sistema previsional de salud pertenece usted?	-88	No Sabe/No responde	436
			1	FONASA A	6.315
			2	FONASA B	9.057
			3	FONASA C	2.959
			4	FONASA D	2.949
			5	FONASA, no sabe grupo	4.034
			6	FF.AA. y del Orden	438
			7	Isapre	3.082
			8	Ninguno (Particular)	559
			9	Otro sistema	181
526	s16	Últ. 12 meses, ¿con qué frecuencia ha sido tratado injustamente o discriminado?	-88	No sabe/No responde	95
			1	Nunca	25.613
			2	Pocas veces	2.314
			3	Algunas veces	1.346
			4	Muchas veces	536
			5	Siempre	106
527	s17_1	¿Qué tipo de trato…? Lo han tratado con menos respeto o amabilidad que al resto	0	No	2.092
			1	Sí	2.210
528	s17_2	¿Qué tipo de trato…? Han actuado como si tuvieran miedo de usted	0	No	4.054
			1	Sí	248
529	s17_3	¿Qué tipo de trato…? Han pensado que usted no es una persona honrada	0	No	4.019
			1	Sí	283
530	s17_4	¿Qué tipo de trato…? Han creído ser mejores personas que usted	0	No	3.213
			1	Sí	1.089
531	s17_5	¿Qué tipo de trato…? Se han referido a usted con apodos o insultos	0	No	3.718
			1	Sí	584
532	s17_6	¿Qué tipo de trato…? Ha sido amenazado y/o acosado	0	No	3.937
			1	Sí	365
533	s17_7	¿Qué tipo de trato…? Ha sido maltratado físicamente	0	No	4.151
			1	Sí	151
534	s17_8	¿Qué tipo de trato…? Las personas actuaron como si usted no fuera inteligente	0	No	3.572
			1	Sí	730
535	s17_9	¿Qué tipo de trato…? Recibió una peor atención o servicio que otras personas	0	No	3.614
			1	Sí	688
536	s17_10	¿Qué tipo de trato…? Las personas actuaron como si usted no pudiera hacer cosas	0	No	3.895
			1	Sí	407
537	s17_11	¿Qué tipo de trato…? Se ha sentido excluido o le han negado el uso o acceso	0	No	3.919
			1	Sí	383
538	s17_12	¿Qué tipo de trato…? Ha sido tratado de forma inadecuada por un funcionario	0	No	3.706
			1	Sí	596
539	s17_77	¿Qué tipo de trato…? Otro	0	No	3.955
			1	Sí	347
540	s18_1	Razones discriminación: Por su nivel socioeconómico	0	No	3.416
			1	Sí	886
541	s18_2	Razones discriminación: Por ser mujer o ser hombre	0	No	3.665
			1	Sí	637
542	s18_3	Razones discriminación: Por su estado civil	0	No	4.242
			1	Sí	60
543	s18_4	Razones discriminación: Por su ropa	0	No	3.983
			1	Sí	319
544	s18_5	Razones discriminación: Por ser extranjero	0	No	4.076
			1	Sí	226
545	s18_6	Razones discriminación: Por su color de piel	0	No	4.210
			1	Sí	92
546	s18_7	Razones discriminación: Por pertenecer a un pueblo indígena	0	No	4.243
			1	Sí	59
547	s18_8	Razones discriminación: Por su edad	0	No	3.431
			1	Sí	871
548	s18_9	Razones discriminación: Por su orientación sexual o identidad de género	0	No	4.240
			1	Sí	62
549	s18_10	Razones discriminación: Por tener tatuajes, piercing, perforaciones, expansiones	0	No	4.215
			1	Sí	87

ID	*Nombre variable*	*Etiqueta de variable*	*Valores*	*Etiquetas de valores*	*Frecuencia*
550	s18_11	Razones discriminación: Por su cuerpo o apariencia física	0	No	3.716
			1	Sí	586
551	s18_12	Razones discriminación: Por sus creencias o religión	0	No	4.204
			1	Sí	98
552	s18_13	Razones discriminación: Por su ideología u opinión política	0	No	4.103
			1	Sí	199
553	s18_14	Razones discriminación: Por participar o no en sindicatos u organizaciones	0	No	4.259
			1	Sí	43
554	s18_15	Razones discriminación: Por el lugar donde vive	0	No	4.061
			1	Sí	241
555	s18_16	Razones discriminación: Por el establecimiento educacional donde estudió	0	No	4.246
			1	Sí	56
556	s18_17	Razones discriminación: Por su nivel educacional	0	No	4.069
			1	Sí	233
557	s18_18	Razones discriminación: Por su condición de salud o tener alguna discapacidad	0	No	3.738
			1	Sí	564
558	s18_77	Razones discriminación: Otro	0	No	3.204
			1	Sí	1.098
559	s19_1	Dónde fue discriminado: En su familia	0	No	3.750
			1	Sí	552
560	s19_2	Dónde fue discriminado: En un centro de salud	0	No	3.108
			1	Sí	1.194
561	s19_3	Dónde fue discriminado: En su barrio o vecindario	0	No	3.839
			1	Sí	463
562	s19_4	Dónde fue discriminado: En su trabajo	0	No	3.164
			1	Sí	1.138
563	s19_5	Dónde fue discriminado: Buscando trabajo	0	No	4.070
			1	Sí	232
564	s19_6	Dónde fue discriminado: En su establecimiento educacional	0	No	4.048
			1	Sí	254
565	s19_7	Dónde fue discriminado: En tribunales de justicia	0	No	4.248
			1	Sí	54
566	s19_8	Dónde fue discriminado: En el transporte público	0	No	3.912
			1	Sí	390
567	s19_9	Dónde fue discriminado: En tiendas o comercio	0	No	3.864
			1	Sí	438
568	s19_10	Dónde fue discriminado: En instituciones de orden y seguridad pública	0	No	4.203
			1	Sí	99
569	s19_11	Dónde fue discriminado: En instituciones públicas	0	No	3.881
			1	Sí	421
570	s19_12	Dónde fue discriminado: En la vía pública	0	No	3.733
			1	Sí	569
571	s19_77	Dónde fue discriminado: Otro	0	No	4.029
			1	Sí	273
572	s20a_1	Últ. 12 meses, participación en org: Junta de vecinos u organización territorial	0	No	26.351
			1	Sí	3.659
573	s20a_2	Últ. 12 meses, participación en org: Club deportivo o recreativo	0	No	27.193
			1	Sí	2.817
574	s20a_3	Últ. 12 meses, participación en org: Organización religiosa o de iglesia	0	No	26.585
			1	Sí	3.425
575	s20a_4	Últ. 12 meses, participación en org: Agrupaciones artísticas o culturales	0	No	29.257
			1	Sí	753
576	s20a_5	Últ. 12 meses, participación en org: Grupos de identidad cultural	0	No	29.662
			1	Sí	348
577	s20a_6	Últ. 12 meses, participación en org: Agrupaciones juveniles o de estudiantes	0	No	29.786
			1	Sí	224
578	s20a_7	Últ. 12 meses, participación en org: Agrupaciones de mujeres	0	No	29.502
			1	Sí	508
579	s20a_8	Últ. 12 meses, participación en org: Agrupaciones de adulto mayor	0	No	29.074
			1	Sí	936
580	s20a_9	Últ. 12 meses, participación en org: Grupos de voluntariado	0	No	29.662
			1	Sí	348
581	s20a_10	Últ. 12 meses, participación en org: Grupos de apoyo o	0	No	29.838

ID	Nombre variable	Etiqueta de variable	Valores	Etiquetas de valores	Frecuencia
		autoayuda en salud	1	Sí	172
582	s20a_11	Últ. 12 meses, participación en org: Agrupación ideológica (partido político)	0	No	29.888
			1	Sí	122
583	s20a_12	Últ. 12 meses, participación en org: Agrupación corporativa	0	No	29.526
			1	Sí	484
584	s20a_13	Últ. 12 meses, participación en org: Centro de padres y apoderados	0	No	28.946
			1	Sí	1.064
585	s20a_14	Últ. 12 meses, participación en org: Agrupación o mov. que defiende causa/ideal	0	No	29.749
			1	Sí	261
586	s20a_77	Últ. 12 meses, participación en org: Otro	0	No	29.617
			1	Sí	393
587	s20a_78	Últ. 12 meses, participación en org: No participa	0	No	11.610
			1	Sí	18.400
588	s20a_88	Últ. 12 meses, participación en org: No sabe/No responde	0	No	29.855
			1	Sí	155
589	s20b_1	¿Usted participa como...? Junta de vecinos u organización territorial	1	Como asistente	1.584
			2	Como socio o integrante	1.728
			3	Como dirigente u organizador	347
590	s20b_2	¿Usted participa como...? Club deportivo o recreativo	1	Como asistente	1.068
			2	Como socio o integrante	1.531
			3	Como dirigente u organizador	218
591	s20b_3	¿Usted participa como...? Organización religiosa o de iglesia	1	Como asistente	2.084
			2	Como socio o integrante	1.096
			3	Como dirigente u organizador	245
592	s20b_4	¿Usted participa como...? Agrupaciones artísticas o culturales	1	Como asistente	246
			2	Como socio o integrante	421
			3	Como dirigente u organizador	86
593	s20b_5	¿Usted participa como...? Grupos de identidad cultural	1	Como asistente	95
			2	Como socio o integrante	200
			3	Como dirigente u organizador	53
594	s20b_6	¿Usted participa como...? Agrupaciones juveniles o de estudiantes	1	Como asistente	101
			2	Como socio o integrante	76
			3	Como dirigente u organizador	47
595	s20b_7	¿Usted participa como...? Agrupaciones de mujeres	1	Como asistente	198
			2	Como socio o integrante	246
			3	Como dirigente u organizador	64
596	s20b_8	¿Usted participa como...? Agrupaciones de adulto mayor	1	Como asistente	341
			2	Como socio o integrante	483
			3	Como dirigente u organizador	112
597	s20b_9	¿Usted participa como...? Grupos de voluntariado	1	Como asistente	109
			2	Como socio o integrante	185
			3	Como dirigente u organizador	54
598	s20b_10	¿Usted participa como...? Grupos de apoyo o autoayuda en salud	1	Como asistente	72
			2	Como socio o integrante	78
			3	Como dirigente u organizador	22
599	s20b_11	¿Usted participa como...? Agrupación ideológica (partido político)	1	Como asistente	38
			2	Como socio o integrante	69
			3	Como dirigente u organizador	15
600	s20b_12	¿Usted participa como...? Agrupación corporativa	1	Como asistente	89
			2	Como socio o integrante	294
			3	Como dirigente u organizador	101
601	s20b_13	¿Usted participa como...? Centro de padres y apoderados	1	Como asistente	605
			2	Como socio o integrante	268
			3	Como dirigente u organizador	191
602	s20b_14	¿Usted participa como...? Agrupación o mov. que defiende causa/ideal	1	Como asistente	98
			2	Como socio o integrante	123
			3	Como dirigente u organizador	40
603	s20b_77	¿Usted participa como...? Otro	1	Como asistente	156
			2	Como socio o integrante	180
			3	Como dirigente u organizador	57
604	s21	Últ. 12 meses, ¿qué tan frecuente ha participado en esa(s) organización(es)?	-88	No sabe/No responde	24
			1	Rara vez	1.146
			2	Ocasionalmente	3.263

ID	Nombre variable	Etiqueta de variable	Valores	Etiquetas de valores	Frecuencia
			3	Frecuentemente	4.786
			4	Muy frecuentemente	2.236
605	s22	Principal razón por la que no participa en ninguna organización	-88	No sabe/No responde	120
			1	No tiene tiempo	7.331
			2	No le interesa o considera que no sirve para nada	5.437
			3	No sabe cómo participar o no conoce una organización que le interese	745
			4	No tiene quién lo acompañe	192
			5	Cuida o ayuda a cuidar de otras personas	352
			6	Falta de accesibilidad en los lugares	166
			7	Ha tenido problemas de salud	1.716
			8	Falta de recursos económicos	60
			9	No existen organizaciones de su interés en su territorio	463
			10	No le agrada cómo funcionan las organizaciones de su interés	338
			11	No ha podido participar por pandemia COVID-19 o producto de restricciones sanitarias	858
			77	Otra razón	622
606	s23_1	¿Conoce o sabe de qué tratan...? Ley 20.422 sobre igualdad de oportunidades PcD	0	No	21.156
			1	Sí	8.854
607	s23_2	¿Conoce o sabe de qué tratan...? Ley 21.015 incentiva inclusión PcD al trabajo	0	No	21.305
			1	Sí	8.705
608	s23_3	¿Conoce o sabe de qué tratan...? Convención internacional sobre derechos de PcD	0	No	25.454
			1	Sí	4.556
609	s23_4	¿Conoce o sabe de qué tratan...? Convención interamericana DDHH personas mayores	0	No	25.966
			1	Sí	4.044
610	s23_5	¿Conoce o sabe de qué tratan...? Ley 20.609 o Ley Zamudio	0	No	13.644
			1	Sí	16.366
611	s23_6	¿Conoce o sabe de qué tratan...? Ley 21.013 que establece nuevo delito maltrato	0	No	24.293
			1	Sí	5.717
612	s23_78	¿Conoce o sabe de qué tratan...? No, ninguna	0	No	18.235
			1	Sí	11.775
613	s24	¿Votó en las elecciones de la segunda vuelta presidencial 19 dic. 2021?	1	Sí	19.963
			2	No	10.047
614	s25a_1	Votó - Dificultades para votar: Razones de salud	0	No	19.850
			1	Sí	113
615	s25a_2	Votó - Dificultades para votar: Llegar a los lugares de votación	0	No	19.520
			1	Sí	443
616	s25a_3	Votó - Dificultades para votar: Acceso a la mesa o en lugar de votación	0	No	19.881
			1	Sí	82
617	s25a_4	Votó - Dificultades para votar: Falta de apoyo del personal en el lugar	0	No	19.902
			1	Sí	61
618	s25a_5	Votó - Dificultades para votar: Falta de una persona que lo acompañara	0	No	19.943
			1	Sí	20
619	s25a_6	Votó - Dificultades para votar: Aglomeraciones en el local de votación	0	No	19.755
			1	Sí	208
620	s25a_7	Votó - Dificultades para votar: Falta de ajustes y elementos para voto asistido	0	No	19.933
			1	Sí	30
621	s25a_77	Votó - Dificultades para votar: Otra	0	No	19.849
			1	Sí	114
622	s25a_78	Votó - Dificultades para votar: No, ninguna	0	No	923
			1	Sí	19.040
623	s25b	No votó. ¿Enfrentó alguna dificultad que le impidiera ejercer su derecho?	1	Sí	1.802
			2	No	8.245
624	s26	¿Se encuentra inscrito en el Registro Nacional de la Discapacidad?	1	Sí	1.434
			2	No	28.576
625	s27a_1	Último mes: Asistir a reuniones sociales o familiares	0	No	13.908

ID	Nombre variable	Etiqueta de variable	Valores	Etiquetas de valores	Frecuencia
			1	Sí	16.102
626	s27a_2	Último mes: Ir al cine, museo, teatro, concierto, danza, arte, etc.	0	No	24.889
			1	Sí	5.121
627	s27a_3	Último mes: Ver un evento deportivo, ir al estadio u otro recinto	0	No	26.034
			1	Sí	3.976
628	s27a_4	Último mes: Hobbies como tocar un instrumento musical, pintar, dibujar, bailar	0	No	25.227
			1	Sí	4.783
629	s27a_5	Último mes: Jugar juegos de mesa, cartas o videojuegos	0	No	24.146
			1	Sí	5.864
630	s27a_6	Último mes: Practicar algún deporte o hacer ejercicio físico	0	No	24.592
			1	Sí	5.418
631	s27a_7	Último mes: Realizar actividades al aire libre	0	No	20.679
			1	Sí	9.331
632	s27a_8	Último mes: Salir de compras o vitrinear fuera de casa	0	No	12.890
			1	Sí	17.120
633	s27a_9	Último mes: Ir a restaurantes, bares, discotecas, pubs o salir de fiesta	0	No	21.607
			1	Sí	8.403
634	s27a_78	Último mes: Ninguna	0	No	23.997
			1	Sí	6.013
635	s27b_1	Últimos 12 meses: Asistir a celebraciones cívicas o religiosas	0	No	18.256
			1	Sí	11.754
636	s27b_2	Últimos 12 meses: Salir de vacaciones o tomarse vacaciones	0	No	19.296
			1	Sí	10.714
637	s27b_78	Últimos 12 meses: Ninguna	0	No	17.673
			1	Sí	12.337
638	s28	Principal razón por la cual no realizó o asistió a alguna actividad	1	Falta de infraestructura o espacios para la realización de las actividades	70
			2	No tiene información suficiente	199
			3	Inexistencia de profesionales capacitados o equipamiento para apoyar las actividades	9
			4	Imposibilidad debido a la pandemia COVID-19	1.704
			5	No tiene quién lo acompañe o lo asista	187
			6	Porque su salud no se lo permite	1.917
			7	No le interesa o no le motiva	1.919
			8	No tuvo tiempo	3.515
			9	No tiene lugares donde hacerlo	220
			10	No le alcanza el dinero para hacerlo	3.076
			77	Otra	1.075
639	s29	¿Con qué frecuencia realizó actividad física por 30 min. o más?	1	3 o más veces por semana	4.074
			2	1 a 2 veces por semana	4.109
			3	Menos de 4 veces en el mes	1.200
			4	1 vez al mes	1.100
			5	No realizó actividad física el último mes	19.527
640	s30	Principal razón para no haber practicado más actividad física	1	Falta de infraestructura o espacios para la realización de las actividades	290
			2	No tiene información suficiente	183
			3	Inexistencia de profesionales capacitados o equipamiento para apoyar las actividades	70
			4	Imposibilidad debido a la pandemia COVID-19	603
			5	No tiene quién lo acompañe o lo asista	285
			6	Porque su salud no se lo permite	5.744
			7	No le interesa o no le motiva	5.089
			8	No tuvo tiempo	7.901

ID	*Nombre variable*	*Etiqueta de variable*	*Valores*	*Etiquetas de valores*	*Frecuencia*
			9	No tiene lugares donde hacerlo	369
			10	No le alcanza el dinero para hacerlo	494
			77	Otra	799
641	s31a	¿Con qué frecuencia utiliza o consume…? Diarios, libros o revistas	1	No utiliza o consume	12.166
			2	Pocas veces a la semana (1 a 2 veces)	7.867
			3	Pocas veces a la semana (1 a 2 veces)	4.334
			4	Pocas veces al día (1 a 2 veces)	2.669
			5	Varias veces al día (3 veces o más)	2.974
642	s31b	¿Con qué frecuencia utiliza o consume…? Programas de tv, películas, series, etc.	1	No utiliza o consume	1.818
			2	Pocas veces a la semana (1 a 2 veces)	4.967
			3	Pocas veces a la semana (1 a 2 veces)	6.204
			4	Pocas veces al día (1 a 2 veces)	8.307
			5	Varias veces al día (3 veces o más)	8.714
643	s31c	¿Con qué frecuencia utiliza o consume…? Radio u otros medios de audio	1	No utiliza o consume	5.324
			2	Pocas veces a la semana (1 a 2 veces)	4.285
			3	Pocas veces a la semana (1 a 2 veces)	4.831
			4	Pocas veces al día (1 a 2 veces)	5.373
			5	Varias veces al día (3 veces o más)	10.197
644	s31d	¿Con qué frecuencia utiliza o consume…? Computador o dispositivos para navegar	1	No utiliza o consume	8.666
			2	Pocas veces a la semana (1 a 2 veces)	2.565
			3	Pocas veces a la semana (1 a 2 veces)	3.349
			4	Pocas veces al día (1 a 2 veces)	4.018
			5	Varias veces al día (3 veces o más)	11.412
645	s31e	¿Con qué frecuencia utiliza o consume…? Redes sociales	1	No utiliza o consume	7.799
			2	Pocas veces a la semana (1 a 2 veces)	2.357
			3	Pocas veces a la semana (1 a 2 veces)	2.697
			4	Pocas veces al día (1 a 2 veces)	4.763
			5	Varias veces al día (3 veces o más)	12.394
646	s32_1	Dificultades de acceso: Falta de infraestructura o servicios para su uso	0	No	29.210
			1	Sí	800
647	s32_2	Dificultades de acceso: No sabe utilizarlos o no tiene información suficiente	0	No	26.195
			1	Sí	3.815
648	s32_3	Dificultades de acceso: Ausencia de personas que lo asistan para usar y acceder	0	No	29.818
			1	Sí	192
649	s32_4	Dificultades de acceso: No dispone del dispositivo o equipamiento adecuado	0	No	29.530
			1	Sí	480
650	s32_5	Dificultades de acceso: Falta de accesibilidad de los medios que dificultan uso	0	No	29.936
			1	Sí	74
651	s32_6	Dificultades de acceso: Debido a su salud, tiene limitado el uso de estos medios	0	No	29.484
			1	Sí	526
652	s32_7	Dificultades de acceso: No tuvo tiempo	0	No	28.903
			1	Sí	1.107
653	s32_8	Dificultades de acceso: No alcanza el dinero para adquirir servicio/dispositivo	0	No	29.499
			1	Sí	511
654	s32_77	Dificultades de acceso: Otra	0	No	29.544
			1	Sí	466
655	s32_78	Dificultades de acceso: Ningún problema o dificultad	0	No	7.047

ID	*Nombre variable*	*Etiqueta de variable*	*Valores*	*Etiquetas de valores*	*Frecuencia*
			1	Sí	22.963
656	s33a	Últ. 12 meses, ¿Producto del COVID-19…? Perdido su trabajo o fuente de ingresos	-97	No aplica	2.492
			1	Sí	5.491
			2	No	22.027
657	s33b	Últ. 12 meses, ¿Producto del COVID-19…? Disminuido su ingreso o de su hogar	1	Sí	14.159
			2	No	15.851
658	s33c	Últ. 12 meses, ¿Producto del COVID-19…? Suspendido consultas médicas, dentales	-97	No aplica	450
			1	Sí	10.200
			2	No	19.360
659	s33d	Últ. 12 meses, ¿Producto del COVID-19…? Suspendido o postergado tratamientos	-97	No aplica	1.284
			1	Sí	3.878
			2	No	24.848
660	s33e	Últ. 12 meses, ¿Producto del COVID-19…? Suspendido participación en org.	-97	No aplica	2.614
			1	Sí	8.123
			2	No	19.273
661	s34a	Últ. 12 meses, ¿en qué medida COVID-19 afectó…? Relación personas con que vive	1	Muy negativamente	540
			2	Negativamente	4.553
			3	No le ha afectado	20.655
			4	Positivamente	3.456
			5	Muy positivamente	806
662	s34b	Últ. 12 meses, ¿en qué medida COVID-19 afectó…? Relación personas externas	1	Muy negativamente	668
			2	Negativamente	7.177
			3	No le ha afectado	19.586
			4	Positivamente	2.214
			5	Muy positivamente	365
663	s34c	Últ. 12 meses, ¿en qué medida COVID-19 afectó…? Actividades que realiza	1	Muy negativamente	1.102
			2	Negativamente	10.059
			3	No le ha afectado	16.982
			4	Positivamente	1.603
			5	Muy positivamente	264
664	s34d	Últ. 12 meses, ¿en qué medida COVID-19 afectó…? Salud física	1	Muy negativamente	1.234
			2	Negativamente	8.492
			3	No le ha afectado	18.792
			4	Positivamente	1.270
			5	Muy positivamente	222
665	s34e	Últ. 12 meses, ¿en qué medida COVID-19 afectó…? Salud mental	1	Muy negativamente	1.835
			2	Negativamente	9.734
			3	No le ha afectado	17.025
			4	Positivamente	1.183
			5	Muy positivamente	233
666	filtro_adulto_mayor	Adulto mayor que entrega respuestas por sí mismo(a)	1	Sí	8.391
			2	No	488
667	am1	¿Se siente básicamente satisfecho con su vida?	1	Sí	7.515
			2	No	876
668	am2	¿Se aburre con frecuencia?	1	Sí	2.389
			2	No	6.002
669	am3	¿Se siente inútil frecuentemente?	1	Sí	1.767
			2	No	6.624
670	am4	¿Se siente frecuentemente ansioso o angustiado?	1	Sí	2.988
			2	No	5.403
671	am5	¿Se siente frecuentemente desvalido o que no vale nada?	1	Sí	1.332
			2	No	7.059
672	am6	Frase que mejor representa la opinión de las personas que conoce sobre la vejez	-88	No sabe	519
			1	En general expresan opiniones negativas sobre el envejecimiento y la vejez	1.058
			2	En general evitan pensar o hablar sobre el envejecimiento y la vejez	1.144
			3	En general asumen la vejez y el envejecimiento como un proceso natural que tiene aspectos tanto positivos como negativos	4.563

ID	Nombre variable	Etiqueta de variable	Valores	Etiquetas de valores	Frecuencia
			4	En general expresan opiniones positivas sobre el envejecimiento y la vejez	1.107
673	am7	¿La imagen que proyectan los medios de comunicación sobre ser adulto mayor es…?	-99	No responde	89
			-88	No sabe	445
			1	Principalmente positiva	2.298
			2	Ni positiva ni negativa	3.323
			3	Principalmente negativa	2.236
674	am8	¿La imagen de Chile sobre las personas mayores a lo largo del tiempo …?	-99	No responde	61
			-88	No sabe	316
			1	Ha ido empeorando	2.512
			2	Se ha mantenido igual	2.483
			3	Ha ido mejorando	3.019
675	forma_ent_adulto_fin	Forma de finalización del cuestionario adulto	1	Respondió la encuesta por sí mismo(a)	28.167
			2	Respondió la encuesta con ayuda de otra persona	1.366
			3	Respondió la encuesta con ayuda del sistema Vi-Sor	0
			4	Otra persona respondió la encuesta en su nombre	477
676	enc_inicia_cuidador	Encuestador inicia el cuestionario cuidador del adulto seleccionado	1	Sí	1.931
677	selec_cuidador	Identificación del cuidador principal del hogar del adulto seleccionado	1	Sí	1.931
678	edad_cuidador	Edad del cuidador principal del hogar del adulto seleccionado	Rango: 13 - 99		
679	forma_ent_cuidador_inicio	Forma de inicio del cuestionario cuidador del adulto seleccionado	1	Responderá la encuesta por sí mismo(	1.888
			2	Responderá la encuesta con ayuda de	43
680	cui1a	¿Ud. u otra persona ayuda a [NOM. ADULTO]...? Moverse	1	Sí, solo Ud. le ayuda	966
			2	Sí, Ud. y otra persona le ayudan	593
			3	Sí, otra persona le ayuda	18
			4	No, nadie le ayuda	354
681	cui1b	¿Ud. u otra persona ayuda a [NOM. ADULTO]...? Cambiar o mantener la posición	1	Sí, solo Ud. le ayuda	640
			2	Sí, Ud. y otra persona le ayudan	265
			3	Sí, otra persona le ayuda	15
			4	No, nadie le ayuda	1.011
682	cui1c	¿Ud. u otra persona ayuda a [NOM. ADULTO]...? Mirar o escuchar	1	Sí, solo Ud. le ayuda	873
			2	Sí, Ud. y otra persona le ayudan	386
			3	Sí, otra persona le ayuda	22
			4	No, nadie le ayuda	650
683	cui1d	¿Ud. u otra persona ayuda a [NOM. ADULTO]...? Recordar cosas o concentrarse	1	Sí, solo Ud. le ayuda	1.018
			2	Sí, Ud. y otra persona le ayudan	400
			3	Sí, otra persona le ayuda	13
			4	No, nadie le ayuda	500
684	cui1e	¿Ud. u otra persona ayuda a [NOM. ADULTO]...? Realizar activ. de autocuidado	1	Sí, solo Ud. le ayuda	812
			2	Sí, Ud. y otra persona le ayudan	325
			3	Sí, otra persona le ayuda	26
			4	No, nadie le ayuda	768
685	cui1f	¿Ud. u otra persona ayuda a [NOM. ADULTO]...? Hacer las tareas de la casa	1	Sí, solo Ud. le ayuda	1.092
			2	Sí, Ud. y otra persona le ayudan	434
			3	Sí, otra persona le ayuda	24
			4	No, nadie le ayuda	381
686	cui1g	¿Ud. u otra persona ayuda a [NOM. ADULTO]...? Relacionarse con gente cercana	1	Sí, solo Ud. le ayuda	676
			2	Sí, Ud. y otra persona le ayudan	297
			3	Sí, otra persona le ayuda	11
			4	No, nadie le ayuda	947
687	cui1h	¿Ud. u otra persona ayuda a [NOM. ADULTO]...? Participar en fiestas	1	Sí, solo Ud. le ayuda	772
			2	Sí, Ud. y otra persona le ayudan	329
			3	Sí, otra persona le ayuda	21
			4	No, nadie le ayuda	809
688	cui1i	¿Ud. u otra persona ayuda a [NOM. ADULTO]...? Manejar sus emociones	1	Sí, solo Ud. le ayuda	1.016
			2	Sí, Ud. y otra persona le ayudan	416

ID	Nombre variable	Etiqueta de variable	Valores	Etiquetas de valores	Frecuencia
			3	Sí, otra persona le ayuda	25
			4	No, nadie le ayuda	474
689	cui1j	¿Ud. u otra persona ayuda a [NOM. ADULTO]...? Manejar sus conductas	1	Sí, solo Ud. le ayuda	732
			2	Sí, Ud. y otra persona le ayudan	244
			3	Sí, otra persona le ayuda	14
			4	No, nadie le ayuda	941
690	cui1k	¿Ud. u otra persona ayuda a [NOM. ADULTO]...? Comunicarse con los demás	1	Sí, solo Ud. le ayuda	685
			2	Sí, Ud. y otra persona le ayudan	277
			3	Sí, otra persona le ayuda	12
			4	No, nadie le ayuda	957
691	cui1l	¿Ud. u otra persona ayuda a [NOM. ADULTO]...? Utilizar dispositivos y medios	1	Sí, solo Ud. le ayuda	768
			2	Sí, Ud. y otra persona le ayudan	344
			3	Sí, otra persona le ayuda	44
			4	No, nadie le ayuda	775
692	cui1m	¿Ud. u otra persona ayuda a [NOM. ADULTO]...? Encargarse de asuntos económicos	1	Sí, solo Ud. le ayuda	912
			2	Sí, Ud. y otra persona le ayudan	266
			3	Sí, otra persona le ayuda	74
			4	No, nadie le ayuda	679
693	cui2a	Grado de dificultad ayudar a [NOM. ADULTO]: Moverse	1	Ninguna dificultad	657
			2	Dificultad leve	252
			3	Dificultad media	279
			4	Dificultad alta	216
			5	Dificultad muy alta	155
694	cui2b	Grado de dificultad ayudar a [NOM. ADULTO]: Cambiar o mantener la posición	1	Ninguna dificultad	374
			2	Dificultad leve	170
			3	Dificultad media	164
			4	Dificultad alta	129
			5	Dificultad muy alta	68
695	cui2c	Grado de dificultad ayudar a [NOM. ADULTO]: Mirar o escuchar	1	Ninguna dificultad	651
			2	Dificultad leve	257
			3	Dificultad media	187
			4	Dificultad alta	105
			5	Dificultad muy alta	59
696	cui2d	Grado de dificultad ayudar a [NOM. ADULTO]: Recordar cosas o concentrarse	1	Ninguna dificultad	709
			2	Dificultad leve	325
			3	Dificultad media	209
			4	Dificultad alta	112
			5	Dificultad muy alta	63
697	cui2e	Grado de dificultad ayudar a [NOM. ADULTO]: Realizar actividades de autocuidado	1	Ninguna dificultad	549
			2	Dificultad leve	191
			3	Dificultad media	176
			4	Dificultad alta	142
			5	Dificultad muy alta	79
698	cui2f	Grado de dificultad ayudar a [NOM. ADULTO]: Hacer las tareas de la casa	1	Ninguna dificultad	799
			2	Dificultad leve	247
			3	Dificultad media	214
			4	Dificultad alta	133
			5	Dificultad muy alta	133
699	cui2g	Grado de dificultad ayudar a [NOM. ADULTO]: Relacionarse con gente cercana	1	Ninguna dificultad	577
			2	Dificultad leve	161
			3	Dificultad media	126
			4	Dificultad alta	73
			5	Dificultad muy alta	36
700	cui2h	Grado de dificultad ayudar a [NOM. ADULTO]: Participar en fiestas	1	Ninguna dificultad	602
			2	Dificultad leve	168
			3	Dificultad media	127
			4	Dificultad alta	109
			5	Dificultad muy alta	95
701	cui2i	Grado de dificultad ayudar a [NOM. ADULTO]: Manejar sus emociones	1	Ninguna dificultad	650
			2	Dificultad leve	325
			3	Dificultad media	268
			4	Dificultad alta	134
			5	Dificultad muy alta	55

ID	*Nombre variable*	*Etiqueta de variable*	*Valores*	*Etiquetas de valores*	*Frecuencia*
702	cui2j	Grado de dificultad ayudar a [NOM. ADULTO]: Manejar sus conductas	1	Ninguna dificultad	490
			2	Dificultad leve	184
			3	Dificultad media	160
			4	Dificultad alta	91
			5	Dificultad muy alta	51
703	cui2k	Grado de dificultad ayudar a [NOM. ADULTO]: Comunicarse con los demás	1	Ninguna dificultad	545
			2	Dificultad leve	165
			3	Dificultad media	123
			4	Dificultad alta	79
			5	Dificultad muy alta	50
704	cui2l	Grado de dificultad ayudar a [NOM. ADULTO]: Utilizar dispositivos y medios	1	Ninguna dificultad	631
			2	Dificultad leve	145
			3	Dificultad media	141
			4	Dificultad alta	100
			5	Dificultad muy alta	95
705	cui2m	Grado de dificultad ayudar a [NOM. ADULTO]: Encargarse de asuntos económicos	1	Ninguna dificultad	725
			2	Dificultad leve	146
			3	Dificultad media	121
			4	Dificultad alta	99
			5	Dificultad muy alta	87
706	cui3a_1	Quién realiza o con quién. Moverse: Familiares que viven en el hogar	0	No	124
			1	Sí	487
707	cui3a_2	Quién realiza o con quién. Moverse: Familiares que no viven en el hogar	0	No	411
			1	Sí	200
708	cui3a_3	Quién realiza o con quién. Moverse: Amigos	0	No	595
			1	Sí	16
709	cui3a_4	Quién realiza o con quién. Moverse: Conocidos, vecinos	0	No	593
			1	Sí	18
710	cui3a_5	Quién realiza o con quién. Moverse: Cuidador y personal de ayuda	0	No	584
			1	Sí	27
711	cui3a_6	Quién realiza o con quién. Moverse: Profesionales de la salud	0	No	602
			1	Sí	9
712	cui3a_7	Quién realiza o con quién. Moverse: Otros profesionales	0	No	609
			1	Sí	2
713	cui3a_8	Quién realiza o con quién. Moverse: Otro	0	No	609
			1	Sí	2
714	cui3a_9	Quién realiza o con quién. Moverse: No comparte	0	No	591
			1	Sí	20
715	cui3b_1	Quién realiza o con quién. Cambiar/mantener posición: Fam. que viven en el hogar	0	No	64
			1	Sí	216
716	cui3b_2	Quién realiza o con quién. Cambiar/mantener posición: Fam. que no viven en hogar	0	No	215
			1	Sí	65
717	cui3b_3	Quién realiza o con quién. Cambiar/mantener posición: Amigos	0	No	277
			1	Sí	3
718	cui3b_4	Quién realiza o con quién. Cambiar/mantener posición: Conocidos, vecinos	0	No	276
			1	Sí	4
719	cui3b_5	Quién realiza o con quién. Cambiar/mantener posición: Cuidador/personal de ayuda	0	No	256
			1	Sí	24
720	cui3b_6	Quién realiza o con quién. Cambiar/mantener posición: Profesionales de la salud	0	No	278
			1	Sí	2
721	cui3b_7	Quién realiza o con quién. Cambiar/mantener posición: Otros profesionales	0	No	280
722	cui3b_8	Quién realiza o con quién. Cambiar/mantener posición: Otro	0	No	280
723	cui3b_9	Quién realiza o con quién. Cambiar/mantener posición: No comparte	0	No	262
			1	Sí	18
724	cui3c_1	Quién realiza o con quién. Mirar o escuchar: Fam. que viven en el hogar	0	No	61
			1	Sí	347
725	cui3c_2	Quién realiza o con quién. Mirar o escuchar: Fam. que no viven en hogar	0	No	313
			1	Sí	95
726	cui3c_3	Quién realiza o con quién. Mirar o escuchar: Amigos	0	No	400
			1	Sí	8
727	cui3c_4	Quién realiza o con quién. Mirar o escuchar: Conocidos,	0	No	400

ID	Nombre variable	Etiqueta de variable	Valores	Etiquetas de valores	Frecuencia
		vecinos	1	Sí	8
728	cui3c_5	Quién realiza o con quién. Mirar o escuchar: Cuidador/personal de ayuda	0	No	390
			1	Sí	18
729	cui3c_6	Quién realiza o con quién. Mirar o escuchar: Profesionales de la salud	0	No	403
			1	Sí	5
730	cui3c_7	Quién realiza o con quién. Mirar o escuchar: Otros profesionales	0	No	408
731	cui3c_8	Quién realiza o con quién. Mirar o escuchar: Otro	0	No	407
			1	Sí	1
732	cui3c_9	Quién realiza o con quién. Mirar o escuchar: No comparte	0	No	394
			1	Sí	14
733	cui3d_1	Quién realiza o con quién. Recordar o concentrarse: Fam. que viven en el hogar	0	No	68
			1	Sí	345
734	cui3d_2	Quién realiza o con quién. Recordar o concentrarse: Fam. que no viven en hogar	0	No	323
			1	Sí	90
735	cui3d_3	Quién realiza o con quién. Recordar o concentrarse: Amigos	0	No	406
			1	Sí	7
736	cui3d_4	Quién realiza o con quién. Recordar o concentrarse: Conocidos, vecinos	0	No	409
			1	Sí	4
737	cui3d_5	Quién realiza o con quién. Recordar o concentrarse: Cuidador/personal de ayuda	0	No	391
			1	Sí	22
738	cui3d_6	Quién realiza o con quién. Recordar o concentrarse: Profesionales de la salud	0	No	408
			1	Sí	5
739	cui3d_7	Quién realiza o con quién. Recordar o concentrarse: Otros profesionales	0	No	413
740	cui3d_8	Quién realiza o con quién. Recordar o concentrarse: Otro	0	No	410
			1	Sí	3
741	cui3d_9	Quién realiza o con quién. Recordar o concentrarse: No comparte	0	No	402
			1	Sí	11
742	cui3e_1	Quién realiza o con quién. Actividades autocuidado: Fam. que viven en el hogar	0	No	67
			1	Sí	284
743	cui3e_2	Quién realiza o con quién. Actividades autocuidado: Fam. que no viven en hogar	0	No	269
			1	Sí	82
744	cui3e_3	Quién realiza o con quién. Actividades autocuidado: Amigos	0	No	348
			1	Sí	3
745	cui3e_4	Quién realiza o con quién. Actividades autocuidado: Conocidos, vecinos	0	No	347
			1	Sí	4
746	cui3e_5	Quién realiza o con quién. Actividades autocuidado: Cuidador/personal de ayuda	0	No	327
			1	Sí	24
747	cui3e_6	Quién realiza o con quién. Actividades autocuidado: Profesionales de la salud	0	No	350
			1	Sí	1
748	cui3e_7	Quién realiza o con quién. Actividades autocuidado: Otros profesionales	0	No	351
749	cui3e_8	Quién realiza o con quién. Actividades autocuidado: Otro	0	No	350
			1	Sí	1
750	cui3e_9	Quién realiza o con quién. Actividades autocuidado: No comparte	0	No	331
			1	Sí	20
751	cui3f_1	Quién realiza o con quién. Hacer tareas de la casa: Fam. que viven en el hogar	0	No	61
			1	Sí	397
752	cui3f_2	Quién realiza o con quién. Hacer tareas de la casa: Fam. que no viven en hogar	0	No	365
			1	Sí	93
753	cui3f_3	Quién realiza o con quién. Hacer tareas de la casa: Amigos	0	No	457
			1	Sí	1
754	cui3f_4	Quién realiza o con quién. Hacer tareas de la casa: Conocidos, vecinos	0	No	453
			1	Sí	5
755	cui3f_5	Quién realiza o con quién. Hacer tareas de la casa: Cuidador/personal de ayuda	0	No	435
			1	Sí	23
756	cui3f_6	Quién realiza o con quién. Hacer tareas de la casa: Profesionales de la salud	0	No	457
			1	Sí	1
757	cui3f_7	Quién realiza o con quién. Hacer tareas de la casa: Otros profesionales	0	No	458
758	cui3f_8	Quién realiza o con quién. Hacer tareas de la casa: Otro	0	No	455
			1	Sí	3

ID	*Nombre variable*	*Etiqueta de variable*	*Valores*	*Etiquetas de valores*	*Frecuencia*
759	cui3f_9	Quién realiza o con quién. Hacer tareas de la casa: No comparte	0	No	447
			1	Sí	11
760	cui3g_1	Quién realiza o con quién. Relac. con gente cercana: Fam. que viven en el hogar	0	No	61
			1	Sí	247
761	cui3g_2	Quién realiza o con quién. Relac. con gente cercana: Fam. que no viven en hogar	0	No	216
			1	Sí	92
762	cui3g_3	Quién realiza o con quién. Relac. con gente cercana: Amigos	0	No	298
			1	Sí	10
763	cui3g_4	Quién realiza o con quién. Relac. con gente cercana: Conocidos, vecinos	0	No	301
			1	Sí	7
764	cui3g_5	Quién realiza o con quién. Relac. con gente cercana: Cuidador/personal de ayuda	0	No	294
			1	Sí	14
765	cui3g_6	Quién realiza o con quién. Relac. con gente cercana: Profesionales de la salud	0	No	305
			1	Sí	3
766	cui3g_7	Quién realiza o con quién. Relac. con gente cercana: Otros profesionales	0	No	308
767	cui3g_8	Quién realiza o con quién. Relac. con gente cercana: Otro	0	No	305
			1	Sí	3
768	cui3g_9	Quién realiza o con quién. Relac. con gente cercana: No comparte	0	No	294
			1	Sí	14
769	cui3h_1	Quién realiza o con quién. Participar en actividades: Fam. que viven en el hogar	0	No	91
			1	Sí	259
770	cui3h_2	Quién realiza o con quién. Participar en actividades: Fam. que no viven en hogar	0	No	247
			1	Sí	103
771	cui3h_3	Quién realiza o con quién. Participar en actividades: Amigos	0	No	340
			1	Sí	10
772	cui3h_4	Quién realiza o con quién. Participar en actividades: Conocidos, vecinos	0	No	341
			1	Sí	9
773	cui3h_5	Quién realiza o con quién. Participar en actividades: Cuidador/personal de ayuda	0	No	345
			1	Sí	5
774	cui3h_6	Quién realiza o con quién. Participar en actividades: Profesionales de la salud	0	No	350
775	cui3h_7	Quién realiza o con quién. Participar en actividades: Otros profesionales	0	No	350
776	cui3h_8	Quién realiza o con quién. Participar en actividades: Otro	0	No	348
			1	Sí	2
777	cui3h_9	Quién realiza o con quién. Participar en actividades: No comparte	0	No	310
			1	Sí	40
778	cui3i_1	Quién realiza o con quién. Manejar sus emociones: Fam. que viven en el hogar	0	No	93
			1	Sí	348
779	cui3i_2	Quién realiza o con quién. Manejar sus emociones: Fam. que no viven en hogar	0	No	307
			1	Sí	134
780	cui3i_3	Quién realiza o con quién. Manejar sus emociones: Amigos	0	No	429
			1	Sí	12
781	cui3i_4	Quién realiza o con quién. Manejar sus emociones: Conocidos, vecinos	0	No	433
			1	Sí	8
782	cui3i_5	Quién realiza o con quién. Manejar sus emociones: Cuidador/personal de ayuda	0	No	425
			1	Sí	16
783	cui3i_6	Quién realiza o con quién. Manejar sus emociones: Profesionales de la salud	0	No	425
			1	Sí	16
784	cui3i_7	Quién realiza o con quién. Manejar sus emociones: Otros profesionales	0	No	441
785	cui3i_8	Quién realiza o con quién. Manejar sus emociones: Otro	0	No	439
			1	Sí	2
786	cui3i_9	Quién realiza o con quién. Manejar sus emociones: No comparte	0	No	427
			1	Sí	14
787	cui3j_1	Quién realiza o con quién. Manejar sus conductas: Fam. que viven en el hogar	0	No	51
			1	Sí	207
788	cui3j_2	Quién realiza o con quién. Manejar sus conductas: Fam. que no viven en hogar	0	No	188
			1	Sí	70
789	cui3j_3	Quién realiza o con quién. Manejar sus conductas: Amigos	0	No	256
			1	Sí	2
790	cui3j_4	Quién realiza o con quién. Manejar sus conductas: Conocidos,	0	No	257

ID	Nombre variable	Etiqueta de variable	Valores	Etiquetas de valores	Frecuencia
		vecinos	1	Sí	1
791	cui3j_5	Quién realiza o con quién. Manejar sus conductas: Cuidador/personal de ayuda	0	No	245
			1	Sí	13
792	cui3j_6	Quién realiza o con quién. Manejar sus conductas: Profesionales de la salud	0	No	244
			1	Sí	14
793	cui3j_7	Quién realiza o con quién. Manejar sus conductas: Otros profesionales	0	No	256
			1	Sí	2
794	cui3j_8	Quién realiza o con quién. Manejar sus conductas: Otro	0	No	256
			1	Sí	2
795	cui3j_9	Quién realiza o con quién. Manejar sus conductas: No comparte	0	No	246
			1	Sí	12
796	cui3k_1	Quién realiza o con quién. Comunicarse con los demás: Fam. que viven en el hogar	0	No	49
			1	Sí	240
797	cui3k_2	Quién realiza o con quién. Comunicarse con los demás: Fam. que no viven en hogar	0	No	214
			1	Sí	75
798	cui3k_3	Quién realiza o con quién. Comunicarse con los demás: Amigos	0	No	282
			1	Sí	7
799	cui3k_4	Quién realiza o con quién. Comunicarse con los demás: Conocidos, vecinos	0	No	284
			1	Sí	5
800	cui3k_5	Quién realiza o con quién. Comunicarse con los demás: Cuidador/personal de ayuda	0	No	279
			1	Sí	10
801	cui3k_6	Quién realiza o con quién. Comunicarse con los demás: Profesionales de la salud	0	No	287
			1	Sí	2
802	cui3k_7	Quién realiza o con quién. Comunicarse con los demás: Otros profesionales	0	No	289
803	cui3k_8	Quién realiza o con quién. Comunicarse con los demás: Otro	0	No	286
			1	Sí	3
804	cui3k_9	Quién realiza o con quién. Comunicarse con los demás: No comparte	0	No	277
			1	Sí	12
805	cui3l_1	Quién realiza o con quién. Usar dispositivos/medios: Fam. que viven en el hogar	0	No	84
			1	Sí	304
806	cui3l_2	Quién realiza o con quién. Usar dispositivos/medios: Fam. que no viven en hogar	0	No	285
			1	Sí	103
807	cui3l_3	Quién realiza o con quién. Usar dispositivos/medios: Amigos	0	No	381
			1	Sí	7
808	cui3l_4	Quién realiza o con quién. Usar dispositivos/medios: Conocidos, vecinos	0	No	383
			1	Sí	5
809	cui3l_5	Quién realiza o con quién. Usar dispositivos/medios: Cuidador/personal de ayuda	0	No	378
			1	Sí	10
810	cui3l_6	Quién realiza o con quién. Usar dispositivos/medios: Profesionales de la salud	0	No	384
			1	Sí	4
811	cui3l_7	Quién realiza o con quién. Usar dispositivos/medios: Otros profesionales	0	No	388
812	cui3l_8	Quién realiza o con quién. Usar dispositivos/medios: Otro	0	No	386
			1	Sí	2
813	cui3l_9	Quién realiza o con quién. Usar dispositivos/medios: No comparte	0	No	360
			1	Sí	28
814	cui3m_1	Quién realiza o con quién. Enc. asuntos económicos: Fam. que viven en el hogar	0	No	76
			1	Sí	264
815	cui3m_2	Quién realiza o con quién. Enc. asuntos económicos: Fam. que no viven en hogar	0	No	232
			1	Sí	108
816	cui3m_3	Quién realiza o con quién. Enc. asuntos económicos: Amigos	0	No	339
			1	Sí	1
817	cui3m_4	Quién realiza o con quién. Enc. asuntos económicos: Conocidos, vecinos	0	No	339
			1	Sí	1
818	cui3m_5	Quién realiza o con quién. Enc. asuntos económicos: Cuidador/personal de ayuda	0	No	338
			1	Sí	2
819	cui3m_6	Quién realiza o con quién. Enc. asuntos económicos: Profesionales de la salud	0	No	340
820	cui3m_7	Quién realiza o con quién. Enc. asuntos económicos: Otros profesionales	0	No	340
821	cui3m_8	Quién realiza o con quién. Enc. asuntos económicos: Otro	0	No	337
			1	Sí	3

ID	Nombre variable	Etiqueta de variable	Valores	Etiquetas de valores	Frecuencia
822	cui3m_9	Quién realiza o con quién. Enc. asuntos económicos: No comparte	0	No	331
			1	Sí	9
823	cui4	Semana habitual de lunes a domingo, número de días que ayuda a [NOM. ADULTO]	Rango: 1 - 7		
824	cui5	Semana habitual de lunes a viernes, horas diarias dedicadas a [NOM. ADULTO]	Rango: 0 - 24		
825	cui6	Fin de semana habitual, sábado-domingo, horas diarias dedicadas a [NOM. ADULTO]	Rango: 0 - 18		
826	cui7	Años que lleva ayudando o asistiendo a [NOM. ADULTO]	-88	No recuerda	100
			0	0. Menos de un año	134
			Válidos		1.697
827	cui8	En los últimos 12 meses, debido al COVID-19, ¿la ayuda que presta...?	1	Ha disminuido mucho	12
			2	Ha disminuido poco	62
			3	Se ha mantenido igual	1.133
			4	Ha aumentado poco	388
			5	Ha aumentado mucho	336
828	cui9	¿Hace cuantos años fue la última vez que descansó de las labores de asistencia?	-88	No recuerda	665
			1	Menos de un año	550
			2	1 a 2 años	217
			3	3 a 4 años	171
			4	5 años o más	328
829	cui10_1	Último mes. ¿Realizó actividad..? Visitar o compartir con amigos o familiares	0	No	918
			1	Sí	1.013
830	cui10_2	Último mes. ¿Realizó actividad..? Participar en organizaciones sociales	0	No	1.554
			1	Sí	377
831	cui10_3	Último mes. ¿Realizó actividad..? Practicar deporte	0	No	1.712
			1	Sí	219
832	cui10_4	Último mes. ¿Realizó actividad..? Ir al cine, teatro o museo	0	No	1.757
			1	Sí	174
833	cui10_5	Último mes. ¿Realizó actividad..? Leer un libro, diario o revista	0	No	1.510
			1	Sí	421
834	cui10_6	Último mes. ¿Realizó actividad..? Ver una película o serie	0	No	995
			1	Sí	936
835	cui10_77	Último mes. ¿Realizó actividad..? Otra	0	No	1.825
			1	Sí	106
836	cui10_78	Último mes. ¿Realizó actividad..? No tiene tiempo libre	0	No	1.471
			1	Sí	460
837	cui11a	¿Con qué frecuencia…? Piensa que no tiene suficiente tiempo para usted	-99	No responde	5
			-88	No sabe	8
			1	Nunca	830
			2	Rara vez	330
			3	Algunas veces	382
			4	Muchas veces	179
			5	Casi siempre	197
838	cui11b	¿Con qué frecuencia…? Se siente agobiado por intentar compatibilizar el cuidado	-99	No responde	3
			-88	No sabe	3
			1	Nunca	977
			2	Rara vez	216
			3	Algunas veces	389
			4	Muchas veces	198
			5	Casi siempre	145
839	cui11c	¿Con qué frecuencia…? Piensa que cuidar afecta neg. relación con otros miembros	-99	No responde	6
			-88	No sabe	4
			1	Nunca	1.411
			2	Rara vez	152
			3	Algunas veces	193
			4	Muchas veces	91
			5	Casi siempre	74
840	cui11d	¿Con qué frecuencia…? Piensa que su salud ha empeorado debido a cuidar	-99	No responde	3
			-88	No sabe	2
			1	Nunca	1.272
			2	Rara vez	166
			3	Algunas veces	237

ID	Nombre variable	Etiqueta de variable	Valores	Etiquetas de valores	Frecuencia
			4	Muchas veces	141
			5	Casi siempre	110
841	cui11e	¿Con qué frecuencia…? Se siente tenso cuando está cerca de [NOM. ADULTO]	-99	No responde	2
			1	Nunca	1.340
			2	Rara vez	203
			3	Algunas veces	228
			4	Muchas veces	84
			5	Casi siempre	74
842	cui11f	¿Con qué frecuencia…? Siente que ha perdido control de su vida desde que cuida	-99	No responde	5
			-88	No sabe	2
			1	Nunca	1.342
			2	Rara vez	165
			3	Algunas veces	193
			4	Muchas veces	117
			5	Casi siempre	107
843	cui11g	¿Con qué frecuencia…? En general, se siente muy sobrecargado al tener que cuidar	-99	No responde	3
			-88	No sabe	2
			1	Nunca	1.045
			2	Rara vez	251
			3	Algunas veces	315
			4	Muchas veces	167
			5	Casi siempre	148
844	cui12a	Últ. 2 semanas, ¿con qué frecuencia ha sentido…? Poco interés en hacer cosas	1	Nunca	1.215
			2	Varios días	490
			3	Más de la mitad de los días	138
			4	Casi todos los días	88
845	cui12b	Últ. 2 semanas, ¿con qué frecuencia ha sentido…? Decaído, deprimido	1	Nunca	1.072
			2	Varios días	582
			3	Más de la mitad de los días	173
			4	Casi todos los días	104
846	cui12c	Últ. 2 semanas, ¿con qué frecuencia ha sentido…? Nervioso, ansioso	1	Nunca	935
			2	Varios días	680
			3	Más de la mitad de los días	194
			4	Casi todos los días	122
847	cui12d	Últ. 2 semanas, ¿con qué frecuencia ha sentido…? No ha controlado preocupación	1	Nunca	1.280
			2	Varios días	420
			3	Más de la mitad de los días	126
			4	Casi todos los días	105
848	cui13	¿Cuántas personas son cercanas que podría contar con ellas si tuviera problemas?	1	Ninguna	212
			2	Una o dos personas	941
			3	De tres a cinco personas	584
			4	Seis personas o más	194
849	cui14	¿Cuánto interés muestra la gente de su entorno por lo que le pasa o hace?	1	Ningún interés	234
			2	Poco interés	288
			3	Algo de interés	400
			4	Bastante interés	647
			5	Mucho interés	362
850	cui15	¿Cuán fácil sería para Ud. obtener ayuda de los vecinos si la necesitara?	1	Muy difícil	228
			2	Difícil	401
			3	Posible	591
			4	Fácil	526
			5	Muy fácil	185
851	cui16a	Apoyo para cuidado: Asistencia técnica o profesional en domicilio para cuidado	-99	No responde	12
			-88	No sabe	50
			1	Sí	1.057
			2	No	812
852	cui16b	Apoyo para cuidado: Apoyo o relevo de otra persona	-99	No responde	8
			-88	No sabe	30
			1	Sí	794
			2	No	1.099
853	cui16c	Apoyo para cuidado: Compartir responsabilidades de cuidado con otras personas	-99	No responde	7
			-88	No sabe	19
			1	Sí	964

ID	*Nombre variable*	*Etiqueta de variable*	*Valores*	*Etiquetas de valores*	*Frecuencia*
			2	No	941
854	cui16d	Apoyo para cuidado: Entrenamiento técnico o capacitación en cuidados específicos	-99	No responde	7
			-88	No sabe	38
			1	Sí	721
			2	No	1.165
855	cui16e	Apoyo para cuidado: Apoyo psicosocial o atención en salud para usted	-99	No responde	9
			-88	No sabe	22
			1	Sí	888
			2	No	1.012
856	cui16f	Apoyo para cuidado: Acceder a oferta de cuidado en centros día o rehabilitación	-99	No responde	12
			-88	No sabe	51
			1	Sí	524
			2	No	1.344
857	cui16g	Apoyo para cuidado: Acceder a oferta de cuidado o servicio de residencias	-99	No responde	13
			-88	No sabe	56
			1	Sí	279
			2	No	1.583
858	cui16h	Apoyo para cuidado: Apoyo y asistencia de la comunidad	-99	No responde	13
			-88	No sabe	56
			1	Sí	407
			2	No	1.455
859	cui16i	Apoyo para cuidado: Apoyo o asistencia en las tareas del hogar	-99	No responde	7
			-88	No sabe	20
			1	Sí	554
			2	No	1.350
860	cui17	¿Usted participa o tiene contacto con organizaciones de cuidadores?	1	Sí	79
			2	No	1.852
861	cui18	¿Alguna vez ha recibido capacitación para realizar labores de cuidado?	1	Sí	146
			2	No	1.785
862	cui19_1	¿Quién o qué institución realizó la capacitación? Institución pública	0	No	70
			1	Sí	76
863	cui19_2	¿Quién o qué institución realizó la capacitación? Una ONG	0	No	143
			1	Sí	3
864	cui19_3	¿Quién o qué institución realizó la capacitación? Otro tipo de institución	0	No	102
			1	Sí	44
865	cui19_4	¿Quién o qué institución realizó la capacitación? Un profesional de salud	0	No	124
			1	Sí	22
866	cui19_88	¿Quién o qué institución realizó la capacitación? No sabe	0	No	140
			1	Sí	6
867	cui20	¿Qué tanto le ha servido la capacitación que recibió? Escala de 1 a 7	1	No le ha servido para nada	1
			2	2	1
			3	3	3
			4	4	10
			5	5	17
			6	6	18
			7	Le ha servido mucho	96
868	forma_ent_cuidador_fin	Forma de finalización del cuestionario cuidador del adulto seleccionado	1	Respondió la encuesta por sí mismo(a	1.871
			2	Respondió la encuesta con ayuda de o	60
869	rp2	Parentesco del responsable principal con el NNA seleccionado	1	Madre	4.268
			2	Padre	583
			3	Madrastra	18
			4	Padrastro	21
			5	Hermano(a)	58
			6	Abuelo(a)	470
			7	Otro familiar	107
			8	No familiar	1
870	forma_ent_nna_rp_inicio	Forma de inicio del cuestionario NNA - responsable principal	1	Responderá la encuesta por sí mismo(	5.338
			2	Responderá la encuesta con ayuda de	188
			3	Responderá la encuesta con ayuda del	0
871	n1	¿Qué tan problemático ha sido…? Caminar	-99	No responde	2
			-88	No sabe	2
			1	Nada problemático	5.241
			2	Levemente problemático	183

ID	*Nombre variable*	*Etiqueta de variable*	*Valores*	*Etiquetas de valores*	*Frecuencia*
			3	Moderadamente problemático	62
			4	Severamente problemático	28
			5	Extremadamente problemático o no p	8
872	n2	¿Qué tan problemático ha sido…? Manipular objetos pequeños o abrir un envase	-99	No responde	1
			-88	No sabe	1
			1	Nada problemático	5.170
			2	Levemente problemático	248
			3	Moderadamente problemático	74
			4	Severamente problemático	17
			5	Extremadamente problemático o no p	15
873	n3	¿Qué tan problemático ha sido…? Ver cosas de lejos	-99	No responde	0
			-88	No sabe	8
			1	Nada problemático	4.259
			2	Levemente problemático	688
			3	Moderadamente problemático	384
			4	Severamente problemático	142
			5	Extremadamente problemático o no p	45
874	n4	¿Qué tan problemático ha sido…? Oír	-99	No responde	0
			-88	No sabe	5
			1	Nada problemático	5.258
			2	Levemente problemático	186
			3	Moderadamente problemático	58
			4	Severamente problemático	17
			5	Extremadamente problemático o no p	2
875	n5	¿Qué tan problemático ha sido…? Asearse o vestirse	-99	No responde	1
			-88	No sabe	0
			1	Nada problemático	4.518
			2	Levemente problemático	165
			3	Moderadamente problemático	57
			4	Severamente problemático	21
			5	Extremadamente problemático o no p	14
876	n6	¿Qué tan problemático ha sido…? Entender lo que usted le dice	-99	No responde	0
			-88	No sabe	2
			1	Nada problemático	653
			2	Levemente problemático	68
			3	Moderadamente problemático	23
			4	Severamente problemático	4
			5	Extremadamente problemático o no p	0
877	n7	¿Qué tan problemático ha sido…? Entender lo que [NNA SELEC] quiere	-99	No responde	0
			-88	No sabe	0
			1	Nada problemático	548
			2	Levemente problemático	150
			3	Moderadamente problemático	38
			4	Severamente problemático	11
			5	Extremadamente problemático o no p	3
878	n8	¿Qué tan problemático ha sido…? Entender a otra gente	-99	No responde	0
			-88	No sabe	0
			1	Nada problemático	4.118
			2	Levemente problemático	434
			3	Moderadamente problemático	163
			4	Severamente problemático	51
			5	Extremadamente problemático o no p	10
879	n9	¿Qué tan problemático ha sido…? Ser entendido	-99	No responde	0
			-88	No sabe	0
			1	Nada problemático	3.911
			2	Levemente problemático	545
			3	Moderadamente problemático	219
			4	Severamente problemático	73
			5	Extremadamente problemático o no p	28
880	n10	¿Qué tan problemático ha sido…? Aprender nombres de objetos cotidianos	-99	No responde	0
			-88	No sabe	0
			1	Nada problemático	614
			2	Levemente problemático	93

ID	Nombre variable	Etiqueta de variable	Valores	Etiquetas de valores	Frecuencia
			3	Moderadamente problemático	26
			4	Severamente problemático	11
			5	Extremadamente problemático o no p	6
881	n11	¿Qué tan problemático ha sido…? Aprender a hacer cosas nuevas	-99	No responde	0
			-88	No sabe	1
			1	Nada problemático	4.082
			2	Levemente problemático	486
			3	Moderadamente problemático	149
			4	Severamente problemático	45
			5	Extremadamente problemático o no p	13
882	n12	¿Qué tan problemático ha sido…? Completar una tarea	-99	No responde	0
			-88	No sabe	1
			1	Nada problemático	3.720
			2	Levemente problemático	719
			3	Moderadamente problemático	238
			4	Severamente problemático	67
			5	Extremadamente problemático o no p	31
883	n13	¿Qué tan problemático ha sido…? Hacer cambios de planes o cambiar su rutina	-99	No responde	2
			-88	No sabe	5
			1	Nada problemático	3.673
			2	Levemente problemático	705
			3	Moderadamente problemático	264
			4	Severamente problemático	83
			5	Extremadamente problemático o no p	44
884	n14	¿Qué tan problemático ha sido…? Hacer las tareas	-99	No responde	3
			-88	No sabe	6
			1	Nada problemático	3.608
			2	Levemente problemático	743
			3	Moderadamente problemático	294
			4	Severamente problemático	91
			5	Extremadamente problemático o no p	31
885	n15	¿Qué tan problemático ha sido…? Jugar con juguetes u objetos de uso doméstico	-99	No responde	0
			-88	No sabe	0
			1	Nada problemático	720
			2	Levemente problemático	21
			3	Moderadamente problemático	7
			4	Severamente problemático	1
			5	Extremadamente problemático o no p	1
886	n16	¿Qué tan problemático ha sido…? Jugar con otros niños y niñas	-99	No responde	0
			-88	No sabe	0
			1	Nada problemático	620
			2	Levemente problemático	89
			3	Moderadamente problemático	32
			4	Severamente problemático	4
			5	Extremadamente problemático o no p	5
887	n17	¿Qué tan problemático ha sido…? Jugar o hacer actividades con niños y niñas	-99	No responde	2
			-88	No sabe	3
			1	Nada problemático	4.192
			2	Levemente problemático	342
			3	Moderadamente problemático	140
			4	Severamente problemático	63
			5	Extremadamente problemático o no p	34
888	n18	¿Qué tan problemático ha sido…? Participar en actividades en comunidad	-99	No responde	23
			-88	No sabe	99
			1	Nada problemático	4.083
			2	Levemente problemático	303
			3	Moderadamente problemático	149
			4	Severamente problemático	64
			5	Extremadamente problemático o no p	55
889	n19	¿Qué tan problemático ha sido…? Llevarse bien con niños y niñas de su misma edad	-99	No responde	2
			-88	No sabe	4
			1	Nada problemático	4.054
			2	Levemente problemático	462

ID	Nombre variable	Etiqueta de variable	Valores	Etiquetas de valores	Frecuencia
			3	Moderadamente problemático	172
			4	Severamente problemático	57
			5	Extremadamente problemático	25
890	n20	¿Qué tan problemático ha sido…? Haber mordido o golpeado a otros	-99	No responde	4
			-88	No sabe	8
			1	Nada problemático	597
			2	Levemente problemático	104
			3	Moderadamente problemático	25
			4	Severamente problemático	9
			5	Extremadamente problemático	3
891	n21	¿Qué tan problemático ha sido…? Controlar su comportamiento	-99	No responde	1
			-88	No sabe	1
			1	Nada problemático	3.600
			2	Levemente problemático	792
			3	Moderadamente problemático	287
			4	Severamente problemático	69
			5	Extremadamente problemático	26
892	n22	¿Qué tan problemático ha sido…? Sentir algún dolor físico	-99	No responde	1
			-88	No sabe	8
			1	Nada problemático	3.660
			2	Levemente problemático	1.299
			3	Moderadamente problemático	432
			4	Severamente problemático	98
			5	Extremadamente problemático	28
893	n23	¿Qué tan problemático ha sido…? No tener suficiente energía	-99	No responde	2
			-88	No sabe	2
			1	Nada problemático	4.583
			2	Levemente problemático	652
			3	Moderadamente problemático	209
			4	Severamente problemático	55
			5	Extremadamente problemático	23
894	n24	¿Qué tan problemático ha sido…? Tener demasiada energía	-99	No responde	3
			-88	No sabe	8
			1	Nada problemático	4.344
			2	Levemente problemático	701
			3	Moderadamente problemático	349
			4	Severamente problemático	79
			5	Extremadamente problemático	42
895	n25	¿Qué tan problemático ha sido…? Tener dificultad para respirar	-99	No responde	3
			-88	No sabe	2
			1	Nada problemático	4.821
			2	Levemente problemático	452
			3	Moderadamente problemático	192
			4	Severamente problemático	38
			5	Extremadamente problemático	18
896	n26	¿Qué tan problemático ha sido…? Sentirse triste, bajo de ánimo o deprimido	-99	No responde	3
			-88	No sabe	5
			1	Nada problemático	3.906
			2	Levemente problemático	1.079
			3	Moderadamente problemático	388
			4	Severamente problemático	111
			5	Extremadamente problemático	34
897	n27	¿Qué tan problemático ha sido…? Sentir preocupación, nerviosismo o ansiedad	-99	No responde	2
			-88	No sabe	8
			1	Nada problemático	3.431
			2	Levemente problemático	1.339
			3	Moderadamente problemático	534
			4	Severamente problemático	151
			5	Extremadamente problemático	61
898	n28	En los últimos 12 meses, ¿producto del COVID-19 estas dificultades…?	1	Han disminuido mucho	262
			2	Han disminuido poco	518
			3	Se han mantenido igual	2.405
			4	Han aumentado poco	688

ID	Nombre variable	Etiqueta de variable	Valores	Etiquetas de valores	Frecuencia
			5	Han aumentado mucho	241
899	n29_1	Enfermedad o condición de salud: Ceguera	0	No	5.511
			1	Sí	15
900	n29_2	Enfermedad o condición de salud: Pérdida de la visión	0	No	4.785
			1	Sí	741
901	n29_3	Enfermedad o condición de salud: Sordera	0	No	5.513
			1	Sí	13
902	n29_4	Enfermedad o condición de salud: Pérdida de la audición	0	No	5.465
			1	Sí	61
903	n29_5	Enfermedad o condición de salud: Sordoceguera	0	No	5.525
			1	Sí	1
904	n29_6	Enfermedad o condición de salud: Asma o enfermedad alérgica respiratoria	0	No	4.976
			1	Sí	550
905	n29_7	Enfermedad o condición de salud: Enfermedad pulmonar crónica	0	No	5.491
			1	Sí	35
906	n29_8	Enfermedad o condición de salud: COVID-19 o PIMS	0	No	4.879
			1	Sí	647
907	n29_9	Enfermedad o condición de salud: Migraña	0	No	5.264
			1	Sí	262
908	n29_10	Enfermedad o condición de salud: Malnutrición por exceso o déficit	0	No	5.259
			1	Sí	267
909	n29_11	Enfermedad o condición de salud: Trastorno déficit atencional o hiperactividad	0	No	5.191
			1	Sí	335
910	n29_12	Enfermedad o condición de salud: Ansiedad o trastornos de ansiedad	0	No	5.147
			1	Sí	379
911	n29_13	Enfermedad o condición de salud: Depresión o trastornos depresivos	0	No	5.364
			1	Sí	162
912	n29_14	Enfermedad o condición de salud: Problema o trastorno del sueño-vigilia	0	No	5.267
			1	Sí	259
913	n29_15	Enfermedad o condición de salud: Trastorno alimentario	0	No	5.464
			1	Sí	62
914	n29_16	Enfermedad o condición de salud: Trastorno de la conducta	0	No	5.316
			1	Sí	210
915	n29_17	Enfermedad o condición de salud: Trastorno bipolar	0	No	5.489
			1	Sí	37
916	n29_18	Enfermedad o condición de salud: Esquizofrenia u otros trastornos psicóticos	0	No	5.523
			1	Sí	3
917	n29_19	Enfermedad o condición de salud: Autismo (Trastorno del espectro Autista)	0	No	5.363
			1	Sí	163
918	n29_20	Enfermedad o condición de salud: Enfermedad al corazón	0	No	5.471
			1	Sí	55
919	n29_21	Enfermedad o condición de salud: Epilepsia	0	No	5.501
			1	Sí	25
920	n29_22	Enfermedad o condición de salud: Diabetes	0	No	5.500
			1	Sí	26
921	n29_23	Enfermedad o condición de salud: Enfermedad renal crónica	0	No	5.509
			1	Sí	17
922	n29_24	Enfermedad o condición de salud: Escoliosis/condición de deformación de columna	0	No	5.485
			1	Sí	41
923	n29_25	Enfermedad o condición de salud: Meningitis o encefalitis	0	No	5.524
			1	Sí	2
924	n29_26	Enfermedad o condición de salud: Síndrome de Down	0	No	5.507
			1	Sí	19
925	n29_27	Enfermedad o condición de salud: Parálisis cerebral	0	No	5.524
			1	Sí	2
926	n29_28	Enfermedad o condición de salud: Retraso del desarrollo psicomotor	0	No	5.481
			1	Sí	45
927	n29_29	Enfermedad o condición de salud: Defectos congénitos de nacimiento	0	No	5.479
			1	Sí	47
928	n29_30	Enfermedad o condición de salud: Artritis	0	No	5.520
			1	Sí	6
929	n29_31	Enfermedad o condición de salud: Tumor o cáncer	0	No	5.508
			1	Sí	18

ID	*Nombre variable*	*Etiqueta de variable*	*Valores*	*Etiquetas de valores*	*Frecuencia*
930	n29_32	Enfermedad o condición de salud: Enfermedad neuromuscular	0	No	5.521
			1	Sí	5
931	n29_33	Enfermedad o condición de salud: Lesión medular	0	No	5.526
			1	Sí	0
932	n29_34	Enfermedad o condición de salud: Lesiones graves por accidente de tránsito	0	No	5.518
			1	Sí	8
933	n29_35	Enfermedad o condición de salud: Lesiones graves por caída, quemadura, golpes	0	No	5.497
			1	Sí	29
934	n29_36	Enfermedad o condición de salud: Lesiones graves infringidas por otra persona	0	No	5.520
			1	Sí	6
935	n29_37	Enfermedad o condición de salud: Problema o trastorno por consumo de alcohol	0	No	5.525
			1	Sí	1
936	n29_38	Enfermedad o condición de salud: Problema o trastorno por consumo de drogas	0	No	5.522
			1	Sí	4
937	n29_77	Enfermedad o condición de salud: Otro problema de salud crónico	0	No	5.221
			1	Sí	305
938	n29_78	Enfermedad o condición de salud: No tiene ninguna	0	No	2.613
			1	Sí	2.913
939	n29_esp	Especifique. Otro problemas de salud crónico	Válidos		305
940	n29a_1	¿Le ha dicho un médico que tiene…? Ceguera	-99	No responde	0
			1	Sí	12
			2	No	3
941	n29a_2	¿Le ha dicho un médico que tiene…? Pérdida de la visión	-99	No responde	9
			1	Sí	632
			2	No	100
942	n29a_3	¿Le ha dicho un médico que tiene…? Sordera	-99	No responde	0
			1	Sí	11
			2	No	2
943	n29a_4	¿Le ha dicho un médico que tiene…? Pérdida de la audición	-99	No responde	1
			1	Sí	36
			2	No	24
944	n29a_5	¿Le ha dicho un médico que tiene…? Sordoceguera	-99	No responde	0
			1	Sí	0
			2	No	1
945	n29a_6	¿Le ha dicho un médico que tiene…? Asma o enfermedad alérgica respiratoria	-99	No responde	7
			1	Sí	503
			2	No	40
946	n29a_7	¿Le ha dicho un médico que tiene…? Enfermedad pulmonar crónica	-99	No responde	2
			1	Sí	32
			2	No	1
947	n29a_8	¿Le ha dicho un médico que tiene…? COVID-19 o PIMS	-99	No responde	0
			1	Sí	545
			2	No	102
948	n29a_9	¿Le ha dicho un médico que tiene…? Migraña	-99	No responde	0
			1	Sí	148
			2	No	114
949	n29a_10	¿Le ha dicho un médico que tiene…? Malnutrición por exceso o déficit	-99	No responde	4
			1	Sí	226
			2	No	37
950	n29a_11	¿Le ha dicho un médico que tiene…? Trastorno déficit atencional o hiperactividad	-99	No responde	5
			1	Sí	267
			2	No	63
951	n29a_12	¿Le ha dicho un médico que tiene…? Ansiedad o trastornos de ansiedad	-99	No responde	2
			1	Sí	219
			2	No	158
952	n29a_13	¿Le ha dicho un médico que tiene…? Depresión o trastornos depresivos	-99	No responde	0
			1	Sí	119
			2	No	43
953	n29a_14	¿Le ha dicho un médico que tiene…? Problema o trastorno del sueño-vigilia	-99	No responde	0
			1	Sí	160
			2	No	99
954	n29a_15	¿Le ha dicho un médico que tiene…? Trastorno alimentario	-99	No responde	0
			1	Sí	44

ID	*Nombre variable*	*Etiqueta de variable*	*Valores*	*Etiquetas de valores*	*Frecuencia*
			2	No	18
955	n29a_16	¿Le ha dicho un médico que tiene…? Trastorno de la conducta	-99	No responde	2
			1	Sí	119
			2	No	89
956	n29a_17	¿Le ha dicho un médico que tiene…? Trastorno bipolar	-99	No responde	0
			1	Sí	18
			2	No	19
957	n29a_18	¿Le ha dicho un médico que tiene…? Esquizofrenia u otros trastornos psicóticos	-99	No responde	0
			1	Sí	3
			2	No	0
958	n29a_19	¿Le ha dicho un médico que tiene…? Autismo (Trastorno del espectro Autista)	-99	No responde	10
			1	Sí	139
			2	No	14
959	n29a_20	¿Le ha dicho un médico que tiene…? Enfermedad al corazón	-99	No responde	1
			1	Sí	51
			2	No	3
960	n29a_21	¿Le ha dicho un médico que tiene…? Epilepsia	-99	No responde	2
			1	Sí	23
			2	No	0
961	n29a_22	¿Le ha dicho un médico que tiene…? Diabetes	-99	No responde	0
			1	Sí	24
			2	No	2
962	n29a_23	¿Le ha dicho un médico que tiene…? Enfermedad renal crónica	-99	No responde	0
			1	Sí	17
			2	No	0
963	n29a_24	¿Le ha dicho un médico que tiene…? Escoliosis/condición deformación de columna	-99	No responde	5
			1	Sí	30
			2	No	6
964	n29a_25	¿Le ha dicho un médico que tiene…? Meningitis o encefalitis	-99	No responde	0
			1	Sí	2
			2	No	0
965	n29a_26	¿Le ha dicho un médico que tiene…? Síndrome de Down	-99	No responde	0
			1	Sí	19
			2	No	0
966	n29a_27	¿Le ha dicho un médico que tiene…? Parálisis cerebral	-99	No responde	1
			1	Sí	1
			2	No	0
967	n29a_28	¿Le ha dicho un médico que tiene…? Retraso del desarrollo psicomotor	-99	No responde	1
			1	Sí	43
			2	No	1
968	n29a_29	¿Le ha dicho un médico que tiene…? Defectos congénitos de nacimiento	-99	No responde	27
			1	Sí	18
			2	No	2
969	n29a_30	¿Le ha dicho un médico que tiene…? Artritis	-99	No responde	0
			1	Sí	5
			2	No	1
970	n29a_31	¿Le ha dicho un médico que tiene…? Tumor o cáncer	-99	No responde	7
			1	Sí	11
			2	No	0
971	n29a_32	¿Le ha dicho un médico que tiene…? Enfermedad neuromuscular	-99	No responde	0
			1	Sí	5
			2	No	0
972	n29a_33	¿Le ha dicho un médico que tiene…? Lesión medular	-99	No responde	0
			1	Sí	0
			2	No	0
973	n29a_34	¿Le ha dicho un médico que tiene…? Lesiones graves por accidente de tránsito	-99	No responde	0
			1	Sí	7
			2	No	1
974	n29a_35	¿Le ha dicho un médico que tiene…? Lesiones graves por caída, quemadura, golpes	-99	No responde	0
			1	Sí	26
			2	No	3
975	n29a_36	¿Le ha dicho un médico que tiene…? Lesiones graves infringidas por otra persona	-99	No responde	0
			1	Sí	2

ID	Nombre variable	Etiqueta de variable	Valores	Etiquetas de valores	Frecuencia
		[illegible]	2	No	4
976	n29a_37	¿Le ha dicho un médico que tiene…? Problema o trastorno por consumo de alcohol	-99	No responde	0
			1	Sí	1
			2	No	0
977	n29a_38	¿Le ha dicho un médico que tiene…? Problema o trastorno por consumo de drogas	-99	No responde	0
			1	Sí	3
			2	No	1
978	n29a_77	¿Le ha dicho un médico que tiene…? Otro problema de salud crónico	-99	No responde	0
			1	Sí	257
			2	No	136
979	n29b_1	¿Ha recibido medicamento para...? Ceguera	-99	No responde	0
			1	Sí	6
			2	No	9
980	n29b_2	¿Ha recibido medicamento para...? Pérdida de la visión	-99	No responde	9
			1	Sí	145
			2	No	587
981	n29b_3	¿Ha recibido medicamento para...? Sordera	-99	No responde	0
			1	Sí	0
			2	No	13
982	n29b_4	¿Ha recibido medicamento para...? Pérdida de la audición	-99	No responde	1
			1	Sí	11
			2	No	49
983	n29b_5	¿Ha recibido medicamento para...? Sordoceguera	-99	No responde	0
			1	Sí	0
			2	No	1
984	n29b_6	¿Ha recibido medicamento para...? Asma o enfermedad alérgica respiratoria	-99	No responde	7
			1	Sí	461
			2	No	82
985	n29b_7	¿Ha recibido medicamento para...? Enfermedad pulmonar crónica	-99	No responde	2
			1	Sí	28
			2	No	5
986	n29b_8	¿Ha recibido medicamento para...? COVID-19 o PIMS	-99	No responde	0
			1	Sí	379
			2	No	268
987	n29b_9	¿Ha recibido medicamento para...? Migraña	-99	No responde	0
			1	Sí	173
			2	No	89
988	n29b_10	¿Ha recibido medicamento para...? Malnutrición por exceso o déficit	-99	No responde	4
			1	Sí	72
			2	No	191
989	n29b_11	¿Ha recibido medicamento para...? Trastorno déficit atencional o hiperactividad	-99	No responde	5
			1	Sí	129
			2	No	201
990	n29b_12	¿Ha recibido medicamento para...? Ansiedad o trastornos de ansiedad	-99	No responde	2
			1	Sí	107
			2	No	270
991	n29b_13	¿Ha recibido medicamento para...? Depresión o trastornos depresivos	-99	No responde	0
			1	Sí	81
			2	No	81
992	n29b_14	¿Ha recibido medicamento para...? Problema o trastorno del sueño-vigilia	-99	No responde	0
			1	Sí	112
			2	No	147
993	n29b_15	¿Ha recibido medicamento para...? Trastorno alimentario	-99	No responde	0
			1	Sí	18
			2	No	44
994	n29b_16	¿Ha recibido medicamento para...? Trastorno de la conducta	-99	No responde	2
			1	Sí	56
			2	No	152
995	n29b_17	¿Ha recibido medicamento para...? Trastorno bipolar	-99	No responde	0
			1	Sí	12
			2	No	25
996	n29b_18	¿Ha recibido medicamento para...? Esquizofrenia u otros trastornos psicóticos	-99	No responde	0
			1	Sí	3

ID	*Nombre variable*	*Etiqueta de variable*	*Valores*	*Etiquetas de valores*	*Frecuencia*
			2	No	0
997	n29b_19	¿Ha recibido medicamento para...? Autismo (Trastorno del espectro Autista)	-99	No responde	10
			1	Sí	74
			2	No	79
998	n29b_20	¿Ha recibido medicamento para...? Enfermedad al corazón	-99	No responde	1
			1	Sí	18
			2	No	36
999	n29b_21	¿Ha recibido medicamento para...? Epilepsia	-99	No responde	2
			1	Sí	21
			2	No	2
1000	n29b_22	¿Ha recibido medicamento para...? Diabetes	-99	No responde	0
			1	Sí	18
			2	No	8
1001	n29b_23	¿Ha recibido medicamento para...? Enfermedad renal crónica	-99	No responde	0
			1	Sí	11
			2	No	6
1002	n29b_24	¿Ha recibido medicamento para...? Escoliosis/condición deformación de columna	-99	No responde	5
			1	Sí	18
			2	No	18
1003	n29b_25	¿Ha recibido medicamento para...? Meningitis o encefalitis	-99	No responde	0
			1	Sí	1
			2	No	1
1004	n29b_26	¿Ha recibido medicamento para...? Síndrome de Down	-99	No responde	0
			1	Sí	6
			2	No	13
1005	n29b_27	¿Ha recibido medicamento para...? Parálisis cerebral	-99	No responde	1
			1	Sí	1
			2	No	0
1006	n29b_28	¿Ha recibido medicamento para...? Retraso del desarrollo psicomotor	-99	No responde	1
			1	Sí	15
			2	No	29
1007	n29b_29	¿Ha recibido medicamento para...? Defectos congénitos de nacimiento	-99	No responde	27
			1	Sí	7
			2	No	13
1008	n29b_30	¿Ha recibido medicamento para...? Artritis	-99	No responde	0
			1	Sí	6
			2	No	0
1009	n29b_31	¿Ha recibido medicamento para...? Tumor o cáncer	-99	No responde	7
			1	Sí	6
			2	No	5
1010	n29b_32	¿Ha recibido medicamento para...? Enfermedad neuromuscular	-99	No responde	0
			1	Sí	3
			2	No	2
1011	n29b_33	¿Ha recibido medicamento para...? Lesión medular	-99	No responde	0
			1	Sí	0
			2	No	0
1012	n29b_34	¿Ha recibido medicamento para...? Lesiones graves por accidente de tránsito	-99	No responde	0
			1	Sí	3
			2	No	5
1013	n29b_35	¿Ha recibido medicamento para...? Lesiones graves por caída, quemadura, golpes	-99	No responde	0
			1	Sí	18
			2	No	11
1014	n29b_36	¿Ha recibido medicamento para...? Lesiones graves infringidas por otra persona	-99	No responde	0
			1	Sí	2
			2	No	4
1015	n29b_37	¿Ha recibido medicamento para...? Problema o trastorno por consumo de alcohol	-99	No responde	0
			1	Sí	1
			2	No	0
1016	n29b_38	¿Ha recibido medicamento para...? Problema o trastorno por consumo de drogas	-99	No responde	0
			1	Sí	1
			2	No	3
1017	n29b_77	¿Ha recibido medicamento para...? Otro problema de salud crónico	-99	No responde	0
			1	Sí	184

ID	Nombre variable	Etiqueta de variable	Valores	Etiquetas de valores	Frecuencia
			2	No	209
1018	n29c_1	¿Ha recibido otro tratam. para...? Ceguera	-99	No responde	0
			1	Sí	6
			2	No	9
1019	n29c_2	¿Ha recibido otro tratam. para...? Pérdida de la visión	-99	No responde	9
			1	Sí	276
			2	No	456
1020	n29c_3	¿Ha recibido otro tratam. para...? Sordera	-99	No responde	0
			1	Sí	4
			2	No	9
1021	n29c_4	¿Ha recibido otro tratam. para...? Pérdida de la audición	-99	No responde	1
			1	Sí	16
			2	No	44
1022	n29c_5	¿Ha recibido otro tratam. para...? Sordoceguera	-99	No responde	0
			1	Sí	0
			2	No	1
1023	n29c_6	¿Ha recibido otro tratam. para...? Asma o enfermedad alérgica respiratoria	-99	No responde	7
			1	Sí	226
			2	No	317
1024	n29c_7	¿Ha recibido otro tratam. para...? Enfermedad pulmonar crónica	-99	No responde	2
			1	Sí	15
			2	No	18
1025	n29c_8	¿Ha recibido otro tratam. para...? COVID-19 o PIMS	-99	No responde	0
			1	Sí	126
			2	No	521
1026	n29c_9	¿Ha recibido otro tratam. para...? Migraña	-99	No responde	0
			1	Sí	48
			2	No	214
1027	n29c_10	¿Ha recibido otro tratam. para...? Malnutrición por exceso o déficit	-99	No responde	4
			1	Sí	93
			2	No	170
1028	n29c_11	¿Ha recibido otro tratam. para...? Trastorno déficit atencional o hiperactividad	-99	No responde	5
			1	Sí	117
			2	No	213
1029	n29c_12	¿Ha recibido otro tratam. para...? Ansiedad o trastornos de ansiedad	-99	No responde	2
			1	Sí	121
			2	No	256
1030	n29c_13	¿Ha recibido otro tratam. para...? Depresión o trastornos depresivos	-99	No responde	0
			1	Sí	75
			2	No	87
1031	n29c_14	¿Ha recibido otro tratam. para...? Problema o trastorno del sueño-vigilia	-99	No responde	0
			1	Sí	70
			2	No	189
1032	n29c_15	¿Ha recibido otro tratam. para...? Trastorno alimentario	-99	No responde	0
			1	Sí	30
			2	No	32
1033	n29c_16	¿Ha recibido otro tratam. para...? Trastorno de la conducta	-99	No responde	2
			1	Sí	60
			2	No	148
1034	n29c_17	¿Ha recibido otro tratam. para...? Trastorno bipolar	-99	No responde	0
			1	Sí	13
			2	No	24
1035	n29c_18	¿Ha recibido otro tratam. para...? Esquizofrenia u otros trastornos psicóticos	-99	No responde	0
			1	Sí	3
			2	No	0
1036	n29c_19	¿Ha recibido otro tratam. para...? Autismo (Trastorno del espectro Autista)	-99	No responde	10
			1	Sí	85
			2	No	68
1037	n29c_20	¿Ha recibido otro tratam. para...? Enfermedad al corazón	-99	No responde	1
			1	Sí	20
			2	No	34
1038	n29c_21	¿Ha recibido otro tratam. para...? Epilepsia	-99	No responde	2
			1	Sí	10

ID	Nombre variable	Etiqueta de variable	Valores	Etiquetas de valores	Frecuencia
			2	No	13
1039	n29c_22	¿Ha recibido otro tratam. para...? Diabetes	-99	No responde	0
			1	Sí	13
			2	No	13
1040	n29c_23	¿Ha recibido otro tratam. para...? Enfermedad renal crónica	-99	No responde	0
			1	Sí	9
			2	No	8
1041	n29c_24	¿Ha recibido otro tratam. para...? Escoliosis/condición deformación de columna	-99	No responde	5
			1	Sí	13
			2	No	23
1042	n29c_25	¿Ha recibido otro tratam. para...? Meningitis o encefalitis	-99	No responde	0
			1	Sí	1
			2	No	1
1043	n29c_26	¿Ha recibido otro tratam. para...? Síndrome de Down	-99	No responde	0
			1	Sí	9
			2	No	10
1044	n29c_27	¿Ha recibido otro tratam. para...? Parálisis cerebral	-99	No responde	1
			1	Sí	1
			2	No	0
1045	n29c_28	¿Ha recibido otro tratam. para...? Retraso del desarrollo psicomotor	-99	No responde	1
			1	Sí	28
			2	No	16
1046	n29c_29	¿Ha recibido otro tratam. para...? Defectos congénitos de nacimiento	-99	No responde	27
			1	Sí	10
			2	No	10
1047	n29c_30	¿Ha recibido otro tratam. para...? Artritis	-99	No responde	0
			1	Sí	2
			2	No	4
1048	n29c_31	¿Ha recibido otro tratam. para...? Tumor o cáncer	-99	No responde	7
			1	Sí	6
			2	No	5
1049	n29c_32	¿Ha recibido otro tratam. para...? Enfermedad neuromuscular	-99	No responde	0
			1	Sí	4
			2	No	1
1050	n29c_33	¿Ha recibido otro tratam. para...? Lesión medular	-99	No responde	0
			1	Sí	0
			2	No	0
1051	n29c_34	¿Ha recibido otro tratam. para...? Lesiones graves por accidente de tránsito	-99	No responde	0
			1	Sí	4
			2	No	4
1052	n29c_35	¿Ha recibido otro tratam. para...? Lesiones graves por caída, quemadura, golpes	-99	No responde	0
			1	Sí	13
			2	No	16
1053	n29c_36	¿Ha recibido otro tratam. para...? Lesiones graves infringidas por otra persona	-99	No responde	0
			1	Sí	4
			2	No	2
1054	n29c_37	¿Ha recibido otro tratam. para...? Problema o trastorno por consumo de alcohol	-99	No responde	0
			1	Sí	1
			2	No	0
1055	n29c_38	¿Ha recibido otro tratam. para...? Problema o trastorno por consumo de drogas	-99	No responde	0
			1	Sí	2
			2	No	2
1056	n29c_77	¿Ha recibido otro tratam. para...? Otro problema de salud crónico	-99	No responde	0
			1	Sí	141
			2	No	252
1057	n30	¿Qué grado de dificultad ha tenido para…? Ver sin lentes	-99	No responde	9
			-88	No sabe	11
			1	Ninguna	4.087
			2	Leve	695
			3	Moderada	425
			4	Severa	226
			5	Extrema o no puede hacerlo	73
1058	n31	¿Qué grado de dificultad ha tenido para…? Escuchar sin	-99	No responde	6

ID	Nombre variable	Etiqueta de variable	Valores	Etiquetas de valores	Frecuencia
		dispositivo de ayuda	-88	No sabe	3
			1	Ninguna	5.390
			2	Leve	90
			3	Moderada	25
			4	Severa	8
			5	Extrema o no puede hacerlo	4
1059	n32	¿Qué grado de dificultad ha tenido para…? Caminar	-99	No responde	3
			-88	No sabe	1
			1	Ninguna	5.294
			2	Leve	135
			3	Moderada	62
			4	Severa	19
			5	Extrema o no puede hacerlo	12
1060	n33	¿Qué grado de dificultad ha tenido para…? Entenderle a usted y a otros	-99	No responde	2
			-88	No sabe	1
			1	Ninguna	4.936
			2	Leve	386
			3	Moderada	148
			4	Severa	40
			5	Extrema o no puede hacerlo	13
1061	n34	¿Qué grado de dificultad ha tenido para…? Aprender	-99	No responde	1
			-88	No sabe	3
			1	Ninguna	4.528
			2	Leve	599
			3	Moderada	279
			4	Severa	86
			5	Extrema o no puede hacerlo	30
1062	n35	¿Qué grado de dificultad ha tenido para…? Controlar su comportamiento	-99	No responde	0
			-88	No sabe	1
			1	Ninguna	4.225
			2	Leve	840
			3	Moderada	345
			4	Severa	81
			5	Extrema o no puede hacerlo	34
1063	n36	¿Qué grado de dificultad ha tenido para…? Completar una tarea	-99	No responde	4
			-88	No sabe	6
			1	Ninguna	4.340
			2	Leve	774
			3	Moderada	269
			4	Severa	92
			5	Extrema o no puede hacerlo	41
1064	n37	¿Qué grado de dificultad ha tenido para…? Llevarse bien con otros niños y niñas	-99	No responde	1
			-88	No sabe	4
			1	Ninguna	4.785
			2	Leve	469
			3	Moderada	186
			4	Severa	54
			5	Extrema o no puede hacerlo	27
1065	n38_1	Condición permanente y/o larga duración: Dificultad física y/o movilidad	0	No	5.423
			1	Sí	103
1066	n38_2	Condición permanente y/o larga duración: Mudez o dificultad en el habla	0	No	5.378
			1	Sí	148
1067	n38_3	Condición permanente y/o larga duración: Dificultad psiquiátrica	0	No	5.472
			1	Sí	54
1068	n38_4	Condición permanente y/o larga duración: Dificultad mental o intelectual	0	No	5.405
			1	Sí	121
1069	n38_5	Condición permanente y/o larga duración: Dificultad psicosocial	0	No	5.395
			1	Sí	131
1070	n38_6	Condición permanente y/o larga duración: Sordera o dificultad para oír	0	No	5.507
			1	Sí	19
1071	n38_7	Condición permanente y/o larga duración: Ceguera o dificultad para ver	0	No	5.453
			1	Sí	73
1072	n38_8	Condición permanente y/o larga duración: No tiene ninguna de	0	No	512

ID	Nombre variable	Etiqueta de variable	Valores	Etiquetas de valores	Frecuencia
		estas condiciones	1	Sí	5.014
1073	n39	¿En qué medida le hacen fácil o difícil…? El est. educacional su aprendizaje	-99	No responde	6
			-88	No sabe	4
			1	Muy fácil	1.410
			2	Fácil	2.903
			3	Ni fácil ni difícil	736
			4	Difícil	203
			5	Muy difícil	25
1074	n40	¿En qué medida le hacen fácil o difícil…? Los lugares participar en actividades	-99	No responde	9
			-88	No sabe	40
			1	Muy fácil	1.458
			2	Fácil	3.026
			3	Ni fácil ni difícil	653
			4	Difícil	286
			5	Muy difícil	54
1075	n41	¿En qué medida le hacen fácil o difícil…? El transporte público utilizarlo	-99	No responde	87
			-88	No sabe	270
			1	Muy fácil	1.197
			2	Fácil	2.592
			3	Ni fácil ni difícil	764
			4	Difícil	473
			5	Muy difícil	143
1076	n42	¿En qué medida le hacen fácil o difícil…? La vivienda y habitaciones vivir	-99	No responde	2
			-88	No sabe	1
			1	Muy fácil	2.148
			2	Fácil	3.082
			3	Ni fácil ni difícil	196
			4	Difícil	81
			5	Muy difícil	16
1077	n43	¿En qué medida le hacen fácil o difícil…? Otros NNA sentirse respetado	-99	No responde	3
			-88	No sabe	43
			1	Muy fácil	1.498
			2	Fácil	3.129
			3	Ni fácil ni difícil	610
			4	Difícil	215
			5	Muy difícil	28
1078	n44	¿Utiliza dispositivos que le faciliten…? Estar en la escuela	1	Sí	863
			2	No	4.424
1079	n45	¿Utiliza dispositivos que le faciliten…? Estar en su casa	1	Sí	150
			2	No	5.376
1080	n46	¿Utiliza dispositivos que le faciliten…? Participar en actividades fuera de casa	1	Sí	1.047
			2	No	4.479
1081	n47	Debido a su salud, ¿[NOM. NNA] toma medicamentos de manera habitual?	1	Sí	842
			2	No	4.684
1082	n48_1	Ayudas técnicas utilizadas: Ninguna	0	No	1.228
			1	Sí	4.298
1083	n48_2	Ayudas técnicas utilizadas: Anteojos o lentes de contacto para ver	0	No	4.456
			1	Sí	1.070
1084	n48_3	Ayudas técnicas utilizadas: Audífonos	0	No	5.514
			1	Sí	12
1085	n48_4	Ayudas técnicas utilizadas: Muleta o bastón	0	No	5.521
			1	Sí	5
1086	n48_5	Ayudas técnicas utilizadas: Andadores	0	No	5.524
			1	Sí	2
1087	n48_6	Ayudas técnicas utilizadas: Silla de ruedas	0	No	5.518
			1	Sí	8
1088	n48_7	Ayudas técnicas utilizadas: Bastón blanco o bastón guiador	0	No	5.524
			1	Sí	2
1089	n48_8	Ayudas técnicas utilizadas: Calzado ortopédico y/o plantillas	0	No	5.456
			1	Sí	70
1090	n48_9	Ayudas técnicas utilizadas: Cojines y/o colchones antiescaras	0	No	5.522
			1	Sí	4
1091	n48_10	Ayudas técnicas utilizadas: Catre clínico	0	No	5.524

ID	Nombre variable	Etiqueta de variable	Valores	Etiquetas de valores	Frecuencia
			1	Sí	2
1092	n48_11	Ayudas técnicas utilizadas: Órtesis para miembro inferior, superior o columna	0	No	5.524
			1	Sí	2
1093	n48_12	Ayudas técnicas utilizadas: Prótesis de miembro inferior	0	No	5.522
			1	Sí	4
1094	n48_13	Ayudas técnicas utilizadas: Prótesis de miembro superior	0	No	5.525
			1	Sí	1
1095	n48_14	Ayudas técnicas utilizadas: Bipedestador	0	No	5.526
			1	Sí	0
1096	n48_15	Ayudas técnicas utilizadas: Productos para transferencias	0	No	5.525
			1	Sí	1
1097	n48_16	Ayudas técnicas utilizadas: Equipos tecnológicos adaptados	0	No	5.511
			1	Sí	15
1098	n48_17	Ayudas técnicas utilizadas: Teclado o mouse adaptados	0	No	5.526
			1	Sí	0
1099	n48_18	Ayudas técnicas utilizadas: Programas de accesibilidad informática	0	No	5.524
			1	Sí	2
1100	n48_19	Ayudas técnicas utilizadas: Tablero de comunicación	0	No	5.523
			1	Sí	3
1101	n48_20	Ayudas técnicas utilizadas: Lupa	0	No	5.523
			1	Sí	3
1102	n48_21	Ayudas técnicas utilizadas: Equipos para proporcionar oxígeno	0	No	5.522
			1	Sí	4
1103	n48_77	Ayudas técnicas utilizadas: Otros	0	No	5.447
			1	Sí	79
1104	n49_1	Ayudas técnicas adicionales: Ninguna	0	No	222
			1	Sí	1.006
1105	n49_2	Ayudas técnicas adicionales: Anteojos o lentes de contacto para ver	0	No	1.068
			1	Sí	160
1106	n49_3	Ayudas técnicas adicionales: Audífonos	0	No	1.221
			1	Sí	7
1107	n49_4	Ayudas técnicas adicionales: Muleta o bastón	0	No	1.228
			1	Sí	0
1108	n49_5	Ayudas técnicas adicionales: Andadores	0	No	1.226
			1	Sí	2
1109	n49_6	Ayudas técnicas adicionales: Silla de ruedas	0	No	1.226
			1	Sí	2
1110	n49_7	Ayudas técnicas adicionales: Bastón blanco o bastón guiador	0	No	1.228
			1	Sí	0
1111	n49_8	Ayudas técnicas adicionales: Calzado ortopédico y/o plantillas	0	No	1.210
			1	Sí	18
1112	n49_9	Ayudas técnicas adicionales: Cojines y/o colchones antiescaras	0	No	1.227
			1	Sí	1
1113	n49_10	Ayudas técnicas adicionales: Catre clínico	0	No	1.228
			1	Sí	0
1114	n49_11	Ayudas técnicas adicionales: Órtesis para miembro inferior, superior o columna	0	No	1.227
			1	Sí	1
1115	n49_12	Ayudas técnicas adicionales: Prótesis de miembro inferior	0	No	1.228
			1	Sí	0
1116	n49_13	Ayudas técnicas adicionales: Prótesis de miembro superior	0	No	1.227
			1	Sí	1
1117	n49_14	Ayudas técnicas adicionales: Bipedestador	0	No	1.228
			1	Sí	0
1118	n49_15	Ayudas técnicas adicionales: Productos para transferencias	0	No	1.226
			1	Sí	2
1119	n49_16	Ayudas técnicas adicionales: Equipos tecnológicos adaptados	0	No	1.224
			1	Sí	4
1120	n49_17	Ayudas técnicas adicionales: Teclado o mouse adaptados	0	No	1.226
			1	Sí	2
1121	n49_18	Ayudas técnicas adicionales: Programas de accesibilidad informática	0	No	1.226
			1	Sí	2
1122	n49_19	Ayudas técnicas adicionales: Tablero de comunicación	0	No	1.226
			1	Sí	2

ID	Nombre variable	Etiqueta de variable	Valores	Etiquetas de valores	Frecuencia
1123	n49_20	Ayudas técnicas adicionales: Lupa	0	No	1.228
			1	Sí	0
1124	n49_21	Ayudas técnicas adicionales: Equipos para proporcionar oxígeno	0	No	1.227
			1	Sí	1
1125	n49_77	Ayudas técnicas adicionales: Otros	0	No	1.195
			1	Sí	33
1126	n50_1	No utiliza. Ayudas técnicas necesarias: Ninguna	0	No	384
			1	Sí	3.914
1127	n50_2	No utiliza. Ayudas técnicas necesarias: Anteojos o lentes de contacto para ver	0	No	3.960
			1	Sí	338
1128	n50_3	No utiliza. Ayudas técnicas necesarias: Audífonos	0	No	4.280
			1	Sí	18
1129	n50_4	No utiliza. Ayudas técnicas necesarias: Muleta o bastón	0	No	4.297
			1	Sí	1
1130	n50_5	No utiliza. Ayudas técnicas necesarias: Andadores	0	No	4.298
			1	Sí	0
1131	n50_6	No utiliza. Ayudas técnicas necesarias: Silla de ruedas	0	No	4.297
			1	Sí	1
1132	n50_7	No utiliza. Ayudas técnicas necesarias: Bastón blanco o bastón guiador	0	No	4.298
			1	Sí	0
1133	n50_8	No utiliza. Ayudas técnicas necesarias: Calzado ortopédico y/o plantillas	0	No	4.270
			1	Sí	28
1134	n50_9	No utiliza. Ayudas técnicas necesarias: Cojines y/o colchones antiescaras	0	No	4.297
			1	Sí	1
1135	n50_10	No utiliza. Ayudas técnicas necesarias: Catre clínico	0	No	4.298
			1	Sí	0
1136	n50_11	No utiliza. Ayudas técnicas necesarias: Órtesis para miembro inf./sup. o columna	0	No	4.298
			1	Sí	0
1137	n50_12	No utiliza. Ayudas técnicas necesarias: Prótesis de miembro inferior	0	No	4.298
			1	Sí	0
1138	n50_13	No utiliza. Ayudas técnicas necesarias: Prótesis de miembro superior	0	No	4.298
			1	Sí	0
1139	n50_14	No utiliza. Ayudas técnicas necesarias: Bipedestador	0	No	4.298
			1	Sí	0
1140	n50_15	No utiliza. Ayudas técnicas necesarias: Productos para transferencias	0	No	4.298
			1	Sí	0
1141	n50_16	No utiliza. Ayudas técnicas necesarias: Equipos tecnológicos adaptados	0	No	4.294
			1	Sí	4
1142	n50_17	No utiliza. Ayudas técnicas necesarias: Teclado o mouse adaptados	0	No	4.298
			1	Sí	0
1143	n50_18	No utiliza. Ayudas técnicas necesarias: Programas de accesibilidad informática	0	No	4.296
			1	Sí	2
1144	n50_19	No utiliza. Ayudas técnicas necesarias: Tablero de comunicación	0	No	4.296
			1	Sí	2
1145	n50_20	No utiliza. Ayudas técnicas necesarias: Lupa	0	No	4.298
			1	Sí	0
1146	n50_21	No utiliza. Ayudas técnicas necesarias: Equipos para proporcionar oxígeno	0	No	4.298
			1	Sí	0
1147	n50_77	No utiliza. Ayudas técnicas necesarias: Otros	0	No	4.290
			1	Sí	8
1148	n51	¿[NOM. NNA] requiere de una persona que le ayude o asista debido a su salud?	1	Sí	355
			2	No	5.171
1149	n52	¿Con qué frecuencia [NOM. NNA] recibe ayuda o asistencia debido a su salud?	1	Casi nunca	58
			2	Algunas veces	90
			3	Muchas veces	83
			4	Siempre	124
1150	n53_1	¿Recibe asistencia…? Para comer	0	No	286
			1	Sí	69
1151	n53_2	¿Recibe asistencia…? Para bañarse, asearse o arreglarse	0	No	159
			1	Sí	196
1152	n53_3	¿Recibe asistencia…? Para tomar sus medicamentos	0	No	154
			1	Sí	201
1153	n53_4	¿Recibe asistencia…? En terapias de rehabilitación o	0	No	246

ID	Nombre variable	Etiqueta de variable	Valores	Etiquetas de valores	Frecuencia
		tratamiento	1	Sí	109
1154	n53_5	¿Recibe asistencia…? En tareas de aprendizaje	0	No	120
			1	Sí	235
1155	n53_6	¿Recibe asistencia…? Para entender a otros o ser entendido	0	No	217
			1	Sí	138
1156	n53_7	¿Recibe asistencia…? Para mirar	0	No	295
			1	Sí	60
1157	n53_8	¿Recibe asistencia…? Para escuchar	0	No	328
			1	Sí	27
1158	n53_9	¿Recibe asistencia…? Para realizar tareas del hogar	0	No	214
			1	Sí	141
1159	n53_10	¿Recibe asistencia…? Para relacionarse con otras personas, familiares o amigos	0	No	256
			1	Sí	99
1160	n53_11	¿Recibe asistencia…? Para jugar, hacer deporte o aficiones personales	0	No	302
			1	Sí	53
1161	n53_12	¿Recibe asistencia…? Para participar en actividades de la escuela	0	No	285
			1	Sí	70
1162	n53_13	¿Recibe asistencia…? En actividades fuera del hogar	0	No	214
			1	Sí	141
1163	n53_14	¿Recibe asistencia…? Para ir a citas médicas o atenciones en salud	0	No	123
			1	Sí	232
1164	n53_15	¿Recibe asistencia…? Alguien que esté pendiente durante el día y la noche	0	No	227
			1	Sí	128
1165	n53_77	¿Recibe asistencia…? Otra	0	No	339
			1	Sí	16
1166	n54	¿Cree que [NOM. NNA] necesita ayuda adicional a la que recibe?	1	Sí	128
			2	No	227
1167	n55	Identificación del cuidador principal que asiste al NNA (orden persona hogar)	Rango: 1 - 9		
1168	n56	Parentesco del cuidador principal que asiste al NNA	1	Padre	26
			2	Madre	266
			3	Abuelo	3
			4	Abuela	44
			5	Hermano(a), cuñado(a)	8
			6	Otro pariente	7
			7	Vecino(a)	0
			8	Amigo(a)	0
			9	Servicio doméstico, servicios personal	0
			77	Otra	1
1169	n57	¿El cuidador principal que asiste al NNA recibe una remuneración?	1	Sí	13
			2	No	342
1170	n58	¿Alguien más ayuda o asiste a [NOM. NNA] en su vida debido a su salud?	1	Sí, especifique	195
			2	No	160
1171	n59a	Cuidador 2: ¿Reside en el hogar del NNA seleccionado?	1	Sí	162
			2	No	33
1172	n59b	Cuidador 2: Identificación dentro del hogar del NNA entrevistado (orden persona)	Rango: 1 - 8		
1173	n60	Cuidador 2: Edad (cuidador fuera del hogar)	Rango: 22 - 78		
1174	n61	Cuidador 2: Sexo (cuidador fuera del hogar)	1	Hombre	9
			2	Mujer	24
1175	n62	Cuidador 2: Parentesco con el NNA seleccionado	1	Padre	73
			2	Madre	30
			3	Abuelo	8
			4	Abuela	41
			5	Hermano(a), cuñado(a)	20
			6	Otro pariente	11
			7	Vecino(a)	0
			8	Amigo(a)	0
			9	Servicio doméstico, servicios personal	4
			77	Otra	8
1176	n63	Cuidador 2: ¿Recibe remuneración?	1	Sí	10
			2	No	185
1177	n64	¿Cree que el cuidador principal necesita ayuda adicional?	1	Sí	39

ID	Nombre variable	Etiqueta de variable	Valores	Etiquetas de valores	Frecuencia
			2	No	121
1178	n65	Últ. 12 meses, veces que ha recibido atención o consulta de salud	-88	No sabe/No responde	30
			0	Ninguna	1.276
			Válidos		4.220
1179	n66	Últ. 12 meses, est. de salud en que recibió atención de forma más frecuente	-88	No sabe/No recuerda	2
			1	Centro de Salud Familiar CESFAM o co	1.904
			2	Posta Rural (Municipal o SNSS)	98
			3	CRS o CDT (Consultorio de especialida	15
			4	COSAM (Centro de salud mental comu	21
			5	SAPU (Servicio de Atención Primaria d	135
			6	SAMU (Servicio de Atención Médico d	25
			7	Posta (Servicio de urgencia de hospita	63
			8	Hospital público o del SNSS	466
			9	CCR, Centro Comunitario de Rehabilita	5
			10	Consulta, centro médico, clínica u hos	1.298
			11	Centro de salud mental privado	19
			12	Establecimiento de las FF.AA. o del Or	36
			13	Servicio de urgencia de clínica u hospi	84
			14	Mutual de Seguridad	0
			15	Servicio médico de alumnos del lugar	12
			77	Otro	37
1180	n67_1	Últ. 12 meses, profesional que lo atendió más: Médico general	0	No	2.079
			1	Sí	2.141
1181	n67_2	Últ. 12 meses, profesional que lo atendió más: Traumatólogo	0	No	4.049
			1	Sí	171
1182	n67_3	Últ. 12 meses, profesional que lo atendió más: Ginecólogo	0	No	4.155
			1	Sí	65
1183	n67_4	Últ. 12 meses, profesional que lo atendió más: Psiquiatra	0	No	4.115
			1	Sí	105
1184	n67_5	Últ. 12 meses, profesional que lo atendió más: Oftalmólogo	0	No	3.952
			1	Sí	268
1185	n67_6	Últ. 12 meses, profesional que lo atendió más: Neurólogo	0	No	4.024
			1	Sí	196
1186	n67_7	Últ. 12 meses, profesional que lo atendió más: Pediatra	0	No	2.997
			1	Sí	1.223
1187	n67_8	Últ. 12 meses, profesional que lo atendió más: Otro médico especialista	0	No	3.845
			1	Sí	375
1188	n67_9	Últ. 12 meses, profesional que lo atendió más: Enfermero	0	No	3.940
			1	Sí	280
1189	n67_10	Últ. 12 meses, profesional que lo atendió más: Matrona	0	No	4.156
			1	Sí	64
1190	n67_11	Últ. 12 meses, profesional que lo atendió más: Dentista	0	No	3.474
			1	Sí	746
1191	n67_12	Últ. 12 meses, profesional que lo atendió más: Kinesiólogo	0	No	4.105
			1	Sí	115
1192	n67_13	Últ. 12 meses, profesional que lo atendió más: Psicólogo	0	No	3.931
			1	Sí	289
1193	n67_14	Últ. 12 meses, profesional que lo atendió más: Nutricionista	0	No	4.013
			1	Sí	207
1194	n67_15	Últ. 12 meses, profesional que lo atendió más: Tecnólogo médico	0	No	4.201
			1	Sí	19
1195	n67_16	Últ. 12 meses, profesional que lo atendió más: Fonoaudiólogo	0	No	4.112
			1	Sí	108
1196	n67_17	Últ. 12 meses, profesional que lo atendió más: Terapeuta ocupacional	0	No	4.149
			1	Sí	71
1197	n67_18	Últ. 12 meses, profesional que lo atendió más: Especialista en med. alternativa	0	No	4.207
			1	Sí	13
1198	n67_77	Últ. 12 meses, profesional que lo atendió más: Otro	0	No	4.052
			1	Sí	168
1199	n67_88	Últ. 12 meses, profesional que lo atendió más: No sabe/No responde	0	No	4.219
			1	Sí	1
1200	n68a	Ult. 12 meses, ¿se le presentó…? Problemas para llegar a la consulta	1	Sí	234
			2	No	3.986

ID	Nombre variable	Etiqueta de variable	Valores	Etiquetas de valores	Frecuencia
1201	n68b	Ult. 12 meses, ¿se le presentó…? Problemas para conseguir cita o atención	1	Sí	1.015
			2	No	3.205
1202	n68c	Ult. 12 meses, ¿se le presentó…? Problemas para ser atendido	1	Sí	919
			2	No	3.301
1203	n68d	Ult. 12 meses, ¿se le presentó…? Problemas para pagar por la atención	1	Sí	346
			2	No	3.874
1204	n68e	Ult. 12 meses, ¿se le presentó…? Problemas para entrega o acceso a medicamentos	1	Sí	401
			2	No	3.819
1205	n69_1	Razón de no atención: No había disponibilidad del profesional requerido	0	No	1.237
			1	Sí	39
1206	n69_2	Razón de no atención: No podía pagar el costo de la atención	0	No	1.269
			1	Sí	7
1207	n69_3	Razón de no atención: Le suspendieron la atención debido al COVID-19	0	No	1.250
			1	Sí	26
1208	n69_4	Razón de no atención: Decidió no asistir debido al COVID-19	0	No	1.244
			1	Sí	32
1209	n69_5	Razón de no atención: Intentó conseguir asistencia, pero se la negaron	0	No	1.269
			1	Sí	7
1210	n69_6	Razón de no atención: Lo trataron mal o con neglicencia y decidió no asistir	0	No	1.274
			1	Sí	2
1211	n69_7	Razón de no atención: No le pareció confiable o adecuada la atención	0	No	1.272
			1	Sí	4
1212	n69_8	Razón de no atención: Tuvo problemas para llegar al establecimiento de salud	0	No	1.270
			1	Sí	6
1213	n69_9	Razón de no atención: No podía ausentarse del trabajo u otros compromisos	0	No	1.274
			1	Sí	2
1214	n69_10	Razón de no atención: No lo consideró necesario	0	No	161
			1	Sí	1.115
1215	n69_77	Razón de no atención: Otra razón	0	No	1.210
			1	Sí	66
1216	n70	Últ. 12 meses, número de veces que ha recibido atención de rehabilitación	-88	No sabe/No responde	45
			0	0. Ninguna	5.173
			Válidos		308
1217	n71	Últ. 12 meses, est de salud en que recibió rehabilitación de forma más frecuente	-88	No sabe/No responde	1
			1	Centro de Salud Familiar CESFAM o co	78
			2	Posta Rural (Municipal o SNSS)	0
			3	CRS o CDT (Consultorio de especialida	2
			4	COSAM (Centro de salud mental comu	7
			5	SAPU (Servicio de Atención Primaria d	1
			6	SAMU (Servicio de Atención Médico d	0
			7	Posta (servicio de urgencia de hospita	5
			8	Hospital público o del SNSS	42
			9	CCR, Centro Comunitario de Rehabilita	19
			10	Consulta, centro médico, clínica u hos	91
			11	Centro de salud mental privado	2
			12	Establecimiento de las FF.AA. o del Or	4
			13	Servicio de urgencia de clínica privada	2
			14	Mutual de Seguridad	1
			15	Servicio médico de alumnos del lugar	17
			16	En su domicilio	11
			77	Otro	25
1218	n72_1	Últ. 12 meses, prof. de rehabilitación que lo atendió más: Traumatólogo	0	No	277
			1	Sí	31
1219	n72_2	Últ. 12 meses, prof. de rehabilitación que lo atendió más: Ginecólogo	0	No	304
			1	Sí	4
1220	n72_3	Últ. 12 meses, prof. de rehabilitación que lo atendió más: Psiquiatra	0	No	290
			1	Sí	18
1221	n72_4	Últ. 12 meses, prof. de rehabilitación que lo atendió más: Oftalmólogo	0	No	301
			1	Sí	7
1222	n72_5	Últ. 12 meses, prof. de rehabilitación que lo atendió más: Neurólogo	0	No	284
			1	Sí	24
1223	n72_6	Últ. 12 meses, prof. de rehabilitación que lo atendió más: Pediatra	0	No	290
			1	Sí	18

ID	Nombre variable	Etiqueta de variable	Valores	Etiquetas de valores	Frecuencia
1224	n72_7	Últ. 12 meses, prof. de rehabilitación que lo atendió más: Kinesiólogo	0	No	176
			1	Sí	132
1225	n72_8	Últ. 12 meses, prof. de rehabilitación que lo atendió más: Psicólogo	0	No	234
			1	Sí	74
1226	n72_9	Últ. 12 meses, prof. de rehabilitación que lo atendió más: Fonoaudiólogo	0	No	258
			1	Sí	50
1227	n72_10	Últ. 12 meses, prof. de rehabilitación que lo atendió más: Terapeuta ocupacional	0	No	251
			1	Sí	57
1228	n72_11	Últ. 12 meses, prof. de rehabilitación que lo atendió más: Especialista med. alt	0	No	304
			1	Sí	4
1229	n72_77	Últ. 12 meses, prof. de rehabilitación que lo atendió más: Otro	0	No	266
			1	Sí	42
1230	n72_88	Últ. 12 meses, prof. de rehabilitación que lo atendió más: No sabe/No responde	0	No	305
			1	Sí	3
1231	n73	Últ. 12 meses, necesitó atención de rehabilitación y no recibió	1	Sí	106
			2	No	5.420
1232	n74	Principal razón por la que no recibió la atención de rehabilitación necesitada	1	No había disponibilidad del profesiona	42
			2	No podía pagar el costo de la atención	24
			3	Le suspendieron la atención o tratami	10
			4	Decidió no asistir debido a la pandemi	1
			5	Intentó conseguir asistencia de salud,	5
			6	Lo trataron mal o con negligencia ante	0
			7	No le pareció confiable o adecuada la	1
			8	Tuvo problemas para llegar al establec	2
			9	No podía ausentarse del trabajo o ten	0
			10	No lo consideró necesario	9
			77	Otra razón	12
1233	n75_1	Últ. 12 meses. ¿Ha sido discriminado…? Por ser hombre o mujer	1	Sí	138
			2	No	5.388
1234	n75_2	Últ. 12 meses. ¿Ha sido discriminado…? Identidad de género u orientación sexual	1	Sí	38
			2	No	5.488
1235	n75_3	Últ. 12 meses. ¿Ha sido discriminado…? Por su edad, o sea, por ser muy joven	1	Sí	132
			2	No	5.394
1236	n75_4	Últ. 12 meses. ¿Ha sido discriminado…? Por su forma de vestir o estilo	1	Sí	192
			2	No	5.334
1237	n75_5	Últ. 12 meses. ¿Ha sido discriminado…? Por su nacionalidad o la de sus padres	1	Sí	45
			2	No	5.481
1238	n75_6	Últ. 12 meses. ¿Ha sido discriminado…? Por su color de piel	1	Sí	78
			2	No	5.448
1239	n75_7	Últ. 12 meses. ¿Ha sido discriminado…? Por pertenecer a un pueblo indígena	1	Sí	24
			2	No	5.502
1240	n75_8	Últ. 12 meses. ¿Ha sido discriminado…? Por su cuerpo o apariencia física	1	Sí	424
			2	No	5.102
1241	n75_9	Últ. 12 meses. ¿Ha sido discriminado…? Por su situación económica	1	Sí	60
			2	No	5.466
1242	n75_10	Últ. 12 meses. ¿Ha sido discriminado…? Por el lugar en el que vive	1	Sí	65
			2	No	5.461
1243	n75_11	Últ. 12 meses. ¿Ha sido discriminado…? Por su religión o creencias religiosas	1	Sí	43
			2	No	5.483
1244	n75_12	Últ. 12 meses. ¿Ha sido discriminado…? Por su condición de salud o discapacidad	1	Sí	167
			2	No	5.359
1245	n75_13	Últ. 12 meses. ¿Ha sido discriminado…? Por su comportamiento	1	Sí	429
			2	No	5.097
1246	n75_14	Últ. 12 meses. ¿Ha sido discriminado…? Por sus opiniones o forma de pensar	1	Sí	368
			2	No	5.158
1247	n75_15	Últ. 12 meses. ¿Ha sido discriminado…? Por alguna dificultad de aprendizaje	1	Sí	262
			2	No	5.264
1248	n75_16	Últ. 12 meses. ¿Ha sido discriminado…? Por otro motivo	1	Sí	142
			2	No	5.384
1249	n76_1	Lugar de discriminación: En su familia	0	No	1.038
			1	Sí	127
1250	n76_2	Lugar de discriminación: En su centro de salud	0	No	1.131
			1	Sí	34

ID	Nombre variable	Etiqueta de variable	Valores	Etiquetas de valores	Frecuencia
1251	n76_3	Lugar de discriminación: En su barrio o vecindario	0	No	1.077
			1	Sí	88
1252	n76_4	Lugar de discriminación: En su establecimiento educacional	0	No	296
			1	Sí	869
1253	n76_5	Lugar de discriminación: En el transporte público	0	No	1.119
			1	Sí	46
1254	n76_6	Lugar de discriminación: En tiendas o comercio	0	No	1.126
			1	Sí	39
1255	n76_7	Lugar de discriminación: En instituciones públicas	0	No	1.143
			1	Sí	22
1256	n76_8	Lugar de discriminación: En la vía pública	0	No	1.082
			1	Sí	83
1257	n76_77	Lugar de discriminación: Otro lugar	0	No	1.062
			1	Sí	103
1258	n77	¿[NOM. NNA] se encuentra inscrito en el Registro Nacional de Discapacidad?	1	Sí	94
			2	No	5.432
1259	n79_1	Modalidad de enseñanza implementada: Clases presenciales	0	No	141
			1	Sí	5.146
1260	n79_2	Modalidad de enseñanza implementada: Clases online con interacción del profesor	0	No	5.100
			1	Sí	187
1261	n79_3	Modalidad de enseñanza implementada: Clases online sin interacción del profesor	0	No	5.273
			1	Sí	14
1262	n79_4	Modalidad de enseñanza implementada: Material académico guías y tareas	0	No	5.187
			1	Sí	100
1263	n79_77	Modalidad de enseñanza implementada: Otra	0	No	5.278
			1	Sí	9
1264	n80a	Últ. 12 meses, ¿producto del COVID-19…? Suspendido consultas médicas, dentales	-97	No aplica	76
			1	Sí	1.348
			2	No	4.102
1265	n80b	Últ. 12 meses, ¿producto del COVID-19…? Suspendido o postergado tratamientos	-97	No aplica	301
			1	Sí	345
			2	No	4.880
1266	n80c	Últ. 12 meses, ¿producto del COVID-19…? Suspendido participación en org.	-97	No aplica	319
			1	Sí	1.006
			2	No	4.201
1267	n81	Últ. 3 meses, ¿con qué frecuencia [NOM. NNA] se ha relacionado con otros NNA?	1	Casi todos los días	4.684
			2	Varias veces a la semana	502
			3	Al menos una vez a la semana	178
			4	Al menos una vez al mes	57
			5	Con menor frecuencia	105
1268	n82	Producto del COVID-19, ¿la interacción de [NOM. NNA] con otros NNA ha…?	1	Disminuido mucho	419
			2	Disminuido un poco	935
			3	No ha cambiado	2.643
			4	Aumentado un poco	719
			5	Aumentado mucho	810
1269	forma_ent_nna_rp_fin	Forma de finalización del cuestionario NNA - responsable principal	1	Respondió la encuesta por sí mismo(a	5.248
			2	Respondió la encuesta con ayuda de o	278
			3	Respondió la encuesta con ayuda del s	0
1270	forma_ent_nna_inicio	Forma de inicio del cuestionario NNA - autoaplicado	1	Responderá la encuesta por sí mismo(	2.308
			2	Responderá la encuesta con ayuda de	116
			3	Responderá la encuesta con ayuda del	0
1271	k1	En general, ¿cómo dirías que es tu salud?	1	Excelente	689
			2	Muy buena	699
			3	Buena	778
			4	Regular	242
			5	Mala	16
1272	k2	¿Te has sentido bien y en buen estado físico?	1	Nada	105
			2	Un poco	341
			3	Moderadamente	636
			4	Mucho	945
			5	Muchísimo	397
1273	k3	¿Has estado físicamente activo?	1	Nada	278
			2	Un poco	518

ID	Nombre variable	Etiqueta de variable	Valores	Etiquetas de valores	Frecuencia
			3	Moderadamente	579
			4	Mucho	630
			5	Muchísimo	419
1274	k4	¿Has sido capaz de correr sin dificultad?	1	Nada	308
			2	Un poco	302
			3	Moderadamente	408
			4	Mucho	797
			5	Muchísimo	609
1275	k5	¿Te has sentido lleno de energía?	1	Nada	59
			2	Un poco	174
			3	Moderadamente	724
			4	Mucho	850
			5	Muchísimo	617
1276	k6	¿Has disfrutado la vida?	1	Nada	40
			2	Un poco	177
			3	Moderadamente	467
			4	Mucho	899
			5	Muchísimo	841
1277	k7	¿Has estado de buen humor?	1	Nunca	26
			2	Casi nunca	86
			3	Algunas veces	634
			4	Casi siempre	1.126
			5	Siempre	552
1278	k8	¿Lo has pasado bien?	1	Nunca	16
			2	Casi nunca	43
			3	Algunas veces	437
			4	Casi siempre	998
			5	Siempre	930
1279	k9	¿Te has sentido triste?	1	Nunca	310
			2	Casi nunca	682
			3	Algunas veces	1.133
			4	Casi siempre	233
			5	Siempre	66
1280	k10	¿Te has sentido tan mal que no querías hacer nada?	1	Nunca	669
			2	Casi nunca	685
			3	Algunas veces	821
			4	Casi siempre	190
			5	Siempre	59
1281	k11	¿Te has sentido solo?	1	Nunca	979
			2	Casi nunca	591
			3	Algunas veces	593
			4	Casi siempre	184
			5	Siempre	77
1282	k12	¿Has estado contento con tu forma de ser?	1	Nunca	81
			2	Casi nunca	153
			3	Algunas veces	439
			4	Casi siempre	724
			5	Siempre	1.027
1283	k13	¿Has tenido suficiente tiempo para ti?	1	Nunca	30
			2	Casi nunca	140
			3	Algunas veces	497
			4	Casi siempre	823
			5	Siempre	934
1284	k14	¿Has podido hacer las cosas que has querido en tu tiempo libre?	1	Nunca	45
			2	Casi nunca	173
			3	Algunas veces	568
			4	Casi siempre	808
			5	Siempre	830
1285	k15	¿Tus padres han tenido suficiente tiempo para ti?	1	Nunca	33
			2	Casi nunca	123
			3	Algunas veces	444
			4	Casi siempre	760
			5	Siempre	1.064

ID	Nombre variable	Etiqueta de variable	Valores	Etiquetas de valores	Frecuencia
1286	k16	¿Tus padres te han tratado de forma justa?	1	Nunca	39
			2	Casi nunca	49
			3	Algunas veces	256
			4	Casi siempre	657
			5	Siempre	1.423
1287	k17	¿Has podido hablar con tus padres cuando lo has querido?	1	Nunca	49
			2	Casi nunca	146
			3	Algunas veces	373
			4	Casi siempre	591
			5	Siempre	1.265
1288	k18	¿Has tenido suficiente dinero para hacer las mismas cosas que tus amigos?	1	Nunca	147
			2	Casi nunca	256
			3	Algunas veces	728
			4	Casi siempre	654
			5	Siempre	639
1289	k19	¿Has tenido suficiente dinero para tus gastos personales?	1	Nunca	143
			2	Casi nunca	242
			3	Algunas veces	610
			4	Casi siempre	643
			5	Siempre	786
1290	k20	¿Has pasado tiempo con tus amigos?	1	Nunca	64
			2	Casi nunca	157
			3	Algunas veces	451
			4	Casi siempre	784
			5	Siempre	968
1291	k21	¿Lo has pasado bien con tus amigos?	1	Nunca	34
			2	Casi nunca	46
			3	Algunas veces	203
			4	Casi siempre	606
			5	Siempre	1.535
1292	k22	¿Tú y tus amigos se han ayudado entre ustedes?	1	Nunca	44
			2	Casi nunca	59
			3	Algunas veces	259
			4	Casi siempre	652
			5	Siempre	1.410
1293	k23	¿Has podido confiar en tus amigos?	1	Nunca	70
			2	Casi nunca	86
			3	Algunas veces	429
			4	Casi siempre	703
			5	Siempre	1.136
1294	k24	¿Te has sentido feliz en el colegio?	1	Nada	63
			2	Un poco	190
			3	Moderadamente	526
			4	Mucho	844
			5	Muchísimo	801
1295	k25	¿Te ha ido bien en el colegio?	1	Nada	36
			2	Un poco	178
			3	Moderadamente	785
			4	Mucho	905
			5	Muchísimo	520
1296	k26	¿Has sido capaz de poner atención?	1	Nunca	32
			2	Casi nunca	101
			3	Algunas veces	553
			4	Casi siempre	1.010
			5	Siempre	728
1297	k27	¿Te has llevado bien con tus profesores?	1	Nunca	22
			2	Casi nunca	56
			3	Algunas veces	354
			4	Casi siempre	865
			5	Siempre	1.127
1298	k28	¿Te has llevado bien con tus compañeros?	1	Nunca	30
			2	Casi nunca	68
			3	Algunas veces	346

ID	*Nombre variable*	*Etiqueta de variable*	*Valores*	*Etiquetas de valores*	*Frecuencia*
			4	Casi siempre	848
			5	Siempre	1.132
1299	nna1_a	Última semana: Compartir con tu familia	1	Sí	2.279
			2	No	145
1300	nna1_b	Última semana: Ver programas de televisión o videos elegidos por ti	1	Sí	2.270
			2	No	154
1301	nna1_c	Última semana: Escuchar radio o música elegida por ti	1	Sí	2.210
			2	No	214
1302	nna1_d	Última semana: Usar el computador, celular, tablet	1	Sí	2.355
			2	No	69
1303	nna1_e	Última semana: Usar redes sociales	1	Sí	2.208
			2	No	216
1304	nna1_f	Última semana: Leer libros, revistas o comics	1	Sí	1.495
			2	No	929
1305	nna1_g	Última semana: Dibujar, pintar, bailar, tocar algún instrumento	1	Sí	1.760
			2	No	664
1306	nna1_h	Última semana: Hacer deporte o actividad física	1	Sí	1.791
			2	No	633
1307	nna1_i	Última semana: Juntarse a jugar o conversar con tus amigos	1	Sí	2.041
			2	No	383
1308	nna2_a	Último mes: Ver obras de teatro, cine o espectáculos culturales	1	Sí	1.067
			2	No	1.357
1309	nna2_b	Último mes: Salir a comprar o vitrinear	1	Sí	1.870
			2	No	554
1310	nna2_c	Último mes: Disfrutar de la naturaleza	1	Sí	1.908
			2	No	516
1311	nna3_a	Participación: Club o taller donde practicas un deporte o actividad física	1	Sí	1.113
			2	No	1.311
1312	nna3_b	Participación: Club religioso, pastoral o iglesia	1	Sí	501
			2	No	1.923
1313	nna3_c	Participación: Grupo artístico o cultural	1	Sí	444
			2	No	1.980
1314	nna3_d	Participación: Grupo de scouts	1	Sí	115
			2	No	2.309
1315	nna3_e	Participación: Centro de alumnos o directiva de tu curso	1	Sí	527
			2	No	1.897
1316	nna3_f	Participación: Otro tipo de grupo. ¿Cuál?	1	Sí	255
			2	No	2.169
1317	nna3_f_esp	Participación: Especifique el otro grupo donde participa	Válidos		255
1318	nna4_a	Últ. 12 meses. ¿Ha sido discriminado…? Por ser hombre o mujer	1	Sí	195
			2	No	2.229
1319	nna4_b	Últ. 12 meses. ¿Ha sido discriminado…? Por tu identidad de género u orientación	1	Sí	82
			2	No	2.342
1320	nna4_c	Últ. 12 meses. ¿Ha sido discriminado…? Por tu edad, o sea, por ser muy joven	1	Sí	220
			2	No	2.204
1321	nna4_d	Últ. 12 meses. ¿Ha sido discriminado…? Por tu forma de vestir o tu estilo	1	Sí	299
			2	No	2.125
1322	nna4_e	Últ. 12 meses. ¿Ha sido discriminado…? Por tu nacionalidad o la de tus padres	1	Sí	53
			2	No	2.371
1323	nna4_f	Últ. 12 meses. ¿Ha sido discriminado…? Por tu color de piel	1	Sí	89
			2	No	2.335
1324	nna4_g	Últ. 12 meses. ¿Ha sido discriminado…? Por pertenecer a un pueblo indígena	1	Sí	29
			2	No	2.395
1325	nna4_h	Últ. 12 meses. ¿Ha sido discriminado…? Por tu cuerpo o tu apariencia física	1	Sí	381
			2	No	2.043
1326	nna4_i	Últ. 12 meses. ¿Ha sido discriminado…? Por tu situación económica	1	Sí	62
			2	No	2.362
1327	nna4_j	Últ. 12 meses. ¿Ha sido discriminado…? Por el lugar en el que vives	1	Sí	70
			2	No	2.354
1328	nna4_k	Últ. 12 meses. ¿Ha sido discriminado…? Por tu religión o creencias religiosas	1	Sí	59
			2	No	2.365
1329	nna4_l	Últ. 12 meses. ¿Ha sido discriminado…? Por tu condición de salud o discapacidad	1	Sí	63
			2	No	2.361

ID	Nombre variable	Etiqueta de variable	Valores	Etiquetas de valores	Frecuencia
1330	nna4_m	Últ. 12 meses. ¿Ha sido discriminado…? Por tu comportamiento	1	Sí	377
			2	No	2.047
1331	nna4_n	Últ. 12 meses. ¿Ha sido discriminado…? Por tus opiniones o forma de pensar	1	Sí	411
			2	No	2.013
1332	nna4_o	Últ. 12 meses. ¿Ha sido discriminado…? Por alguna dificultad de aprendizaje	1	Sí	301
			2	No	2.123
1333	nna4_p	Últ. 12 meses. ¿Ha sido discriminado…? Por otro motivo. ¿Cuál?	1	Sí	56
			2	No	2.368
1334	nna4_p_esp	Últ. 12 meses. ¿Ha sido discriminado…? Especifique otro motivo	Válidos		56
1335	forma_ent_nna_fin	Forma de finalización del cuestionario NNA - autoaplicado	1	Respondió la encuesta por sí mismo(a	2.310
			2	Respondió la encuesta con ayuda de o	114
			3	Respondió la encuesta con ayuda del s	0
1336	tot_per_hogar	Total de personas en el hogar	Rango: 1 - 14		
1337	esc	Escolaridad - 15 años o más	Rango: 0 - 32		
1338	educ	Nivel de escolaridad	-88	No sabe	621
			0	Sin educación formal	6.186
			1	Básica incompleta	15.619
			2	Básica completa	7.634
			3	Media humanista incompleta	8.945
			4	Media técnica profesional incompleta	1.767
			5	Media humanista completa	16.618
			6	Media técnica profesional completa	4.838
			7	Técnico nivel superior incompleto	2.799
			8	Técnico nivel superior completo	4.673
			9	Profesional incompleto	6.048
			10	Postgrado incompleto	7.105
			11	Profesional completo	249
			12	Postgrado completo	645
1339	educc	Nivel de escolaridad - Agregado	-88	No sabe	621
			0	Sin educación formal	6.186
			1	Básica incompleta	15.619
			2	Básica completa	7.634
			3	Media incompleta	10.712
			4	Media completa	21.456
			5	Superior incompleta	8.847
			6	Superior completa	12.672
1340	media_no_terminada	Educación media no terminada	0	Terminada	43.188
			1	No terminada	40.388
1341	activ	Condición de actividad	1	Ocupados	34.136
			2	Desocupados	2.947
			3	Inactivos	32.136
1342	cat_ocup	Categoría ocupacional abreviada	1	Patrón o empleador	546
			2	Cuenta propia	4.396
			3	Asalariado	10.875
			4	Familiar no remunerado	60
1343	yaut	Ingreso autónomo	Rango: 0 - 21.500.000		
1344	yautcor	Ingreso autónomo corregido	Rango: 0 - 21.500.000		
1345	ysub1	Subsidios y transferencias (sin IFE)	Rango: 232 - 6.000.000		
1346	ysub	Subsidios y transferencias	Rango: 0 - 6.000.000		
1347	ytot	Ingreso total	Rango: 0 - 21.500.000		
1348	ytotcor	Ingreso total corregido	Rango: 0 - 21.500.000		
1349	yauth	Ingreso autónomo del hogar	Rango: 0 - 27.465.000		
1350	yautcorh	Ingreso autónomo corregido del hogar	Rango: 0 - 27.465.000		
1351	ysub1h	Subsidios y transferencias (sin IFE) del hogar	Rango: 0 - 6.000.000		
1352	ysubh	Subsidios y transferencias del hogar	Rango: 0 - 6.000.000		
1353	ytoth	Ingreso total del hogar	Rango: 0 - 28.049.242		
1354	ytotcorh	Ingreso total corregido del hogar	Rango: 0 - 28.049.242		
1355	ypchautcor	Ingreso autónomo corregido per cápita del hogar	Rango: 0 - 10.000.000		
1356	ypchsubcor	Ingreso subsidios corregido per cápita del hogar	Rango: 0 - 3.000.000		
1357	dau	Decil de ingreso autónomo per cápita	Rango: 1 - 10		

ID	Nombre variable	Etiqueta de variable	Valores	Etiquetas de valores	Frecuencia
1358	qaut	Quintil de ingreso autónomo per cápita	Rango: 1 - 5		
1359	mefo_ptje	MEFO: prueba cribado de deterioro cognitivo (puntaje) - personas mayores	Rango: 0 - 13		
1360	n_cuidadores	Número de cuidadores(as) del adulto seleccionado	Rango: 0 - 13		
1361	n_cuidadores_hogar	Número de cuidadores del adulto seleccionado que son parte de su hogar	Rango: 0 - 5		
1362	disp_cuidadores	Disponibilidad y residencia de los cuidadores(as) del adulto entrevistado(a)	1	No tiene cuidador(a)	27.073
			2	Solo tiene cuidador(a) dentro del hoga	2.022
			3	Solo tiene cuidador(a) fuera del hogar	530
			4	Tiene cuidador(a) dentro y fuera del h	385
1363	id_col_cuidador_principal	Identificación cuidador(a) principal del adulto entrevistado(a) (num. columna)	Rango: 0 - 13		
1364	res_cuidador_principal	Residencia del cuidador(a) principal del adulto entrevistado(a)	1	Sí	2.309
			2	No	628
1365	sexo_cuidador_principal	Sexo del cuidador(a) principal del adulto entrevistado(a)	1	Hombre	849
			2	Mujer	2.084
1366	edad_cuidador_principal	Edad del cuidador(a) principal del adulto entrevistado(a)	Rango: 6 - 99		
1367	paren_cuidador_principal	Relación de parentesco del cuidador(a) principal con el adulto entrevistado(a)	1	Cónyuge o conviviente	877
			2	Hijo(a) o hijastro(a)	1.071
			3	Yerno o nuera	59
			4	Nieto(a)	94
			5	Hermano(a) o cuñado(a)	180
			6	Padres o suegros	301
			7	Abuelo(a)	14
			8	Otro pariente	123
			9	Vecino(a)	30
			10	Amigo(a)	64
			11	Servicio doméstico puertas adentro	16
			12	Servicio personal de salud	22
			77	Otro no pariente	86
1368	rem_cuidador_principal	Recepción de remuneración del cuidador(a) principal del adulto entrevistado(a)	1	Sí	161
			2	No	2.776
1369	id_col_cuidador_principal_hog	Identificación cuidador(a) principal hogar del adulto entrevistad	Rango: 1 - 5		
1370	id_cuidador_principal_hogar	Identificación cuidador(a) principal hogar del adulto entrevistado/a (n orden)	Rango: 1 - 10		
1371	sexo_cuidador_principal_hoga	Sexo del cuidador(a) principal del hogar del adulto entrevistado(a)	1	Hombre	781
			2	Mujer	1.626
1372	edad_cuidador_principal_hog	Edad del cuidador(a) principal del hogar del adulto entrevistado(a)	Rango: 6 - 99		
1373	paren_cuidador_principal_hog	Relación de parentesco cuidador(a) principal hogar con el adulto entrevistado(a)	1	Cónyuge o conviviente	886
			2	Hijo(a) o hijastro(a)	861
			3	Yerno o nuera	38
			4	Nieto(a)	83
			5	Hermano(a) o cuñado(a)	138
			6	Padres o suegros	275
			7	Abuelo(a)	13
			8	Otro pariente	75
			10	Amigo(a)	5
			11	Servicio doméstico puertas adentro	8
			77	Otro no pariente	25
1374	rem_cuidador_principal_hoga	Recepción de remuneración cuidador(a) principal hogar del adulto entrevistado(a)	1	Sí	69
			2	No	2.338
1375	cap_puntaje_adulto	Puntaje dificultad en métrica capacidad (0 a 100) - personas adultas	Rango: 0,0 - 100,0		
1376	des_puntaje_adulto	Puntaje dificultad en métrica desempeño (0 a 100) - personas adultas	Rango: 0,0 - 100,0		
1377	cap_nivel_adulto	Nivel de dificultad en capacidad - personas adultas	1	Sin dificultad	6.909
			2	Dificultad leve	5.979
			3	Dificultad moderada	12.129
			4	Dificultad severa	4.993
1378	des_nivel_adulto	Nivel de dificultad en desempeño - personas adultas	1	Sin dificultad	7.057
			2	Dificultad leve	9.649
			3	Dificultad moderada	7.532

ID	*Nombre variable*	*Etiqueta de variable*	*Valores*	*Etiquetas de valores*	*Frecuencia*
			4	Dificultad severa	5.772
1379	disc_grado_adulto	Niveles de discapacidad - personas adulta	1	Sin discapacidad	24.238
			2	Discapacidad leve a moderada	1.855
			3	Discapacidad severa	3.917
1380	disc_adulto	Condición de discapacidad - personas adultas	0	Sin discapacidad	24.238
			1	Con discapacidad	5.772
1381	depen_grado_adulto	Niveles de dependencia - personas adultas	1	Sin dependencia	26.722
			2	Dependencia leve	1.071
			3	Dependencia moderada	1.248
			4	Dependencia severa	969
1382	depen_adulto	Situación de dependencia - personas adultas	0	Sin dependencia	26.722
			1	Con dependencia	3.288
1383	depen_edpm	Dependencia funcional personas 60 años y más: proxy EDPM 2009	1	Sin dependencia	6.558
			2	Dependencia leve	490
			3	Dependencia moderada	364
			4	Dependencia severa	1.759
1384	id_adulto_cui	Identificación del adulto entrevistado/a asistido (n orden)	Rango: 1 - 10		
1385	edad_n55	Edad cuidador principal que presta asistencia al NNA (declarado en n55)	Rango: 17 - 85		
1386	sexo_n55	Sexo cuidador principal que presta asistencia al NNA (declarado en n55)	1	Hombre	34
			2	Mujer	321
1387	edad_n58	Edad consolidada segundo cuidador que asiste al NNA (declarado en n58)	Rango: 13 - 94		
1388	sexo_n58	Sexo consolidado segundo cuidador que asiste al NNA (declarado en n58)	1	Hombre	95
			2	Mujer	100
1389	cap_puntaje_nna	Puntaje dificultad en métrica capacidad (0 a 100) - NNA	Rango: 0 - 84,75		
1390	cap_nivel_nna	Nivel de dificultad en capacidad - NNA	1	Sin dificultad	2.595
			2	Dificultad leve	16
			3	Dificultad moderada	2.116
			4	Dificultad severa	799
1391	des_puntaje_nna	Puntaje dificultad en métrica desempeño (0 a 100) - NNA	Rango: 0 - 60,25		
1392	des_nivel_nna	Nivel de dificultad en desempeño - NNA	1	Sin dificultad	1.412
			2	Dificultad leve	1.179
			3	Dificultad moderada	2.133
			4	Dificultad severa	802
1393	disc_nna	Condición de discapacidad - NNA	0	Sin discapacidad	4.724
			1	Con discapacidad	802
1394	disc_grado_nna	Niveles de discapacidad - NNA	1	Sin discapacidad	4.724
			2	Discapacidad leve a moderada	255
			3	Discapacidad severa	547
